# Exploring the Role of Ferroptosis in the Pathophysiology and Circadian Regulation of Restless Legs Syndrome

**DOI:** 10.3390/biom15081184

**Published:** 2025-08-18

**Authors:** Maria Paola Mogavero, Giovanna Marchese, Giovanna Maria Ventola, Giuseppe Lanza, Oliviero Bruni, Luigi Ferini-Strambi, Raffaele Ferri

**Affiliations:** 1 Vita-Salute San Raffaele University, 20132 Milan, Italy; paola_mogavero@libero.it (M.P.M.); ferinistrambi.luigi@hsr.it (L.F.-S.); 2 Sleep Disorders Center, Division of Neuroscience, San Raffaele Scientific Institute, 20127 Milan, Italy; 3 Genomix4Life Srl, 84081 Baronissi, Italy; giovanna.marchese@genomix4life.com (G.M.); giovanna.ventola@genomix4life.com (G.M.V.); 4 Genome Research Center for Health-CRGS, 84081 Baronissi, Italy; 5 Oasi Research Institute-IRCCS, 94018 Troina, Italy; glanza@oasi.en.it; 6 Department of Surgery and Medical-Surgical Specialties, University of Catania, 95123 Catania, Italy; 7 Department of Human Neuroscience, Sapienza University of Rome, 00185 Rome, Italy; oliviero.bruni@uniroma1.it

**Keywords:** ferroptosis, restless legs syndrome, circadian rhythms, oxidative stress, iron metabolism

## Abstract

The study objectives were to investigate the role of ferroptosis, the mechanism linking iron accumulation, oxidative stress, and dopaminergic dysfunction, in restless legs syndrome (RLS), and to explore its connection with circadian regulation, a key feature of RLS and a known modulator of ferroptosis. We conducted pathway and gene expression analyses in 17 RLS patients and 39 controls, focusing on pathways related to ferroptosis, oxidative stress, iron metabolism, dopaminergic signaling, circadian rhythms, and immune responses. Enrichment analysis, differential gene expression, and cross-pathway gene overlaps were assessed. Ferroptosis and efferocytosis pathways were significantly upregulated in RLS, while oxidative phosphorylation, phosphatidylinositol signaling, PI3K-Akt, FoxO, and adipocytokine pathways were downregulated. The circadian rhythm pathway was markedly suppressed, with 12 circadian genes downregulated, suggesting that circadian disruption may drive ferroptosis activation. Decreased expression of protective pathways, including antioxidant responses and autophagy, was associated with increased iron accumulation, oxidative stress, and inflammation. Dopaminergic synapse genes were upregulated, possibly as a compensatory response to neuronal damage. Several genes overlapped across ferroptosis, circadian, and dopaminergic pathways, indicating a shared pathogenic mechanism. Our findings support a model in which circadian disruption promotes ferroptosis in RLS, contributing to iron overload, oxidative damage, and dopaminergic dysfunction. This pathogenic cascade may also enhance immune activation and inflammation. Circadian regulation and ferroptosis emerge as promising therapeutic targets in RLS. Further studies in larger cohorts are warranted to validate these mechanistic insights.

## 1. Introduction

Restless legs syndrome (RLS) is a sensorimotor disorder whose symptoms, at any frequency or severity, affect approximately 5% to 10% of the general population in Western industrialized countries [1,2,3]. The condition is characterized by an irresistible urge to move the legs, often accompanied by unpleasant sensations. These symptoms typically begin or worsen during periods of rest or inactivity, especially in the evening or at night, and are partially or completely relieved by movement. Importantly, they are not attributable to other medical conditions [4].

RLS is clinically heterogeneous, with symptom expression varying by sex and age, which may also affect changes in time structure of periodic limb movements during sleep (PLMS) and the response to dopaminergic drugs [5,6,7]. However, a consistent and defining feature across phenotypes is its circadian pattern, with symptoms predominantly emerging in the evening and night [8].

Although the etiopathogenesis of RLS remains under investigation, current evidence points to the involvement of central nervous system structures, iron metabolism, and the dopaminergic system. Other neurotransmitter systems, including glutamatergic [9], GABAergic, and cholinergic pathways [8,10] have also been implicated. Notably, recent studies highlight the contribution of the mesolimbic dopaminergic pathway, as well as the basal ganglia, hippocampus, and amygdala, in RLS pathophysiology [11,12].

Several converging theories have been proposed to explain RLS pathogenesis, including central dopaminergic dysfunction, impaired brain iron homeostasis, increased glutamatergic transmission, and neuroinflammatory processes [8]. These mechanisms are not mutually exclusive and may act synergistically, with iron deficiency leading to altered dopamine synthesis and oxidative stress, further exacerbated by excitatory neurotransmission and immune activation.

Given the complex interplay of neural circuits and neurotransmitters involved, the origins of RLS circadianity are not fully understood. However, dopaminergic transmission is believed to play a central role. Symptom onset and worsening typically coincide with the circadian nadir of dopamine levels, while symptom relief occurs as dopamine levels normalize in the morning [8]. Moreover, this pattern is mirrored by PLMS, which occur in up to 80–90% of RLS patients [8]. The timing of PLMS, peaking from sleep onset to early morning, when dopamine levels are lowest, may help as a distinguishing feature of RLS compared to PLMS in other sleep disorders [13].

A recent transcriptomic analysis identified autophagy-related mechanisms, particularly ferroptosis, as key pathways in RLS [10]. Ferroptosis not only influences dopaminergic signaling but is also closely tied to iron metabolism [14,15,16], both of which are critical to RLS pathogenesis and treatment [17].

Despite these findings, the molecular basis for the circadian nature of RLS remains largely unresolved. Emerging evidence suggests that ferroptosis may itself be modulated by circadian genes [18], raising the possibility of a regulatory link between circadian molecular rhythms and the neurodegenerative processes observed in RLS. This intersection is particularly compelling, as it may explain how fluctuations in gene expression over the 24 h cycle influence dopaminergic vulnerability and symptom timing.

One emerging focus in RLS research is the role of the A11 dopaminergic nucleus, the primary source of descending dopaminergic input to the spinal cord [19]. Lesions or dysfunctions in the A11 region have been shown to induce RLS-like symptoms in animal models, suggesting its critical role in sensorimotor integration and dopaminergic control of spinal excitability [20]. While direct evidence of clock gene expression specifically in A11 neurons remains limited, recent findings demonstrate rhythmic expression of core circadian genes such as *PER1* and *BMAL1* in the posterior hypothalamus, which encompasses the A11 region [21]. This suggests that A11 neurons may be influenced by local circadian gene activity and potentially act as a relay between central circadian networks and motor regulatory circuits. Such a dopaminergic-circadian interaction could provide a mechanistic bridge linking circadian timing and RLS symptomatology, particularly in relation to spinal excitability and the expression of PLMS.

The hypothalamus, a central node of circadian control, has also gained attention in RLS pathophysiology. Functional imaging and clinical studies suggest altered activity in hypothalamic regions involved in arousal and sleep regulation, including the suprachiasmatic nucleus (SCN) and lateral hypothalamus [12,19,22]. Dysregulation in these regions may influence both dopaminergic and orexinergic pathways, thereby contributing to the characteristic evening-worsening of RLS symptoms and their disruption of sleep. Furthermore, the SCN regulates peripheral clocks [23], which can modulate iron transport and metabolism, further linking circadian mechanisms with key pathological features of RLS.

Therefore, the aim of this study is to perform an in-depth transcriptomic analysis to investigate the interplay between circadian gene networks and ferroptosis-related pathways in RLS. Specifically, we seek to explore the role of ferroptosis, the mechanism linking iron accumulation, oxidative stress, and dopaminergic dysfunction, in the context of RLS, where these processes are also implicated. We further examine the connection between ferroptosis, RLS, and circadian regulation, given the established interplay between ferroptosis and circadian rhythms, and the fact that circadian disruption is a defining feature of RLS. By integrating high-resolution gene expression data, we aim to uncover novel molecular mechanisms driving RLS and identify potential targets for future therapeutic strategies.

## 2. Materials and Methods

Raw sequencing data for RLS patients and controls were obtained from datasets E-MTAB-13155 and E-MTAB-11326, respectively. A total of 17 RLS (13 women and 4 men, with an age range 24–76 years, mean age 55.8 years, a mean International RLS Study Group rating scale [24] of 21.1 and a mean disease duration of 6.6 years) were included in the analysis, along with 39 controls (22 women and 17 men, with an age range 23–92 years, mean age 62.9 years).

Two patients reported a family history of RLS. The diagnosis was established based on the criteria of the International RLS Study Group [4], using a semi-structured clinical interview designed to rigorously exclude conditions that may mimic RLS. Exclusion criteria included the presence of other sleep disorders, psychiatric, neurological, cardiovascular, or neurodegenerative conditions, neurodevelopmental delay, use of central nervous system-active medications within the year prior to the study, or any pharmacological treatment within three weeks before the polysomnographic recording. Participants with an apnea–hypopnea index greater than 10 events per hour of sleep were also excluded. Control subjects were free from medication and had no history of sleep, neurological, psychiatric, or physical disorders. Written informed consent was obtained in accordance with the Declaration of Helsinki, and the study protocol was approved by the Ethics Committee of the Oasi Research Institute.

The RNA-seq pipeline followed was previously described in 2024 by Mogavero et al. [10]. For all samples, from both controls and patients, venous blood was drawn in the morning following a 12 h fast. Immediately after collection, the samples were processed using Ficoll-Paque density gradient centrifugation (Ficoll-Paque PLUS-GE Healthcare Life Sciences, Piscataway, NJ, USA), and peripheral blood mononuclear cells (PBMCs) were stored at −80 °C until RNA extraction. RNA was isolated using TRIzol reagent (TRIzol Reagent, Invitrogen Life Technologies, Carlsbad, CA, USA) according to the manufacturer’s instructions. All samples were handled, stored, and processed using standardized procedures. RNA extraction was performed simultaneously for samples within the same group. The extracted RNA was stored at −80 °C until further analysis. A DNase treatment was applied after RNA isolation to ensure sample purity and enable accurate quantification.

Before proceeding with the protocol, RNA yield and quality were assessed using a NanoDrop One spectrophotometer (NanoDrop Technologies LLC, Wilmington, DE, USA) and a TapeStation 4200 (Agilent Technologies, 5301 Stevens Creek Blvd, Santa Clara, CA, USA), respectively. Specifically, the 260/280 absorbance ratio, as measured by NanoDrop, ranged from 1.8 to 2.0, while the RNA Integrity Number (RIN), as determined by the TapeStation, ranged from 6 to 8.

RNA extraction, library preparation, and sequencing were all performed using a standardized protocol, with consistent procedures applied within the same laboratory. Specifically, indexed libraries for all samples were prepared using the Illumina Stranded mRNA Prep Kit (Illumina, San Diego, CA, USA) according to the manufacturer’s instructions. Following mRNA enrichment and fragmentation, cDNA synthesis, adapter ligation, and PCR amplification were carried out. Library concentrations were measured using the TapeStation 4200 (Agilent Technologies) and the Qubit 4 Fluorometer (Thermo Fisher Scientific, Waltham, MA, USA). Equimolar amounts of the indexed libraries were pooled and sequenced on the Illumina platform using a 2 × 75 bp paired-end read configuration.

Gene expression quantification was performed using the featureCounts tool, aligning reads to the GRCh38 human reference genome and GENCODE Version 37 annotations, via the STAR aligner with standard parameters. Quantification of expressed genes for each sample was computed using the featureCounts algorithm.

Data normalization was conducted in Rversion 4.4.2 using negative binomial generalized linear models, implemented through the Bioconductor DESeq2 version 1.49age. Only genes expressed in ≥25% of samples were included in the analysis. This model incorporates both the mean expression level and a gene-specific dispersion parameter, allowing for robust estimation of differential expression between conditions.

The DESeq2 package applies the Benjamini–Hochberg (BH) correction for multiple testing to calculate adjusted *p*-values (*p*adj), using default parameters. The BH correction controls the false discovery rate (FDR), which is the expected proportion of false positives among the results deemed statistically significant.

Genes were considered differentially expressed if they exhibited a fold change ≥ 1.50 or ≤−1.50 (|FC| ≥ 1.50) and an adjusted *p*-value (*p*adj) ≤ 0.01.

For functional analysis, pathway enrichment was conducted using the pathfindR version 2.4.2in R, leveraging the Kyoto Encyclopedia of Genes and Genomes (KEGG) database. This approach enabled the identification of biological pathways significantly associated with the observed gene expression changes.

Principal Component Analysis (PCA) was performed to assess overall variability among samples and to detect potential outliers or group-specific clustering patterns. PCA plots, along with volcano plots, used to visualize the distribution of regulated genes in terms of statistical significance and magnitude of fold change, were generated using the ggplot2 version 3.5.1 in R. Heatmaps of the differentially expressed genes were generated using the ComplexHeatmap version 2.14.0

## 3. Results

**Figure 1:**
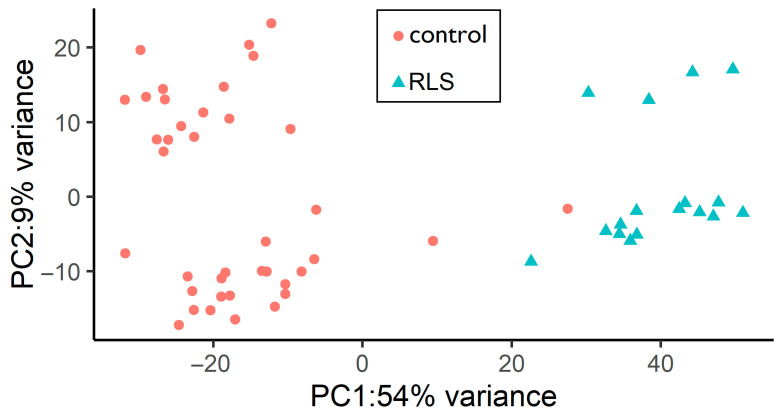
Principal Component Analysis (PCA) of RLS and control samples showing distinct clustering of the two groups based on the first principal component (PC1) and second principal component (PC2), explaining 54% of the variance.

The first step involved assessing sample distribution using PCA, which revealed a clear separation between RLS and control groups along the first two principal components, indicating systematic transcriptomic differences (Figure 1).

**Figure 2:**
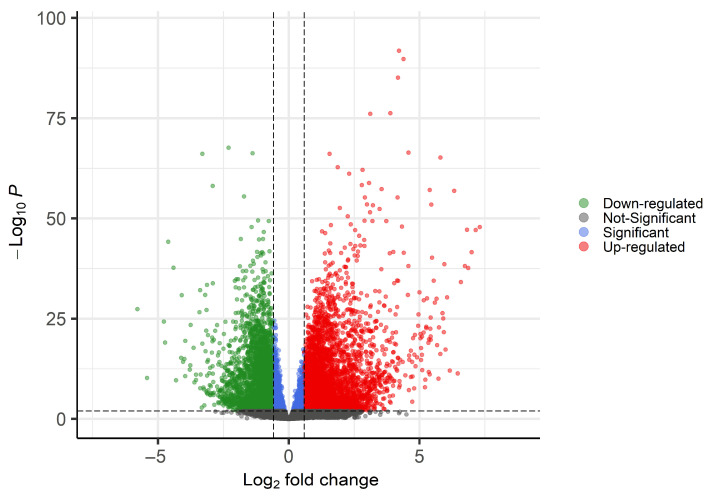
Volcano plot of differentially expressed genes between RLS and control samples. Upregulated genes are shown in red, downregulated genes in green, non-significant genes (*p*adj > 0.01) in gray, and genes significant only for *p*adj ≤ 0.01 in blue.

We detected 23,331 expressed genes across all samples (expressed in at least 25% of RLS and control samples). Differential expression analysis identified 10,185 differentially expressed genes (*p*adj ≤ 0.01). Among these, 4008 genes were significantly upregulated (*p*adj ≤ 0.01 and fold-change ≥ 1.5), and 3413 were significantly downregulated (*p*adj ≤ 0.01 and fold-change ≤ –1.5) in RLS samples compared to controls (Supplementary Table S1). The volcano plot illustrates the distribution of differentially expressed genes between groups (Figure 2).

Pathway enrichment analysis using pathfindR revealed 228 enriched KEGG pathways. We focused our analysis on 12 pathways of particular relevance, selected for their functional connections to ferroptosis, oxidative phosphorylation, and circadian clock genes, three critical biological axes involved in the regulation of oxidative stress and neuronal survival, particularly within the dopaminergic system.

All selected pathways were significantly enriched (*p*adj < 0.05), with fold enrichment values ranging from 1.09 for Oxidative Phosphorylation to 2.89 for Circadian Rhythm, as shown in Table 1.

Among these, the ferroptosis pathway (hsa04216) may represent a particularly relevant mechanism potentially implicated in RLS, given its role in iron-mediated oxidative damage and its potential contribution to disease pathophysiology.

As ferroptosis is closely linked to oxidative stress and redox regulation, we extended our analysis to pathways such as oxidative phosphorylation (hsa00190) and chemical carcinogenesis—reactive oxygen species (ROS) (hsa05208), both critical for understanding oxidative stress-mediated injury.

Oxidative stress and iron dysregulation appear to intersect with circadian rhythm control (hsa04710, hsa04713), influencing cellular energy metabolism and susceptibility to ferroptosis. Disruptions in circadian rhythms may impair antioxidant defense systems, promoting ROS accumulation and cell death.

Additional pathways, FoxO signaling (hsa04068), PI3K-Akt (hsa04151), autophagy (hsa04140), and adipocytokine signaling (hsa04920), were also analyzed for their roles in stress responses, cell survival, and inflammation, all of which are relevant to ferroptosis susceptibility.

**Figure 3:**
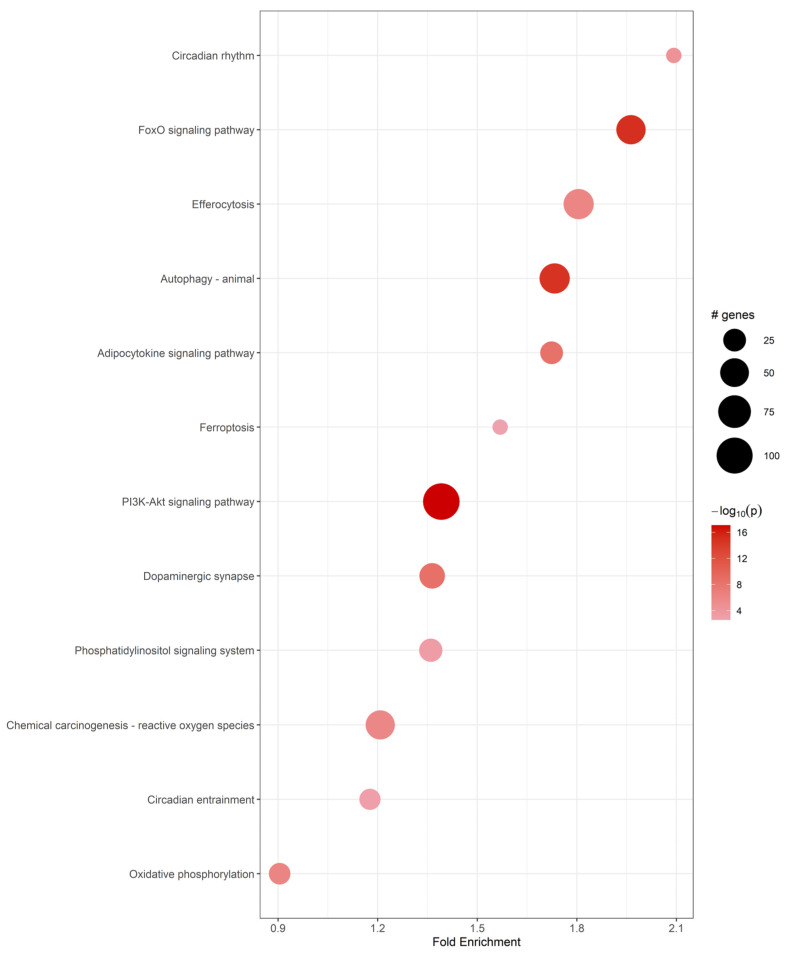
Summary of pathway enrichment results. Graphical representation of 12 selected enriched pathways based on differentially expressed genes.

The phosphatidylinositol signaling system (hsa04070) is highlighted for its involvement in regulating iron metabolism and oxidative damage responses. Autophagy (hsa04140) plays a protective role by removing excess iron and damaged cellular components, while the dopaminergic synapse pathway (hsa04728) is crucial for brain function and is notably affected in RLS. Dysregulation of iron metabolism in dopaminergic neurons may increase susceptibility to ferroptosis activation, thereby contributing to the progression and exacerbation of neurodegenerative processes. Additionally, the efferocytosis pathway (hsa04148), the mechanism by which immune cells clear apoptotic or damaged cells, appears to play a protective role by mitigating oxidative damage. (Figure 3, Table 1).

**Figure 4:**
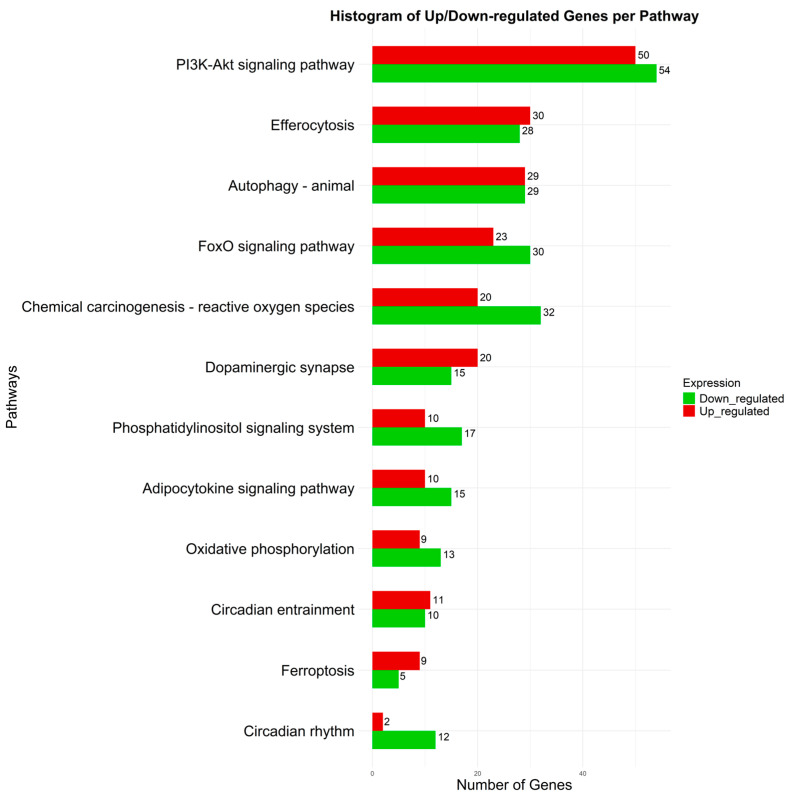
Histogram showing the number of upregulated (red) and downregulated (green) genes contributing to the enrichment of the 12 selected biological pathways.

Shared genes among these pathways suggest a common pathogenic thread. Figure 4 displays a histogram of upregulated (red) and downregulated (green) genes for each biological pathway. In the Chemical Carcinogenesis–ROS pathway, there was a predominance of downregulated genes (32) versus upregulated ones (20), suggesting a suppressed oxidative stress response. Although this may reflect an adaptive mechanism to limit harmful oxidative pathways, it could also impair the cell’s ability to neutralize ROS, favoring sustained oxidative stress and the initiation of ferroptosis.

The Circadian Rhythm pathway shows a strong imbalance, with only 2 upregulated and 12 downregulated genes, a marked downregulation of the genes involved in this pathway. The concurrent enrichment of pathways related to energy metabolism, the circadian clock, and ferroptosis suggests the existence of an integrated biological network in which oxidative stress, iron dysregulation, and disruption of circadian rhythms converge to increase neuronal vulnerability.

Downregulation of core circadian clock genes (*PER1-3*, *CLOCK*, *RORA*), which are essential for maintaining the rhythmicity of antioxidant defenses, may impair cellular homeostasis and heighten susceptibility to ferroptosis.

This network appears to be further modulated by key regulatory pathways, including FoxO signaling, PI3K-Akt, and autophagy, which orchestrate cellular stress responses and the clearance of damaged organelles and proteins.

Finally, the involvement of the dopaminergic synapse pathway suggests potential alterations in synaptic function and neuronal clearance mechanisms, both of which are critical to the pathophysiology of RLS.

**Figure 5:**
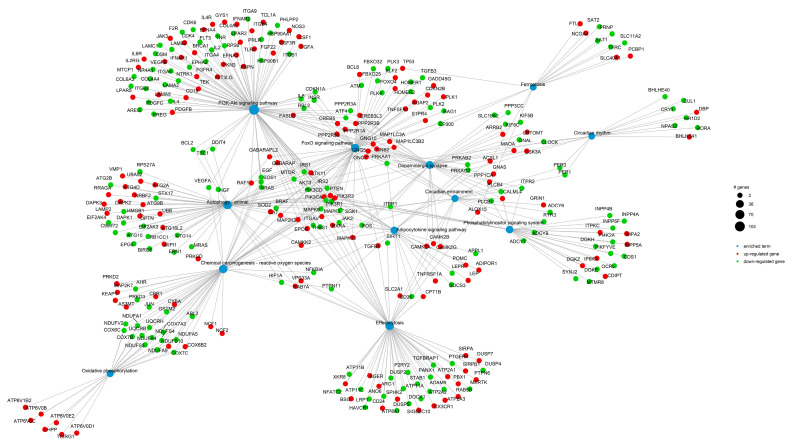
Network diagram illustrating the relationships between upregulated and downregulated genes and the 12 selected enriched KEGG pathways. Each node represents a KEGG pathway; red and green nodes indicate pathways with predominantly upregulated or downregulated gene sets, respectively. The complexity of the network reflects the multifactorial nature of RLS pathophysiology and the high degree of gene overlap among pathways. While dense, the full network was retained to preserve the biological detail and interconnectedness revealed by the transcriptomic data.

In Figure 5, we show a network representation of pathway interactions, illustrating how gene expression changes are integrated across pathways. This visualization highlights the interplay between biological processes and the complexity of signaling alterations in RLS.

**Figure 6:**
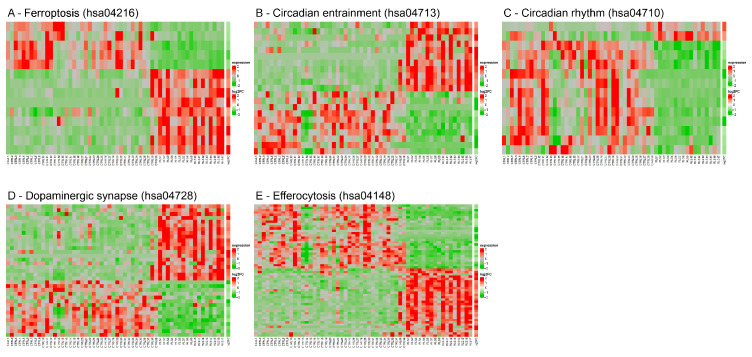
Heatmap of (**A**) ferroptosis-related gene expression; (**B**) circadian entrainment pathway gene expression; (**C**) circadian rhythm pathway gene expression; (**D**) dopaminergic synapse pathway gene expression; and (**E**) efferocytosis pathway gene expression, across all RLS and controls (CTRL). samples.

In Figure 6A–E, we focus on key pathways including ferroptosis (hsa04216), circadian entrainment (hsa04713), circadian rhythm (hsa04710), dopaminergic synapse (hsa04728), and efferocytosis (hsa04148), respectively, analyzing gene expression variations between controls and RLS patients.

Hierarchical clustering analysis reveals consistent and statistically significant expression differences (*p*adj ≤ 0.01 and |FC| ≥ 1.5). RLS samples exhibited a clear upregulation pattern (red) compared to controls (green/neutral), particularly for ferroptosis-related genes. This heatmap strongly supports the hypothesis that ferroptosis pathways are activated or dysregulated in RLS.

## 4. Discussion

Our research investigated the role of ferroptosis in RLS, a mechanism recently implicated in the disorder [10], also considering its crucial involvement in inflammation and oxidative stress [14,15], both of which have been increasingly linked to RLS [25,26]. Moreover, ferroptosis is known to contribute to intracellular dysregulation of iron metabolism [14] and to impact dopaminergic metabolism [18], both of which are highly relevant to the pathogenesis of RLS [8,27].

Given that circadian dysregulation is a hallmark of RLS [4], we also explored potential connections between ferroptosis-related mechanisms (oxidative phosphorylation, phosphatidylinositol signaling, FoxO, PI3K-Akt and adipocytokine pathways, autophagy, efferocytosis), circadian regulation, and the dopaminergic system. Our analyses revealed that multiple genes are shared across these pathways, indicating a common underlying thread.

Our findings demonstrated a significant difference in the expression of the analyzed pathways, as well as in the regulation of transcripts associated with these biological processes, between RLS patients and controls. Each pathway exhibited high fold enrichment, with circadian rhythms showing the greatest enrichment and involving numerous genes. Importantly, we highlighted the predominance of either up- or down-regulated genes within each pathway, providing insights into the genetic mechanisms underpinning circadian dysfunction and its related biological processes in RLS.

Specifically, we observed a marked predominance of down-regulated genes within the Chemical Carcinogenesis–ROS pathway in RLS, suggesting a potential suppression of the cellular oxidative stress response. This suppression could lead to the accumulation of oxidative damage, creating a favorable environment for the activation of ferroptosis, a process highly sensitive to increased ROS levels and redox imbalance [15]. Indeed, ferroptosis was markedly upregulated in RLS. This process appears further exacerbated by the downregulation of FoxO, PI3K-Akt, and phosphatidylinositol signaling pathways, all of which are crucial for oxidative stress response, cell survival, autophagy, and cellular repair mechanisms [28,29]. Additionally, downregulation of oxidative phosphorylation and adipocytokine signaling pathways may also critically contribute to increased oxidative stress and inflammation, thereby further promoting ferroptosis and neuronal damage [30,31].

Among these mechanisms, phosphatidylinositol signaling is also involved in iron metabolism regulation [30], suggesting it might directly influence ferroptosis and further contribute to a vicious cycle of dysregulation of iron metabolism.

It is well established that disruption of brain iron homeostasis is a fundamental pathological feature of RLS, with consequences for both dopaminergic and glutamatergic dysfunction [27]. However, the mechanisms driving dysregulation of iron metabolism remain unclear. Our group was the first to report a pronounced upregulation of ferroptosis in RLS [10], and this study provides a more in-depth exploration of that finding. Ferroptosis activation promotes intracellular dysregulation of iron metabolism [31], offering a key explanation for previous observations and the onset of inflammatory mechanisms in RLS [25], while also suggesting therapeutic potential for ferroptosis inhibitors in the disorder.

Of note, the upregulation of efferocytosis identified in our study, a crucial immune-modulatory process by which macrophages and other immune cells clear apoptotic or damaged cells [32], reinforces growing evidence implicating inflammatory, immune, and infectious mechanisms in RLS [10,25], consistent with autopsy studies in the disorder [33].

Another critical finding of our study is the downregulation of the PI3K-Akt pathway in RLS, a signaling cascade known to regulate neuroinflammation, neurogenesis, synaptic plasticity, and neurotransmission [29]. Impairment of this pathway could thus contribute to the dysfunction of all these processes, which are known to be altered in RLS [10,34], and may also be linked to the frequent comorbidity of depression in RLS patients [35].

Importantly, no difference in autophagy regulation was observed, with an equal number of up- and downregulated genes, suggesting that the protective effect of autophagy, key in eliminating excess iron and damaged cellular components, may be insufficient to counteract ferroptosis in RLS [36], thereby facilitating further dysregulation of iron metabolism, oxidative stress, and inflammation.

Another major goal of our study was to investigate the interplay between ferroptosis, circadian rhythms, and dopaminergic transmission; again, we found that multiple genes are shared among these pathways. Namely, we observed a strongly disrupted circadian rhythm pathway, with only two upregulated genes versus twelve downregulated ones, suggesting a marked suppression of the circadian system. Since circadian regulation governs many metabolic and antioxidant processes [37], its disruption may indirectly facilitate ferroptosis by impairing protective systems such as glutathione production and lipid peroxide detoxification.

Therefore, the dysfunction of the molecular clock may act as a key modulator of cellular vulnerability to ferroptosis [38,39] by amplifying oxidative stress imbalance. Investigation of RLS circadian rhythmicity in relation to circadian markers remains a crucial yet largely unexplored research area [40]. Despite the recognized importance of genetic factors in RLS and the diagnostic relevance of its circadian characteristics [8], studies to date have mainly focused on possible links between *CLOCK* genes and dopaminergic transmission [40].

It is important to note that the causal relationship between circadian dysregulation and ferroptosis activation remains to be clarified. While our findings suggest that circadian disruption may promote ferroptotic vulnerability, the inverse scenario is also plausible: ferroptosis-induced oxidative damage and neuroinflammation, particularly in dopaminergic pathways, may in turn impair circadian gene expression. The interaction may thus be bidirectional or self-reinforcing. Future longitudinal and experimental studies will be crucial to dissect these complex temporal dynamics and clarify the sequence of events in RLS pathophysiology.

Recent evidence suggests that circadian genes may also regulate ferroptosis [18], with *BMAL1* emerging as a key regulator of antioxidant systems suppressing ferroptosis [41]. In our study, *CRY1*, *PER1*, *PER3*, *CLOCK*, and *RORA* were all downregulated, also *BMAL1* (alias *ARNTL*) was downregulated, but with fold change −1.48 and *p*adj = 2.59 × 10^−6^, indicating that circadian suppression might represent a primary trigger for ferroptosis activation, intracellular dysregulation of iron metabolism, and inflammation in RLS.

Furthermore, recent studies in mouse models have shown that hypothalamic A11 dopaminergic neurons, known to be implicated in RLS pathogenesis [8], may be influenced by local circadian gene activity and could act as a relay between central circadian networks and motor regulation circuits [21]. This might explain the upregulation of dopaminergic synapses observed in our study as a compensatory mechanism for circadian rhythm disruption, even though increased synaptic transmission does not necessarily imply enhanced dopaminergic output, which is known to be impaired in RLS, likely due to ferroptosis-induced neuronal damage. Further research on this topic is necessary to confirm this hypothesis. Similarly, upregulation of circadian entrainment might reflect compensatory responses to circadian gene downregulation.

The circadian modulation of dopamine production has long been associated with RLS symptoms and motor manifestations such as PLMS [8]; however, this does not fully explain the link between circadian rhythms and other pathogenetic factors like iron deficiency, inflammation, or neurotransmitter network alterations [8].

In this context, it would be intriguing to investigate the potential correlation between daily and seasonal variations in RLS symptoms [8,42] and periodic, including seasonal, regulation of circadian genes. Innate circannual timing is an evolutionarily conserved trait present in many species, including humans, and contributes to seasonal variations in cellular function across different geographical regions [43]. At the molecular level, epigenetically regulated chromatin remodeling in pituitary cells of the hypothalamus is thought to drive seasonal oscillations in the transcriptional activity of specific circadian timer genes, mediating the shift between summer and winter phenotypes [43]. Further research into circannual rhythms may provide valuable insights into their contribution to human physiology and disease, including RLS, and may help explain both the circadian pattern of symptoms and the geographical variability in disease prevalence.

In this context, it is intriguing to consider recent hypotheses suggesting involvement of additional biological pathways in RLS, including orexinergic signaling [44] and the Calcitonin Gene-Related Peptide [45], both linked to hypothalamic structures and subject to circadian regulation [46].

This study has some limitations. First, the relatively small sample size (17 RLS patients and 39 controls) may limit the generalizability of the findings. However, this limitation was mitigated by stringent criteria for differential gene expression (adjusted *p*-value ≤ 0.01 and |fold-change| ≥ 1.5) and by the use of robust pathway enrichment analyses. An additional limitation is the unequal group size (17 RLS patients vs. 39 controls), which may introduce potential bias. However, all samples were processed using standardized protocols, and differential expression analysis was conducted using DESeq2, a method specifically designed to handle unbalanced groups through rigorous normalization and dispersion modeling. Furthermore, gene expression data were obtained from peripheral blood rather than neural tissue, which may not fully reflect central nervous system processes. Nonetheless, blood-based transcriptomic profiling has been increasingly recognized as a valid proxy for identifying systemic molecular alterations relevant to neurological disorders. Another limitation of our study is the lack of standardized assessment for subclinical anxiety and depression symptoms, which are common comorbidities in RLS. Although major psychiatric disorders were part of the exclusion criteria, the absence of formal rating scales may have limited our ability to account for milder affective symptoms that could potentially influence gene expression. Finally, while the study establishes associations between circadian gene expression, ferroptosis, and RLS, causal relationships cannot be inferred from transcriptomic data alone. Future studies with larger cohorts, longitudinal designs, and functional validation will be necessary to confirm and expand upon these findings.

## 5. Conclusions

**Figure 7:**
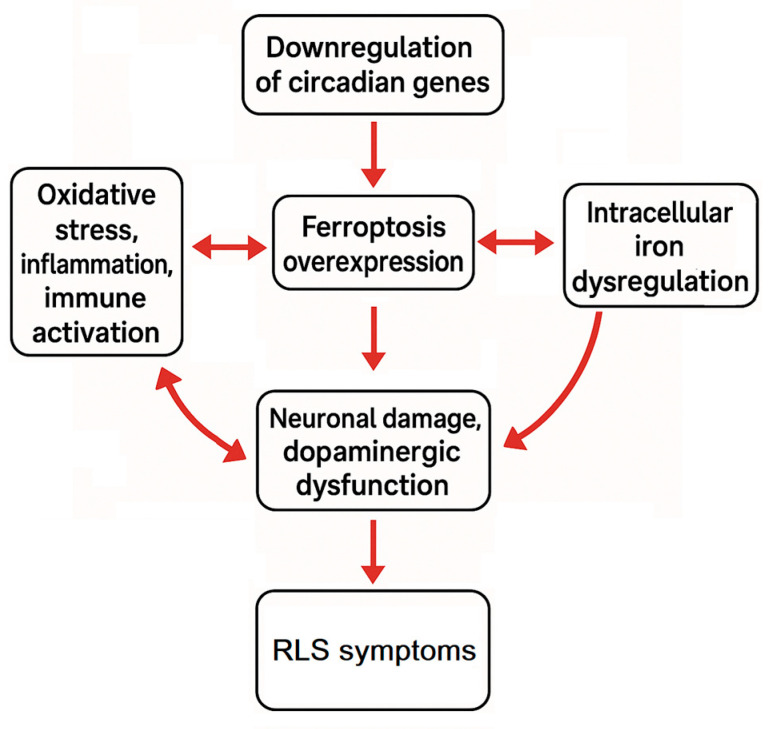
Schematic representation of the proposed pathogenic cycle in RLS. The diagram illustrates how the downregulation of circadian genes may initiate the overexpression of ferroptosis pathways. This, in turn, leads to intracellular iron accumulation, resulting in neuronal damage and dopaminergic dysfunction. These alterations promote increased oxidative stress, inflammation, and immune system activation, which further amplify ferroptosis processes. The cycle represents a self-perpetuating loop that may contribute to the neurobiological and clinical manifestations of RLS.

In conclusion, our study offers a comprehensive framework of RLS, suggesting that downregulation of circadian genes may drive ferroptosis overexpression, leading to intracellular dysregulation of iron metabolism, neuronal damage, dopaminergic dysfunction, and heightened oxidative stress, inflammation, and immune activation, thus establishing a vicious pathogenic cycle (Figure 7). Nevertheless, further studies with larger cohorts are needed to validate these findings and to clarify the complex role of circadian genes and ferroptosis in RLS pathogenesis.

Such research may also help determine whether ferroptosis could serve as a novel therapeutic target, as has been proposed in other conditions, including Parkinson’s disease and other neurodegenerative disorders [47].

## Figures and Tables

**Table 1:** List of 12 KEGG pathways enriched in RLS versus controls, with fold enrichment values, statistical significance (lowest and highest *p*-values), and the corresponding sets of upregulated and downregulated genes for each pathway.

ID	Term Description	Fold Change	Lowest *p*	Highest *p*	Up-Regulated	Down-Regulated
hsa04151	PI3K-Akt signaling pathway	1.39	3.30 × 10^−18^	2.89 × 10^−14^	*PIK3CB*, *PIK3R3*, *PIK3R1*, *EGF*, *ITGA5*, *LPAR2*, *FGF22*, *PDGFA*, *GNG11*, *SYNE1*, *COL1A1*, *FGF1*, *MAP2K2*, *MAP3K5*, *COL4A6*, *LAMA2*, *LAMB1*	*SPP1*, *MMP9*, *ITGA5*, *COL1A1*, *COL4A6*, *LAMB1*, *LAMA2*, *LAMA4*, *COL4A5*, *LAMB2*, *COL4A4*, *LAMC2*, *COL4A3*, *COL4A2*
hsa04068	FoxO signaling pathway	1.95	1.12 × 10^−15^	3.34 × 10^−8^	*MAP3K5*, *FOXO4*, *MAP2K2*, *BAD*, *PIK3CB*, *PIK3R3*, *PIK3R1*, *GABARAPL1*, *CALM3*, *GABARAP*, *CALM2*, *CALM1*	*MAP3K5*, *PIK3CB*, *PIK3R3*, *PIK3R1*, *SOX4*, *IRS1*, *BAD*, *GABARAPL1*, *GABARAP*, *CALM3*, *CALM2*, *CALM1*
hsa04140	Autophagy—animal	1.73	2.00 × 10^−13^	1.16 × 10^−13^	*IRS1*, *PIK3CB*, *PIK3R3*, *PIK3R1*, *MAP3K5*, *ATG2B*, *ATG4C*, *ATG7*, *RB1CC1*, *STX17*, *GABARAPL1*, *GABARAP*, *CALM3*, *CALM2*, *CALM1*	*IRS1*, *PIK3CB*, *PIK3R3*, *PIK3R1*, *MAP3K5*, *ATG2B*, *ATG4C*, *ATG7*, *RB1CC1*, *STX17*, *GABARAPL1*, *GABARAP*, *CALM3*, *CALM2*, *CALM1*
hsa04728	Dopaminergic synapse	1.36	3.84 × 10^−9^	1.09 × 10^−4^	*MAOA*, *VMAT2*, *GNAI1*, *GNG5*, *GNG3*, *GNG4*, *GNAQ*, *CAMK2B*, *CAMK2A*, *GRIN1*, *GRIA1*, *GRID1*, *GABRA2*, *CALM2*, *CALM1*	*SLC6A3*, *LEP*, *CALM1*, *CALM2*, *CAMK2A*, *CAMK2B*, *GNG5*, *GNG4*, *GNG3*, *GNAI1*
hsa04920	Adipocytokine signaling pathway	1.29	3.45 × 10^−8^	1.04 × 10^−2^	*CRPRA*, *ACSL1*, *LEP*, *POMC*, *ADIPOR1*, *STK11*, *RXRA*, *SLC2A1*, *TNFSF4*, *CAMKK2*	*NR1H3*, *PTPN11*, *SOCS3*, *IRS1*, *RXRA*, *PRKAA1*, *PRKAA2*, *LEP*, *RXRG*, *AKT3*, *MAPK8*, *STAT1*, *STAT3*
hsa00190	Oxidative phosphorylation	1.09	7.27 × 10^−6^	1.01 × 10^−5^	*NUDFB8*, *COX6C*, *TTC19*, *ATP6V2*, *ATP6V1B2*, *ATP6V1E1*, *COX7A2*, *ATP6V1G2*	*NUDFB6*, *NUDFB4*, *NDUFA1*, *NUDFB9*, *NUDFB5*, *NDUFA2*, *COX6C*, *ATP6V1E1*, *ATP6V1B2*
hsa04148	Efferocytosis	1.32	3.10 × 10^−5^	1.30 × 10^−3^	*AGER*, *GRK6*, *ROCK1*, *ERBB4*, *MAP3K5*, *BNIP3L*, *ITGA5*, *SYNE1*, *MAP2K2*, *GABARAPL1*, *PIK3CB*, *PIK3R1*, *PIK3R3*, *COL4A6*, *LAMA2*, *LAMB1*	*METRNL*, *OXTR*, *ITGA1*, *S1PR1*, *CD47*, *HVCN1*, *LRP1*, *VAV1*, *TUBB2B*, *CSF1R*, *GRK6*, *BNIP3L*, *MAP3K5*, *SYNE1*, *AGER*
hsa05208	Chemical carcinogenesis—reactive oxygen species	1.23	1.05 × 10^−6^	1.04 × 10^−5^	*PIK3CB*, *MAP3K5*, *MAP2K2*, *FGF1*, *CYBA*, *NCF1*, *NCF2*, *NDUFA2*, *COX6C*, *PRDX2*, *PRDX6*, *PRDX1*, *MAPKAPK2*, *MAPKAPK3*, *KEAP1*, *ASMT*, *SOD2*	*PIK3CB*, *MAP3K5*, *MAP2K2*, *FGF1*, *CYBA*, *NCFA*, *NCF2*, *PRDX2*, *PRDX1*, *PRDX6*, *MAPKAPK2*, *MAPKAPK3*, *KEAP1*, *ASMT*, *SOD2*
hsa04710	Circadian rhythm	2.89	3.91 × 10^−16^	4.62 × 10^−3^	*DBP*, *BHLHE41*	*RORA*, *PER1*, *PER2*, *PER3*, *CLOCK*
hsa04070	Phosphatidylinositol signaling system	1.35	6.61 × 10^−8^	3.73 × 10^−2^	*PTEN*, *CALM1*, *CALM2*, *PIK3R1*, *PIK3R3*, *DGKZ*, *GRIN1*, *CALM3*, *CALML4*, *CAMK2A*, *CAMK2B*, *CAMK2G*, *MAP2K2*, *MAP3K5*	*PIK3CB*, *MAP3K5*, *MAP2K2*, *GRIN1*, *CALM1*, *CALM2*, *CALM3*, *CAMK2A*, *CAMK2B*, *CAMK2G*
hsa04713	Circadian entrainment	1.29	4.36 × 10^−7^	4.85 × 10^−3^	*GRIN1*, *CALM1*, *CALM2*, *CAMK2A*, *CAMK2B*, *CAMK2G*, *MAP2K2*, *MAP3K5*	*GRIN1*, *CALM1*, *CALM2*, *CAMK2A*, *CAMK2B*, *CAMK2G*
hsa04216	Ferroptosis	1.57	2.45 × 10^−8^	4.60 × 10^−2^	*TP53*, *SLC1A4*, *PINK1*, *SLC7A11*, *ACSL4*	*TP53*, *SLC1A4*, *PINK1*, *SLC7A11*, *ACSL4*

## Data Availability

Raw sequencing data for RLS patients and controls were obtained from datasets E-MTAB-13155 and E-MTAB-11326, respectively. No new raw data were generated during the conduct of this new study.

## Supplementary Materials

The following supporting information can be downloaded at: https://www.mdpi.com/article/10.3390/biom15081184/s1. Table S1: List of differentially expressed genes (DEGs) identified between Restless Legs Syndrome (RLS) patients and control subjects. The table includes gene symbols, log2 fold change (log2FC), adjusted *p*-values (*p*adj), and expression direction (upregulated or downregulated) based on RNA-seq analysis. Genes were considered differentially expressed if *p*adj ≤ 0.01 and |log2FC| ≥ 1.5. This dataset forms the basis for subsequent pathway enrichment and gene network analyses presented in the main text.

## Abbreviations

The following abbreviations are used in this manuscript:

FC	Fold Change
FDR	False Discovery Rate
KEGG	Kyoto Encyclopedia of Genes and Genomes
PBMCs	Peripheral Blood Mononuclear Cells
PCA	Principal Component Analysis
PLMS	Periodic Limb Movements during Sleep
RIN	RNA Integrity Number
RLS	Restless Legs Syndrome
RNA	Ribonucleic Acid
ROS	Reactive Oxygen Species
SCN	Suprachiasmatic Nucleus
