# Supplementary material for: Exploring the Role of Ferroptosis in the Pathophysiology and Circadian Regulation of Restless Legs Syndrome
Source: Biomolecules. 2025 Aug 18;15(8):1184. doi: 10.3390/biom15081184 (PMC12384461; doi:10.3390/biom15081184)
Supplement: Supplementary file 1 [file biomolecules-15-01184-s001.zip › biomolecules-3754426-supplementary.pdf]

Exploring the Role of Ferroptosis in the Pathophysiology and Circadian Regulation of Restless Legs Syndrome

Maria P. Mogavero, [1,2]† Giovanna Marchese,[3,4]† Giovanna Ventola,[3,4] Giuseppe Lanza,[5,6] Oliviero Bruni,[7] Luigi Ferini-Strambi,[1,2] Raffaele Ferri[5]

[1]Vita-Salute San Raffaele University, 20132 Milan, Italy

[2]San Raffaele Scientific Institute, Division of Neuroscience, Sleep Disorders Center, 20127 Milan, Italy

[3]Genomix4Life Srl, 84081 Baronissi, Italy.

[4]Genome Research Center for Health-CRGS, 84081 Baronissi, Italy.

[5]Oasi Research Institute-IRCCS, 94018 Troina, Italy

[6]University of Catania, Department of Surgery and Medical-Surgical Specialties, 95123 Catania, Italy

[7]Sapienza University of Rome, Developmental and Social Psychology, 00185 Rome, Italy

†Equal contribution

Corresponding author: Dr. Raffaele Ferri, Sleep Research Centre, Oasi Research Institute-IRCCS, Via C. Ruggero 73, 94018 Troina (Italy) – rferri@oasi.en.it - +39-0935-936111

Supplementary Table S1. List of differentially expressed genes (DEGs) identified between Restless Legs Syndrome (RLS) patients and control subjects. The table includes gene symbols, log2 fold change (log2FC), adjusted p-values (padj), and expression direction (upregulated or downregulated) based on RNA-seq analysis. Genes were considered differentially expressed if padj ≤ 0.01 and $|log2FC| \geq 1.5$. This dataset forms the basis for subsequent pathway enrichment and gene network analyses presented in the main text.

GeneID	log2FoldChange	FoldChange	padj
AREG	-5.786	-55.173	0.000E+00
CCL3L1	-5.419	-42.781	0.000E+00
FOSB	-4.777	-27.420	0.000E+00
EREG	-4.727	-26.487	0.000E+00
NR4A2	-4.610	-24.422	0.000E+00
RGS1	-4.406	-21.199	0.000E+00
CXCL2	-4.312	-19.858	2.277E-10
FAM106A	-4.112	-17.295	0.000E+00
ZNF208	-4.092	-17.057	0.000E+00
AC093416.2	-4.041	-16.462	0.000E+00
ID1	-3.988	-15.869	0.000E+00
NR4A3	-3.961	-15.572	0.000E+00
EGR1	-3.956	-15.524	0.000E+00
AC073840.1	-3.784	-13.775	0.000E+00
G0S2	-3.764	-13.589	0.000E+00
AC044849.1	-3.753	-13.481	0.000E+00
CCDC144B	-3.638	-12.450	0.000E+00
MIR4772	-3.607	-12.182	7.899E-10
CXCL8	-3.546	-11.683	0.000E+00
AL512603.2	-3.545	-11.670	1.870E-10
HBEGF	-3.413	-10.654	0.000E+00
NDUFV2	-3.389	-10.475	0.000E+00
AP000560.1	-3.381	-10.421	2.323E-08
PTGER3	-3.367	-10.317	0.000E+00
FST	-3.326	-10.025	1.502E-03
UBE2FP1	-3.305	-9.883	0.000E+00
CD69	-3.304	-9.876	0.000E+00
AL596325.2	-3.261	-9.585	1.469E-08
FOSL1	-3.245	-9.480	4.213E-08
CCDC144A	-3.229	-9.378	4.236E-04
TNFAIP3	-3.197	-9.169	0.000E+00
FAT4	-3.183	-9.080	0.000E+00
NT5C3AP2	-3.156	-8.914	0.000E+00
JUN	-3.145	-8.849	0.000E+00
AC104619.3	-3.145	-8.845	1.514E-07
AC107032.1	-3.138	-8.801	3.277E-07
DUSP2	-3.128	-8.741	0.000E+00
EEF1A1P14	-3.121	-8.701	0.000E+00
AC090181.2	-3.113	-8.649	0.000E+00
CCIN	-3.103	-8.591	6.081E-07
CD83	-3.090	-8.514	0.000E+00
ZBTB40-IT1	-3.086	-8.493	0.000E+00
AL031667.3	-3.070	-8.397	7.006E-08
AC234775.2	-3.040	-8.227	0.000E+00
AC010547.4	-3.020	-8.110	4.031E-09
AC026124.2	-3.006	-8.032	0.000E+00
AP000892.4	-3.005	-8.030	9.072E-07
RPL34P27	-3.001	-8.006	1.283E-06
SNORD11	-2.979	-7.886	7.240E-09
EEF1A1P38	-2.972	-7.848	3.857E-07
DUSP4	-2.939	-7.667	0.000E+00
NCR3LG1	-2.924	-7.590	0.000E+00
AL136126.1	-2.919	-7.565	4.285E-07
AC006042.2	-2.916	-7.546	0.000E+00
SIK1B	-2.912	-7.524	0.000E+00
EIF4A1	-2.910	-7.514	0.000E+00
SNORD59A	-2.903	-7.480	0.000E+00
DDX47	-2.902	-7.477	0.000E+00
AC118658.1	-2.884	-7.381	6.478E-10
HUS1B	-2.877	-7.348	1.134E-06
LINC02728	-2.865	-7.285	0.000E+00
AC005076.2	-2.862	-7.272	3.050E-07
AC024940.6	-2.846	-7.190	3.076E-04
EBLN2	-2.813	-7.026	0.000E+00
MIR6503	-2.741	-6.685	8.777E-09
PDE4D	-2.740	-6.679	0.000E+00
AC005244.1	-2.709	-6.538	1.313E-06
CNTF	-2.708	-6.534	0.000E+00
CHRM4	-2.703	-6.512	7.180E-08
AC095055.1	-2.695	-6.475	0.000E+00
SIK1	-2.692	-6.464	7.072E-05
MIR4742	-2.681	-6.411	1.206E-04
SYDE2	-2.669	-6.360	0.000E+00
AC012065.3	-2.654	-6.293	0.000E+00
MT-TH	-2.650	-6.275	2.522E-07
AC108727.1	-2.642	-6.243	3.070E-06
FOS	-2.640	-6.234	0.000E+00
ZNF705E	-2.639	-6.229	1.034E-07
AC108693.1	-2.629	-6.187	3.108E-06
Z68871.1	-2.620	-6.149	6.880E-08
HTD2	-2.609	-6.099	7.790E-08
CXCL3	-2.575	-5.957	1.389E-04
AL589993.1	-2.574	-5.955	0.000E+00
AL133352.1	-2.570	-5.940	2.661E-08
RPS3AP20	-2.564	-5.912	0.000E+00
AC022001.2	-2.562	-5.904	3.522E-05
RNU6-878P	-2.557	-5.884	2.582E-06
NAP1L4P1	-2.552	-5.865	2.690E-10
RPS26P21	-2.547	-5.843	1.578E-05
AC069499.1	-2.543	-5.828	1.151E-07
AC005840.3	-2.538	-5.809	5.572E-08
ANKRD26P4	-2.527	-5.763	1.092E-05
AL360270.1	-2.525	-5.755	3.429E-10
AC104623.2	-2.517	-5.726	1.303E-05
AC013474.1	-2.497	-5.644	7.286E-07
PER1	-2.496	-5.641	0.000E+00
RPL39P15	-2.494	-5.635	4.384E-04
LINC01395	-2.494	-5.634	4.106E-06
RPS7P3	-2.481	-5.582	0.000E+00
ELOCP19	-2.477	-5.566	0.000E+00
AC007278.2	-2.466	-5.527	5.298E-10
BTF3P3	-2.464	-5.516	3.003E-05
FARP1-AS1	-2.463	-5.514	6.139E-06
FFAR1	-2.456	-5.486	5.393E-07
SNORD100	-2.452	-5.473	0.000E+00
AC004453.1	-2.450	-5.463	5.364E-06
U2AF1L5	-2.435	-5.409	2.028E-05
AC091516.1	-2.433	-5.401	8.438E-05
AURKAP1	-2.431	-5.392	4.505E-05

AC015911.4	-2.431	-5.392	9.374E-09
DEPDC1P2	-2.426	-5.375	1.846E-04
AC108449.2	-2.424	-5.368	0.000E+00
RNU6-1016P	-2.424	-5.367	0.000E+00
DLEU2L	-2.423	-5.364	0.000E+00
RNU6-32P	-2.421	-5.356	9.391E-05
SNORD19	-2.412	-5.323	1.308E-07
NCBP2-AS1	-2.391	-5.244	4.355E-05
FSIP2	-2.389	-5.238	1.548E-04
AC027544.1	-2.386	-5.228	5.116E-09
AC068831.6	-2.383	-5.215	0.000E+00
RPL41P5	-2.381	-5.208	1.591E-06
SRGAP2D	-2.378	-5.198	9.494E-06
TAF9	-2.376	-5.191	6.801E-10
CEP170P1	-2.376	-5.190	3.189E-05
THBS1	-2.374	-5.185	2.522E-09
AC025594.2	-2.373	-5.181	0.000E+00
SNRPGP15	-2.373	-5.180	3.468E-05
AL117336.1	-2.364	-5.147	1.126E-05
MCAM	-2.364	-5.147	2.852E-07
RPS15AP30	-2.354	-5.111	1.594E-04
VASH2	-2.353	-5.110	2.226E-05
AL590560.4	-2.351	-5.100	2.317E-09
AC253536.3	-2.350	-5.099	0.000E+00
AC069234.5	-2.350	-5.098	1.383E-08
MIR4420	-2.344	-5.078	1.373E-09
MIR616	-2.337	-5.052	3.463E-09
AC063950.1	-2.334	-5.042	2.832E-09
AC109326.1	-2.327	-5.016	2.757E-06
RNY1P16	-2.319	-4.988	1.596E-06
RPS15AP17	-2.315	-4.977	1.023E-03
TAMALIN	-2.313	-4.969	0.000E+00
MIR7161	-2.309	-4.955	9.481E-08
ANKRD36C	-2.308	-4.952	0.000E+00
ACTG1P3	-2.308	-4.952	0.000E+00
KLHL11	-2.302	-4.930	0.000E+00
RABGGTB	-2.300	-4.924	0.000E+00
AL022067.1	-2.294	-4.905	9.081E-06
NPM1P37	-2.293	-4.900	1.378E-05
FAM182A	-2.289	-4.886	1.988E-05
F3	-2.286	-4.876	1.192E-03
LINC01088	-2.283	-4.867	1.790E-05
EIF4BP7	-2.276	-4.842	0.000E+00
AL157902.2	-2.273	-4.834	4.033E-04
LRRC70	-2.267	-4.814	5.571E-10
TCEA1P4	-2.265	-4.808	7.148E-05
SEC14L1P1	-2.263	-4.801	0.000E+00
SEC14L2	-2.263	-4.800	0.000E+00
C2orf27A	-2.261	-4.794	3.247E-08
GOLGA8H	-2.261	-4.793	4.247E-05
RASGEF1B	-2.261	-4.793	0.000E+00
AC079684.1	-2.261	-4.792	2.215E-09
AC007435.1	-2.260	-4.789	5.973E-05
NBEAL1	-2.259	-4.785	0.000E+00
RPL37P6	-2.252	-4.763	6.275E-04
RNU6ATAC24P	-2.249	-4.752	3.695E-10
WDR45P1	-2.246	-4.744	7.831E-04
RNA5SP82	-2.239	-4.721	8.315E-10
AC234775.3	-2.238	-4.716	5.369E-06
B3GNT7	-2.234	-4.705	0.000E+00
Z83840.1	-2.226	-4.678	0.000E+00
USP32P3	-2.224	-4.672	0.000E+00
RN7SKP173	-2.224	-4.671	4.589E-09
AC079193.2	-2.222	-4.667	1.473E-05
RPL23AP81	-2.221	-4.662	2.435E-05
AC020910.5	-2.219	-4.655	0.000E+00
MRPS31P5	-2.211	-4.631	0.000E+00
CCDC144CP	-2.207	-4.617	1.036E-06
USP32P2	-2.205	-4.612	8.084E-05
SNORA31	-2.202	-4.602	6.042E-07
AL356966.1	-2.201	-4.599	2.645E-07
AC138393.2	-2.193	-4.572	1.403E-06
KLF7-IT1	-2.191	-4.567	7.426E-10
LINC02700	-2.191	-4.567	5.859E-05
MIR6766	-2.182	-4.536	5.257E-04
EMP1	-2.175	-4.516	0.000E+00
MIR3136	-2.174	-4.513	8.080E-06
AL050343.2	-2.174	-4.512	0.000E+00
RN7SL834P	-2.171	-4.504	0.000E+00
AP001020.2	-2.171	-4.502	1.675E-04
REL	-2.169	-4.496	0.000E+00
SYNM	-2.162	-4.474	0.000E+00
AP000786.1	-2.160	-4.469	3.158E-09
WDR49	-2.158	-4.463	0.000E+00
RGS17P1	-2.156	-4.457	0.000E+00
AC097372.2	-2.154	-4.451	2.215E-05
NR4A1	-2.151	-4.440	0.000E+00
HERC2P10	-2.148	-4.433	1.135E-03
TANC1	-2.143	-4.415	1.874E-08
DIP2A-IT1	-2.141	-4.410	4.364E-09
AC022795.1	-2.139	-4.403	6.753E-03
ANKRD36B	-2.135	-4.394	0.000E+00
SGCD	-2.134	-4.389	2.183E-06
AL133355.1	-2.133	-4.386	1.283E-06
AL008718.3	-2.129	-4.375	5.930E-05
RPL21P119	-2.129	-4.375	2.477E-05
KLF10	-2.128	-4.371	0.000E+00
KRT17P8	-2.119	-4.344	2.974E-10
AC048382.2	-2.114	-4.330	2.905E-04
AL451139.1	-2.111	-4.320	1.560E-03
MT-TL2	-2.110	-4.317	2.818E-04
VN1R83P	-2.097	-4.279	1.074E-08
AC108448.2	-2.097	-4.278	1.337E-06
CCDC168	-2.094	-4.268	2.568E-04
AL049697.1	-2.093	-4.268	0.000E+00
AC091060.1	-2.093	-4.265	1.192E-10
BNC2	-2.092	-4.264	0.000E+00
PPP2R5CP	-2.092	-4.262	0.000E+00
AC009961.2	-2.090	-4.258	1.019E-05
AC027544.2	-2.089	-4.254	9.815E-05

SNORD46	-2.088	-4.251	1.593E-08
PCDHGB5	-2.086	-4.246	1.163E-05
ITGB8	-2.083	-4.238	2.916E-08
NFKBIZ	-2.077	-4.220	0.000E+00
MIR573	-2.073	-4.206	3.505E-10
SREK1	-2.063	-4.179	0.000E+00
SNORA16B	-2.062	-4.175	5.795E-06
AC018690.1	-2.061	-4.173	0.000E+00
DDX50P1	-2.059	-4.166	2.956E-10
AC104109.4	-2.054	-4.153	1.867E-06
LINC02256	-2.040	-4.112	2.318E-05
PIK3R1	-2.040	-4.112	0.000E+00
AC020663.3	-2.038	-4.106	0.000E+00
AC012181.1	-2.036	-4.100	0.000E+00
AL365475.1	-2.034	-4.096	9.241E-10
SNORA2B	-2.032	-4.090	1.958E-04
LINC02390	-2.031	-4.086	0.000E+00
SBDSP1	-2.030	-4.084	0.000E+00
AC008115.3	-2.029	-4.081	0.000E+00
VDAC1P8	-2.028	-4.078	0.000E+00
N4BP2	-2.022	-4.061	0.000E+00
AP003717.1	-2.021	-4.058	0.000E+00
PTX3	-2.017	-4.047	2.930E-07
AC015911.7	-2.016	-4.044	0.000E+00
KRT5	-2.010	-4.027	1.268E-08
ERRFI1	-2.010	-4.027	4.839E-10
AC130448.2	-2.008	-4.022	2.812E-09
SLC26A5	-2.006	-4.015	8.961E-03
AC091814.1	-2.002	-4.005	4.517E-05
DST	-1.999	-3.998	0.000E+00
AL118516.1	-1.999	-3.998	0.000E+00
DDN-AS1	-1.999	-3.998	5.039E-03
LINC02605	-1.999	-3.997	1.161E-04
DNAJC19P5	-1.998	-3.993	1.796E-10
LINC01876	-1.996	-3.989	4.105E-05
ENC1	-1.995	-3.987	0.000E+00
ATF3	-1.994	-3.983	2.482E-09
MTCO1P40	-1.991	-3.975	7.976E-08
AC090181.3	-1.990	-3.973	5.370E-09
C15orf48	-1.989	-3.970	6.903E-05
AL355073.1	-1.987	-3.964	3.771E-06
EGR3	-1.984	-3.955	4.632E-06
AL132656.1	-1.983	-3.953	2.607E-08
AC020951.1	-1.981	-3.948	9.911E-10
KLF11	-1.981	-3.947	0.000E+00
GUSBP4	-1.981	-3.947	8.129E-04
IL1RL1	-1.980	-3.946	2.356E-08
BCL2L2-PABPN1	-1.972	-3.922	1.141E-04
AL163932.1	-1.967	-3.909	4.630E-07
AL353763.1	-1.966	-3.907	1.228E-08
RALGAPA1P1	-1.965	-3.903	0.000E+00
AC025539.1	-1.960	-3.892	4.571E-03
AP000254.1	-1.958	-3.884	1.915E-08
AC113410.3	-1.957	-3.883	3.195E-04
GCSAML	-1.957	-3.882	0.000E+00
SNORA3B	-1.953	-3.873	0.000E+00
ARHGAP8	-1.949	-3.862	6.729E-05
RPL37P2	-1.949	-3.862	3.744E-06
ALMS1-IT1	-1.948	-3.860	0.000E+00
AC018647.3	-1.948	-3.859	9.433E-08
AC022558.1	-1.945	-3.849	2.321E-07
SNAI1	-1.940	-3.838	4.567E-06
SUCLG2-AS1	-1.940	-3.838	0.000E+00
AC092535.3	-1.940	-3.836	1.962E-03
SNORD101	-1.939	-3.834	2.602E-07
ZBTB10	-1.936	-3.826	0.000E+00
AHNAK	-1.935	-3.823	0.000E+00
TLK2P2	-1.928	-3.804	3.874E-03
ACAD11	-1.925	-3.799	0.000E+00
LINC02598	-1.924	-3.796	8.168E-05
SACS	-1.923	-3.792	0.000E+00
MATN1	-1.923	-3.791	3.886E-06
IL31RA	-1.922	-3.790	8.945E-09
AC008119.1	-1.921	-3.787	3.535E-04
AC068989.1	-1.916	-3.775	7.184E-06
AC012186.2	-1.916	-3.774	1.440E-07
NOCT	-1.910	-3.758	0.000E+00
AC012181.2	-1.907	-3.750	0.000E+00
AC099336.2	-1.906	-3.748	3.533E-07
PGAP1	-1.904	-3.742	0.000E+00
AC000123.3	-1.901	-3.735	0.000E+00
NAP1L5	-1.901	-3.734	0.000E+00
AC104695.3	-1.898	-3.727	1.206E-06
Z93943.1	-1.896	-3.722	7.856E-04
NFKBIA	-1.896	-3.722	0.000E+00
RPL36A	-1.894	-3.718	0.000E+00
AC019197.1	-1.893	-3.714	1.450E-04
RPL32P2	-1.887	-3.698	5.321E-06
OSM	-1.887	-3.698	0.000E+00
RNY1P12	-1.886	-3.697	1.634E-03
IGSF3	-1.886	-3.696	4.395E-04
FMR1-IT1	-1.885	-3.693	0.000E+00
WDR17	-1.885	-3.693	0.000E+00
PRLR	-1.883	-3.687	0.000E+00
RPL34P18	-1.878	-3.675	3.176E-07
MIR590	-1.877	-3.674	1.841E-10
RPA4	-1.876	-3.671	1.123E-03
AL139407.1	-1.875	-3.667	0.000E+00
AL136018.1	-1.874	-3.667	2.757E-03
AC074050.4	-1.874	-3.665	5.275E-03
ANKRD36	-1.871	-3.657	0.000E+00
SNORA2C	-1.870	-3.654	2.549E-03
AC090948.2	-1.869	-3.654	0.000E+00
LINC00641	-1.869	-3.654	0.000E+00
AL133243.3	-1.867	-3.648	0.000E+00
AL354822.1	-1.867	-3.647	8.102E-04
LINC01619	-1.867	-3.647	0.000E+00
MIR186	-1.863	-3.638	3.063E-06
SCML1	-1.858	-3.625	0.000E+00
AL442067.2	-1.857	-3.623	3.220E-03

SCML2P2	-1.855	-3.618	9.059E-05
TNRC18P1	-1.853	-3.613	5.937E-06
NIPA1	-1.852	-3.610	1.924E-09
PLCB4	-1.851	-3.609	6.979E-04
GPRIN3	-1.848	-3.599	0.000E+00
NONOP2	-1.846	-3.596	4.646E-08
COL5A2	-1.846	-3.594	1.964E-05
LINC02470	-1.845	-3.593	4.215E-03
AL161787.1	-1.845	-3.592	2.964E-09
DDR2	-1.844	-3.590	2.330E-09
CHML	-1.843	-3.588	0.000E+00
AL359715.1	-1.843	-3.588	0.000E+00
HSP90B2P	-1.841	-3.582	3.170E-05
KRT128P	-1.839	-3.578	2.336E-06
AC245060.5	-1.839	-3.578	1.963E-08
OR7E7P	-1.838	-3.575	0.000E+00
AL358790.1	-1.833	-3.563	5.368E-03
HMGN5	-1.832	-3.561	0.000E+00
AC006978.1	-1.831	-3.559	0.000E+00
TJP2	-1.829	-3.552	0.000E+00
HAVCR1	-1.827	-3.548	0.000E+00
RPL21P116	-1.825	-3.543	3.023E-03
XIST	-1.823	-3.538	0.000E+00
SNORA20	-1.822	-3.536	3.697E-05
AC109992.2	-1.821	-3.533	2.051E-05
GOLGA8M	-1.820	-3.532	3.035E-04
AC068620.1	-1.820	-3.530	1.805E-04
MCF2	-1.819	-3.528	0.000E+00
RNU6-226P	-1.818	-3.525	1.117E-03
BDP1	-1.815	-3.518	3.278E-03
KLRC4	-1.813	-3.513	2.827E-07
MIR6774	-1.812	-3.511	2.282E-07
SEM1	-1.812	-3.511	0.000E+00
MT-TT	-1.810	-3.506	3.466E-08
AC093525.7	-1.809	-3.504	6.080E-03
MIR5692C2	-1.805	-3.495	6.217E-03
EGR2	-1.804	-3.491	1.931E-05
AP001893.1	-1.803	-3.489	4.558E-07
SMAD6	-1.803	-3.489	8.964E-05
AL161443.1	-1.801	-3.484	5.510E-04
LRRFIP1P1	-1.800	-3.482	3.248E-07
RAB39A	-1.799	-3.479	2.333E-10
AL606834.1	-1.797	-3.474	4.022E-04
AP000943.3	-1.797	-3.474	5.899E-03
NPIPB8	-1.795	-3.470	4.658E-03
LINC02861	-1.794	-3.468	7.045E-04
ZNF618	-1.794	-3.467	0.000E+00
AL138955.1	-1.792	-3.463	3.861E-05
AC125437.1	-1.791	-3.461	1.087E-05
SBDS	-1.791	-3.459	0.000E+00
INHBA	-1.789	-3.456	2.600E-03
MT-TD	-1.788	-3.453	2.307E-05
AC069234.4	-1.785	-3.447	3.922E-03
AC019131.2	-1.783	-3.441	3.812E-07
CEP290	-1.783	-3.440	0.000E+00
MT-TR	-1.783	-3.440	3.024E-03
AC027290.2	-1.782	-3.438	0.000E+00
CCNL1	-1.781	-3.437	0.000E+00
AC115223.1	-1.780	-3.435	4.990E-08
ZNF215	-1.779	-3.432	8.928E-04
AC009495.1	-1.779	-3.432	3.217E-04
FNDC7	-1.777	-3.428	1.132E-03
FREM1	-1.777	-3.426	9.131E-03
AL356273.2	-1.776	-3.424	1.929E-04
CREM	-1.774	-3.420	0.000E+00
TPT1P6	-1.772	-3.416	6.382E-05
AC132872.4	-1.771	-3.412	6.524E-06
PIGA	-1.766	-3.402	0.000E+00
AC007610.4	-1.766	-3.400	8.836E-09
CHASERR	-1.762	-3.391	0.000E+00
ZMAT1	-1.759	-3.385	0.000E+00
FRG1JP	-1.758	-3.383	1.299E-04
PTS	-1.752	-3.367	0.000E+00
MIR181A1HG	-1.750	-3.365	1.518E-03
IL6	-1.750	-3.363	4.015E-03
AC022150.4	-1.750	-3.363	0.000E+00
LRP1	-1.746	-3.354	0.000E+00
AC090948.1	-1.745	-3.352	0.000E+00
ZNF334	-1.745	-3.351	2.935E-05
MTND4P14	-1.743	-3.346	1.368E-04
CTTNBP2	-1.742	-3.346	1.703E-04
C1GALT1P1	-1.742	-3.344	8.855E-04
P2RY4	-1.741	-3.343	1.056E-03
SCUBE3	-1.739	-3.338	4.008E-03
NKTR	-1.737	-3.334	0.000E+00
AC131391.1	-1.730	-3.317	3.796E-03
BCLAF1P2	-1.729	-3.316	1.109E-05
SNORA2A	-1.729	-3.314	2.148E-04
KLF9	-1.728	-3.312	0.000E+00
CCT8P1	-1.724	-3.303	0.000E+00
PCDHGA10	-1.724	-3.303	4.685E-04
LONRF3	-1.723	-3.301	0.000E+00
GPR75	-1.721	-3.297	0.000E+00
IGSF11	-1.719	-3.292	2.448E-03
TEX12	-1.719	-3.292	3.816E-04
RNU6ATAC18P	-1.718	-3.291	1.308E-04
KRT8P33	-1.717	-3.288	0.000E+00
DCT	-1.716	-3.286	1.113E-05
AC000120.1	-1.716	-3.286	1.015E-09
ZNF331	-1.716	-3.284	0.000E+00
AP000866.6	-1.715	-3.282	0.000E+00
AC090181.1	-1.713	-3.279	7.886E-07
SFMBT2	-1.712	-3.277	0.000E+00
RPL34	-1.711	-3.273	0.000E+00
ARHGAP21	-1.710	-3.273	0.000E+00
AC092376.2	-1.710	-3.272	1.722E-03
BTG1P1	-1.708	-3.267	0.000E+00
PSMD6-AS2	-1.706	-3.261	0.000E+00
AC114495.2	-1.705	-3.261	1.065E-09
SH3PXD2B	-1.705	-3.260	0.000E+00

AC244034.1	-1.701	-3.252	2.112E-03
RBM44	-1.701	-3.251	0.000E+00
AL356273.3	-1.698	-3.245	0.000E+00
ROBO1	-1.698	-3.244	1.252E-04
IL2	-1.697	-3.243	9.030E-03
PCDHGB3	-1.694	-3.236	1.700E-03
EML6	-1.693	-3.233	0.000E+00
STARD4	-1.692	-3.230	0.000E+00
AL161891.1	-1.691	-3.228	0.000E+00
MT-TV	-1.690	-3.226	1.603E-06
RPS15AP38	-1.687	-3.221	4.582E-04
RPL36AP15	-1.687	-3.221	3.261E-03
FSBP	-1.684	-3.213	0.000E+00
AL353743.4	-1.684	-3.213	1.648E-04
ACTG1P20	-1.684	-3.213	8.387E-07
AC009704.2	-1.683	-3.212	7.605E-06
SLC25A36	-1.682	-3.209	0.000E+00
MIR6731	-1.681	-3.207	7.408E-03
IGSF10	-1.681	-3.206	1.900E-03
AL162258.1	-1.680	-3.204	7.792E-03
AC036214.2	-1.677	-3.197	3.308E-10
BTG3	-1.677	-3.197	0.000E+00
EEF1A1P11	-1.674	-3.190	1.044E-07
FRG1CP	-1.673	-3.189	0.000E+00
VCAN	-1.673	-3.189	0.000E+00
RN7SL81P	-1.672	-3.187	7.276E-04
SNORD62B	-1.672	-3.186	0.000E+00
TCTE1	-1.670	-3.183	3.646E-04
LINC01058	-1.670	-3.183	3.462E-03
GOLGA6L4	-1.669	-3.181	7.517E-03
ERVK3-1	-1.666	-3.174	0.000E+00
KLHL15	-1.665	-3.172	0.000E+00
TRPS1	-1.665	-3.171	0.000E+00
VPS13A	-1.664	-3.169	0.000E+00
TMSB4XP4	-1.664	-3.169	5.801E-06
AC023590.1	-1.663	-3.168	5.069E-05
RPL24P2	-1.662	-3.165	5.066E-04
RPS7P10	-1.662	-3.164	2.049E-05
GUSBP2	-1.661	-3.163	4.944E-09
AC010186.4	-1.658	-3.156	5.954E-05
RORA	-1.658	-3.155	0.000E+00
BTAF1	-1.657	-3.154	0.000E+00
SHLD3	-1.657	-3.154	0.000E+00
WHAMMP3	-1.657	-3.153	0.000E+00
ABCA13	-1.655	-3.149	3.476E-04
COL24A1	-1.654	-3.148	2.137E-09
SPAG4	-1.653	-3.145	1.567E-05
PPIAP41	-1.652	-3.142	1.146E-05
MIR5581	-1.652	-3.142	1.524E-03
SNORA50A	-1.651	-3.140	1.139E-03
SORBS2	-1.651	-3.140	1.966E-03
AL353746.1	-1.650	-3.139	1.986E-03
HMGN1P1	-1.647	-3.132	2.594E-06
FAM133DP	-1.645	-3.128	8.989E-04
AVPI1	-1.644	-3.124	1.360E-07
TMEM170B	-1.639	-3.115	0.000E+00
SNORA28	-1.635	-3.107	3.148E-03
PMAIP1	-1.632	-3.099	0.000E+00
TSIX	-1.630	-3.096	4.370E-03
ZNF326	-1.630	-3.095	0.000E+00
RN7SKP70	-1.629	-3.094	1.450E-05
GPR135	-1.627	-3.088	0.000E+00
SNORD83A	-1.627	-3.088	1.384E-07
UHRF1BP1	-1.627	-3.088	0.000E+00
ALG11	-1.624	-3.081	0.000E+00
AL020997.2	-1.624	-3.081	3.695E-03
NME2P1	-1.623	-3.081	6.058E-03
MT-TC	-1.623	-3.079	1.412E-04
HMGB1P8	-1.614	-3.060	9.913E-03
KCNMA1	-1.614	-3.060	4.154E-04
HSPE1P3	-1.612	-3.057	7.555E-04
LACTB2-AS1	-1.612	-3.057	1.356E-08
TNKS	-1.609	-3.051	0.000E+00
AC132872.3	-1.608	-3.048	3.262E-04
KRT2	-1.606	-3.044	1.511E-04
FAM221B	-1.606	-3.044	1.604E-03
FBN2	-1.606	-3.043	0.000E+00
RBM41	-1.605	-3.043	0.000E+00
ZFAND2A	-1.605	-3.042	0.000E+00
AC021321.2	-1.603	-3.038	1.525E-04
DNM1P46	-1.603	-3.037	1.069E-05
AC004938.2	-1.603	-3.037	7.382E-06
AKAP5	-1.601	-3.034	9.002E-10
PTGS2	-1.601	-3.033	4.736E-03
NDUFAF5	-1.601	-3.033	0.000E+00
SUGT1P4-STRA6LP-CCDC180	1.731E-03	-1.599	-3.029
BET1P1	-1.598	-3.028	9.146E-04
HMGN1P8	-1.598	-3.028	4.868E-04
FNBP1P1	-1.598	-3.028	2.083E-07
AC004053.1	-1.597	-3.025	4.881E-03
ANKRD20A5P	-1.597	-3.025	7.389E-10
AL591848.3	-1.596	-3.022	4.041E-05
SNORA72	-1.595	-3.021	7.809E-10
FMN1	-1.593	-3.016	2.951E-06
CCSER1	-1.593	-3.016	3.438E-08
RSPH4A	-1.592	-3.014	6.530E-08
CTLA4	-1.591	-3.012	0.000E+00
ARHGAP32	-1.590	-3.010	0.000E+00
IL15	-1.589	-3.009	0.000E+00
ACVR1C	-1.589	-3.008	0.000E+00
COL19A1	-1.589	-3.008	5.694E-07
PPARGC1B	-1.589	-3.008	0.000E+00
CRNDE	-1.588	-3.007	1.302E-04
EAF2	-1.585	-3.000	0.000E+00
ZNF731P	-1.585	-2.999	0.000E+00
RN7SL145P	-1.585	-2.999	1.312E-05
ADAM9	-1.584	-2.997	0.000E+00
ZBTB20	-1.582	-2.994	2.324E-09
ARL5B	-1.581	-2.992	4.298E-09

TTC3P1	-1.581	-2.992	0.000E+00
TENM1	-1.580	-2.990	0.000E+00
GLYATL1P2	-1.579	-2.988	1.135E-03
MT-TK	-1.579	-2.988	2.721E-03
AC090615.1	-1.577	-2.984	7.357E-03
PLK2	-1.577	-2.984	3.974E-09
ELF3	-1.575	-2.978	0.000E+00
AL354798.1	-1.574	-2.978	3.733E-04
SNORA66	-1.574	-2.978	4.571E-08
SCARNA4	-1.574	-2.978	1.950E-03
TIMM23B-AGAP6	-1.574	-2.977	1.770E-10
AC106865.1	-1.573	-2.975	1.668E-03
ADGRA3	-1.571	-2.972	0.000E+00
AUTS2	-1.571	-2.970	0.000E+00
RPL26	-1.571	-2.970	0.000E+00
RNF217	-1.570	-2.968	0.000E+00
RN7SL851P	-1.569	-2.968	9.436E-03
NEXMIF	-1.568	-2.965	3.761E-09
SPDYA	-1.568	-2.965	3.578E-05
AC018845.3	-1.568	-2.964	1.933E-03
ZBTB43	-1.567	-2.964	0.000E+00
PPP1R9A	-1.567	-2.963	2.374E-07
PFDN4	-1.566	-2.961	0.000E+00
TTC28	-1.566	-2.961	0.000E+00
ARAP2	-1.563	-2.954	0.000E+00
WAKMAR2	-1.562	-2.952	0.000E+00
SENP3-EIF4A1	-1.559	-2.948	1.010E-04
HLA-E	-1.558	-2.944	8.720E-04
AC137630.3	-1.558	-2.944	1.238E-06
PLCL1	-1.556	-2.941	0.000E+00
PLXNA4	-1.555	-2.938	0.000E+00
ZFHX3	-1.555	-2.938	0.000E+00
LINGO2	-1.553	-2.935	8.019E-05
GOLGA8K	-1.552	-2.932	6.104E-03
HMCN1	-1.552	-2.932	3.898E-03
AC146507.2	-1.551	-2.931	7.323E-04
AC064801.2	-1.551	-2.929	8.800E-06
ZNF727	-1.550	-2.928	1.617E-04
ZNF704	-1.549	-2.927	1.316E-05
TNR	-1.548	-2.924	2.450E-05
JMY	-1.548	-2.924	0.000E+00
RPS29P16	-1.548	-2.924	1.911E-04
OR7E140P	-1.545	-2.919	1.485E-06
WWC2	-1.545	-2.917	0.000E+00
MIR1285-1	-1.541	-2.911	1.520E-03
NUAK1	-1.540	-2.908	2.376E-05
SMG1P1	-1.540	-2.908	1.537E-06
Z97633.1	-1.540	-2.907	1.116E-03
ZBED6	-1.535	-2.898	3.890E-05
CAVIN4	-1.532	-2.892	6.179E-03
GAS2L3	-1.532	-2.891	1.534E-06
SRGAP1	-1.530	-2.887	1.720E-05
TRGJ1	-1.528	-2.884	7.737E-03
AC090517.2	-1.527	-2.882	1.833E-05
MYSM1	-1.525	-2.879	0.000E+00
NBEA	-1.525	-2.878	0.000E+00
NRIP1	-1.525	-2.877	0.000E+00
CACHD1	-1.523	-2.874	4.218E-05
RYR3	-1.522	-2.872	2.616E-04
HEG1	-1.522	-2.871	0.000E+00
TFEC	-1.521	-2.869	0.000E+00
CLEC1B	-1.521	-2.869	2.727E-05
GOLGA8R	-1.520	-2.869	7.943E-04
KCNA3	-1.519	-2.866	0.000E+00
IFI44	-1.518	-2.864	1.724E-08
MERTK	-1.517	-2.862	0.000E+00
AC105129.1	-1.516	-2.860	1.639E-03
RAB11FIP2	-1.516	-2.860	0.000E+00
RPS24P19	-1.516	-2.860	1.064E-04
IFI44L	-1.515	-2.858	3.591E-05
RPL22L1	-1.514	-2.857	0.000E+00
LRGUK	-1.513	-2.855	6.534E-04
KLF4	-1.513	-2.855	0.000E+00
AC079142.1	-1.512	-2.852	1.915E-04
PRC1	-1.512	-2.851	8.246E-05
AHI1	-1.510	-2.848	0.000E+00
RBM34	-1.510	-2.848	0.000E+00
AGPAT4	-1.510	-2.847	0.000E+00
ZBTB21	-1.508	-2.844	0.000E+00
KLRC4-KLRK1	-1.508	-2.843	8.604E-09
IGHEP2	-1.507	-2.842	4.818E-05
RALGAPA1	-1.506	-2.841	0.000E+00
FBXW4P1	-1.506	-2.840	7.283E-09
DFFBP1	-1.504	-2.836	4.443E-09
ZNF462	-1.503	-2.833	4.958E-05
RGS13	-1.501	-2.831	2.202E-03
AL450992.1	-1.500	-2.829	8.588E-03
YES1	-1.499	-2.826	1.067E-08
PKD2L1	-1.498	-2.825	9.556E-03
XYLT1	-1.498	-2.824	9.439E-08
PGBD5	-1.497	-2.823	1.809E-03
EBAG9P1	-1.497	-2.823	5.181E-04
PCDHGB6	-1.496	-2.820	6.301E-05
MT1XP1	-1.493	-2.815	4.374E-03
RPS2P36	-1.493	-2.815	1.698E-05
AC004922.1	-1.493	-2.814	9.498E-04
AKAP9	-1.491	-2.810	0.000E+00
CEP85L	-1.491	-2.810	0.000E+00
AC009303.1	-1.491	-2.810	3.108E-03
AC117503.2	-1.490	-2.808	7.264E-04
AL158055.1	-1.489	-2.807	0.000E+00
AC087276.1	-1.487	-2.802	3.983E-06
AL132656.4	-1.486	-2.800	2.081E-08
SASH1	-1.485	-2.800	0.000E+00
SPATA6L	-1.484	-2.797	2.571E-09
PPM1L	-1.484	-2.797	0.000E+00
SNHG5	-1.484	-2.796	1.504E-08
FO393411.1	-1.481	-2.792	1.274E-04
AASS	-1.481	-2.790	0.000E+00
MTND5P14	-1.480	-2.790	1.032E-03

HIPK2	-1.480	-2.790	0.000E+00
RPS24	-1.480	-2.789	0.000E+00
AC108673.2	-1.478	-2.785	8.633E-08
SRSF10	-1.474	-2.779	0.000E+00
IL12A	-1.474	-2.778	2.682E-09
RPL34P22	-1.473	-2.776	4.437E-03
DMXL1	-1.472	-2.774	0.000E+00
ZNF292	-1.471	-2.772	0.000E+00
C3orf35	-1.470	-2.770	1.164E-09
SELENOI	-1.470	-2.770	0.000E+00
SETBP1	-1.469	-2.769	0.000E+00
SNORA71B	-1.469	-2.768	1.667E-06
SNHG14	-1.468	-2.767	0.000E+00
AL109923.1	-1.467	-2.765	1.732E-03
SNORA33	-1.467	-2.765	8.393E-08
RPS15AP1	-1.467	-2.764	1.154E-05
NQO2-AS1	-1.466	-2.763	7.942E-03
SYTL2	-1.465	-2.761	0.000E+00
AL136984.1	-1.465	-2.761	9.912E-04
GFPT2	-1.465	-2.761	4.026E-05
AC092683.1	-1.465	-2.761	9.289E-05
ALG10	-1.464	-2.758	0.000E+00
VPS13C	-1.464	-2.758	0.000E+00
RIC1	-1.463	-2.757	0.000E+00
ATXN1L	-1.462	-2.754	0.000E+00
TC2N	-1.461	-2.752	0.000E+00
PLAG1	-1.460	-2.751	0.000E+00
SNORA53	-1.459	-2.750	3.578E-05
AC007878.1	-1.458	-2.748	2.385E-07
CCDC59	-1.458	-2.747	0.000E+00
RASAL2	-1.458	-2.747	8.342E-09
ZBTB16	-1.457	-2.746	0.000E+00
PLCXD2	-1.456	-2.744	0.000E+00
P2RY1	-1.456	-2.743	9.317E-10
RPL7AP50	-1.455	-2.742	5.722E-06
AC068700.1	-1.454	-2.739	2.540E-06
AC010203.1	-1.452	-2.737	1.949E-06
CLCN3P1	-1.452	-2.736	6.984E-03
TMEM232	-1.451	-2.733	3.332E-03
AL513548.4	-1.450	-2.733	5.166E-03
MN1	-1.450	-2.733	3.349E-06
TRAPPC10	-1.449	-2.730	0.000E+00
ANKRD36BP2	-1.448	-2.728	1.368E-07
RPS27AP11	-1.447	-2.727	3.453E-03
KIAA1549	-1.447	-2.726	9.473E-03
ZGRF1	-1.446	-2.724	0.000E+00
BRCA1P1	-1.445	-2.723	2.764E-03
LINC00653	-1.444	-2.721	0.000E+00
RBBP4P1	-1.443	-2.720	5.356E-04
ODF2L	-1.443	-2.720	0.000E+00
UBE2Q2P1	-1.442	-2.717	2.372E-09
EML5	-1.442	-2.716	0.000E+00
CHD9	-1.442	-2.716	0.000E+00
FAM229B	-1.440	-2.712	8.375E-06
AC040169.3	-1.439	-2.711	2.770E-04
LRRC32	-1.438	-2.709	7.651E-04
ANKRD50	-1.437	-2.707	0.000E+00
AL589765.5	-1.436	-2.706	8.507E-04
MAPK6	-1.435	-2.704	0.000E+00
ANKRD26	-1.435	-2.703	0.000E+00
CCDC88A	-1.435	-2.703	0.000E+00
AC007278.1	-1.434	-2.703	3.010E-06
IKZF2	-1.433	-2.701	0.000E+00
KIAA1958	-1.433	-2.700	0.000E+00
CCDC14	-1.430	-2.695	0.000E+00
AC026979.2	-1.430	-2.695	1.733E-08
FLT3	-1.430	-2.694	0.000E+00
AL033519.3	-1.429	-2.692	4.139E-05
ACTA2-AS1	-1.428	-2.690	1.590E-07
AC103974.1	-1.427	-2.689	7.008E-04
EPHA2	-1.427	-2.688	8.438E-07
AC114752.2	-1.427	-2.688	1.308E-03
SLC4A7	-1.424	-2.683	0.000E+00
CXCR4	-1.423	-2.681	0.000E+00
AC093010.1	-1.423	-2.681	0.000E+00
RPL17	-1.423	-2.681	0.000E+00
PHOSPHO2	-1.422	-2.680	3.805E-07
CNTLN	-1.422	-2.680	0.000E+00
AL021368.2	-1.422	-2.679	0.000E+00
HERC2	-1.421	-2.677	0.000E+00
C2orf49-DT	-1.420	-2.677	0.000E+00
FGD6	-1.420	-2.677	0.000E+00
SNORD14A	-1.419	-2.674	1.656E-07
NACA2	-1.418	-2.673	3.506E-04
MTR	-1.418	-2.672	0.000E+00
BICC1	-1.417	-2.671	1.356E-03
ERCC5	-1.416	-2.669	0.000E+00
NHSL1	-1.415	-2.667	0.000E+00
SNORD38A	-1.414	-2.666	2.906E-04
AIDAP2	-1.414	-2.665	3.161E-05
PRKXP1	-1.413	-2.664	2.730E-05
ABCA9	-1.413	-2.663	7.522E-05
PKD1P4	-1.413	-2.663	3.928E-05
NGDN	-1.411	-2.659	0.000E+00
FAM169A	-1.411	-2.659	0.000E+00
TRMT11	-1.410	-2.658	0.000E+00
RAI14	-1.410	-2.657	1.510E-03
KLF12	-1.410	-2.656	0.000E+00
DSG2	-1.409	-2.656	4.793E-03
AP003557.1	-1.408	-2.653	2.891E-05
KIAA1109	-1.407	-2.652	0.000E+00
RN7SL809P	-1.406	-2.650	3.292E-05
AL354809.1	-1.405	-2.649	5.591E-06
AL096816.1	-1.405	-2.648	0.000E+00
MACF1	-1.404	-2.647	0.000E+00
GALNT4	-1.404	-2.646	2.693E-04
RPS29P5	-1.403	-2.645	3.395E-03
AL080276.2	-1.401	-2.641	0.000E+00
RPS29	-1.401	-2.641	2.553E-08
AC022001.3	-1.400	-2.639	3.315E-03

RNU6-100P	-1.399	-2.637	4.328E-03
RN7SKP296	-1.398	-2.635	5.538E-04
RBM8B	-1.397	-2.634	3.796E-03
AC006008.1	-1.397	-2.634	3.263E-05
KCNQ3	-1.397	-2.633	6.640E-04
MYBL1	-1.396	-2.632	0.000E+00
TGFBR3	-1.395	-2.630	0.000E+00
LAMB2	-1.395	-2.630	0.000E+00
AC073912.2	-1.394	-2.629	6.270E-04
ZDHHC21	-1.394	-2.628	0.000E+00
PDE4DIPP2	-1.392	-2.625	0.000E+00
LUZP1	-1.392	-2.625	0.000E+00
OLFM5P	-1.392	-2.624	7.106E-04
NUDT11	-1.391	-2.623	8.321E-06
RNVU1-30	-1.391	-2.622	4.071E-05
SYCP2	-1.390	-2.621	0.000E+00
HERC2P9	-1.390	-2.621	1.622E-04
CHDH	-1.390	-2.620	9.073E-05
TMEM178A	-1.388	-2.617	6.964E-03
GTF2IP23	-1.388	-2.617	1.797E-05
AP003170.1	-1.387	-2.616	1.315E-04
MT-TE	-1.387	-2.615	6.589E-05
LRP8	-1.386	-2.613	0.000E+00
OR2L2	-1.386	-2.613	9.534E-03
YPEL2	-1.386	-2.613	0.000E+00
ACAP2-IT1	-1.385	-2.611	1.010E-04
PNN	-1.382	-2.607	0.000E+00
GK5	-1.380	-2.604	0.000E+00
AC079305.1	-1.380	-2.603	4.779E-03
ZNF354C	-1.380	-2.602	0.000E+00
MRPS31P4	-1.379	-2.602	1.904E-09
AC084824.3	-1.379	-2.601	4.196E-07
AC096921.2	-1.378	-2.599	0.000E+00
AL138963.3	-1.377	-2.598	5.827E-07
CCDC18	-1.377	-2.598	0.000E+00
ZC3H11A	-1.377	-2.597	4.748E-07
ZNF507	-1.376	-2.595	0.000E+00
RPL31	-1.375	-2.594	6.206E-10
SNORA12	-1.375	-2.593	7.767E-04
HMGB3	-1.374	-2.593	0.000E+00
BTBD8	-1.374	-2.592	0.000E+00
PLCB1	-1.374	-2.592	0.000E+00
ZNF471	-1.374	-2.591	1.927E-08
MAFB	-1.372	-2.588	8.869E-09
KTN1	-1.372	-2.588	0.000E+00
ARFGEF2	-1.370	-2.585	0.000E+00
FUT4	-1.369	-2.584	0.000E+00
BOLA2-SMG1P6	-1.368	-2.581	1.424E-03
TMEM135	-1.368	-2.581	0.000E+00
AC006504.3	-1.367	-2.580	9.804E-03
AC093849.4	-1.367	-2.579	1.840E-04
ASTL	-1.366	-2.578	1.847E-03
AL161457.2	-1.366	-2.578	5.557E-04
FAM66D	-1.366	-2.577	8.695E-04
AC092653.1	-1.366	-2.577	1.284E-09
CEP112	-1.365	-2.576	1.548E-04
SNX18P3	-1.364	-2.573	1.689E-03
RPS13P2	-1.362	-2.571	5.213E-08
PPEF2	-1.361	-2.569	1.322E-04
AL606834.2	-1.361	-2.569	0.000E+00
MDN1	-1.361	-2.569	0.000E+00
MICU3	-1.360	-2.568	5.748E-06
RPS27AP5	-1.357	-2.562	4.584E-05
LINC00852	-1.356	-2.560	0.000E+00
BMS1P1	-1.356	-2.560	9.183E-06
ERMN	-1.355	-2.558	6.314E-10
PHIP	-1.354	-2.557	0.000E+00
OTUD4	-1.354	-2.555	0.000E+00
GOLGA8O	-1.353	-2.555	3.026E-03
CHD1	-1.353	-2.554	0.000E+00
UBE2V1	-1.353	-2.554	0.000E+00
DNAJC2	-1.353	-2.554	0.000E+00
TTC39B	-1.351	-2.551	0.000E+00
PEAK1	-1.351	-2.551	0.000E+00
AL137058.2	-1.350	-2.549	8.282E-05
TNFSF9	-1.350	-2.549	1.502E-04
TRIM51BP	-1.349	-2.548	2.087E-03
RNF32-AS1	-1.349	-2.548	0.000E+00
SNORA11	-1.349	-2.548	1.703E-03
MIR7152	-1.348	-2.546	7.315E-05
NACA3P	-1.348	-2.545	5.495E-04
CUBN	-1.347	-2.544	0.000E+00
DYTN	-1.346	-2.543	2.365E-03
OR56B2P	-1.346	-2.543	5.127E-03
PSD3	-1.346	-2.543	0.000E+00
AC108010.1	-1.346	-2.542	9.561E-08
MT-TA	-1.345	-2.541	8.750E-05
AL138889.3	-1.345	-2.540	9.650E-03
SNRPA1	-1.344	-2.538	0.000E+00
AKR1D1P1	-1.344	-2.538	8.333E-04
TXLNGY	-1.342	-2.535	3.377E-06
C16orf87	-1.340	-2.531	0.000E+00
TTBK2	-1.338	-2.528	0.000E+00
DMTF1	-1.337	-2.527	0.000E+00
RPS27AP12	-1.337	-2.527	6.857E-03
LINC01881	-1.336	-2.524	0.000E+00
PANK3	-1.335	-2.524	0.000E+00
AL663070.1	-1.335	-2.522	9.342E-04
CDYL2	-1.333	-2.520	0.000E+00
AC122718.2	-1.332	-2.517	8.294E-04
AC100830.1	-1.332	-2.517	1.397E-03
PSTK	-1.331	-2.516	0.000E+00
COX10-AS1	-1.330	-2.515	0.000E+00
HDC	-1.330	-2.515	9.683E-07
ADAMTS6	-1.330	-2.514	6.756E-09
CSRNP1	-1.330	-2.514	9.397E-10
SSBP3-AS1	-1.330	-2.514	4.475E-05
DDHD1	-1.328	-2.510	0.000E+00
AL133330.1	-1.328	-2.510	3.858E-07
TTC32	-1.326	-2.506	0.000E+00

RHOT1P1	-1.325	-2.506	8.433E-04
UGGT2	-1.325	-2.505	0.000E+00
FAM160A1	-1.324	-2.503	7.194E-08
AC139795.1	-1.323	-2.502	7.066E-08
NCAM1	-1.323	-2.502	4.058E-10
AL669831.4	-1.323	-2.501	1.924E-09
MAP4K3	-1.322	-2.501	0.000E+00
TFRC	-1.322	-2.500	0.000E+00
CATSPERE	-1.322	-2.499	2.623E-09
SPICE1	-1.321	-2.498	1.147E-10
CHD6	-1.321	-2.498	0.000E+00
TMPPE	-1.320	-2.497	1.070E-09
PAK3	-1.319	-2.496	6.272E-04
SLC28A3	-1.317	-2.492	3.038E-06
AC098679.4	-1.317	-2.492	6.767E-03
SLC2A13	-1.317	-2.491	0.000E+00
DYNC2H1	-1.316	-2.489	0.000E+00
AP001372.4	-1.316	-2.489	4.547E-03
AC018450.1	-1.315	-2.489	2.355E-06
AC053513.2	-1.315	-2.487	1.438E-04
MT-TP	-1.315	-2.487	7.734E-05
TIMM23B	-1.314	-2.487	0.000E+00
CNBD2	-1.314	-2.486	2.133E-09
AL360270.2	-1.314	-2.486	6.411E-04
AL391988.1	-1.314	-2.486	1.557E-07
SMPD4P1	-1.313	-2.485	2.679E-03
AC135983.2	-1.312	-2.483	0.000E+00
TNFRSF21	-1.312	-2.483	2.630E-07
ATAD5	-1.312	-2.483	0.000E+00
AC107071.1	-1.311	-2.482	2.162E-06
AC124283.3	-1.311	-2.481	5.240E-04
PAXBP1	-1.311	-2.481	0.000E+00
SIGLEC22P	-1.311	-2.481	8.949E-10
ZBED4	-1.309	-2.478	0.000E+00
DUSP1	-1.309	-2.477	3.588E-09
CSGALNACT2	-1.309	-2.477	0.000E+00
PARGP1	-1.309	-2.477	0.000E+00
AC021078.1	-1.307	-2.475	0.000E+00
SYNE2	-1.307	-2.475	0.000E+00
RANBP6	-1.306	-2.473	0.000E+00
PTAR1	-1.306	-2.473	0.000E+00
OXNAD1	-1.305	-2.472	0.000E+00
RTTN	-1.305	-2.471	0.000E+00
SEPTIN7-DT	-1.305	-2.470	0.000E+00
AC092835.1	-1.304	-2.470	7.714E-06
AC098934.1	-1.304	-2.469	3.775E-03
RASSF6	-1.304	-2.469	6.216E-04
TPH1	-1.303	-2.467	2.967E-10
CAMSAP2	-1.303	-2.467	0.000E+00
RN7SL608P	-1.301	-2.464	1.278E-09
TRPC1	-1.301	-2.464	0.000E+00
UQCRB	-1.301	-2.464	1.363E-09
CPXM1	-1.300	-2.463	4.956E-04
AC092718.6	-1.300	-2.463	1.353E-05
SNHG15	-1.298	-2.458	0.000E+00
AC005831.1	-1.298	-2.458	0.000E+00
NOTCH2NLC	-1.297	-2.456	8.134E-07
RFX3-AS1	-1.296	-2.455	9.065E-08
KCTD3	-1.296	-2.455	0.000E+00
LDB2	-1.296	-2.455	9.660E-05
DUSP5	-1.294	-2.452	0.000E+00
AC118553.2	-1.293	-2.450	4.167E-04
SHPRH	-1.292	-2.449	0.000E+00
RPL13AP25	-1.291	-2.448	3.474E-04
MTX3	-1.290	-2.446	0.000E+00
SAXO2	-1.290	-2.446	7.005E-03
MRTFB	-1.289	-2.444	0.000E+00
GPRASP1	-1.289	-2.444	0.000E+00
BACH2	-1.289	-2.444	2.212E-07
SOCS6	-1.289	-2.443	0.000E+00
ZNF711	-1.289	-2.443	4.019E-10
ATP5PO	-1.288	-2.442	0.000E+00
RPL21P10	-1.288	-2.442	4.299E-04
EEF1A1P4	-1.287	-2.440	6.722E-04
AC005839.1	-1.287	-2.440	2.369E-07
ANAPC1P1	-1.287	-2.440	2.702E-05
SMARCE1	-1.287	-2.440	0.000E+00
NOTCH3	-1.287	-2.439	3.999E-04
SERPINI2	-1.286	-2.439	2.638E-04
MLLT10	-1.284	-2.434	0.000E+00
LINC01505	-1.283	-2.434	4.238E-04
AL359885.1	-1.283	-2.434	2.595E-03
LINC01980	-1.283	-2.434	2.176E-03
CCNJ	-1.283	-2.433	0.000E+00
AC104806.2	-1.283	-2.433	1.016E-04
AC010615.1	-1.283	-2.433	4.167E-06
BRWD1	-1.282	-2.432	0.000E+00
NPTN-IT1	-1.281	-2.431	8.515E-07
MYCBP2	-1.281	-2.431	0.000E+00
CD163	-1.280	-2.429	0.000E+00
FRMD6	-1.280	-2.429	1.354E-04
DPY19L2P2	-1.280	-2.428	0.000E+00
DGKH	-1.279	-2.427	0.000E+00
TRIO	-1.278	-2.425	0.000E+00
DOCK1	-1.278	-2.425	1.253E-06
GZF1	-1.278	-2.424	0.000E+00
KRR1	-1.277	-2.423	0.000E+00
CIP2A	-1.274	-2.418	0.000E+00
NAA16	-1.274	-2.418	0.000E+00
ZNF736	-1.274	-2.418	0.000E+00
IFI30	-1.273	-2.416	9.041E-10
AL139317.5	-1.271	-2.413	6.019E-03
IL4	-1.271	-2.413	6.542E-03
COL27A1	-1.270	-2.411	1.011E-05
RNU6-322P	-1.269	-2.411	1.790E-04
ZNF675	-1.269	-2.411	0.000E+00
DCTN6	-1.269	-2.410	0.000E+00
PHC3	-1.269	-2.410	0.000E+00
TBC1D32	-1.267	-2.407	0.000E+00
AC062029.1	-1.267	-2.406	6.113E-03

NECAP1	-1.266	-2.405	0.000E+00
PAQR3	-1.266	-2.405	0.000E+00
AP005212.4	-1.265	-2.404	2.755E-05
MIPEPP3	-1.265	-2.402	6.202E-03
EEF1A1P10	-1.264	-2.402	8.079E-04
ZNF256	-1.263	-2.400	0.000E+00
AC073569.3	-1.262	-2.399	9.237E-04
ZNF678	-1.262	-2.398	0.000E+00
PLEKHA5	-1.262	-2.398	1.091E-10
AC006504.1	-1.262	-2.398	3.647E-10
METTL14-DT	-1.262	-2.397	6.334E-03
RNPC3	-1.261	-2.397	0.000E+00
MIER3	-1.261	-2.397	0.000E+00
ITGA6	-1.261	-2.396	0.000E+00
ATP2A2	-1.260	-2.396	0.000E+00
NFAT5	-1.259	-2.393	9.331E-07
ZNF720	-1.258	-2.392	0.000E+00
ZNF721	-1.258	-2.392	0.000E+00
FAM102B	-1.258	-2.392	0.000E+00
NYNRIN	-1.258	-2.391	7.917E-05
TXK	-1.257	-2.391	0.000E+00
AC024084.1	-1.257	-2.390	1.129E-05
EXPH5	-1.257	-2.390	3.611E-10
FOSL2	-1.257	-2.390	1.172E-10
SMG1P3	-1.257	-2.390	3.018E-05
FRG1EP	-1.257	-2.390	7.219E-04
GUSBP1	-1.256	-2.389	2.799E-06
UACA	-1.256	-2.388	4.818E-10
SRSF11	-1.256	-2.388	0.000E+00
MAP9	-1.255	-2.387	1.714E-10
TENT4B	-1.255	-2.386	0.000E+00
PHF2P2	-1.254	-2.386	1.994E-04
SNX30	-1.254	-2.385	0.000E+00
KCNK10	-1.254	-2.385	5.141E-05
TRAJ6	-1.253	-2.384	2.026E-05
ZNF639	-1.253	-2.383	0.000E+00
MTCO3P12	-1.253	-2.383	1.176E-05
DMXL2	-1.252	-2.382	5.481E-10
ZBTB1	-1.252	-2.382	0.000E+00
RLF	-1.252	-2.381	0.000E+00
CCDC171	-1.252	-2.381	7.009E-08
AC016590.2	-1.252	-2.381	2.781E-04
NOMO2	-1.252	-2.381	0.000E+00
OTULIN	-1.251	-2.380	0.000E+00
MYEF2	-1.251	-2.379	0.000E+00
RBAK	-1.250	-2.379	0.000E+00
PPP2R3A	-1.250	-2.378	1.253E-04
NRIP3	-1.250	-2.378	2.035E-03
AKT3	-1.250	-2.378	9.217E-04
STARD4-AS1	-1.249	-2.378	9.447E-05
ANKRD49P1	-1.249	-2.377	4.095E-03
MYLK4	-1.249	-2.376	1.742E-08
ZNF295-AS1	-1.248	-2.374	3.872E-03
BTBD3	-1.247	-2.374	0.000E+00
ATP8A1	-1.247	-2.373	0.000E+00

CEP152	-1.247	-2.373	0.000E+00
SEMA5A	-1.246	-2.372	1.181E-05
AC005911.1	-1.245	-2.370	9.320E-04
AC020765.2	-1.245	-2.370	2.514E-04
AC139494.2	-1.245	-2.370	2.745E-03
EPB41L4A-AS1	-1.243	-2.367	0.000E+00
ZNF827	-1.243	-2.367	0.000E+00
EDAR	-1.242	-2.366	4.366E-08
SLC26A7	-1.242	-2.365	5.450E-03
TPP2	-1.241	-2.364	0.000E+00
ARHGAP12	-1.241	-2.363	0.000E+00
ARHGAP31	-1.240	-2.362	0.000E+00
GLIS3	-1.240	-2.361	2.522E-04
HNRNPA1P27	-1.239	-2.360	6.433E-03
CCNT1	-1.237	-2.357	0.000E+00
NR1D2	-1.237	-2.357	0.000E+00
ITGA9	-1.234	-2.353	8.412E-05
LRRC36	-1.234	-2.352	1.120E-04
CASC15	-1.234	-2.352	7.528E-04
PKD1L1	-1.234	-2.351	1.641E-05
ZNF521	-1.231	-2.347	1.722E-04
AFF4	-1.230	-2.346	0.000E+00
CCSER2	-1.230	-2.346	0.000E+00
AC116158.3	-1.230	-2.346	2.100E-05
RPL9	-1.230	-2.345	5.575E-08
FAM76B	-1.229	-2.344	0.000E+00
TRAJ7	-1.229	-2.344	5.035E-04
PPP1R17	-1.229	-2.344	2.766E-03
AC068631.3	-1.229	-2.344	3.950E-03
CLMN	-1.228	-2.343	0.000E+00
CD109	-1.228	-2.342	1.296E-09
ZNF66	-1.227	-2.341	3.840E-07
IKZF5	-1.226	-2.339	1.107E-10
RPS7	-1.226	-2.339	2.598E-10
PDZD2	-1.226	-2.339	1.809E-06
NOP58	-1.225	-2.338	0.000E+00
TNFSF11	-1.225	-2.338	2.634E-03
KCNA2	-1.225	-2.337	0.000E+00
HTR7	-1.223	-2.335	3.072E-06
ADNP2	-1.223	-2.334	0.000E+00
SPAG17	-1.222	-2.333	9.611E-03
RFX7	-1.220	-2.329	0.000E+00
AC078881.1	-1.219	-2.328	5.721E-07
TMTC2	-1.219	-2.327	0.000E+00
SPTBN1	-1.218	-2.326	0.000E+00
ALMS1	-1.218	-2.326	0.000E+00
AL353611.1	-1.218	-2.326	1.694E-03
TRA2B	-1.217	-2.324	0.000E+00
SLC16A7	-1.215	-2.322	0.000E+00
RNF139	-1.215	-2.321	0.000E+00
B4GALT6	-1.212	-2.317	3.504E-07
RPL26P6	-1.212	-2.316	3.983E-06
RBBP6	-1.210	-2.314	0.000E+00
SLC4A4	-1.210	-2.313	2.939E-07
GREM2	-1.209	-2.312	4.706E-04

AC010186.2	-1.209	-2.312	0.000E+00
DCBLD2	-1.207	-2.308	1.913E-07
HELZ	-1.206	-2.307	0.000E+00
FAM133B	-1.205	-2.306	0.000E+00
RBM38-AS1	-1.205	-2.306	7.127E-08
SLC5A3	-1.205	-2.306	2.521E-10
CMTM4	-1.205	-2.305	0.000E+00
RPL23	-1.205	-2.305	3.319E-08
USP46	-1.204	-2.304	0.000E+00
USP43	-1.204	-2.304	4.905E-05
AC134407.3	-1.203	-2.302	2.109E-06
RBM15	-1.202	-2.301	0.000E+00
CCNA1	-1.202	-2.300	1.148E-03
LMLN	-1.199	-2.296	0.000E+00
MYO7A	-1.198	-2.295	0.000E+00
ZFHX2-AS1	-1.198	-2.294	0.000E+00
LINC00630	-1.196	-2.291	9.145E-08
CCAR1	-1.196	-2.291	0.000E+00
EEF1B2P6	-1.196	-2.291	5.012E-04
ARMCX4	-1.196	-2.291	0.000E+00
TMEM161B	-1.195	-2.289	0.000E+00
NUS1P1	-1.195	-2.289	3.255E-03
HECTD1	-1.195	-2.289	0.000E+00
AF117829.1	-1.194	-2.287	0.000E+00
CDC14A	-1.193	-2.287	0.000E+00
MOB1B	-1.193	-2.287	0.000E+00
PSMD10P1	-1.193	-2.287	8.493E-08
ZNF460	-1.193	-2.286	7.699E-07
AC025171.2	-1.192	-2.285	0.000E+00
DDX21	-1.192	-2.285	0.000E+00
ANKRD18A	-1.192	-2.285	1.118E-04
TTC3	-1.192	-2.284	0.000E+00
ANKDD1B	-1.191	-2.284	3.479E-05
NAA35	-1.191	-2.284	0.000E+00
AC090204.1	-1.191	-2.283	7.430E-08
TBC1D12	-1.191	-2.282	0.000E+00
ZNF724	-1.190	-2.282	1.509E-05
FMNL2	-1.189	-2.280	1.302E-09
AL031320.2	-1.189	-2.280	3.744E-04
NEB	-1.189	-2.280	2.798E-07
LRCH1	-1.189	-2.279	0.000E+00
RPS27A	-1.188	-2.279	1.300E-07
AL359715.2	-1.188	-2.278	4.014E-05
PRKY	-1.188	-2.278	1.888E-09
SEMA3C	-1.187	-2.277	2.599E-09
C4orf50	-1.186	-2.276	7.691E-06
AC146507.3	-1.185	-2.274	5.671E-05
XRCC2	-1.184	-2.273	1.149E-05
AC002558.3	-1.184	-2.273	1.091E-03
AC103724.4	-1.184	-2.272	3.195E-03
EEF1B2P3	-1.184	-2.272	5.041E-05
ARPC4-TTLL3	-1.184	-2.271	4.224E-05
LRP2BP	-1.183	-2.270	1.335E-05
AC022211.2	-1.182	-2.270	0.000E+00
AC002558.1	-1.182	-2.269	2.619E-03
FSIP1	-1.182	-2.269	1.637E-04
SLFN12L	-1.182	-2.269	0.000E+00
PAG1	-1.181	-2.268	0.000E+00
MMP21	-1.181	-2.267	2.629E-04
EEA1	-1.181	-2.267	7.116E-09
BMS1P4	-1.181	-2.267	0.000E+00
AL133375.1	-1.181	-2.267	2.145E-03
AC103810.1	-1.180	-2.266	6.247E-04
NR3C2	-1.180	-2.265	0.000E+00
SNHG8	-1.179	-2.264	5.756E-08
LDLRAD3	-1.179	-2.264	0.000E+00
LINC00402	-1.178	-2.263	0.000E+00
OCRL	-1.176	-2.260	0.000E+00
CCDC50	-1.176	-2.259	0.000E+00
TRIM44	-1.175	-2.258	0.000E+00
SLC35A3	-1.175	-2.258	0.000E+00
UTRN	-1.174	-2.257	0.000E+00
LINC02757	-1.174	-2.256	4.811E-03
RNF19A	-1.174	-2.256	0.000E+00
AC092645.1	-1.173	-2.255	1.442E-05
PANX1	-1.173	-2.255	0.000E+00
GDAP1	-1.173	-2.254	0.000E+00
GRPR	-1.172	-2.254	1.863E-03
AC004494.2	-1.172	-2.253	1.572E-04
AC007262.2	-1.171	-2.252	7.803E-07
ACTG1P14	-1.171	-2.252	3.841E-04
PRKAR2B	-1.170	-2.251	9.829E-07
RPL11P1	-1.169	-2.249	2.487E-03
AC008870.2	-1.169	-2.248	5.321E-10
AC139491.7	-1.169	-2.248	2.375E-04
CEP78	-1.168	-2.247	0.000E+00
NLRP3	-1.167	-2.246	4.566E-09
ZXDB	-1.166	-2.243	0.000E+00
ZNF738	-1.165	-2.243	0.000E+00
AL096701.1	-1.165	-2.242	6.594E-03
AC009495.2	-1.165	-2.242	8.119E-03
KRT86	-1.164	-2.241	9.658E-03
LRRCC1	-1.164	-2.240	0.000E+00
OGT	-1.164	-2.240	0.000E+00
TNRC6A	-1.164	-2.240	0.000E+00
ABL2	-1.163	-2.240	0.000E+00
CDKN1A	-1.162	-2.237	5.838E-08
MPHOSPH9	-1.161	-2.237	0.000E+00
HMGB1P6	-1.161	-2.236	4.547E-08
COLGALT2	-1.161	-2.236	5.322E-06
AC004877.1	-1.161	-2.236	1.932E-04
PTPRG	-1.160	-2.235	2.130E-03
TYW5	-1.160	-2.235	0.000E+00
RBM25	-1.160	-2.235	0.000E+00
SNORD4A	-1.160	-2.234	8.470E-08
PHLPP2	-1.159	-2.234	0.000E+00
HACE1	-1.159	-2.233	0.000E+00
AL604028.2	-1.159	-2.233	2.060E-06
AC100827.2	-1.158	-2.232	4.658E-05
CHD1L	-1.158	-2.231	8.327E-09

ZXDA	-1.157	-2.230	0.000E+00
AC068587.4	-1.156	-2.228	2.886E-03
FNIP2	-1.155	-2.227	0.000E+00
AC096586.1	-1.155	-2.227	5.778E-05
PCSK5	-1.155	-2.226	0.000E+00
AL109614.1	-1.155	-2.226	3.253E-04
TRAJ8	-1.154	-2.225	2.970E-03
TIGD1	-1.154	-2.225	0.000E+00
AC007390.1	-1.154	-2.225	2.972E-06
EIF2AK3-DT	-1.153	-2.224	9.602E-09
GFOD1	-1.152	-2.223	0.000E+00
AC068790.3	-1.151	-2.220	9.811E-03
PET117	-1.150	-2.219	8.885E-05
ATXN7	-1.150	-2.219	1.121E-10
GNAL	-1.150	-2.219	8.892E-05
ZNF662	-1.149	-2.217	3.935E-08
CPED1	-1.149	-2.217	3.689E-08
PTBP2	-1.149	-2.217	0.000E+00
RFX8	-1.148	-2.217	1.246E-03
ZNF221	-1.147	-2.215	9.876E-03
FRG1DP	-1.147	-2.215	1.731E-03
GOLGA8B	-1.147	-2.215	0.000E+00
FZD8	-1.147	-2.215	5.111E-03
CENPF	-1.146	-2.213	5.167E-08
IRGM	-1.146	-2.213	8.525E-06
CNNM3-DT	-1.146	-2.213	3.025E-05
AC016074.2	-1.146	-2.213	2.964E-07
ADPRM	-1.146	-2.212	2.304E-09
CMYA5	-1.144	-2.210	4.288E-04
AC108134.2	-1.143	-2.209	2.284E-03
WWP1	-1.143	-2.209	0.000E+00
TET1	-1.143	-2.209	4.980E-06
NDUFAF2	-1.142	-2.207	7.932E-07
RNU6-638P	-1.142	-2.206	1.443E-03
CEP95	-1.142	-2.206	0.000E+00
ZNF484	-1.142	-2.206	0.000E+00
AC073283.1	-1.142	-2.206	2.220E-03
ZNF714	-1.141	-2.206	2.855E-10
CENPE	-1.141	-2.206	8.518E-07
IFTAP	-1.141	-2.205	1.913E-06
MIR222HG	-1.141	-2.205	7.453E-04
RNU6-5P	-1.140	-2.204	1.403E-06
LINC01237	-1.140	-2.204	7.839E-05
AL136169.1	-1.140	-2.204	1.434E-05
RPL11	-1.140	-2.204	1.038E-08
GSAP	-1.139	-2.203	0.000E+00
CD300E	-1.139	-2.202	0.000E+00
EVC	-1.139	-2.202	4.248E-05
AIMP1	-1.138	-2.201	0.000E+00
PNISR	-1.138	-2.201	0.000E+00
CCDC150	-1.138	-2.200	1.858E-03
ZNF804A	-1.137	-2.199	1.853E-05
NACA	-1.137	-2.199	0.000E+00
RNASE4	-1.137	-2.199	7.382E-03
SPOCK1	-1.137	-2.199	1.775E-07
NPR3	-1.136	-2.198	1.935E-03
SPIN4	-1.136	-2.198	1.322E-07
LINC01226	-1.134	-2.195	1.475E-03
FAM135A	-1.133	-2.193	0.000E+00
HERC2P3	-1.132	-2.192	1.085E-03
ENKUR	-1.132	-2.191	2.707E-04
JOSD1	-1.131	-2.191	0.000E+00
ASCC3	-1.131	-2.191	0.000E+00
LINC00342	-1.131	-2.190	0.000E+00
DOCK9	-1.130	-2.189	0.000E+00
AC011246.1	-1.130	-2.189	5.770E-03
AC027644.1	-1.130	-2.188	1.439E-04
FER	-1.129	-2.188	0.000E+00
AL355802.3	-1.128	-2.186	5.862E-04
STAG3L4	-1.128	-2.186	0.000E+00
YTHDC2	-1.128	-2.186	0.000E+00
CPS1	-1.128	-2.186	9.549E-03
ELAPOR2	-1.128	-2.185	3.217E-05
SMARCA1	-1.126	-2.182	6.611E-03
INSR	-1.126	-2.182	0.000E+00
KRT8P46	-1.125	-2.181	8.939E-06
HMBOX1	-1.124	-2.180	0.000E+00
ZNF280D	-1.124	-2.180	0.000E+00
PIBF1	-1.124	-2.179	0.000E+00
MIR7111	-1.124	-2.179	6.826E-03
FAM72A	-1.123	-2.178	2.332E-05
GCATP1	-1.122	-2.177	1.231E-04
GORAB	-1.122	-2.176	0.000E+00
ITGAV	-1.121	-2.176	0.000E+00
ZNF300	-1.121	-2.175	1.474E-08
KLHL3	-1.121	-2.175	0.000E+00
RPS3AP21	-1.120	-2.174	9.410E-05
THAP9-AS1	-1.120	-2.174	0.000E+00
LEPR	-1.120	-2.174	1.575E-07
AC097375.1	-1.120	-2.174	8.159E-03
AC099811.1	-1.120	-2.174	7.148E-04
SYCE2	-1.120	-2.174	8.735E-03
FANCB	-1.120	-2.173	2.524E-09
RIMKLB	-1.119	-2.172	0.000E+00
SNORD6	-1.118	-2.171	6.587E-04
ANKIB1	-1.118	-2.171	0.000E+00
AKAP12	-1.118	-2.171	1.740E-06
AC090607.2	-1.118	-2.170	1.564E-08
SYNE1	-1.117	-2.169	0.000E+00
PHF3	-1.117	-2.168	0.000E+00
ANKRD28	-1.116	-2.167	0.000E+00
AC002128.1	-1.116	-2.167	4.627E-04
DDX58	-1.115	-2.167	4.431E-06
ILDR2	-1.115	-2.166	1.570E-03
AC012615.1	-1.115	-2.166	4.772E-09
ZNF121	-1.115	-2.165	0.000E+00
MATN2	-1.114	-2.165	7.474E-04
ALG10B	-1.114	-2.165	3.034E-10
LMO7	-1.113	-2.163	0.000E+00
HIVEP2	-1.113	-2.163	0.000E+00

PHF10	-1.113	-2.163	0.000E+00
NRP1	-1.113	-2.163	3.102E-05
AL513534.1	-1.113	-2.162	1.292E-05
CAMK4	-1.112	-2.162	0.000E+00
DSC1	-1.112	-2.162	5.201E-04
TCF3P1	-1.112	-2.161	3.656E-03
NT5DC3	-1.112	-2.161	7.276E-10
KPNA5	-1.112	-2.161	3.474E-10
AC108108.1	-1.111	-2.160	2.219E-06
AC000123.2	-1.111	-2.160	2.889E-09
CRYBG3	-1.110	-2.158	0.000E+00
MAFF	-1.109	-2.157	7.405E-05
ATM	-1.109	-2.157	0.000E+00
RPS3AP47	-1.108	-2.156	5.010E-03
MED13L	-1.108	-2.156	1.194E-09
DTHD1	-1.108	-2.156	8.215E-06
AC064799.1	-1.108	-2.155	3.953E-03
RNF165	-1.107	-2.154	2.758E-06
THUMPD2	-1.107	-2.154	0.000E+00
AP3M2	-1.106	-2.153	3.265E-10
ZNF573	-1.106	-2.152	1.600E-07
C1orf50	-1.106	-2.152	0.000E+00
ANKRD29	-1.104	-2.150	4.789E-03
RPS23P8	-1.104	-2.149	3.170E-05
RNF125	-1.102	-2.147	0.000E+00
NRBF2P6	-1.102	-2.147	4.693E-05
UNC13B	-1.102	-2.146	4.091E-09
AC092794.1	-1.102	-2.146	1.220E-04
EAF1	-1.101	-2.145	9.457E-10
RPS3A	-1.101	-2.145	2.786E-06
SATB1	-1.101	-2.145	0.000E+00
KIAA2026	-1.101	-2.145	0.000E+00
GVINP1	-1.100	-2.143	2.393E-07
TNIK	-1.100	-2.143	0.000E+00
NEK10	-1.099	-2.143	3.058E-03
AC005838.2	-1.099	-2.142	7.887E-03
NKD1	-1.099	-2.142	1.426E-08
MPP3	-1.098	-2.140	7.658E-04
ARHGAP42	-1.097	-2.139	1.800E-05
CNNM2	-1.097	-2.139	0.000E+00
SCN11A	-1.096	-2.138	5.312E-03
LARP1B	-1.096	-2.138	0.000E+00
USP45	-1.096	-2.138	0.000E+00
AC139494.1	-1.096	-2.138	4.439E-03
SNRPD1	-1.095	-2.137	0.000E+00
ESF1	-1.095	-2.136	0.000E+00
AC009812.1	-1.095	-2.135	2.763E-09
AL391807.1	-1.094	-2.135	8.546E-04
ASIC1	-1.093	-2.133	1.970E-03
SENP7	-1.092	-2.132	1.192E-10
MIR3936HG	-1.092	-2.132	2.330E-07
ASH1L	-1.091	-2.131	0.000E+00
AC073115.2	-1.091	-2.130	9.784E-04
CAPN7	-1.091	-2.130	0.000E+00
CLOCK	-1.090	-2.129	0.000E+00

RASSF8	-1.090	-2.128	1.364E-03
NPM1	-1.089	-2.128	0.000E+00
CLECL1	-1.089	-2.128	2.479E-06
VPS13B	-1.089	-2.127	0.000E+00
LINC01374	-1.088	-2.126	7.200E-03
SNHG1	-1.088	-2.126	0.000E+00
NDUFS5	-1.088	-2.125	6.522E-07
HOOK1	-1.087	-2.125	3.173E-10
DTNA	-1.087	-2.125	1.162E-05
SEPSECS	-1.087	-2.125	0.000E+00
PRKCA	-1.087	-2.125	0.000E+00
MMS22L	-1.087	-2.125	0.000E+00
Z95114.3	-1.087	-2.124	1.188E-09
ZNF800	-1.086	-2.123	1.053E-10
CFL2	-1.086	-2.123	2.491E-08
RSRC2	-1.086	-2.123	0.000E+00
RUFY3	-1.085	-2.121	0.000E+00
TRPV3	-1.085	-2.121	7.610E-05
CSPP1	-1.084	-2.121	0.000E+00
AP002026.1	-1.084	-2.121	1.261E-09
AL357143.1	-1.084	-2.119	1.632E-05
NUCB2	-1.083	-2.119	0.000E+00
NRP2	-1.083	-2.118	3.598E-05
AC139769.1	-1.083	-2.118	4.385E-07
MPO	-1.083	-2.118	2.368E-04
HAUS3	-1.082	-2.118	0.000E+00
PIKFYVE	-1.082	-2.117	0.000E+00
PTPN13	-1.082	-2.117	1.009E-07
AP000254.2	-1.082	-2.117	5.597E-05
AKAP11	-1.082	-2.116	5.321E-10
GARRE1	-1.081	-2.116	4.863E-09
ZNF805	-1.081	-2.115	1.360E-08
AL139317.3	-1.081	-2.115	1.561E-03
CNKSR2	-1.080	-2.115	8.243E-08
ZNF667	-1.080	-2.114	1.286E-04
USP16	-1.080	-2.114	0.000E+00
SMC5	-1.080	-2.114	0.000E+00
SMAD7	-1.080	-2.114	2.089E-08
ODR4	-1.080	-2.114	0.000E+00
PLEKHH2	-1.079	-2.113	4.338E-03
ASPM	-1.079	-2.113	2.584E-04
INTS6L	-1.079	-2.113	0.000E+00
NUP62CL	-1.079	-2.112	1.350E-03
LCOR	-1.078	-2.112	0.000E+00
PCNPP1	-1.078	-2.111	7.168E-03
C9orf72	-1.078	-2.111	0.000E+00
COX6C	-1.078	-2.111	1.909E-08
SEZ6L	-1.078	-2.111	9.709E-07
TRAJ10	-1.077	-2.110	6.837E-04
HNRNPA1	-1.077	-2.109	0.000E+00
ZCCHC14	-1.076	-2.108	0.000E+00
RPS27	-1.075	-2.107	3.380E-06
LTV1	-1.075	-2.107	0.000E+00
TMEM269	-1.075	-2.107	3.558E-04
AC008115.4	-1.075	-2.107	6.807E-04

KCTD9	-1.075	-2.107	0.000E+00
IMPG2	-1.075	-2.107	8.319E-07
AC127502.1	-1.075	-2.107	4.015E-06
DNTTIP2	-1.075	-2.106	0.000E+00
GUSBP5	-1.074	-2.106	5.323E-03
TWF1	-1.074	-2.105	3.808E-09
ATG2B	-1.074	-2.105	0.000E+00
CNOT6L	-1.073	-2.104	4.545E-09
U2SURP	-1.073	-2.104	0.000E+00
AMOTL1	-1.073	-2.103	1.278E-08
ZNF577	-1.073	-2.103	0.000E+00
JMJD7	-1.073	-2.103	5.148E-05
COL4A3	-1.072	-2.102	4.034E-04
GRM2	-1.071	-2.101	2.278E-09
TASOR2	-1.070	-2.100	0.000E+00
CFH	-1.070	-2.099	2.437E-06
RASGRF2	-1.069	-2.098	0.000E+00
STARD9	-1.069	-2.098	4.958E-10
ARID5B	-1.069	-2.098	0.000E+00
FBXO33	-1.068	-2.097	0.000E+00
ZNF365	-1.068	-2.097	8.096E-07
MBIP	-1.068	-2.097	0.000E+00
RPL37	-1.068	-2.096	3.072E-06
RAD54L2	-1.067	-2.096	0.000E+00
TRIB1	-1.067	-2.095	6.346E-05
AC007216.3	-1.067	-2.095	8.596E-04
PRPF39	-1.067	-2.095	0.000E+00
AC110611.2	-1.067	-2.095	5.464E-04
TIPARP	-1.066	-2.094	2.419E-10
KATNAL2	-1.066	-2.093	5.966E-04
EIF4A2	-1.066	-2.093	0.000E+00
ANKRD12	-1.065	-2.093	0.000E+00
STK31	-1.065	-2.092	6.498E-04
MAP3K1	-1.064	-2.091	0.000E+00
APP	-1.064	-2.091	0.000E+00
MYO9A	-1.064	-2.090	0.000E+00
ZBTB39	-1.063	-2.090	0.000E+00
AC116158.1	-1.063	-2.090	1.568E-03
EPHA4	-1.063	-2.089	0.000E+00
DENND4C	-1.063	-2.089	0.000E+00
SORCS2	-1.063	-2.089	5.792E-04
SLC38A11	-1.062	-2.088	2.668E-03
MRPL32	-1.062	-2.088	0.000E+00
SLC16A10	-1.061	-2.086	1.575E-06
C1orf21	-1.061	-2.086	5.541E-09
ZNF677	-1.061	-2.086	1.186E-07
ITGB1BP2	-1.060	-2.085	1.741E-07
CWC27	-1.060	-2.085	0.000E+00
GPM6B	-1.060	-2.084	7.441E-03
AL356417.3	-1.059	-2.084	2.728E-03
UHRF2	-1.059	-2.083	0.000E+00
CYP46A1	-1.058	-2.082	5.099E-04
PTPRM	-1.058	-2.082	1.187E-06
ZNF609	-1.058	-2.081	0.000E+00
KCNQ5	-1.057	-2.081	3.109E-08

NEDD4	-1.057	-2.081	0.000E+00
SECISBP2L	-1.057	-2.080	0.000E+00
AC093525.8	-1.057	-2.080	1.159E-06
ARGLU1	-1.057	-2.080	0.000E+00
USP47	-1.056	-2.080	0.000E+00
CCDC18-AS1	-1.056	-2.080	0.000E+00
ATP13A4	-1.056	-2.079	4.269E-05
NDUFV2P1	-1.055	-2.078	3.640E-03
BNIP1	-1.054	-2.077	0.000E+00
NDUFA5	-1.054	-2.076	0.000E+00
AP001931.2	-1.053	-2.075	1.190E-03
USF3	-1.053	-2.075	1.907E-09
RASD1	-1.053	-2.075	2.940E-03
ANKHD1	-1.053	-2.075	2.148E-10
LGR4	-1.053	-2.075	7.481E-07
ZKSCAN8	-1.053	-2.075	0.000E+00
AL512306.3	-1.053	-2.074	8.565E-06
PRPF4B	-1.052	-2.074	0.000E+00
SESTD1	-1.052	-2.073	0.000E+00
XPO4	-1.051	-2.073	0.000E+00
ZNF236	-1.051	-2.073	0.000E+00
NAF1	-1.051	-2.072	0.000E+00
LATS1	-1.051	-2.072	0.000E+00
ACADSB	-1.051	-2.072	0.000E+00
CENPW	-1.050	-2.071	3.920E-06
HECTD2	-1.050	-2.071	3.676E-07
SLC19A2	-1.050	-2.071	3.889E-08
ZNF318	-1.050	-2.070	0.000E+00
TET3	-1.049	-2.070	0.000E+00
ANKRD10-IT1	-1.048	-2.067	1.497E-05
BCLAF1	-1.048	-2.067	0.000E+00
SFPQ	-1.047	-2.066	0.000E+00
TFCP2L1	-1.047	-2.066	1.440E-03
AL049840.6	-1.046	-2.065	1.576E-05
FBXW7	-1.046	-2.065	0.000E+00
GOLGA4	-1.046	-2.064	0.000E+00
PRPF18	-1.045	-2.064	0.000E+00
CEP83	-1.045	-2.063	0.000E+00
MAP3K8	-1.045	-2.063	0.000E+00
POLK	-1.045	-2.063	0.000E+00
RSL24D1	-1.044	-2.062	0.000E+00
LYPLAL1-DT	-1.044	-2.061	4.428E-04
EME1	-1.043	-2.061	4.378E-05
SETD7	-1.043	-2.061	0.000E+00
SEPTIN7P13	-1.042	-2.060	0.000E+00
ERC1	-1.042	-2.059	0.000E+00
AL135791.1	-1.042	-2.059	1.120E-03
TIPARP-AS1	-1.042	-2.059	6.749E-06
DNAH6	-1.042	-2.059	5.647E-06
ZNF37BP	-1.042	-2.058	0.000E+00
KLRC2	-1.041	-2.058	9.826E-03
GGTA1	-1.041	-2.058	1.250E-05
GPATCH2L	-1.040	-2.057	0.000E+00
TEC	-1.040	-2.056	0.000E+00
DNAJC13	-1.040	-2.056	0.000E+00

SUCO	-1.039	-2.055	0.000E+00
PLK4	-1.039	-2.055	9.737E-09
PPIG	-1.039	-2.055	0.000E+00
GCC2	-1.039	-2.054	0.000E+00
CEP120	-1.038	-2.054	0.000E+00
CHD2	-1.038	-2.053	0.000E+00
CLEC6A	-1.038	-2.053	5.497E-05
ZC3H15	-1.036	-2.051	0.000E+00
AC073367.1	-1.036	-2.050	7.495E-04
USP31	-1.035	-2.050	0.000E+00
CRY1	-1.035	-2.049	0.000E+00
DHX36	-1.035	-2.049	3.040E-08
MLF1	-1.035	-2.049	1.455E-03
MGP	-1.032	-2.045	6.191E-04
AC135050.5	-1.032	-2.045	2.675E-06
USP53	-1.032	-2.044	9.385E-06
CRACR2A	-1.031	-2.044	0.000E+00
IL6ST	-1.030	-2.042	3.792E-08
TARBP1	-1.030	-2.042	0.000E+00
ERVK13-1	-1.029	-2.041	0.000E+00
DDIT4	-1.029	-2.041	3.573E-06
SERTAD2	-1.029	-2.041	0.000E+00
MITF	-1.029	-2.040	1.545E-09
NBPF8	-1.028	-2.040	2.096E-08
ICE1	-1.028	-2.039	0.000E+00
GOLGA6L9	-1.028	-2.039	3.534E-05
DPY19L1P1	-1.027	-2.038	5.997E-03
RPS15A	-1.027	-2.038	1.349E-05
RGPD8	-1.027	-2.038	2.051E-04
AC009831.3	-1.027	-2.037	8.732E-03
SC5D	-1.027	-2.037	2.166E-06
KRT73	-1.026	-2.037	1.526E-03
U3	-1.026	-2.036	2.609E-03
SIGLEC1	-1.026	-2.036	2.642E-03
CDK17	-1.025	-2.035	3.242E-07
AC008875.2	-1.025	-2.035	0.000E+00
CCDC82	-1.025	-2.035	1.496E-08
LINC00674	-1.025	-2.034	0.000E+00
TXNDC5	-1.024	-2.034	8.516E-04
MRC1	-1.024	-2.033	7.192E-05
CPNE8	-1.023	-2.032	0.000E+00
WDR43	-1.022	-2.031	0.000E+00
ASXL1	-1.022	-2.031	0.000E+00
DHX15	-1.022	-2.030	0.000E+00
NSD1	-1.021	-2.029	0.000E+00
ZNF532	-1.021	-2.029	0.000E+00
LDHB	-1.021	-2.029	1.600E-06
FPGT-TNNI3K	-1.020	-2.028	1.040E-04
KIF18A	-1.020	-2.028	1.341E-05
GOLGA8N	-1.019	-2.027	9.789E-05
CAMSAP1	-1.019	-2.027	0.000E+00
MAN1A2	-1.019	-2.026	0.000E+00
AMMECR1	-1.018	-2.025	0.000E+00
ITSN1	-1.018	-2.025	0.000E+00
LRRC39	-1.018	-2.025	4.980E-06
KMT2C	-1.017	-2.024	3.312E-08
PDP1	-1.017	-2.024	0.000E+00
PBDC1	-1.017	-2.024	2.902E-08
SCN9A	-1.017	-2.024	8.330E-05
HSF2	-1.016	-2.022	0.000E+00
PDPR	-1.015	-2.022	0.000E+00
CAPS2	-1.015	-2.021	1.658E-08
AC073861.1	-1.015	-2.021	1.853E-04
RASA1	-1.015	-2.021	0.000E+00
CEP126	-1.015	-2.021	3.837E-10
CSTA	-1.015	-2.021	4.194E-07
KIF5B	-1.014	-2.019	0.000E+00
AC010680.4	-1.014	-2.019	2.220E-07
TENT4A	-1.013	-2.018	0.000E+00
PEX1	-1.013	-2.018	0.000E+00
BHLHE40	-1.012	-2.017	5.711E-09
GPR174	-1.012	-2.017	0.000E+00
ERCC6L	-1.012	-2.017	8.213E-05
RAD50	-1.012	-2.017	1.710E-08
NAA25	-1.012	-2.017	0.000E+00
TCERG1	-1.012	-2.016	0.000E+00
AL359644.1	-1.011	-2.016	9.205E-03
DUSP10	-1.009	-2.013	1.964E-07
STIM2	-1.008	-2.012	0.000E+00
KMT2A	-1.007	-2.010	0.000E+00
GGNBP2	-1.006	-2.009	5.370E-04
PARP15	-1.006	-2.009	0.000E+00
AC240274.1	-1.006	-2.008	3.726E-06
AC107223.1	-1.005	-2.007	1.836E-03
RWDD1	-1.005	-2.007	1.224E-09
SAMD12	-1.004	-2.006	3.770E-06
FASTKD5	-1.003	-2.004	0.000E+00
LINC01597	-1.003	-2.004	9.320E-03
EFCAB7	-1.003	-2.004	0.000E+00
AHCTF1P1	-1.003	-2.004	4.440E-03
TCAF1	-1.002	-2.003	1.371E-04
SLC9A7P1	-1.002	-2.003	1.008E-05
AL161421.1	-1.001	-2.002	1.881E-06
ATAD2	-1.001	-2.002	0.000E+00
KDM4C	-1.001	-2.002	0.000E+00
MRPL45P2	-1.001	-2.002	0.000E+00
gen-01	-1.001	-2.002	8.145E-10
RPS23	-1.001	-2.002	2.742E-07
NDST2	-1.001	-2.001	4.911E-08
MIB1	-1.001	-2.001	1.587E-09
COL6A3	-1.000	-2.001	1.118E-06
AF165147.1	-1.000	-2.000	1.911E-04
TMTC4	-1.000	-2.000	0.000E+00
ARHGAP29	-0.999	-1.999	6.031E-05
DYNC1LI2	-0.999	-1.999	0.000E+00
NEXN	-0.999	-1.998	1.605E-05
SAMD8	-0.998	-1.998	0.000E+00
LRRC8B	-0.998	-1.998	1.300E-09
UTP15	-0.997	-1.996	0.000E+00
UICLM	-0.997	-1.995	4.951E-04

POLI	-0.996	-1.995	0.000E+00
UBR1	-0.996	-1.995	0.000E+00
DBF4	-0.996	-1.994	6.607E-08
TP53BP2	-0.996	-1.994	0.000E+00
RN7SL481P	-0.996	-1.994	4.546E-04
CLIP4	-0.996	-1.994	0.000E+00
ZC3H7A	-0.994	-1.992	0.000E+00
STX17-AS1	-0.994	-1.992	7.426E-03
AL928711.1	-0.994	-1.991	7.526E-04
AL355581.1	-0.993	-1.990	2.403E-07
EIF2AK4	-0.993	-1.990	0.000E+00
COPS2	-0.993	-1.990	0.000E+00
GDF7	-0.992	-1.989	1.507E-03
YRDC	-0.992	-1.989	0.000E+00
AK9	-0.991	-1.987	0.000E+00
MYOF	-0.991	-1.987	6.175E-06
AL390195.1	-0.990	-1.987	6.638E-05
DUSP8P5	-0.989	-1.984	3.450E-08
MAST4	-0.989	-1.984	0.000E+00
TOMM7	-0.988	-1.983	2.156E-06
RNF103	-0.987	-1.982	1.667E-08
SMARCA2	-0.987	-1.982	0.000E+00
REV1	-0.985	-1.979	0.000E+00
FKTN	-0.985	-1.979	0.000E+00
PHLDB2	-0.985	-1.979	3.463E-07
CDR2	-0.985	-1.979	6.880E-10
DOP1A	-0.984	-1.979	0.000E+00
ATR	-0.984	-1.979	0.000E+00
ABCA6	-0.984	-1.978	7.253E-03
AC005332.3	-0.984	-1.978	5.177E-10
LIF	-0.984	-1.978	8.711E-03
GPATCH8	-0.984	-1.978	0.000E+00
CDS1	-0.984	-1.977	1.682E-03
SNRPGP14	-0.983	-1.977	1.140E-03
SLITRK5	-0.983	-1.977	7.775E-03
TAOK1	-0.983	-1.977	0.000E+00
CLDND1	-0.983	-1.977	0.000E+00
CARF	-0.983	-1.976	7.866E-10
LINC01473	-0.982	-1.976	4.817E-04
SNORD94	-0.982	-1.975	3.015E-04
EDNRB-AS1	-0.981	-1.974	9.779E-04
THUMPD1	-0.981	-1.974	0.000E+00
GNPTAB	-0.980	-1.972	0.000E+00
RPL23P2	-0.980	-1.972	1.189E-04
IDI1	-0.979	-1.972	2.384E-10
KNDC1	-0.979	-1.971	2.514E-03
BCL2L2	-0.979	-1.971	0.000E+00
ETAA1	-0.978	-1.970	0.000E+00
FAM184A	-0.978	-1.970	7.933E-07
FAM111B	-0.978	-1.970	4.726E-04
UBR5	-0.978	-1.969	0.000E+00
FKBP14	-0.978	-1.969	0.000E+00
MEX3C	-0.977	-1.968	0.000E+00
AL355987.4	-0.977	-1.968	6.459E-08
USP6NL	-0.976	-1.968	1.259E-10

CA8	-0.976	-1.967	4.726E-04
AL356489.2	-0.976	-1.967	6.951E-03
SLFN13	-0.976	-1.967	0.000E+00
ZFP1	-0.975	-1.966	2.554E-07
CHN2	-0.975	-1.966	2.108E-05
USP36	-0.975	-1.965	8.261E-09
PDE3B	-0.975	-1.965	0.000E+00
NBPF12	-0.975	-1.965	0.000E+00
SCAF8	-0.974	-1.965	0.000E+00
FBXO3	-0.974	-1.964	0.000E+00
COX7C	-0.974	-1.964	1.387E-06
GAS5	-0.973	-1.963	4.990E-09
DDX5	-0.973	-1.963	0.000E+00
ZEB2-AS1	-0.973	-1.963	1.065E-07
MBNL2	-0.972	-1.962	7.086E-09
VPS54	-0.972	-1.962	0.000E+00
GOLGA6L5P	-0.972	-1.961	1.732E-03
ZNF558	-0.972	-1.961	0.000E+00
ANO6	-0.972	-1.961	0.000E+00
MYO10	-0.971	-1.961	1.859E-05
SLIRP	-0.971	-1.961	7.032E-08
ABCA5	-0.971	-1.960	1.068E-10
PAPLN	-0.970	-1.959	3.133E-06
C5	-0.970	-1.959	0.000E+00
AP001033.2	-0.969	-1.958	1.631E-03
CRIM1	-0.969	-1.958	7.919E-07
ANKRD31	-0.969	-1.958	1.880E-03
LINC01036	-0.969	-1.957	9.561E-03
MT-ND6	-0.969	-1.957	3.646E-04
BBX	-0.968	-1.956	0.000E+00
ARHGAP5	-0.967	-1.955	1.803E-08
FANCM	-0.967	-1.955	0.000E+00
SHTN1	-0.967	-1.955	4.732E-07
ACVR2A	-0.967	-1.955	0.000E+00
SCAPER	-0.967	-1.955	0.000E+00
AL390195.2	-0.966	-1.954	7.235E-07
CPLANE1	-0.966	-1.954	2.322E-09
INPP5F	-0.965	-1.952	0.000E+00
ADCY9	-0.965	-1.952	0.000E+00
CHIC1	-0.965	-1.952	0.000E+00
CLEC12B	-0.965	-1.952	4.015E-06
MCPH1	-0.964	-1.951	0.000E+00
ZC3H12C	-0.964	-1.951	1.348E-05
MINDY3	-0.964	-1.951	0.000E+00
LINC01801	-0.964	-1.951	1.932E-04
FAM198B-AS1	-0.964	-1.951	2.339E-06
AHR	-0.964	-1.951	1.223E-05
FAM221A	-0.964	-1.950	1.540E-09
TGFBRAP1	-0.963	-1.950	0.000E+00
NDUFA1	-0.963	-1.949	7.058E-06
ZNF451	-0.963	-1.949	0.000E+00
DNAJB11	-0.963	-1.949	0.000E+00
NMD3	-0.962	-1.948	0.000E+00
MGA	-0.962	-1.948	0.000E+00
ZNF876P	-0.962	-1.948	3.541E-04

CMC1	-0.962	-1.948	9.667E-07
FEM1C	-0.961	-1.947	2.090E-05
AFAP1	-0.961	-1.947	2.469E-07
ADPGK-AS1	-0.960	-1.946	1.417E-03
ADAM12	-0.960	-1.945	1.591E-03
ZNF706	-0.960	-1.945	0.000E+00
CECR7	-0.960	-1.945	3.535E-04
SPRY1	-0.959	-1.945	6.129E-04
CCT6P1	-0.959	-1.944	0.000E+00
TTC21B	-0.959	-1.944	0.000E+00
PRRC2C	-0.959	-1.943	0.000E+00
CEP192	-0.959	-1.943	0.000E+00
ITGB3BP	-0.959	-1.943	0.000E+00
WRN	-0.958	-1.943	0.000E+00
DENND1B	-0.957	-1.941	1.424E-09
NSRP1	-0.957	-1.941	0.000E+00
LRRC43	-0.957	-1.941	3.315E-03
ECPAS	-0.957	-1.941	0.000E+00
TOGARAM1	-0.956	-1.940	0.000E+00
SH2D1B	-0.956	-1.940	1.170E-06
CCDC186	-0.956	-1.940	1.134E-07
CFAP44	-0.956	-1.940	0.000E+00
RNVU1-4	-0.956	-1.940	7.481E-03
CACNA2D2	-0.956	-1.940	1.321E-07
KIF20B	-0.955	-1.939	4.434E-08
MIGA1	-0.955	-1.939	0.000E+00
PUM1	-0.955	-1.939	0.000E+00
AL355916.1	-0.955	-1.938	6.446E-05
MKI67	-0.954	-1.937	2.696E-04
MYO5A	-0.954	-1.937	0.000E+00
TBC1D8	-0.954	-1.937	0.000E+00
ATP13A3	-0.953	-1.936	1.612E-08
CBLB	-0.953	-1.936	0.000E+00
MORN2	-0.953	-1.936	3.898E-04
MAPK8	-0.953	-1.935	0.000E+00
PLEKHA1	-0.952	-1.934	1.689E-10
RAP2C-AS1	-0.951	-1.933	2.790E-06
IRS1	-0.950	-1.933	4.297E-07
UBXN7	-0.950	-1.932	0.000E+00
DGKE	-0.950	-1.932	0.000E+00
SCARB2	-0.949	-1.930	0.000E+00
RRAGB	-0.949	-1.930	0.000E+00
VTI1A	-0.948	-1.929	0.000E+00
FLVCR2	-0.948	-1.929	1.408E-08
HELLPAR	-0.947	-1.928	1.462E-03
SRGAP2B	-0.947	-1.928	1.311E-06
SNRPE	-0.947	-1.928	8.240E-08
BAALC	-0.947	-1.928	5.896E-03
PAN2	-0.947	-1.928	0.000E+00
SSB	-0.946	-1.926	0.000E+00
CEP350	-0.945	-1.925	0.000E+00
USP24	-0.945	-1.925	0.000E+00
RPS2P7	-0.944	-1.924	3.870E-04
SLFN12	-0.944	-1.924	1.280E-10
CDK6	-0.944	-1.924	0.000E+00
ABCB1	-0.943	-1.923	2.294E-09
ADAM22	-0.943	-1.922	5.801E-08
PRPF38B	-0.943	-1.922	0.000E+00
MCF2L2	-0.943	-1.922	2.402E-06
SF3B1	-0.943	-1.922	0.000E+00
KNTC1	-0.942	-1.922	0.000E+00
PLEKHM3	-0.942	-1.922	0.000E+00
RAPH1	-0.942	-1.921	3.635E-06
AL512408.1	-0.941	-1.920	7.975E-03
DOCK7	-0.941	-1.920	0.000E+00
RPL7	-0.941	-1.920	2.091E-06
COL4A4	-0.941	-1.920	9.445E-04
TCF4	-0.941	-1.920	7.774E-09
CPEB3	-0.941	-1.920	1.590E-09
AC034236.1	-0.941	-1.920	6.941E-03
SON	-0.939	-1.918	0.000E+00
RICTOR	-0.939	-1.917	2.855E-05
NSA2	-0.937	-1.915	1.996E-06
AC087163.2	-0.937	-1.914	8.040E-05
AL049828.1	-0.937	-1.914	9.153E-04
HIF1A	-0.937	-1.914	3.778E-05
CNTRL	-0.936	-1.914	0.000E+00
MAP3K20	-0.936	-1.914	0.000E+00
AC138409.2	-0.936	-1.914	2.753E-09
ORC6	-0.936	-1.913	4.444E-06
TMEM202-AS1	-0.935	-1.912	1.213E-04
EIF4HP1	-0.935	-1.912	8.640E-05
OTUD3	-0.935	-1.912	0.000E+00
ATRN	-0.934	-1.911	0.000E+00
RPS7P1	-0.934	-1.911	4.701E-04
ZNF254	-0.934	-1.910	0.000E+00
VPS13D	-0.934	-1.910	0.000E+00
BACE1	-0.933	-1.910	1.604E-10
SERPINE2	-0.933	-1.909	8.997E-04
CERS6	-0.933	-1.909	0.000E+00
ATRX	-0.933	-1.909	3.176E-09
ZNF273	-0.933	-1.909	1.412E-09
TSPYL2	-0.931	-1.907	1.074E-07
LDLR	-0.931	-1.906	2.526E-08
GANC	-0.930	-1.906	0.000E+00
ZNF518A	-0.929	-1.904	1.455E-09
AGAP1	-0.929	-1.903	1.176E-06
TOB1	-0.929	-1.903	2.305E-09
LINC01252	-0.928	-1.903	4.500E-03
AL133444.1	-0.928	-1.903	5.498E-03
EPB41L3	-0.928	-1.903	3.028E-09
LRIG1	-0.928	-1.903	6.617E-07
LUC7L3	-0.928	-1.902	0.000E+00
ADCY6	-0.928	-1.902	2.357E-04
LINC02615	-0.928	-1.902	4.869E-06
SOCS3	-0.927	-1.901	3.332E-05
N4BP2L2-IT2	-0.927	-1.901	0.000E+00
PEG10	-0.927	-1.901	8.786E-04
AC009812.4	-0.926	-1.901	5.711E-04
PHC1P1	-0.926	-1.900	1.913E-04

RPL35A	-0.926	-1.900	1.266E-05
CAPRIN2	-0.926	-1.899	0.000E+00
MTCP1	-0.925	-1.898	2.133E-05
ULK2	-0.925	-1.898	0.000E+00
ITGA4	-0.925	-1.898	0.000E+00
CDKN2B-AS1	-0.924	-1.898	4.748E-05
IQGAP2	-0.924	-1.897	0.000E+00
SUB1	-0.923	-1.896	1.752E-10
EIF3A	-0.922	-1.895	0.000E+00
CEP135	-0.922	-1.895	1.350E-10
HTR7P1	-0.922	-1.894	2.438E-10
WDR75	-0.922	-1.894	0.000E+00
SEC61G	-0.922	-1.894	4.599E-08
AL592183.1	-0.922	-1.894	1.834E-04
TBC1D4	-0.921	-1.894	0.000E+00
HDX	-0.921	-1.894	5.443E-07
AC068050.1	-0.921	-1.894	1.244E-04
MAP7D2	-0.921	-1.893	1.523E-04
HELB	-0.921	-1.893	6.833E-05
G2E3	-0.921	-1.893	1.045E-06
ABI2	-0.921	-1.893	0.000E+00
VRK1	-0.920	-1.892	0.000E+00
NR2C1	-0.920	-1.892	0.000E+00
HEATR5B	-0.920	-1.892	0.000E+00
ZC3HAV1L	-0.920	-1.892	9.047E-04
TMF1	-0.920	-1.892	3.091E-09
FUBP1	-0.919	-1.891	0.000E+00
PDK1	-0.919	-1.891	0.000E+00
MCC	-0.919	-1.891	8.066E-05
ZNF107	-0.919	-1.890	5.914E-07
TATDN1	-0.918	-1.890	1.249E-09
TANC2	-0.918	-1.890	1.776E-10
OTUD6B	-0.918	-1.889	3.197E-07
RAB20	-0.918	-1.889	4.005E-06
CCR6	-0.918	-1.889	4.216E-05
FSD1L	-0.918	-1.889	9.867E-06
BRMS1L	-0.918	-1.889	1.766E-07
FLNB	-0.917	-1.889	0.000E+00
SPAG5	-0.917	-1.889	0.000E+00
GUCY2D	-0.917	-1.889	6.792E-03
ZHX3	-0.917	-1.888	0.000E+00
NDUFS4	-0.917	-1.888	1.158E-07
ZNF808	-0.917	-1.888	6.660E-09
SDE2	-0.917	-1.888	3.086E-06
PPTC7	-0.917	-1.888	0.000E+00
DNAJB14	-0.916	-1.887	3.285E-06
EXOG	-0.916	-1.887	0.000E+00
LARP7	-0.916	-1.886	0.000E+00
SPEF2	-0.915	-1.886	0.000E+00
EIF3E	-0.915	-1.886	0.000E+00
HERC2P2	-0.915	-1.886	1.843E-03
AC116533.1	-0.915	-1.886	4.399E-03
AL590064.1	-0.915	-1.885	0.000E+00
GALNT11	-0.915	-1.885	0.000E+00
AC004825.2	-0.914	-1.885	3.301E-03
ZNF317	-0.914	-1.885	0.000E+00
ZEB2	-0.914	-1.885	0.000E+00
PACRGL	-0.914	-1.884	0.000E+00
THOC2	-0.914	-1.884	0.000E+00
LINC02391	-0.914	-1.884	8.242E-03
LAMP5	-0.913	-1.883	3.435E-05
CCDC141	-0.913	-1.883	3.418E-04
WHAMM	-0.913	-1.883	0.000E+00
DOCK3	-0.913	-1.882	4.343E-08
HMGN3	-0.912	-1.882	0.000E+00
MBNL1	-0.912	-1.881	0.000E+00
LAYN	-0.911	-1.881	7.998E-03
NRCAM	-0.911	-1.880	1.852E-03
DOCK10	-0.910	-1.879	0.000E+00
IREB2	-0.909	-1.878	0.000E+00
RNF213	-0.909	-1.878	2.484E-10
SAMSN1	-0.908	-1.877	1.269E-06
SLC36A4	-0.908	-1.876	0.000E+00
ROCK1	-0.907	-1.875	1.580E-08
NEMF	-0.906	-1.874	0.000E+00
EPC2	-0.906	-1.874	0.000E+00
ELK4	-0.906	-1.873	0.000E+00
ZNF605	-0.905	-1.873	0.000E+00
ABHD13	-0.905	-1.872	7.754E-08
ITPR1	-0.904	-1.872	9.699E-10
EPM2AIP1	-0.904	-1.872	0.000E+00
RAG1	-0.904	-1.871	8.922E-03
AL355075.5	-0.904	-1.871	1.455E-03
TRAJ4	-0.903	-1.871	1.522E-03
MT-TL1	-0.903	-1.870	3.346E-04
MAP4K5	-0.903	-1.870	0.000E+00
AF127577.4	-0.903	-1.870	2.822E-04
SDR42E1	-0.903	-1.869	2.017E-09
DLG1	-0.902	-1.869	0.000E+00
VEGFA	-0.902	-1.869	4.352E-05
ANKRD49	-0.901	-1.867	0.000E+00
ATP2B1-AS1	-0.901	-1.867	1.995E-03
DAPK1	-0.901	-1.867	0.000E+00
PYROXD1	-0.901	-1.867	0.000E+00
LINC02705	-0.901	-1.867	7.803E-03
LCORL	-0.901	-1.867	3.004E-08
SEC22B4P	-0.900	-1.866	2.007E-07
KCNC4	-0.900	-1.866	2.441E-10
WDR19	-0.900	-1.866	0.000E+00
ZBTB40	-0.900	-1.866	0.000E+00
DZIP1L	-0.899	-1.865	4.890E-03
ITK	-0.899	-1.865	0.000E+00
RPAP2	-0.899	-1.865	0.000E+00
PARD3	-0.898	-1.864	2.749E-03
WDR27	-0.898	-1.863	0.000E+00
VWA8	-0.897	-1.863	0.000E+00
FAM66C	-0.897	-1.863	3.228E-09
C2orf69	-0.897	-1.863	4.544E-07
LRBA	-0.897	-1.862	0.000E+00
ZSCAN30	-0.897	-1.862	0.000E+00

PFKFB3	-0.896	-1.862	1.383E-03
HOMER1	-0.896	-1.862	3.946E-05
KATNAL1	-0.896	-1.861	1.435E-07
FBXO32	-0.896	-1.861	2.509E-09
ETS1	-0.896	-1.860	0.000E+00
SFMBT1	-0.895	-1.860	0.000E+00
ZFP69B	-0.895	-1.860	1.016E-06
AF064858.1	-0.895	-1.860	2.791E-03
CPEB2	-0.895	-1.859	1.409E-05
TBCA	-0.895	-1.859	8.890E-08
TGFBI	-0.894	-1.859	4.562E-10
ZNF541	-0.894	-1.858	2.502E-03
THOC1	-0.894	-1.858	0.000E+00
DAAM1	-0.894	-1.858	0.000E+00
SATB1-AS1	-0.893	-1.858	0.000E+00
ZSCAN25	-0.893	-1.857	0.000E+00
SMG1	-0.893	-1.856	6.797E-08
GXYLT1	-0.892	-1.856	0.000E+00
PDZD8	-0.892	-1.856	5.387E-06
LSM3	-0.891	-1.855	2.034E-06
KDM7A	-0.891	-1.855	0.000E+00
AAK1	-0.891	-1.855	0.000E+00
SH3RF1	-0.891	-1.854	7.475E-06
SNX29	-0.891	-1.854	0.000E+00
SLC38A1	-0.891	-1.854	0.000E+00
CKAP2L	-0.890	-1.854	7.726E-03
DUTP6	-0.890	-1.854	9.532E-03
CELSR2	-0.890	-1.854	1.621E-08
AC018644.1	-0.889	-1.852	8.544E-04
SHANK1	-0.889	-1.852	5.758E-03
SLC25A27	-0.889	-1.852	3.732E-04
LGALS8	-0.889	-1.851	0.000E+00
SMURF1	-0.888	-1.851	4.473E-08
CLCN5	-0.888	-1.851	0.000E+00
AC004803.1	-0.888	-1.851	1.066E-03
COG3	-0.888	-1.851	0.000E+00
GPATCH2	-0.888	-1.850	0.000E+00
APLF	-0.888	-1.850	3.209E-09
IFT74	-0.887	-1.850	0.000E+00
HGF	-0.887	-1.850	4.660E-07
CCDC138	-0.887	-1.849	0.000E+00
FAM91A1	-0.887	-1.849	2.868E-06
REV3L	-0.887	-1.849	2.089E-09
ZC2HC1A	-0.887	-1.849	4.798E-06
ZNF551	-0.886	-1.849	1.355E-07
RNASEH1	-0.886	-1.848	0.000E+00
SYNE3	-0.886	-1.848	0.000E+00
FEM1B	-0.886	-1.848	0.000E+00
AC011815.1	-0.886	-1.848	6.781E-08
GBP1P1	-0.886	-1.847	8.442E-03
RPL39P3	-0.885	-1.847	4.160E-03
UQCRH	-0.885	-1.847	2.257E-07
PTCH1	-0.885	-1.847	3.591E-08
MAN2A1	-0.885	-1.847	0.000E+00
AC084824.1	-0.884	-1.846	0.000E+00

GOLGB1	-0.884	-1.846	0.000E+00
CMPK2	-0.884	-1.846	5.280E-03
TLN2	-0.884	-1.845	2.772E-04
CUL5	-0.884	-1.845	6.333E-10
GOLIM4	-0.884	-1.845	1.489E-09
KIAA1328	-0.884	-1.845	0.000E+00
PPP1R15B	-0.883	-1.845	1.609E-08
OAS3	-0.883	-1.844	1.025E-03
LRRC58	-0.883	-1.844	3.097E-09
EMC2	-0.883	-1.844	0.000E+00
LTN1	-0.883	-1.844	1.718E-07
PROX2	-0.883	-1.844	6.996E-03
IFT57	-0.883	-1.844	0.000E+00
AGFG1	-0.883	-1.844	0.000E+00
CALU	-0.882	-1.842	0.000E+00
ZDHHC17	-0.881	-1.842	9.099E-09
GLUD1P3	-0.881	-1.841	2.745E-10
LINS1	-0.881	-1.841	0.000E+00
AL110114.1	-0.880	-1.841	1.351E-05
FBN1	-0.880	-1.841	3.171E-03
LINC00987	-0.880	-1.840	1.546E-04
SETD9	-0.880	-1.840	1.222E-06
CLASP1	-0.879	-1.839	0.000E+00
CREBZF	-0.878	-1.838	0.000E+00
FAM161A	-0.878	-1.838	7.138E-05
MTHFD2L	-0.878	-1.838	6.766E-09
DNM1L	-0.878	-1.838	0.000E+00
ZNF23	-0.878	-1.838	5.116E-04
RHOBTB3	-0.877	-1.837	1.826E-04
KYNU	-0.877	-1.837	4.754E-10
FOXJ3	-0.877	-1.837	0.000E+00
NSUN6	-0.877	-1.837	0.000E+00
SMC4	-0.877	-1.836	0.000E+00
RELCH	-0.876	-1.836	0.000E+00
GRK3	-0.876	-1.836	0.000E+00
ZNF674-AS1	-0.876	-1.836	0.000E+00
PLCH2	-0.876	-1.836	2.088E-04
POLQ	-0.875	-1.834	5.896E-04
ATP5ME	-0.875	-1.834	1.857E-04
HUWE1	-0.875	-1.834	0.000E+00
DNAJB1	-0.874	-1.833	2.997E-10
MED14OS	-0.874	-1.832	8.108E-03
MED21	-0.874	-1.832	9.333E-08
TRAK1	-0.874	-1.832	0.000E+00
DYNC1H1	-0.873	-1.832	0.000E+00
TPT1P9	-0.873	-1.832	1.615E-04
TFDP2	-0.873	-1.832	0.000E+00
NHLRC3	-0.873	-1.832	0.000E+00
PRNP	-0.873	-1.831	0.000E+00
SLC11A2	-0.873	-1.831	0.000E+00
ZNF34	-0.873	-1.831	0.000E+00
RIF1	-0.873	-1.831	2.898E-09
CC2D2A	-0.873	-1.831	1.453E-03
TAB3	-0.873	-1.831	1.920E-09
ZHX1-C8orf76	-0.873	-1.831	1.706E-03

LRRC37A2	-0.872	-1.830	3.745E-04
AC233976.1	-0.872	-1.830	1.753E-05
SPEN	-0.872	-1.830	0.000E+00
ALOX15B	-0.871	-1.829	4.035E-03
KLRB1	-0.870	-1.828	1.475E-04
COX7A2	-0.870	-1.828	9.515E-07
CCDC66	-0.870	-1.828	0.000E+00
CCNT2	-0.870	-1.827	0.000E+00
SNX9	-0.869	-1.827	2.885E-07
ATP2B1	-0.869	-1.826	2.043E-06
ZDHHC20	-0.869	-1.826	1.454E-08
NCEH1	-0.868	-1.826	0.000E+00
ZBTB37	-0.868	-1.825	3.158E-07
ARMCX5-GPRASP2	-0.868	-1.825	3.454E-07
RPS21	-0.868	-1.825	2.581E-04
STAB1	-0.868	-1.825	1.283E-06
SKIL	-0.867	-1.824	1.526E-04
RRN3P1	-0.867	-1.824	0.000E+00
AC016394.2	-0.867	-1.824	2.696E-06
KDM3A	-0.866	-1.823	0.000E+00
ACVR2B	-0.866	-1.823	7.608E-08
AC090114.3	-0.866	-1.823	2.952E-03
DPY19L2	-0.866	-1.823	8.772E-03
PRKCE	-0.866	-1.823	0.000E+00
SYNJ2BP	-0.866	-1.823	0.000E+00
AC010809.2	-0.866	-1.822	3.868E-05
RPL24	-0.865	-1.822	5.686E-05
ARHGAP24	-0.865	-1.822	5.818E-08
ERMP1	-0.865	-1.821	0.000E+00
RPS20	-0.865	-1.821	7.987E-05
ENPP4	-0.865	-1.821	1.023E-05
RPL23AP53	-0.864	-1.821	1.089E-03
DDX3X	-0.864	-1.820	2.516E-08
NCAPD3	-0.864	-1.819	0.000E+00
HMGB3P4	-0.863	-1.819	1.756E-03
CASD1	-0.863	-1.819	3.763E-06
CD226	-0.863	-1.818	1.282E-07
ARHGAP35	-0.862	-1.818	0.000E+00
NCKAP1	-0.862	-1.817	6.672E-06
HIVEP1	-0.862	-1.817	2.593E-08
ZNF394	-0.862	-1.817	3.234E-09
MIS18BP1	-0.861	-1.817	8.677E-10
PLXDC1	-0.861	-1.816	3.097E-09
CYP27B1	-0.861	-1.816	7.468E-03
ZNF37A	-0.861	-1.816	0.000E+00
MS4A4E	-0.860	-1.815	1.504E-08
EIF2AK3	-0.860	-1.815	2.233E-10
ADAT2	-0.860	-1.815	0.000E+00
ADTRP	-0.859	-1.814	4.428E-04
PIGL	-0.859	-1.814	0.000E+00
PROSER1	-0.859	-1.814	0.000E+00
KIF21A	-0.858	-1.812	5.612E-06
F2R	-0.858	-1.812	2.242E-06
FRYL	-0.857	-1.812	0.000E+00
AL132989.1	-0.857	-1.811	2.947E-05
ZNF571	-0.857	-1.811	4.540E-06
RRM2P3	-0.857	-1.811	4.588E-03
SCOC-AS1	-0.857	-1.811	2.224E-04
CEP70	-0.857	-1.811	6.053E-09
ZBTB41	-0.857	-1.811	2.158E-05
MAP3K7CL	-0.856	-1.810	2.711E-03
CEBPZ	-0.856	-1.810	0.000E+00
AC112128.1	-0.856	-1.810	2.513E-05
GARNL3	-0.855	-1.809	1.239E-05
MFSD2A	-0.855	-1.809	2.692E-06
RPS4X	-0.855	-1.808	3.621E-07
LINC01550	-0.855	-1.808	4.408E-08
LMBRD2	-0.855	-1.808	1.505E-06
SNRPGP10	-0.855	-1.808	8.542E-04
AC090739.1	-0.854	-1.808	3.257E-03
ZNF275	-0.854	-1.807	0.000E+00
ZNF621	-0.854	-1.807	0.000E+00
PPP1R15A	-0.854	-1.807	1.228E-03
AKAP13	-0.853	-1.807	0.000E+00
RREB1	-0.853	-1.806	0.000E+00
RNF138	-0.853	-1.806	9.898E-06
WDR7	-0.853	-1.806	0.000E+00
FRG1HP	-0.853	-1.806	0.000E+00
TRPM7	-0.853	-1.806	0.000E+00
KIAA0895	-0.853	-1.806	4.130E-03
LRP12	-0.852	-1.805	9.629E-05
DIS3L2	-0.852	-1.805	0.000E+00
TTC14	-0.852	-1.805	0.000E+00
KMT2E	-0.851	-1.804	1.879E-10
PBRM1	-0.851	-1.803	0.000E+00
GOLGA8A	-0.851	-1.803	0.000E+00
EGF	-0.851	-1.803	1.512E-03
GMFB	-0.850	-1.802	1.766E-06
RBMXL1	-0.849	-1.801	2.570E-10
ZHX1	-0.849	-1.801	0.000E+00
RSF1	-0.849	-1.801	0.000E+00
C2CD4D-AS1	-0.848	-1.800	1.956E-06
ICA1L	-0.848	-1.800	8.087E-09
HSPA5	-0.848	-1.800	0.000E+00
GPNMB	-0.848	-1.800	9.693E-03
ZNF519	-0.848	-1.799	8.383E-08
MPP7	-0.847	-1.799	0.000E+00
RPS13	-0.847	-1.799	1.349E-06
RUNX3	-0.847	-1.799	1.557E-09
DIP2A	-0.847	-1.799	6.542E-08
KANSL2	-0.847	-1.798	0.000E+00
PDXDC2P-NPIPB14P	-0.847	-1.798	0.000E+00
ID2	-0.847	-1.798	0.000E+00
DPRXP4	-0.846	-1.798	2.333E-03
ZFP36	-0.846	-1.797	5.425E-05
ZNF826P	-0.846	-1.797	1.542E-03
BEX2	-0.845	-1.797	4.786E-06
NAPB	-0.845	-1.796	0.000E+00
CPSF6	-0.845	-1.796	2.825E-10
RBIS	-0.844	-1.795	8.651E-09

UFL1	-0.844	-1.795	1.514E-07
AC008083.3	-0.844	-1.795	1.144E-03
SGK1	-0.844	-1.795	1.279E-03
FRA10AC1	-0.843	-1.794	1.788E-09
NECTIN3	-0.843	-1.794	3.215E-04
AC006130.3	-0.843	-1.794	9.185E-03
AL022322.1	-0.843	-1.794	6.574E-03
XYLB	-0.843	-1.794	3.598E-05
AL441992.1	-0.842	-1.793	1.459E-03
MALINC1	-0.842	-1.793	1.470E-07
ZMYND11	-0.842	-1.792	0.000E+00
AP003084.1	-0.841	-1.792	6.472E-07
CLASP2	-0.841	-1.791	0.000E+00
HDGFL3	-0.841	-1.791	2.860E-05
SCFD1	-0.841	-1.791	0.000E+00
GOLT1B	-0.840	-1.791	1.774E-06
AC098614.2	-0.840	-1.790	1.647E-03
CENPC	-0.840	-1.790	0.000E+00
NEO1	-0.840	-1.789	3.676E-09
ATAD2B	-0.839	-1.789	2.198E-09
CIBAR1	-0.839	-1.789	8.839E-03
TIE1	-0.839	-1.789	6.125E-03
CCDC81	-0.839	-1.789	2.832E-03
ALDH2	-0.839	-1.788	2.997E-06
SOX4	-0.838	-1.788	9.240E-08
ZNF83	-0.838	-1.787	0.000E+00
SAMD4A	-0.838	-1.787	8.857E-07
SETD2	-0.838	-1.787	0.000E+00
LAMA2	-0.838	-1.787	1.178E-03
MAGI3	-0.837	-1.787	1.011E-05
HSP90B1	-0.836	-1.786	0.000E+00
ZBTB11	-0.836	-1.786	6.496E-07
BIRC6	-0.836	-1.785	1.738E-08
ARRDC4	-0.836	-1.785	9.209E-06
COMMD6	-0.836	-1.784	1.372E-04
LINC01572	-0.835	-1.784	6.628E-03
FBXO36	-0.835	-1.784	1.310E-03
JAG1	-0.835	-1.784	1.509E-05
CYLD	-0.835	-1.784	0.000E+00
ANKRA2	-0.835	-1.784	0.000E+00
RPL21	-0.835	-1.783	5.820E-04
FNDC3A	-0.834	-1.783	1.921E-07
ZCRB1	-0.834	-1.783	0.000E+00
AC092111.1	-0.834	-1.783	5.216E-03
TSHZ1	-0.833	-1.782	0.000E+00
KCNH8	-0.833	-1.782	4.490E-03
DNAJC10	-0.833	-1.782	0.000E+00
CASK	-0.833	-1.782	0.000E+00
CDC42BPB	-0.833	-1.781	0.000E+00
AC024293.1	-0.833	-1.781	2.868E-03
MDM1	-0.832	-1.781	0.000E+00
LINC00968	-0.832	-1.781	2.044E-04
ANAPC1	-0.832	-1.780	0.000E+00
ATF7IP2	-0.832	-1.780	5.135E-10
BRCA2	-0.832	-1.780	3.680E-06

EDRF1	-0.832	-1.780	0.000E+00
PLEKHG1	-0.831	-1.779	6.939E-05
SPIN1	-0.831	-1.779	0.000E+00
ZNF441	-0.830	-1.778	0.000E+00
C20orf194	-0.830	-1.778	1.247E-10
COA6-AS1	-0.830	-1.778	1.437E-04
SLC12A2	-0.830	-1.777	1.621E-09
ZNF726	-0.830	-1.777	3.286E-06
LRRC37B	-0.829	-1.777	0.000E+00
RPL5	-0.829	-1.776	3.823E-08
BPTF	-0.829	-1.776	0.000E+00
TOP2A	-0.829	-1.776	6.035E-04
DDX10	-0.829	-1.776	0.000E+00
PHACTR1	-0.828	-1.776	7.277E-03
ZNF550	-0.828	-1.776	0.000E+00
HECTD4	-0.828	-1.775	8.774E-10
ZBTB44	-0.828	-1.775	0.000E+00
PIK3C2A	-0.828	-1.775	3.997E-06
SLC25A30	-0.828	-1.775	0.000E+00
ZNF655	-0.827	-1.774	0.000E+00
ZKSCAN2	-0.827	-1.774	5.575E-08
PRKCQ-AS1	-0.827	-1.774	0.000E+00
ZFAND5	-0.827	-1.774	0.000E+00
TRIM33	-0.826	-1.773	1.164E-08
EIF3M	-0.826	-1.773	0.000E+00
DUSP12	-0.825	-1.772	0.000E+00
ITSN2	-0.824	-1.771	1.347E-10
AGL	-0.824	-1.770	1.987E-07
AL512791.2	-0.824	-1.770	5.887E-03
TRRAP	-0.824	-1.770	0.000E+00
CCDC136	-0.824	-1.770	1.762E-10
MLLT3	-0.824	-1.770	8.231E-08
DYRK2	-0.824	-1.770	0.000E+00
PPWD1	-0.823	-1.769	0.000E+00
MYRF	-0.823	-1.769	7.215E-03
TTN-AS1	-0.822	-1.768	0.000E+00
SMC6	-0.822	-1.768	0.000E+00
ZNF891	-0.822	-1.768	1.111E-07
CENPJ	-0.822	-1.768	2.639E-10
CLINT1	-0.822	-1.767	0.000E+00
HSPA9	-0.821	-1.767	0.000E+00
AZIN1	-0.821	-1.766	1.605E-10
HIPK1	-0.820	-1.766	1.710E-08
WDPCP	-0.820	-1.765	0.000E+00
NID1	-0.819	-1.765	4.690E-06
ENY2	-0.819	-1.764	0.000E+00
TMEM163	-0.819	-1.764	8.130E-03
SLC24A4	-0.818	-1.764	1.889E-07
BEX5	-0.818	-1.763	1.412E-03
TBCK	-0.818	-1.763	0.000E+00
ANGEL2	-0.818	-1.763	0.000E+00
AC090948.4	-0.818	-1.763	8.748E-04
CCDC6	-0.818	-1.762	0.000E+00
NAE1	-0.817	-1.762	0.000E+00
COX7B	-0.817	-1.762	3.245E-06

SPIN3	-0.816	-1.761	0.000E+00
AC012513.3	-0.816	-1.761	4.212E-04
AKR1E2	-0.816	-1.761	2.129E-03
SCML2	-0.816	-1.761	4.890E-04
UTP20	-0.816	-1.760	0.000E+00
LPP	-0.816	-1.760	1.538E-06
PRKX	-0.816	-1.760	0.000E+00
QSER1	-0.815	-1.760	2.516E-10
FXR1	-0.815	-1.759	0.000E+00
AC095057.3	-0.815	-1.759	3.379E-05
POMP	-0.815	-1.759	3.763E-07
PHF14	-0.814	-1.758	0.000E+00
UBE3A	-0.814	-1.758	0.000E+00
SPATA6	-0.813	-1.757	2.006E-10
STAM	-0.813	-1.757	4.840E-10
TEX14	-0.813	-1.757	4.424E-03
CHD7	-0.813	-1.757	8.575E-08
FZD3	-0.812	-1.756	5.206E-06
AL353660.1	-0.812	-1.756	1.587E-05
RPL37A	-0.812	-1.756	4.665E-05
OSBPL3	-0.812	-1.755	0.000E+00
ZNF204P	-0.812	-1.755	1.262E-05
LINC01891	-0.812	-1.755	2.402E-03
FMO5	-0.811	-1.755	1.616E-08
TULP4	-0.811	-1.755	0.000E+00
FAM13A	-0.811	-1.755	3.810E-07
ABLIM1	-0.811	-1.755	0.000E+00
CECR2	-0.811	-1.754	5.725E-03
NUF2	-0.811	-1.754	8.364E-06
ATF4	-0.811	-1.754	5.493E-09
NEGR1	-0.811	-1.754	5.438E-03
ATP11A	-0.810	-1.753	3.359E-06
ATE1	-0.809	-1.752	0.000E+00
TRAJ3	-0.809	-1.752	2.224E-03
IRS2	-0.808	-1.751	1.779E-05
C1S	-0.808	-1.750	2.100E-04
RPS3AP6	-0.807	-1.750	4.559E-03
VRK2	-0.807	-1.750	1.985E-10
ZNF600	-0.807	-1.749	9.974E-07
TUBGCP5	-0.806	-1.748	2.668E-03
UBR4	-0.806	-1.748	0.000E+00
EFCAB13	-0.806	-1.748	1.002E-04
ANKRD17	-0.806	-1.748	0.000E+00
DICER1	-0.806	-1.748	1.126E-05
BRIX1	-0.805	-1.748	1.515E-08
PRDM2	-0.805	-1.748	0.000E+00
CEP128	-0.805	-1.748	8.052E-09
YLPM1	-0.805	-1.748	0.000E+00
SYNJ2	-0.805	-1.748	0.000E+00
ZBTB38	-0.805	-1.747	6.611E-06
EZH2	-0.804	-1.746	0.000E+00
RFX3	-0.804	-1.746	3.245E-08
ICOS	-0.804	-1.746	8.985E-07
AL023806.3	-0.804	-1.746	8.262E-03
INPP4B	-0.804	-1.746	1.236E-07
HSP90AA1	-0.804	-1.746	0.000E+00
TUG1	-0.803	-1.745	0.000E+00
HAS3	-0.803	-1.745	6.598E-05
RUFY2	-0.803	-1.744	0.000E+00
EMILIN2	-0.803	-1.744	0.000E+00
STXBP4	-0.803	-1.744	1.779E-07
HLF	-0.803	-1.744	2.176E-04
DNAH8	-0.802	-1.744	3.783E-03
TOPBP1	-0.802	-1.743	0.000E+00
TNFRSF11A	-0.801	-1.743	8.295E-04
SIAH1	-0.801	-1.743	1.790E-05
RNF169	-0.801	-1.742	0.000E+00
SRSF6	-0.801	-1.742	0.000E+00
FOXK1	-0.800	-1.741	7.852E-10
DNMT3B	-0.800	-1.741	3.375E-04
AC005562.1	-0.800	-1.741	1.275E-03
PUS7L	-0.800	-1.741	1.040E-07
AP003108.3	-0.800	-1.741	6.099E-03
ZSWIM6	-0.800	-1.741	4.862E-10
NUP205	-0.799	-1.740	0.000E+00
KDM6A	-0.799	-1.740	3.212E-09
G3BP2	-0.799	-1.740	9.338E-10
AC025171.3	-0.799	-1.740	8.009E-04
BMPR2	-0.798	-1.739	1.303E-08
AL122035.2	-0.798	-1.739	2.368E-07
RBM17	-0.798	-1.739	0.000E+00
LGALS2	-0.798	-1.739	1.462E-03
ZNF708	-0.798	-1.739	1.224E-06
CWC15	-0.798	-1.739	1.158E-07
RYBP	-0.798	-1.738	1.967E-04
PAPSS2	-0.798	-1.738	6.171E-06
EP300	-0.797	-1.738	6.439E-07
C1orf54	-0.797	-1.738	9.853E-05
DIAPH2	-0.797	-1.738	0.000E+00
ZNF192P1	-0.797	-1.738	5.504E-03
NCBP2AS2	-0.797	-1.738	2.898E-05
LCNL1	-0.797	-1.738	4.588E-03
HSPE1	-0.797	-1.738	6.646E-08
FBXO25	-0.795	-1.736	0.000E+00
PDE4B	-0.795	-1.735	6.207E-05
ZNF548	-0.795	-1.735	0.000E+00
CEP295	-0.795	-1.735	0.000E+00
SPIRE2	-0.795	-1.735	9.320E-03
DLG2	-0.795	-1.735	9.765E-03
C1orf112	-0.794	-1.734	2.618E-08
TNRC6B	-0.794	-1.734	1.276E-08
CEMIP2	-0.794	-1.734	1.968E-05
MPP5	-0.794	-1.734	6.282E-07
HAUS6	-0.794	-1.734	0.000E+00
ASH1L-AS1	-0.794	-1.733	4.070E-05
ARL4C	-0.793	-1.733	2.045E-10
MALT1	-0.793	-1.733	0.000E+00
BICD1	-0.793	-1.733	0.000E+00
INVS	-0.793	-1.733	0.000E+00
PHACTR2	-0.793	-1.733	2.263E-08

NCOA7	-0.793	-1.733	4.637E-09
EBLN3P	-0.793	-1.732	9.046E-10
HMGXB4	-0.793	-1.732	0.000E+00
RPL36AL	-0.793	-1.732	3.206E-05
SUN1	-0.793	-1.732	0.000E+00
GASK1B	-0.792	-1.732	1.249E-06
PRELID2	-0.792	-1.732	3.899E-03
SRSF7	-0.792	-1.732	0.000E+00
RPL27	-0.792	-1.731	3.808E-04
HSPA13	-0.792	-1.731	2.013E-05
STIMATE-MUSTN1	-0.792	-1.731	3.505E-10
PLXND1	-0.792	-1.731	7.490E-09
MEST	-0.791	-1.731	3.890E-05
KAT6A	-0.791	-1.730	1.356E-08
CBWD1	-0.791	-1.730	0.000E+00
TAF3	-0.791	-1.730	0.000E+00
UBR3	-0.791	-1.730	3.617E-08
CD84	-0.791	-1.730	1.881E-09
LINC01684	-0.791	-1.730	7.546E-04
SCRN1	-0.790	-1.730	0.000E+00
CHRM3-AS2	-0.790	-1.730	8.018E-07
OXR1	-0.790	-1.729	0.000E+00
ZNF81	-0.790	-1.729	1.492E-08
DBI	-0.789	-1.728	3.804E-06
NNT	-0.789	-1.728	0.000E+00
AC092645.2	-0.789	-1.728	1.233E-04
CHEK1	-0.788	-1.727	9.126E-04
NEK1	-0.788	-1.727	0.000E+00
NPAS2	-0.788	-1.726	2.872E-03
ZNF92	-0.787	-1.726	3.468E-05
ELF1	-0.787	-1.726	7.076E-08
LYRM4-AS1	-0.787	-1.725	7.404E-04
SLTM	-0.786	-1.725	0.000E+00
CRTC3	-0.786	-1.725	0.000E+00
MTSS2	-0.786	-1.725	7.696E-04
RBBP8	-0.786	-1.724	3.734E-10
BTBD11	-0.786	-1.724	0.000E+00
TXNRD3	-0.786	-1.724	2.922E-04
MGAT4A	-0.785	-1.724	0.000E+00
CRYZL2P	-0.785	-1.724	2.279E-08
SOGA1	-0.785	-1.723	2.939E-07
AC104653.1	-0.785	-1.723	3.739E-03
NBPF9	-0.784	-1.722	2.990E-08
WASHC4	-0.784	-1.722	7.304E-06
OPA1	-0.784	-1.722	0.000E+00
COG5	-0.784	-1.722	2.476E-03
RASGRP1	-0.783	-1.721	0.000E+00
CCDC93	-0.783	-1.721	0.000E+00
MTBP	-0.783	-1.721	6.528E-07
SCAI	-0.783	-1.721	8.910E-07
SNORA5C	-0.783	-1.721	2.990E-03
PLK3	-0.783	-1.721	2.964E-05
UFM1	-0.783	-1.721	0.000E+00
ETF1	-0.783	-1.720	9.771E-10
FRS2	-0.782	-1.719	9.242E-10

NBPF26	-0.781	-1.719	1.574E-05
TCF12	-0.781	-1.718	0.000E+00
TMEM181	-0.781	-1.718	7.354E-10
PJVK	-0.781	-1.718	9.801E-07
USP25	-0.780	-1.718	0.000E+00
DDHD2	-0.780	-1.717	0.000E+00
HNRNPH3	-0.780	-1.717	0.000E+00
PGM3	-0.779	-1.716	0.000E+00
EPC1	-0.779	-1.716	0.000E+00
RCBTB1	-0.779	-1.716	0.000E+00
C1QTNF3	-0.779	-1.715	1.091E-08
ZNF587B	-0.778	-1.715	1.978E-10
HINT1	-0.778	-1.715	9.619E-10
TOMM20	-0.778	-1.715	0.000E+00
PRKD3	-0.777	-1.714	0.000E+00
FBXO34	-0.777	-1.714	0.000E+00
HECA	-0.777	-1.713	3.143E-08
CHROMR	-0.776	-1.712	2.913E-07
MS4A14	-0.775	-1.712	2.731E-04
SPA17	-0.775	-1.712	1.227E-03
RBM11	-0.775	-1.711	1.853E-03
NRAS	-0.775	-1.711	0.000E+00
CEP57	-0.775	-1.711	0.000E+00
SPIRE1	-0.775	-1.711	5.881E-07
CMAHP	-0.775	-1.711	0.000E+00
ATP5MPL	-0.775	-1.711	1.691E-04
ZPR1	-0.774	-1.711	0.000E+00
SGSH	-0.774	-1.710	2.362E-10
DYNC2I1	-0.774	-1.710	0.000E+00
BUB1B	-0.773	-1.709	7.632E-03
UBP1	-0.773	-1.709	0.000E+00
DENND6A	-0.773	-1.709	5.768E-09
EFHC1	-0.773	-1.709	0.000E+00
MAML2	-0.773	-1.709	2.324E-10
HNRNPDL	-0.772	-1.708	0.000E+00
NDUFA4	-0.772	-1.708	1.310E-05
ULK4	-0.772	-1.707	2.696E-09
ITPR2	-0.772	-1.707	1.184E-08
ZNF330	-0.772	-1.707	0.000E+00
ARL4A	-0.771	-1.707	2.822E-05
LMTK2	-0.771	-1.707	9.035E-10
ATP10A	-0.771	-1.707	0.000E+00
SEPTIN7P2	-0.770	-1.706	0.000E+00
ENPP2	-0.770	-1.705	7.519E-05
ZNF483	-0.770	-1.705	5.807E-05
DNMBP	-0.770	-1.705	3.748E-09
ZEB1	-0.770	-1.705	3.453E-07
MED17	-0.769	-1.704	1.010E-06
HDAC8	-0.769	-1.704	0.000E+00
PRKAA1	-0.769	-1.704	1.078E-09
ABCA10	-0.769	-1.704	4.121E-05
TAF4B	-0.769	-1.704	6.034E-08
ELL2	-0.768	-1.703	3.179E-05
FAM199X	-0.768	-1.703	2.267E-06
PRDM4	-0.768	-1.703	0.000E+00

PRKAB2	-0.768	-1.703	0.000E+00
DYNC2LI1	-0.768	-1.703	6.483E-07
ROCK2	-0.768	-1.703	1.530E-10
LINC00299	-0.767	-1.702	9.435E-05
AC007566.1	-0.767	-1.702	1.439E-10
MED10	-0.767	-1.702	0.000E+00
CGRRF1	-0.767	-1.702	1.092E-08
DCAF16	-0.767	-1.702	3.003E-08
MFSD6	-0.767	-1.702	0.000E+00
HERC1	-0.767	-1.701	6.080E-09
ZNF277	-0.767	-1.701	0.000E+00
RPL6	-0.767	-1.701	7.642E-06
NUBPL	-0.766	-1.701	2.201E-09
SOCS4	-0.766	-1.701	2.203E-06
RAB13	-0.766	-1.700	5.557E-03
SMAD1	-0.766	-1.700	1.745E-04
ZNF195	-0.765	-1.700	0.000E+00
CPEB4	-0.764	-1.698	5.332E-04
ZZZ3	-0.764	-1.698	0.000E+00
AL353743.1	-0.764	-1.698	1.492E-09
MTOR	-0.764	-1.698	0.000E+00
BEX4	-0.763	-1.697	2.580E-09
CTDSPL2	-0.763	-1.697	2.281E-07
PIK3CA	-0.763	-1.697	1.865E-07
RPL39	-0.763	-1.697	9.017E-04
TFG	-0.763	-1.697	0.000E+00
RIC3	-0.763	-1.697	1.121E-07
MTRF1L	-0.763	-1.697	9.205E-10
CD38	-0.763	-1.697	3.831E-06
DUS4L-BCAP29	-0.763	-1.696	1.427E-04
PARPBP	-0.762	-1.696	1.390E-04
GLG1	-0.762	-1.696	0.000E+00
ANXA1	-0.762	-1.696	4.067E-09
B3GLCT	-0.762	-1.696	0.000E+00
ERCC6	-0.762	-1.696	0.000E+00
RASA2	-0.762	-1.696	9.053E-07
ITPRID2	-0.761	-1.695	1.016E-06
SUPT3H	-0.761	-1.695	3.161E-07
IFT80	-0.761	-1.695	6.554E-10
PTK7	-0.761	-1.695	2.939E-05
SYNGAP1	-0.761	-1.695	1.181E-04
TTC37	-0.761	-1.694	3.159E-10
IL12RB2	-0.760	-1.693	8.008E-04
TMX3	-0.759	-1.692	1.428E-09
KDM5A	-0.758	-1.692	0.000E+00
ARL5A	-0.758	-1.691	6.488E-06
LINC00989	-0.757	-1.690	5.331E-03
ATG10	-0.757	-1.690	8.324E-07
LEMD3	-0.756	-1.689	6.756E-09
MED23	-0.756	-1.689	5.872E-08
PMS1	-0.756	-1.689	6.872E-10
PRMT9	-0.756	-1.688	0.000E+00
CENPU	-0.755	-1.688	1.722E-05
SSX2IP	-0.755	-1.688	5.027E-09
ZNF595	-0.755	-1.688	2.041E-05
MED13	-0.754	-1.686	1.304E-09
ASAP2	-0.754	-1.686	1.009E-05
LINC02648	-0.754	-1.686	1.250E-04
STAT4	-0.754	-1.686	0.000E+00
EPHB2	-0.754	-1.686	2.424E-03
DROSHA	-0.753	-1.685	0.000E+00
TMC3-AS1	-0.753	-1.685	7.620E-05
TTC12	-0.752	-1.684	0.000E+00
AC091978.1	-0.752	-1.684	1.826E-03
SORCS3	-0.752	-1.684	6.668E-03
GUCY1A1	-0.752	-1.684	1.088E-04
SNED1	-0.751	-1.683	4.441E-05
LINC01934	-0.751	-1.683	1.150E-07
CTH	-0.751	-1.683	3.092E-03
RPGRIP1L	-0.751	-1.682	4.136E-05
CNST	-0.750	-1.682	0.000E+00
ZNF433	-0.749	-1.681	1.587E-03
ENTPD1-AS1	-0.749	-1.681	0.000E+00
YPEL5	-0.749	-1.680	7.045E-07
USP7	-0.749	-1.680	0.000E+00
LARP1	-0.749	-1.680	0.000E+00
PPFIBP1	-0.748	-1.680	1.485E-05
KANTR	-0.748	-1.680	1.081E-10
BCL11B	-0.748	-1.679	0.000E+00
LPIN1	-0.747	-1.679	0.000E+00
RPL30	-0.747	-1.679	2.417E-04
ZUP1	-0.747	-1.678	1.164E-09
RNF6	-0.747	-1.678	9.497E-10
DDIT3	-0.747	-1.678	5.888E-04
L3MBTL3	-0.747	-1.678	1.364E-09
ZMYM2	-0.746	-1.677	0.000E+00
EIF1	-0.746	-1.677	9.164E-09
HUS1	-0.746	-1.677	0.000E+00
SLC22A5	-0.746	-1.677	6.321E-07
ARID2	-0.746	-1.677	3.076E-06
NTRK1	-0.745	-1.676	6.928E-03
ZBTB5	-0.745	-1.676	1.238E-09
SMC3	-0.745	-1.676	0.000E+00
ABL1	-0.744	-1.675	0.000E+00
SLC38A2	-0.744	-1.675	5.431E-07
SLC25A13	-0.744	-1.674	0.000E+00
ZC3H12B	-0.744	-1.674	9.242E-05
NEK3	-0.743	-1.674	0.000E+00
USP30	-0.743	-1.674	2.570E-10
RAB12	-0.743	-1.674	1.241E-07
HBS1L	-0.743	-1.674	0.000E+00
STRN	-0.743	-1.674	1.114E-08
CDK5RAP2	-0.743	-1.674	0.000E+00
ELP1	-0.743	-1.673	0.000E+00
PTCD3	-0.743	-1.673	0.000E+00
MMD	-0.743	-1.673	2.156E-05
AC022400.4	-0.743	-1.673	6.642E-05
BLOC1S6	-0.742	-1.673	4.233E-10
CCDC7	-0.741	-1.672	2.748E-06
MASTL	-0.741	-1.672	2.092E-05

JMJD1C	-0.741	-1.671	2.948E-04
TSC1	-0.741	-1.671	0.000E+00
MARCHF6	-0.740	-1.671	0.000E+00
AC015871.6	-0.740	-1.670	5.750E-05
AC105345.1	-0.740	-1.670	2.334E-04
CEP97	-0.740	-1.670	1.761E-06
EIF2A	-0.739	-1.670	0.000E+00
SERINC5	-0.739	-1.669	1.086E-06
TAF5L	-0.739	-1.669	0.000E+00
ANAPC1P2	-0.738	-1.668	3.587E-08
CDCA7	-0.738	-1.668	8.540E-06
HTATSF1	-0.738	-1.668	0.000E+00
C18orf25	-0.737	-1.667	1.755E-10
ZFAND4	-0.737	-1.667	1.457E-07
NEU3	-0.737	-1.667	0.000E+00
USP48	-0.737	-1.667	0.000E+00
TIAM1	-0.737	-1.666	0.000E+00
NAV1	-0.737	-1.666	5.222E-06
AEBP2	-0.735	-1.665	9.963E-08
SNHG3	-0.735	-1.665	0.000E+00
DCHS1	-0.735	-1.665	1.891E-05
AC060766.1	-0.735	-1.664	3.842E-04
RNF7	-0.735	-1.664	0.000E+00
DDX42	-0.734	-1.664	0.000E+00
SEC24A	-0.734	-1.664	1.569E-06
MTPAP	-0.734	-1.663	0.000E+00
COBLL1	-0.734	-1.663	1.257E-03
MAPKAPK5	-0.734	-1.663	0.000E+00
SUPV3L1	-0.734	-1.663	1.978E-06
PPP1R16B	-0.734	-1.663	2.584E-06
AL356512.1	-0.733	-1.663	1.416E-03
MED1	-0.733	-1.662	0.000E+00
DPY19L4	-0.732	-1.661	3.867E-07
ZNF846	-0.732	-1.661	4.305E-09
TNS3	-0.732	-1.661	4.708E-08
L3MBTL4	-0.731	-1.660	1.477E-05
SOCS7	-0.731	-1.660	1.024E-04
UBA5	-0.731	-1.660	0.000E+00
DCAF1	-0.731	-1.660	0.000E+00
FUT10	-0.731	-1.659	5.519E-04
ZMYM6	-0.730	-1.659	0.000E+00
SPATA7	-0.730	-1.659	1.053E-06
ENPP3	-0.729	-1.658	6.105E-03
C5orf24	-0.729	-1.657	3.118E-05
AC138207.9	-0.729	-1.657	4.839E-03
DIS3	-0.729	-1.657	1.913E-08
LSM8	-0.728	-1.657	0.000E+00
FRMD4B	-0.728	-1.657	5.737E-08
FAM214A	-0.728	-1.657	7.966E-08
SDC4	-0.728	-1.656	4.500E-03
RANBP2	-0.728	-1.656	2.987E-08
GPD1L	-0.728	-1.656	0.000E+00
RNF168	-0.727	-1.656	1.138E-07
GAN	-0.726	-1.654	2.277E-10
SHCBP1	-0.726	-1.654	1.486E-04

ZNF10	-0.726	-1.654	5.478E-08
TAF1	-0.726	-1.653	0.000E+00
BX571818.1	-0.725	-1.653	5.747E-03
AL122035.1	-0.725	-1.653	4.275E-04
NSD3	-0.725	-1.653	0.000E+00
SNX25	-0.725	-1.653	6.319E-10
ADCY7	-0.724	-1.652	6.578E-10
RAB30	-0.724	-1.652	2.008E-04
ZMYM4	-0.723	-1.651	0.000E+00
TECTA	-0.723	-1.651	1.529E-03
ZNF699	-0.723	-1.651	1.475E-06
CYP2R1	-0.723	-1.651	0.000E+00
RBM26	-0.723	-1.651	0.000E+00
AC015911.3	-0.723	-1.650	4.871E-05
MTRR	-0.722	-1.650	0.000E+00
MYO1A	-0.722	-1.650	5.050E-04
FLJ40194	-0.722	-1.649	4.448E-04
RTCA-AS1	-0.722	-1.649	1.867E-05
RNF115	-0.721	-1.649	0.000E+00
SGMS2	-0.721	-1.649	1.143E-03
UBE3C	-0.720	-1.647	0.000E+00
ATP9A	-0.720	-1.647	2.469E-03
CYB5D1	-0.719	-1.646	8.920E-03
AC005332.7	-0.719	-1.646	2.320E-03
POLR1A	-0.719	-1.646	3.313E-10
DNHD1	-0.719	-1.646	3.792E-09
AC090589.3	-0.719	-1.646	3.204E-05
MCM9	-0.719	-1.645	0.000E+00
BCAS2	-0.719	-1.645	6.121E-09
CLIP1	-0.718	-1.645	1.426E-08
TSPYL4	-0.718	-1.645	0.000E+00
LINC01146	-0.718	-1.645	3.366E-04
MPRIP	-0.718	-1.644	0.000E+00
EIF4G3	-0.717	-1.644	0.000E+00
SMARCC1	-0.717	-1.644	0.000E+00
TNKS2	-0.717	-1.644	3.424E-07
SNHG21	-0.717	-1.644	3.361E-03
IMPACT	-0.716	-1.643	0.000E+00
BCCIP	-0.716	-1.643	0.000E+00
TMTC3	-0.716	-1.643	1.164E-06
CD28	-0.716	-1.643	8.376E-06
M1AP	-0.716	-1.643	5.680E-03
DZIP3	-0.716	-1.643	4.229E-08
SEC63	-0.716	-1.642	0.000E+00
LINC00667	-0.715	-1.641	0.000E+00
SAT1	-0.715	-1.641	1.068E-05
ZNF697	-0.715	-1.641	2.214E-04
RB1CC1	-0.715	-1.641	1.529E-05
PER3	-0.715	-1.641	3.739E-06
ARRDC3-AS1	-0.715	-1.641	8.481E-05
ACER2	-0.714	-1.641	1.116E-03
SMARCAD1	-0.714	-1.641	1.882E-08
SUGT1P4-STRA6LP	-0.714	-1.640	9.051E-05
TRAM2	-0.714	-1.640	2.171E-09
ZNF91	-0.713	-1.640	3.343E-10

CCDC112	-0.713	-1.639	1.146E-08
TCEAL4	-0.713	-1.639	2.906E-06
RPS6	-0.713	-1.639	1.642E-05
KLF7	-0.713	-1.639	1.827E-07
PTPN9	-0.712	-1.639	0.000E+00
SLC25A33	-0.712	-1.638	1.501E-06
BBS2	-0.712	-1.638	0.000E+00
AKAP7	-0.711	-1.637	0.000E+00
GOLM1	-0.711	-1.637	9.987E-06
PNPT1	-0.711	-1.637	0.000E+00
AMER1	-0.711	-1.637	1.386E-09
ZNF664	-0.711	-1.637	0.000E+00
CFAP36	-0.711	-1.637	3.583E-08
BBS9	-0.711	-1.636	0.000E+00
R3HDM1	-0.710	-1.636	0.000E+00
CCDC149	-0.710	-1.636	1.296E-07
LDLRAD4	-0.710	-1.636	0.000E+00
PLCG1-AS1	-0.710	-1.635	1.863E-05
CBFA2T2	-0.710	-1.635	0.000E+00
UHRF1BP1L	-0.709	-1.635	1.775E-06
PRKCH	-0.709	-1.635	0.000E+00
ZRANB3	-0.708	-1.633	4.072E-06
MATR3	-0.708	-1.633	1.668E-10
ARID1B	-0.708	-1.633	0.000E+00
IRF2BP2	-0.708	-1.633	0.000E+00
SLC9B1	-0.708	-1.633	1.391E-03
LUC7L2	-0.707	-1.633	4.624E-08
POLR1F	-0.707	-1.633	3.657E-05
ING3	-0.706	-1.632	7.613E-09
PAXIP1	-0.706	-1.631	0.000E+00
SRFBP1	-0.706	-1.631	1.346E-08
ZFP36L2	-0.706	-1.631	6.229E-08
RTKN2	-0.706	-1.631	1.136E-03
GLRX2	-0.706	-1.631	2.005E-07
MTMR8	-0.705	-1.631	9.271E-05
XPOT	-0.705	-1.630	0.000E+00
ITGB1	-0.705	-1.630	1.244E-08
VSIG4	-0.705	-1.630	5.903E-03
CEP57L1	-0.705	-1.630	3.324E-07
ATP10D	-0.705	-1.630	9.136E-08
ZSWIM5	-0.705	-1.630	3.739E-06
TTC24	-0.705	-1.630	6.698E-04
GSE1	-0.704	-1.629	4.832E-10
ZNF8-ERVK3-1	-0.704	-1.629	2.139E-03
AGK	-0.704	-1.629	9.009E-09
GTF3C3	-0.704	-1.629	0.000E+00
RRN3P2	-0.703	-1.628	1.913E-03
AL356235.1	-0.703	-1.628	5.136E-03
AC024560.4	-0.703	-1.628	9.582E-03
STX17	-0.703	-1.628	0.000E+00
NUFIP2	-0.703	-1.628	8.986E-07
FAM153A	-0.703	-1.628	6.235E-03
ZBED3	-0.703	-1.628	2.530E-07
GFRA2	-0.703	-1.627	2.036E-03
AC105429.1	-0.702	-1.627	9.433E-03

IRAK3	-0.702	-1.627	3.089E-07
EXOSC9	-0.702	-1.627	0.000E+00
HMGB1	-0.702	-1.627	0.000E+00
C5orf63	-0.702	-1.627	5.749E-06
NSD2	-0.702	-1.626	0.000E+00
PKD2	-0.701	-1.626	2.275E-07
RPL22	-0.701	-1.626	1.105E-07
PACC1	-0.701	-1.625	0.000E+00
FAM83D	-0.701	-1.625	3.752E-03
RALGAPB	-0.701	-1.625	0.000E+00
ZNF529	-0.700	-1.625	3.050E-07
ORMDL1	-0.700	-1.625	0.000E+00
ATP5MG	-0.700	-1.625	3.842E-05
ZNF26	-0.700	-1.625	0.000E+00
NBPF11	-0.700	-1.625	0.000E+00
HEATR1	-0.700	-1.624	5.857E-09
DNAJC21	-0.700	-1.624	0.000E+00
TMEM30A-DT	-0.699	-1.624	1.210E-03
NOL4L	-0.699	-1.624	0.000E+00
AFDN	-0.699	-1.624	4.260E-03
MAP1A	-0.699	-1.624	5.368E-05
BBS7	-0.699	-1.623	3.263E-09
LONP2	-0.699	-1.623	0.000E+00
MTATP6P1	-0.699	-1.623	1.343E-05
ATP11C	-0.699	-1.623	1.144E-08
SIK2	-0.699	-1.623	0.000E+00
SHLD2	-0.699	-1.623	0.000E+00
WNT2B	-0.698	-1.623	3.315E-03
PRPF40A	-0.698	-1.622	1.283E-09
TDG	-0.698	-1.622	0.000E+00
LAMC1	-0.698	-1.622	4.208E-04
TSTD2	-0.698	-1.622	0.000E+00
MAPK9	-0.698	-1.622	0.000E+00
STX2	-0.698	-1.622	1.307E-06
SERPINB8	-0.697	-1.621	0.000E+00
HNRNPH1	-0.697	-1.621	3.709E-09
RIN2	-0.697	-1.621	1.256E-06
EXT1	-0.696	-1.621	1.221E-09
AHSA2P	-0.696	-1.621	0.000E+00
WDR11	-0.696	-1.620	1.537E-10
NCL	-0.696	-1.620	0.000E+00
TXNDC16	-0.696	-1.620	1.043E-07
PTPN2	-0.696	-1.620	0.000E+00
RTL5	-0.695	-1.619	5.540E-05
TUT4	-0.695	-1.619	8.272E-08
PHF7	-0.695	-1.619	1.901E-10
XPNPEP1	-0.695	-1.619	0.000E+00
PDE8A	-0.695	-1.618	0.000E+00
LINC01284	-0.694	-1.618	6.427E-03
PSMD6	-0.694	-1.617	0.000E+00
KDM5D	-0.693	-1.617	7.808E-04
PPP4R3B-DT	-0.693	-1.617	3.023E-03
INPP4A	-0.693	-1.617	0.000E+00
ANAPC4	-0.693	-1.616	0.000E+00
FOXN3	-0.693	-1.616	0.000E+00

LMNA	-0.693	-1.616	1.458E-03
ARID4B	-0.693	-1.616	8.506E-06
AC067852.3	-0.692	-1.616	8.649E-03
AC090616.6	-0.692	-1.615	2.787E-05
ZNF792	-0.691	-1.615	1.639E-09
SAT2	-0.691	-1.615	6.587E-07
RBMS2	-0.691	-1.615	4.409E-05
TSPYL5	-0.691	-1.614	2.777E-03
PCNX2	-0.691	-1.614	0.000E+00
KNL1	-0.690	-1.613	1.056E-03
PCNX4	-0.689	-1.612	1.226E-07
AP002985.1	-0.689	-1.612	7.739E-04
AC113935.1	-0.689	-1.612	4.423E-03
DYNLT1	-0.689	-1.612	1.341E-05
NAP1L1	-0.689	-1.612	0.000E+00
MID2	-0.689	-1.612	8.670E-07
PDGFC	-0.688	-1.611	5.207E-05
JUNB	-0.688	-1.611	2.395E-03
DCBLD1	-0.688	-1.611	3.897E-06
SEC24B	-0.688	-1.611	1.074E-08
ZNF680	-0.687	-1.610	5.497E-05
KLHL20	-0.687	-1.610	3.709E-09
NUP160	-0.687	-1.610	4.197E-07
PHF6	-0.687	-1.610	1.255E-06
TBC1D9	-0.687	-1.610	8.447E-06
HNRNPA1L2	-0.687	-1.610	1.256E-07
ZNF138	-0.687	-1.610	1.343E-07
H4-16	-0.687	-1.609	6.094E-04
CCDC91	-0.686	-1.609	1.300E-09
INO80B	-0.686	-1.609	6.304E-04
PKD1P6	-0.685	-1.608	6.430E-04
TSC22D2	-0.685	-1.608	1.707E-07
ZMYM5	-0.685	-1.608	2.398E-10
PET100	-0.685	-1.608	5.539E-05
SGSM1	-0.685	-1.607	3.186E-04
REPS1	-0.685	-1.607	0.000E+00
AL606491.1	-0.685	-1.607	8.588E-03
ADHFE1	-0.684	-1.607	2.086E-09
MOB4	-0.684	-1.607	2.075E-05
ALG13	-0.684	-1.606	0.000E+00
LINC01772	-0.684	-1.606	4.685E-04
ZNF90	-0.683	-1.606	8.241E-04
RAD51AP1	-0.682	-1.605	1.293E-03
ZBTB26	-0.682	-1.605	1.350E-07
UTP4	-0.682	-1.604	8.470E-08
ZNF525	-0.682	-1.604	1.915E-08
IMMP1L	-0.681	-1.604	3.926E-05
USP49	-0.681	-1.604	1.024E-05
ARIH1	-0.681	-1.604	1.353E-10
TAF1D	-0.681	-1.603	2.871E-07
RPS6KC1	-0.681	-1.603	0.000E+00
XRN1	-0.681	-1.603	1.603E-04
HSPB11	-0.681	-1.603	0.000E+00
PXYLP1	-0.681	-1.603	5.701E-09
ZBTB25	-0.680	-1.603	0.000E+00

ZNF831	-0.680	-1.603	0.000E+00
RP9P	-0.680	-1.603	4.315E-03
MS4A6A	-0.680	-1.602	1.641E-06
DYM	-0.680	-1.602	0.000E+00
OPHN1	-0.680	-1.602	7.410E-04
AC010327.8	-0.680	-1.602	6.792E-04
GRIP1	-0.680	-1.602	9.366E-03
C4orf33	-0.680	-1.602	2.282E-08
OTUD6B-AS1	-0.679	-1.601	8.891E-06
PSMA1	-0.678	-1.600	3.435E-07
USP13	-0.678	-1.600	3.762E-10
IRGQ	-0.678	-1.600	8.157E-07
RB1	-0.678	-1.600	3.479E-07
FAM24B	-0.678	-1.600	3.390E-06
THBS4-AS1	-0.678	-1.600	4.490E-06
AC073957.3	-0.678	-1.600	8.285E-04
C2CD2	-0.678	-1.600	0.000E+00
ZNF682	-0.677	-1.599	1.721E-06
ZFP91	-0.677	-1.599	0.000E+00
PCCA	-0.677	-1.599	0.000E+00
THAP12	-0.677	-1.599	0.000E+00
Z84485.1	-0.677	-1.599	3.497E-04
FAM228B	-0.677	-1.598	1.930E-09
AC127502.2	-0.676	-1.598	1.918E-03
CFAP97	-0.676	-1.597	2.253E-06
MICOS10	-0.675	-1.597	6.344E-08
CAPN2	-0.675	-1.597	0.000E+00
USPL1	-0.675	-1.596	0.000E+00
AC055811.4	-0.675	-1.596	2.170E-03
TRIM52-AS1	-0.674	-1.596	5.466E-04
ATP6AP1L	-0.674	-1.596	9.712E-03
FAM136A	-0.674	-1.595	0.000E+00
POLA1	-0.674	-1.595	0.000E+00
CDC42BPA	-0.674	-1.595	2.396E-03
ZC3HAV1	-0.674	-1.595	0.000E+00
KLF5	-0.673	-1.595	8.869E-03
Z98884.2	-0.673	-1.594	4.539E-03
TRAF4	-0.672	-1.594	6.650E-05
AL450384.2	-0.672	-1.594	1.250E-04
PRORSD1P	-0.672	-1.593	2.707E-06
EPG5	-0.672	-1.593	0.000E+00
SLC4A8	-0.672	-1.593	1.875E-07
ING2	-0.672	-1.593	3.856E-06
TBC1D5	-0.671	-1.592	0.000E+00
HSD17B7P2	-0.670	-1.591	3.188E-03
SMIM13	-0.670	-1.591	2.798E-08
ATL2	-0.670	-1.591	1.048E-08
GTF2I	-0.670	-1.591	3.123E-05
KIF13B	-0.669	-1.590	0.000E+00
NUDT6	-0.669	-1.590	1.836E-04
WDR70	-0.669	-1.590	0.000E+00
ZFC3H1	-0.669	-1.590	3.162E-07
ZZEF1	-0.669	-1.590	8.150E-10
DISP1	-0.668	-1.589	6.037E-08
ZNF235	-0.668	-1.589	8.835E-09

PTGER4	-0.668	-1.589	1.209E-06
AC004846.2	-0.668	-1.589	1.709E-03
DNAJC15	-0.667	-1.588	1.075E-06
LRRC40	-0.667	-1.588	4.009E-04
MAGEE1	-0.667	-1.588	1.946E-09
NBPF10	-0.667	-1.588	5.184E-05
NADK2	-0.667	-1.588	6.257E-09
SRSF5	-0.667	-1.588	0.000E+00
MAGOH	-0.667	-1.588	2.487E-06
CNOT2	-0.666	-1.587	0.000E+00
GNL2	-0.666	-1.587	0.000E+00
FIRRE	-0.666	-1.587	3.911E-04
MPP6	-0.666	-1.587	3.385E-05
LONRF1	-0.666	-1.586	6.299E-03
KLRF1	-0.666	-1.586	2.649E-03
TCN2	-0.666	-1.586	1.680E-03
COX14	-0.665	-1.586	1.469E-03
ADAMTS17	-0.665	-1.586	1.332E-04
OBSCN	-0.665	-1.586	3.734E-05
KIF3A	-0.665	-1.585	1.596E-05
PHTF2	-0.665	-1.585	3.266E-05
PTPN22	-0.665	-1.585	1.610E-05
AC022400.5	-0.665	-1.585	4.208E-03
PRDM15	-0.664	-1.585	0.000E+00
MBTD1	-0.664	-1.585	3.813E-09
ZC4H2	-0.664	-1.585	5.074E-08
BARD1	-0.664	-1.584	7.755E-06
SYNCRIP	-0.663	-1.584	0.000E+00
DNAH1	-0.663	-1.584	2.999E-08
PIAS2	-0.663	-1.583	0.000E+00
RAB3GAP2	-0.663	-1.583	2.563E-10
GP5	-0.663	-1.583	3.081E-04
RPL38	-0.662	-1.583	3.044E-04
KLF13	-0.662	-1.583	3.455E-10
CPVL	-0.661	-1.582	4.381E-06
STK32B	-0.661	-1.581	9.251E-03
GNL3	-0.661	-1.581	0.000E+00
MS4A7	-0.661	-1.581	5.525E-04
PLXNB2	-0.660	-1.580	1.299E-04
CDON	-0.660	-1.580	1.119E-03
EVC2	-0.660	-1.580	6.889E-03
SOS1	-0.660	-1.580	4.736E-07
KIDINS220	-0.660	-1.580	6.173E-07
MATN1-AS1	-0.660	-1.580	4.344E-04
PDIA3P1	-0.659	-1.579	1.587E-09
ARHGEF7	-0.659	-1.579	0.000E+00
AC104564.3	-0.659	-1.579	7.052E-03
MTIF2	-0.659	-1.579	0.000E+00
WDFY2	-0.659	-1.579	1.515E-10
TAB2	-0.659	-1.579	0.000E+00
CD2AP	-0.659	-1.579	3.158E-05
SLC22A15	-0.659	-1.579	2.338E-05
TRAF3IP1	-0.658	-1.578	0.000E+00
CDK12	-0.658	-1.578	6.218E-09
POLR3G	-0.658	-1.578	2.814E-03
SUSD1	-0.658	-1.578	0.000E+00
POLR3F	-0.658	-1.578	1.440E-07
ARMCX5	-0.658	-1.577	0.000E+00
AC004890.2	-0.658	-1.577	3.382E-05
KIF16B	-0.658	-1.577	7.366E-05
GPAM	-0.657	-1.577	7.678E-08
CENPP	-0.657	-1.577	2.898E-04
MBTPS1	-0.657	-1.577	0.000E+00
TTC17	-0.657	-1.577	0.000E+00
TOMM5	-0.657	-1.577	2.912E-05
MINDY2	-0.657	-1.576	8.939E-04
ZFP28	-0.657	-1.576	2.987E-07
SLC18A2	-0.656	-1.576	4.251E-03
TTLL5	-0.656	-1.576	0.000E+00
SUGP2	-0.656	-1.576	0.000E+00
QSOX2	-0.656	-1.576	1.305E-07
PANK1	-0.656	-1.576	8.051E-03
AC026412.1	-0.656	-1.575	1.254E-08
MBTPS2	-0.656	-1.575	8.241E-06
POLR2K	-0.656	-1.575	2.972E-06
USP34	-0.654	-1.574	5.385E-10
NF1	-0.654	-1.574	1.422E-08
ANKRD13C	-0.654	-1.574	2.641E-10
TENT5A	-0.654	-1.574	9.081E-06
PTPN11	-0.654	-1.573	5.372E-10
SH3BP4	-0.654	-1.573	2.017E-04
PTPDC1	-0.653	-1.573	3.657E-06
CCNT2-AS1	-0.653	-1.573	5.088E-08
CD1D	-0.653	-1.573	2.689E-07
KLHDC1	-0.653	-1.572	2.913E-04
PASK	-0.652	-1.572	2.393E-05
PPP3CC	-0.652	-1.571	0.000E+00
SLC10A7	-0.652	-1.571	4.165E-09
HNRNPA2B1	-0.651	-1.571	0.000E+00
ATP5MD	-0.651	-1.570	5.422E-04
ABCC3	-0.651	-1.570	8.420E-04
APPL1	-0.651	-1.570	4.593E-08
PSMA6	-0.651	-1.570	2.391E-09
DNA2	-0.650	-1.570	6.643E-05
AC005224.3	-0.650	-1.570	7.487E-03
PITRM1	-0.650	-1.569	0.000E+00
TRAPPC11	-0.650	-1.569	0.000E+00
VEZT	-0.650	-1.569	0.000E+00
ZNF654	-0.650	-1.569	7.199E-05
EMC1	-0.650	-1.569	0.000E+00
HOOK3	-0.649	-1.569	5.066E-06
WDFY1	-0.649	-1.568	5.748E-07
NAPG	-0.649	-1.568	1.111E-05
PPP4R3A	-0.649	-1.568	0.000E+00
MED12L	-0.649	-1.568	8.610E-03
INTS10	-0.649	-1.568	0.000E+00
TBC1D19	-0.649	-1.568	2.897E-05
SUPT7L	-0.648	-1.567	0.000E+00
MAGOHB	-0.648	-1.567	9.099E-08
RAD18	-0.648	-1.567	0.000E+00

ZNF367	-0.648	-1.567	3.133E-05
TRIAP1	-0.648	-1.567	1.835E-07
RPS8	-0.648	-1.567	4.456E-04
CDNF	-0.648	-1.567	2.006E-03
PTPRS	-0.648	-1.567	4.424E-03
RIPK2	-0.647	-1.566	4.916E-06
TMEM267	-0.647	-1.566	1.302E-05
SERPINF1	-0.647	-1.566	5.305E-04
SNRPD2	-0.647	-1.566	3.854E-03
ATP1B3	-0.647	-1.566	0.000E+00
CUL1	-0.647	-1.566	0.000E+00
ZBED5	-0.647	-1.565	0.000E+00
EIF5	-0.646	-1.565	2.038E-06
PTPMT1	-0.646	-1.565	1.269E-06
ZFP62	-0.646	-1.565	0.000E+00
FBXL19-AS1	-0.646	-1.565	3.692E-03
CLEC2B	-0.646	-1.565	9.293E-04
FAM126A	-0.646	-1.564	5.939E-06
GFPT1	-0.646	-1.564	5.211E-06
ERCC6L2	-0.646	-1.564	3.107E-10
STARD8	-0.646	-1.564	5.466E-04
RPS4XP16	-0.645	-1.564	2.594E-03
CLCN6	-0.645	-1.564	3.657E-08
ENOX2	-0.645	-1.564	0.000E+00
EML4	-0.645	-1.564	3.395E-08
CAVIN2	-0.645	-1.564	5.486E-03
DHX35	-0.645	-1.563	0.000E+00
MT-ATP6	-0.644	-1.563	1.152E-04
HEATR6	-0.644	-1.562	0.000E+00
FYTTD1	-0.644	-1.562	1.298E-10
IQCH-AS1	-0.644	-1.562	1.898E-08
NIT2	-0.643	-1.562	0.000E+00
CD36	-0.643	-1.561	1.127E-05
RGS5	-0.643	-1.561	3.478E-05
MRPL33	-0.642	-1.561	5.753E-05
AC011912.1	-0.642	-1.561	8.097E-03
SLC41A1	-0.642	-1.561	0.000E+00
HSPA14	-0.642	-1.561	6.486E-10
SLC30A1	-0.642	-1.560	1.373E-03
INO80	-0.641	-1.560	0.000E+00
SPDL1	-0.641	-1.560	6.088E-06
AC022893.1	-0.641	-1.560	2.289E-03
PDS5A	-0.641	-1.560	0.000E+00
ZFX	-0.641	-1.560	1.904E-09
NOL8	-0.641	-1.559	0.000E+00
PIK3IP1	-0.641	-1.559	1.931E-04
RPL41	-0.641	-1.559	2.802E-03
C10orf88B	-0.641	-1.559	7.950E-04
NIPBL-DT	-0.640	-1.558	8.260E-08
TSHZ2	-0.640	-1.558	2.224E-03
USP37	-0.639	-1.557	9.234E-08
ST8SIA6	-0.639	-1.557	1.515E-03
PCNT	-0.639	-1.557	0.000E+00
PAXBP1-AS1	-0.639	-1.557	3.553E-06
NR2C2	-0.639	-1.557	5.090E-06
ATP8B4	-0.639	-1.557	5.982E-07
C8orf37	-0.639	-1.557	4.371E-05
HOMER2	-0.639	-1.557	4.746E-06
ERBIN	-0.639	-1.557	6.834E-07
IARS2	-0.638	-1.557	0.000E+00
AFG1L	-0.638	-1.556	3.432E-06
VAMP4	-0.638	-1.556	5.724E-09
DYRK1A	-0.638	-1.556	0.000E+00
INCENP	-0.637	-1.556	1.079E-07
TARS3	-0.637	-1.556	0.000E+00
TRIP11	-0.637	-1.555	8.284E-07
AL592148.3	-0.637	-1.555	6.542E-05
RPL14	-0.637	-1.555	1.238E-04
ARNILA	-0.637	-1.555	5.324E-03
ZRANB2	-0.637	-1.555	2.971E-06
ZNF644	-0.636	-1.554	1.337E-05
AC015813.6	-0.636	-1.554	4.431E-06
MIDEAS	-0.636	-1.554	7.868E-06
ZNF778	-0.636	-1.554	0.000E+00
LANCL1	-0.635	-1.553	1.820E-08
GRAMD2B	-0.635	-1.553	2.077E-06
SPATA13	-0.635	-1.553	0.000E+00
UBN2	-0.635	-1.552	5.270E-06
ZNF439	-0.635	-1.552	4.024E-08
MAP3K21	-0.634	-1.552	1.612E-03
MYLIP	-0.634	-1.552	1.812E-05
TSC22D1	-0.634	-1.551	6.478E-10
ARID4A	-0.633	-1.551	2.136E-05
SH3PXD2A	-0.633	-1.551	1.507E-05
C2CD5	-0.633	-1.551	0.000E+00
SNHG6	-0.633	-1.550	1.227E-03
RFC3	-0.633	-1.550	4.326E-07
TAF2	-0.632	-1.550	9.416E-07
PON2	-0.632	-1.550	6.241E-08
TANGO6	-0.632	-1.550	0.000E+00
LRRC8C	-0.632	-1.549	4.232E-06
NXPE3	-0.632	-1.549	7.448E-10
EXOC6B	-0.631	-1.548	1.074E-10
VANGL1	-0.630	-1.548	1.100E-04
DCUN1D4	-0.630	-1.548	4.209E-08
MAD2L1	-0.630	-1.547	3.566E-04
SLC35D1	-0.630	-1.547	4.974E-08
ZNF713	-0.630	-1.547	9.010E-04
CHCHD7	-0.629	-1.547	0.000E+00
DOCK2	-0.629	-1.547	0.000E+00
SNHG17	-0.629	-1.547	5.677E-07
EPM2A	-0.629	-1.546	3.010E-08
TTF2	-0.629	-1.546	3.302E-08
PSMD7	-0.629	-1.546	7.525E-10
NFATC2	-0.629	-1.546	0.000E+00
ATP2C1	-0.628	-1.546	0.000E+00
DDX46	-0.628	-1.545	0.000E+00
MZT1	-0.628	-1.545	8.411E-05
RUSC2	-0.628	-1.545	7.306E-04
RANBP9	-0.628	-1.545	0.000E+00

NIN	-0.628	-1.545	1.781E-08
ZNF354B	-0.627	-1.545	6.900E-08
PPIL3	-0.627	-1.545	0.000E+00
NOM1	-0.627	-1.545	0.000E+00
SPIDR	-0.627	-1.545	0.000E+00
COIL	-0.627	-1.545	2.273E-10
TAF11	-0.627	-1.544	0.000E+00
NDC80	-0.627	-1.544	1.047E-03
TASOR	-0.626	-1.544	5.830E-10
ESR1	-0.626	-1.544	1.822E-03
AC026979.4	-0.626	-1.544	6.479E-03
SLC5A6	-0.626	-1.543	5.592E-07
ATP11B	-0.626	-1.543	3.093E-06
SEC31B	-0.626	-1.543	0.000E+00
TRIT1	-0.625	-1.543	2.745E-10
CTNNB1	-0.625	-1.543	0.000E+00
TIMM17A	-0.625	-1.542	2.493E-08
TP53BP1	-0.625	-1.542	0.000E+00
PI4K2A	-0.625	-1.542	6.672E-05
PSPC1	-0.625	-1.542	0.000E+00
POU2F1	-0.625	-1.542	8.553E-06
TRAF6	-0.624	-1.542	3.944E-07
MIR4292	-0.624	-1.541	7.417E-03
PCGF3	-0.624	-1.541	1.363E-06
STX8	-0.624	-1.541	1.311E-03
MTX2	-0.624	-1.541	2.638E-06
BIRC3	-0.623	-1.541	9.583E-06
PDXDC1	-0.623	-1.541	1.440E-06
RPL32	-0.623	-1.540	4.098E-03
LRATD2	-0.623	-1.540	1.963E-06
CMC2	-0.623	-1.540	1.151E-06
ZNF136	-0.623	-1.540	5.267E-06
FAM151B	-0.623	-1.540	1.121E-03
CSNK1G3	-0.622	-1.539	2.485E-06
COQ8A	-0.622	-1.539	4.105E-04
TTL	-0.622	-1.539	1.922E-10
JAK2	-0.622	-1.539	1.474E-04
RNASEH2B	-0.621	-1.538	2.364E-10
AGTPBP1	-0.621	-1.538	7.197E-09
BMPR1A	-0.620	-1.537	1.725E-05
MRRF	-0.620	-1.537	0.000E+00
SLC44A1	-0.620	-1.537	0.000E+00
ERN1	-0.620	-1.537	6.692E-06
DDI2	-0.620	-1.537	0.000E+00
HPGD	-0.620	-1.537	8.654E-04
RAPGEF2	-0.620	-1.537	4.787E-04
CRBN	-0.620	-1.537	0.000E+00
RTRAF	-0.620	-1.536	2.191E-07
MRAS	-0.620	-1.536	6.411E-05
AL691432.2	-0.619	-1.536	8.261E-06
FBXL17	-0.619	-1.536	6.117E-07
AC008124.1	-0.619	-1.536	0.000E+00
MCCC1	-0.619	-1.535	0.000E+00
CBWD2	-0.619	-1.535	0.000E+00
CFDP1	-0.618	-1.535	2.624E-04
RBL2	-0.618	-1.535	1.126E-05
SNX13	-0.618	-1.535	6.383E-05
DSTYK	-0.618	-1.534	0.000E+00
TPR	-0.618	-1.534	0.000E+00
ZNF649	-0.618	-1.534	5.977E-06
CCDC191	-0.617	-1.534	2.922E-07
RDH10	-0.617	-1.534	7.557E-07
AC073130.3	-0.617	-1.534	7.800E-04
RUNX1	-0.617	-1.534	0.000E+00
NORAD	-0.617	-1.534	0.000E+00
NUP210	-0.617	-1.534	0.000E+00
FANCI	-0.617	-1.534	0.000E+00
TMPRSS5	-0.617	-1.534	2.166E-03
MYCBPAP	-0.617	-1.534	6.021E-03
AC004967.2	-0.617	-1.533	8.442E-03
ACTR5	-0.616	-1.533	0.000E+00
SPRED2	-0.616	-1.533	1.443E-03
EPS8	-0.616	-1.533	4.812E-04
OFD1	-0.616	-1.533	0.000E+00
SLC7A6	-0.616	-1.533	7.640E-10
PHC1	-0.616	-1.533	2.168E-08
VASH1	-0.616	-1.533	5.463E-03
MT-ND4L	-0.615	-1.532	2.704E-07
EIF5B	-0.615	-1.532	9.903E-06
ATP2B4	-0.615	-1.532	7.009E-06
ATG14	-0.615	-1.532	1.428E-08
NIPBL	-0.615	-1.532	5.344E-06
NFYA	-0.615	-1.531	1.448E-10
DCUN1D5	-0.615	-1.531	5.343E-09
DCAF17	-0.615	-1.531	9.843E-06
ST3GAL5	-0.615	-1.531	0.000E+00
CBX3P2	-0.614	-1.531	2.413E-04
BDH2	-0.614	-1.531	1.444E-05
MNAT1	-0.614	-1.531	2.947E-04
TMEM242	-0.614	-1.531	0.000E+00
ARL6	-0.614	-1.530	2.800E-04
ANAPC10	-0.614	-1.530	4.306E-04
TMEM62	-0.614	-1.530	5.453E-10
ARMC2	-0.613	-1.530	1.087E-07
SRGAP2C	-0.613	-1.530	2.615E-04
ATP5F1C	-0.613	-1.529	1.642E-07
COMMD10	-0.613	-1.529	1.886E-08
ANTXRLP1	-0.613	-1.529	7.417E-04
MB21D2	-0.613	-1.529	1.455E-03
ATF7IP	-0.613	-1.529	8.661E-07
RNF38	-0.613	-1.529	1.777E-07
CLK1	-0.612	-1.529	3.752E-04
DDX52	-0.612	-1.529	2.256E-05
PNO1	-0.612	-1.529	1.639E-06
METAP2	-0.612	-1.528	0.000E+00
SP140L	-0.612	-1.528	0.000E+00
ZNF814	-0.612	-1.528	1.485E-06
ME3	-0.611	-1.528	1.639E-03
RDX	-0.611	-1.527	3.549E-07
HIPK3	-0.610	-1.526	1.516E-03

DNAJC9	-0.610	-1.526	2.359E-08
ZNF25	-0.610	-1.526	1.256E-10
MIOS	-0.609	-1.525	0.000E+00
CDADC1	-0.609	-1.525	8.838E-10
U91328.1	-0.609	-1.525	5.069E-05
RPS6KB1	-0.608	-1.525	1.094E-07
GON4L	-0.608	-1.525	0.000E+00
C12orf45	-0.608	-1.525	7.786E-06
PCM1	-0.608	-1.524	6.291E-10
CCDC134	-0.608	-1.524	3.442E-06
NFIA	-0.608	-1.524	5.040E-05
KIF5C	-0.608	-1.524	8.160E-04
RBM19	-0.608	-1.524	0.000E+00
BTN2A3P	-0.608	-1.524	6.085E-06
HADH	-0.608	-1.524	0.000E+00
NUDT15	-0.607	-1.523	5.742E-06
POM121	-0.607	-1.523	5.063E-09
TEX2	-0.607	-1.523	0.000E+00
RARS2	-0.607	-1.523	0.000E+00
UPRT	-0.607	-1.523	0.000E+00
HNRNPA3	-0.606	-1.522	0.000E+00
CLIC4	-0.606	-1.522	1.206E-04
RPL10A	-0.606	-1.522	1.389E-03
ALG9	-0.606	-1.522	3.824E-05
MLH3	-0.605	-1.521	1.807E-03
NDUFA9	-0.605	-1.521	5.629E-07
SFT2D2	-0.605	-1.521	6.110E-07
ZBTB14	-0.604	-1.520	0.000E+00
BCAT1	-0.604	-1.520	4.330E-03
BPNT2	-0.604	-1.520	3.577E-08
MYO1D	-0.604	-1.520	6.687E-06
TCF7L2	-0.604	-1.520	5.706E-05
RCL1	-0.604	-1.520	2.702E-06
LINC00598	-0.604	-1.520	3.369E-03
DDX12P	-0.604	-1.520	2.474E-03
RBMX	-0.603	-1.519	0.000E+00
SH2B3	-0.603	-1.519	3.586E-09
SNHG26	-0.603	-1.519	9.226E-04
CCDC43	-0.603	-1.518	7.230E-09
GRAMD1B	-0.602	-1.518	6.303E-07
ZNF77	-0.602	-1.518	5.439E-06
FAM168A	-0.602	-1.518	0.000E+00
VAV3	-0.602	-1.518	7.121E-09
TGFB3	-0.602	-1.517	8.054E-07
NPC1	-0.602	-1.517	2.545E-07
P2RY2	-0.601	-1.517	3.287E-03
SRSF3	-0.601	-1.517	2.050E-10
ALS2	-0.601	-1.517	1.636E-07
BRWD3	-0.600	-1.516	2.206E-03
MON2	-0.600	-1.516	3.283E-06
HCFC2	-0.600	-1.516	2.419E-07
RNFT1	-0.600	-1.516	2.452E-08
C3orf38	-0.600	-1.516	5.090E-06
PPFIA1	-0.600	-1.515	8.529E-04
EHBP1	-0.599	-1.515	0.000E+00

TMEM182	-0.599	-1.515	3.023E-04
BEND3	-0.599	-1.514	1.095E-03
CGGBP1	-0.599	-1.514	1.853E-09
BRAF	-0.599	-1.514	7.352E-07
POLG2	-0.599	-1.514	0.000E+00
RAB27B	-0.598	-1.514	2.615E-03
PDCL3	-0.598	-1.514	1.883E-07
ACER3	-0.598	-1.513	5.835E-07
P2RX7	-0.598	-1.513	5.022E-05
TPTE2P5	-0.598	-1.513	5.728E-03
PDHX	-0.598	-1.513	0.000E+00
ZNF569	-0.597	-1.513	1.001E-06
IFT81	-0.597	-1.513	3.369E-03
ERCC8	-0.597	-1.513	1.419E-10
FAM172A	-0.597	-1.513	8.599E-08
TMEM86A	-0.597	-1.512	1.451E-04
GSTM2	-0.597	-1.512	1.930E-03
TXLNG	-0.597	-1.512	2.243E-09
TYW3	-0.597	-1.512	8.408E-10
PIWIL4	-0.596	-1.512	1.413E-06
EPB41L2	-0.596	-1.512	2.460E-07
SUGT1P3	-0.596	-1.512	7.903E-03
ARHGEF9	-0.596	-1.511	0.000E+00
NAA30	-0.596	-1.511	1.473E-05
SNX19	-0.596	-1.511	4.653E-08
MAPKAPK5-AS1	-0.595	-1.511	7.355E-04
AC107871.1	-0.595	-1.511	4.185E-03
EOGT	-0.595	-1.511	8.555E-06
LINC02175	-0.595	-1.511	3.295E-04
SLFN11	-0.595	-1.511	6.700E-06
NEK6	-0.595	-1.510	1.097E-04
ETV3	-0.595	-1.510	6.222E-04
LINC00944	-0.595	-1.510	6.426E-03
RADX	-0.594	-1.510	6.591E-05
RNMT	-0.594	-1.510	2.574E-07
TLK1	-0.594	-1.510	4.137E-09
PIN4	-0.594	-1.509	7.872E-03
BCL2	-0.593	-1.509	1.607E-08
MT-CO3	-0.593	-1.509	1.140E-04
MAP3K9	-0.593	-1.509	9.129E-06
MRPL22	-0.593	-1.509	3.336E-05
SPNS1	-0.593	-1.508	5.845E-05
PRKAG2	-0.593	-1.508	6.360E-09
KIF2A	-0.593	-1.508	0.000E+00
AC090948.3	-0.592	-1.507	4.295E-03
AP4E1	-0.592	-1.507	1.319E-09
DNAJB9	-0.590	-1.505	5.210E-04
MTDH	-0.590	-1.505	0.000E+00
OTULINL	-0.589	-1.504	0.000E+00
CYTH3	-0.588	-1.504	3.827E-06
ZNF528	-0.588	-1.503	5.247E-07
DDX19B	-0.587	-1.503	4.253E-10
SIRT1	-0.587	-1.502	1.064E-05
AHCYL2	-0.587	-1.502	1.828E-08
TRAPPC13	-0.587	-1.502	2.759E-09

AC022400.6	-0.587	-1.502	8.697E-05
HNRNPCP2	-0.587	-1.502	1.873E-04
GUF1	-0.586	-1.502	1.386E-06
EXOSC7	-0.586	-1.501	3.093E-10
MTRF1	-0.585	-1.500	0.000E+00
SAFB	0.585	1.500	0.000E+00
PLP2	0.585	1.500	9.392E-07
LPP-AS2	0.586	1.501	5.582E-03
PLD6	0.586	1.501	6.374E-05
TXNRD1	0.586	1.501	1.318E-06
IFT46	0.586	1.502	3.079E-07
VPS33A	0.586	1.502	3.688E-09
PBXIP1	0.587	1.502	1.353E-07
HCLS1	0.587	1.502	0.000E+00
GYS1	0.587	1.502	0.000E+00
MBP	0.587	1.502	2.132E-06
ZNF768	0.587	1.502	1.055E-05
TBC1D22A	0.587	1.502	0.000E+00
ZNF416	0.587	1.503	4.078E-05
DVL1	0.588	1.503	1.204E-04
MFSD4B	0.588	1.503	2.675E-04
NOP53	0.588	1.503	3.727E-04
FAM193B	0.588	1.503	0.000E+00
FADS3	0.588	1.503	7.258E-05
MHENCR	0.588	1.503	3.507E-04
AL390066.1	0.589	1.504	1.223E-03
HOXA1	0.589	1.504	7.646E-03
SRRM2-AS1	0.589	1.504	2.183E-03
CCNF	0.589	1.504	2.075E-05
ARHGAP30	0.589	1.504	3.093E-10
OSCAR	0.590	1.505	1.780E-03
ZNF316	0.590	1.505	3.838E-09
FAM220A	0.590	1.505	1.885E-07
MICALL2	0.590	1.506	8.711E-04
GDE1	0.591	1.506	2.237E-10
COTL1	0.591	1.506	7.569E-07
ZGPAT	0.591	1.507	1.117E-06
RNF126	0.592	1.507	3.192E-03
TAB1	0.592	1.507	0.000E+00
ZMAT5	0.592	1.507	2.737E-04
NAA60	0.592	1.507	0.000E+00
AC040977.1	0.592	1.507	1.835E-04
PCBP4	0.592	1.508	6.102E-07
GPR55	0.592	1.508	1.783E-03
AMPD3	0.592	1.508	0.000E+00
NEDD9	0.593	1.508	7.664E-06
TIGD6	0.593	1.508	1.380E-04
FLT3LG	0.593	1.508	4.846E-07
SP110	0.593	1.509	1.706E-05
NT5E	0.594	1.509	8.639E-03
CCDC107	0.594	1.509	1.643E-05
TMCO6	0.594	1.509	0.000E+00
PRELID1	0.594	1.510	3.693E-06
HS1BP3	0.594	1.510	8.577E-08
METTL26	0.594	1.510	1.311E-03
MARK4	0.594	1.510	6.188E-08
FKBP1A	0.594	1.510	0.000E+00
ATG2A	0.595	1.510	2.104E-03
RABL6	0.595	1.510	1.016E-07
CHIC2	0.595	1.510	5.103E-07
DENND1C	0.595	1.510	0.000E+00
N4BP1	0.595	1.510	1.291E-05
TRMT2A	0.595	1.511	1.176E-07
CCDC9	0.595	1.511	1.742E-05
MSMO1	0.595	1.511	6.557E-05
HOXB4	0.596	1.511	5.744E-04
GNRH1	0.596	1.511	3.045E-06
LAG3	0.596	1.511	3.478E-04
CCDC126	0.596	1.512	1.677E-03
RNF13	0.597	1.512	1.117E-04
TRMT61A	0.597	1.512	1.946E-04
GET4	0.597	1.512	5.629E-05
HDGF	0.597	1.512	8.539E-10
VPS28	0.597	1.513	2.241E-04
GNMT	0.597	1.513	5.375E-04
NCKAP5L	0.598	1.513	2.706E-05
TPD52L2	0.598	1.513	7.759E-09
STAT5B	0.598	1.514	1.875E-06
ROGDI	0.599	1.514	1.431E-03
BX293535.1	0.599	1.514	8.732E-03
AC245100.6	0.599	1.514	7.346E-03
UBLCP1	0.599	1.515	2.381E-07
CHCHD5	0.599	1.515	2.340E-04
FLCN	0.599	1.515	1.009E-07
CD59	0.600	1.515	5.919E-05
RABEPK	0.600	1.516	1.259E-07
BST1	0.600	1.516	1.695E-04
FAM131B	0.600	1.516	5.997E-03
ACAA1	0.601	1.517	6.102E-09
NAPA	0.601	1.517	1.998E-08
ZNF485	0.601	1.517	1.585E-04
GPR162	0.602	1.517	1.310E-03
CFL1	0.602	1.517	0.000E+00
LYRM1	0.602	1.517	4.868E-06
CEP131	0.602	1.518	1.763E-05
SLC29A2	0.602	1.518	4.334E-06
PITPNA	0.602	1.518	2.831E-09
VASH1-AS1	0.603	1.519	6.343E-04
DOK2	0.603	1.519	3.447E-06
CCDC28A	0.603	1.519	3.617E-08
AP5B1	0.604	1.520	2.022E-04
ZBTB46	0.604	1.520	2.591E-03
CLEC1A	0.604	1.520	1.991E-03
CISD2	0.605	1.521	1.329E-05
EGLN2	0.605	1.521	2.256E-10
ALDOC	0.605	1.521	2.388E-06
XPNPEP2	0.605	1.521	2.753E-03
TRAV13-1	0.606	1.522	4.199E-05
SAMD4B	0.606	1.522	7.000E-10
CD3G	0.606	1.522	3.418E-07

UBOX5	0.606	1.522	4.387E-10
SPATA33	0.606	1.522	7.409E-06
ABHD6	0.607	1.523	3.165E-08
CEP89	0.607	1.523	4.037E-05
PRC1-AS1	0.608	1.524	5.851E-04
MED20	0.608	1.524	1.341E-05
TXNDC15	0.609	1.525	0.000E+00
HSPBP1	0.609	1.525	2.261E-05
STARD3	0.609	1.525	0.000E+00
WIPI1	0.609	1.526	1.709E-07
KCNH3	0.610	1.526	2.267E-03
DGKZP1	0.610	1.526	1.972E-04
FAM110A	0.610	1.526	2.004E-04
PRMT6	0.610	1.526	3.963E-07
PXN-AS1	0.610	1.526	1.860E-04
IGFBP4	0.611	1.527	5.003E-03
MAP3K10	0.611	1.527	2.251E-05
INIP	0.611	1.528	7.541E-07
CSTB	0.611	1.528	6.431E-06
MT1F	0.611	1.528	6.982E-03
VPS51	0.612	1.528	2.227E-06
PLEKHJ1	0.612	1.528	2.022E-06
ZNF443	0.612	1.528	8.514E-04
TRAV29DV5	0.612	1.528	1.205E-03
TMEM127	0.612	1.528	0.000E+00
WDR25	0.612	1.529	5.330E-07
ATP1A3	0.613	1.529	4.244E-03
LINC00910	0.613	1.529	5.094E-05
TRAV38-2DV8	0.613	1.529	8.475E-03
AC011477.1	0.614	1.530	8.254E-09
TMEM107	0.614	1.530	3.344E-06
RNF14	0.616	1.533	2.632E-08
AC145207.5	0.617	1.533	2.544E-04
PLEKHB1	0.617	1.534	5.566E-06
MGAT2	0.617	1.534	2.441E-10
RNF44	0.617	1.534	2.743E-10
PNPO	0.617	1.534	5.481E-05
FERMT3	0.618	1.534	5.572E-08
ALDH16A1	0.618	1.534	2.541E-06
SH3GL1	0.618	1.535	1.011E-08
AC007485.2	0.618	1.535	1.167E-03
MIIP	0.618	1.535	3.033E-05
LINC01144	0.618	1.535	9.368E-03
SMUG1	0.618	1.535	2.454E-05
GIMAP7	0.618	1.535	6.372E-03
BTLA	0.618	1.535	2.074E-03
LGALS9	0.619	1.535	6.238E-05
TRAV3	0.619	1.536	8.335E-03
CHPF	0.619	1.536	3.593E-04
DACH1	0.619	1.536	7.356E-03
CDCA8	0.619	1.536	2.053E-03
CIC	0.620	1.536	3.490E-06
TMEM129	0.620	1.537	2.282E-09
LINC01003	0.620	1.537	1.358E-05
ATP6V0E2-AS1	0.620	1.537	5.195E-03

BRCA1	0.621	1.538	7.425E-04
GABARAPL2	0.621	1.538	1.692E-08
TMEM259	0.621	1.538	1.901E-05
RIPOR1	0.621	1.538	1.503E-07
IGHMBP2	0.621	1.538	1.956E-04
AC068338.2	0.622	1.539	3.215E-03
FAR2	0.622	1.539	8.862E-04
CCR7	0.622	1.539	3.611E-03
CPT2	0.623	1.540	6.293E-08
DOCK5	0.623	1.540	6.497E-03
CD40LG	0.623	1.540	8.185E-06
FAM171A1	0.624	1.541	9.269E-04
ATG4D	0.624	1.541	5.687E-05
ATF7-NPFF	0.624	1.541	2.655E-04
NOSIP	0.624	1.541	8.812E-04
AC016876.1	0.624	1.541	4.873E-03
POLM	0.624	1.541	0.000E+00
TRIM73	0.625	1.542	5.770E-03
ZBTB18	0.625	1.542	3.506E-06
ZNF500	0.625	1.542	4.397E-10
C11orf54	0.625	1.542	7.091E-09
RAF1	0.625	1.542	1.080E-06
CDC123	0.625	1.542	3.316E-10
ISYNA1	0.626	1.543	6.569E-05
TRIB3	0.626	1.544	8.537E-05
FAM122A	0.627	1.544	0.000E+00
MPZL2	0.627	1.544	3.671E-03
AC007406.4	0.627	1.545	5.062E-06
PANK2	0.627	1.545	0.000E+00
TYROBP	0.628	1.545	5.420E-04
EFCAB8	0.628	1.545	1.486E-04
TSPAN13	0.628	1.546	8.248E-04
AC015871.3	0.628	1.546	1.250E-04
CD40	0.628	1.546	1.059E-04
ALKBH1	0.629	1.546	0.000E+00
NBL1	0.629	1.546	4.081E-03
C1orf159	0.629	1.546	2.524E-04
NUDT16L1	0.630	1.547	1.230E-06
SRGN	0.630	1.548	3.290E-03
HIRIP3	0.630	1.548	8.191E-06
NOP10	0.630	1.548	4.531E-04
DNAJC4	0.630	1.548	1.237E-06
FAM222B	0.630	1.548	0.000E+00
TIFA	0.630	1.548	1.722E-05
SCNN1A	0.631	1.549	3.073E-04
PPP4R1	0.631	1.549	4.905E-04
TRIM8	0.631	1.549	6.843E-09
TRBJ2-7	0.632	1.549	3.739E-03
PDE4DIPP6	0.632	1.550	9.061E-03
SMARCD2	0.632	1.550	0.000E+00
GPR108	0.632	1.550	0.000E+00
DALRD3	0.633	1.551	3.078E-08
PYCR2	0.634	1.552	1.269E-09
TMEM175	0.634	1.552	3.042E-08
OGG1	0.635	1.553	0.000E+00

HILPDA	0.635	1.553	3.458E-03
HMGCL	0.635	1.553	8.969E-09
FAM86C1P	0.635	1.553	2.585E-03
ARHGDIA	0.636	1.554	9.710E-07
POLR2E	0.636	1.554	1.090E-07
PSKH1	0.636	1.554	0.000E+00
REPIN1	0.636	1.554	0.000E+00
SMOX	0.637	1.555	7.753E-03
PPP2R1A	0.637	1.555	0.000E+00
SNX8	0.637	1.555	4.751E-08
STX5	0.637	1.555	0.000E+00
OXLD1	0.637	1.555	2.493E-04
AL080317.2	0.637	1.555	1.539E-03
TKT	0.637	1.556	3.533E-07
FAM86EP	0.637	1.556	1.171E-03
VEGFB	0.638	1.556	1.094E-07
CHERP	0.638	1.556	0.000E+00
PRKAR1B	0.638	1.556	4.108E-04
SH3GLB2	0.638	1.556	0.000E+00
MZT2A	0.638	1.557	2.170E-03
FTH1P8	0.639	1.557	3.927E-03
SLC8B1	0.639	1.557	0.000E+00
CACNA1D	0.639	1.557	9.517E-03
EOMES	0.640	1.558	7.055E-04
INTS5	0.640	1.558	2.262E-05
RAB5IF	0.640	1.558	3.875E-06
FBXW9	0.641	1.559	2.627E-04
AC010210.1	0.641	1.559	5.460E-04
ATP8B3	0.641	1.559	5.298E-07
PGAM1	0.641	1.560	0.000E+00
SF3B4	0.641	1.560	0.000E+00
GPR160	0.641	1.560	4.190E-03
ZFPL1	0.642	1.560	0.000E+00
SH3RF3	0.642	1.560	3.745E-04
GGACT	0.642	1.560	2.941E-07
TNFAIP2	0.642	1.560	7.335E-05
NUDT16L2P	0.643	1.561	6.276E-04
RETREG2	0.643	1.561	0.000E+00
ACADS	0.643	1.561	1.587E-05
COP1	0.643	1.561	2.403E-06
DLGAP4	0.643	1.562	0.000E+00
RUBCNL	0.643	1.562	2.241E-04
HPS1	0.643	1.562	7.086E-10
CD2	0.643	1.562	3.835E-09
FAM3A	0.644	1.562	8.806E-08
AL356481.1	0.644	1.562	5.029E-03
NECTIN1	0.644	1.563	1.207E-04
EPS15L1	0.644	1.563	0.000E+00
MEGF6	0.644	1.563	6.503E-06
AC079807.1	0.644	1.563	1.568E-04
RSBN1L	0.645	1.564	2.067E-06
PPIL1	0.646	1.564	4.390E-05
ADPRHL1	0.646	1.564	4.570E-03
BX255925.3	0.646	1.565	6.820E-05
SLAMF6	0.646	1.565	6.446E-05

SCNM1	0.646	1.565	1.008E-07
GTF2IP20	0.646	1.565	2.323E-08
PCBP1	0.646	1.565	0.000E+00
AC004812.2	0.647	1.565	2.126E-05
CDK4	0.647	1.566	3.882E-07
TUBD1	0.647	1.566	2.281E-08
CRELD1	0.647	1.566	1.272E-05
AC092279.1	0.647	1.566	7.975E-05
CDK14	0.647	1.566	1.863E-03
SPATA25	0.647	1.566	9.911E-03
CTBS	0.647	1.566	4.696E-04
TLE5	0.648	1.567	9.613E-09
SLC43A1	0.648	1.567	1.505E-08
NDST1	0.648	1.567	9.574E-05
TRAV26-1	0.649	1.568	1.626E-03
XRCC3	0.649	1.568	1.404E-10
LINC00861	0.649	1.568	5.167E-04
TTLL12	0.649	1.568	2.204E-08
RIN3	0.649	1.568	0.000E+00
CLASRP	0.649	1.568	1.477E-09
ORMDL2	0.649	1.568	2.788E-06
TIMP2	0.649	1.568	4.868E-06
AC012313.1	0.649	1.568	4.003E-08
TMEM71	0.649	1.568	5.809E-06
TEDC1	0.649	1.568	1.860E-03
ZNF764	0.650	1.569	4.186E-10
AATBC	0.650	1.569	4.090E-03
TSG101	0.651	1.570	0.000E+00
PGD	0.651	1.570	2.490E-07
KIF1C-AS1	0.651	1.570	3.285E-03
ZBED5-AS1	0.651	1.571	2.422E-05
SERPINH1	0.652	1.571	1.444E-09
CNPY3	0.652	1.571	4.213E-08
SLC25A19	0.652	1.571	1.269E-05
SEPHS2	0.652	1.571	4.778E-09
CAHM	0.653	1.572	1.865E-03
TNK2	0.653	1.572	3.097E-09
LSM10	0.653	1.573	4.384E-06
NAA10	0.653	1.573	1.871E-07
CCNI	0.654	1.573	0.000E+00
AXIN1	0.654	1.573	0.000E+00
ZGLP1	0.654	1.573	9.774E-03
SIGIRR	0.654	1.574	5.372E-08
POC1A	0.655	1.574	2.934E-04
GEMIN7	0.655	1.575	2.845E-08
RELL1	0.655	1.575	4.253E-04
ST3GAL6	0.656	1.576	9.949E-05
TLR5	0.656	1.576	1.919E-03
RPUSD1	0.656	1.576	5.460E-05
NAT1	0.656	1.576	9.686E-05
AC002091.1	0.656	1.576	1.043E-03
NDUFB10	0.656	1.576	7.487E-06
GAMT	0.656	1.576	1.775E-04
MVK	0.657	1.577	2.411E-07
PIM2	0.657	1.577	5.509E-08

CATIP-AS1	0.657	1.577	2.202E-03
RIPOR2	0.658	1.578	8.251E-08
LZTS2	0.658	1.578	0.000E+00
RPS6KB2	0.658	1.578	0.000E+00
CCNI2	0.659	1.579	7.665E-07
ARRDC1-AS1	0.659	1.579	0.000E+00
ELOB	0.659	1.579	2.065E-03
MPG	0.659	1.579	3.968E-04
AC092295.2	0.659	1.579	7.318E-04
PPP1R3F	0.660	1.580	5.516E-09
LINC00623	0.660	1.580	7.074E-08
AL049795.1	0.660	1.580	2.852E-05
CDKN2B	0.660	1.580	5.251E-03
UBL4A	0.660	1.580	2.523E-07
RAB7A	0.660	1.580	0.000E+00
ADGRE5	0.660	1.580	1.353E-06
BCAT2	0.661	1.581	3.449E-08
HAUS4	0.661	1.581	6.802E-06
IQCE	0.662	1.582	0.000E+00
WASH7P	0.662	1.582	7.483E-04
SLC31A2	0.662	1.582	2.743E-05
FAM219A	0.662	1.582	0.000E+00
TRIR	0.662	1.582	2.942E-06
TAFA2	0.662	1.582	5.371E-04
CARHSP1	0.662	1.582	0.000E+00
RFNG	0.662	1.582	2.551E-06
C14orf93	0.662	1.582	0.000E+00
SH2B1	0.662	1.583	0.000E+00
AC011978.1	0.663	1.583	6.003E-03
TTC30B	0.663	1.583	9.430E-04
AL049840.5	0.663	1.583	0.000E+00
AMPD2	0.663	1.583	4.780E-06
HPS6	0.663	1.583	2.332E-10
PIGB	0.663	1.583	1.106E-05
RPUSD3	0.663	1.584	5.600E-07
YDJC	0.663	1.584	9.395E-04
RTF2	0.664	1.584	2.351E-09
TJP3	0.664	1.584	2.486E-04
ZNF324B	0.664	1.584	3.636E-07
LDLRAP1	0.664	1.584	2.412E-07
GATD3B	0.664	1.584	3.233E-03
UBN1	0.664	1.584	8.146E-05
ZNF232	0.664	1.584	1.927E-06
APOBEC3G	0.664	1.585	3.015E-05
C1orf122	0.665	1.585	2.029E-04
TMCC1	0.665	1.585	2.707E-06
ZNF529-AS1	0.665	1.585	2.175E-09
MARCHF9	0.665	1.586	0.000E+00
TP53	0.665	1.586	2.594E-10
AC002091.2	0.665	1.586	6.315E-04
ZFYVE27	0.666	1.586	0.000E+00
TXNL4B	0.666	1.587	0.000E+00
JMJD4	0.666	1.587	4.463E-08
MLYCD	0.667	1.587	0.000E+00
AC060780.1	0.667	1.587	2.354E-07

CENPB	0.667	1.588	2.634E-08
TFAP4	0.667	1.588	0.000E+00
RAD23A	0.667	1.588	1.073E-09
DGKZ	0.668	1.588	0.000E+00
DARS1-AS1	0.668	1.589	3.766E-06
NT5C	0.668	1.589	1.692E-04
RAB35	0.669	1.590	0.000E+00
TPRG1L	0.669	1.590	2.295E-10
RER1	0.669	1.590	0.000E+00
P2RX1	0.670	1.591	2.351E-05
CHST12	0.670	1.591	1.503E-08
TESK1	0.670	1.591	8.315E-10
IGSF8	0.670	1.591	1.276E-09
RNF123	0.670	1.591	0.000E+00
TMEM186	0.671	1.592	5.983E-05
TLR2	0.671	1.592	1.193E-04
VRK3	0.671	1.593	0.000E+00
HINT2	0.672	1.593	3.470E-03
UNC93B1	0.672	1.594	3.442E-06
DCAKD	0.672	1.594	0.000E+00
GBP1	0.672	1.594	8.206E-03
EHHADH	0.673	1.594	5.178E-03
RUNDC1	0.674	1.595	0.000E+00
NAGK	0.674	1.595	3.207E-07
LINC01841	0.674	1.595	2.970E-03
HOOK2	0.674	1.595	0.000E+00
MFSD5	0.674	1.595	0.000E+00
CAMTA2	0.674	1.596	0.000E+00
AL445524.1	0.675	1.596	1.488E-03
CAPN15	0.676	1.597	8.376E-05
FBXO48	0.676	1.597	1.021E-07
NINJ2-AS1	0.676	1.598	5.599E-07
ZNF428	0.676	1.598	1.823E-04
FLJ37453	0.676	1.598	8.368E-08
ZNF397	0.677	1.599	6.835E-08
BTN3A2	0.677	1.599	8.176E-04
ZNF284	0.677	1.599	3.246E-07
KATNB1	0.678	1.599	0.000E+00
TLR1	0.678	1.600	2.672E-03
AP2A1	0.679	1.601	2.231E-10
HMGA1	0.679	1.601	2.830E-10
WDR24	0.679	1.601	6.058E-08
FAM8A1	0.679	1.602	2.390E-03
FBRSL1	0.679	1.602	9.897E-07
ITGB2-AS1	0.680	1.602	7.355E-07
OSBPL2	0.680	1.602	3.960E-10
UBAP1	0.680	1.602	4.114E-08
SDF2L1	0.680	1.602	4.888E-05
RRP1	0.680	1.602	1.116E-08
UBE2R2	0.680	1.602	1.318E-09
C8orf88	0.680	1.602	6.991E-03
TEN1-CDK3	0.680	1.602	1.150E-05
MANBAL	0.680	1.602	2.318E-10
MRPL46	0.680	1.603	7.689E-07
ABHD11	0.680	1.603	4.004E-07

DNAJC5	0.681	1.603	0.000E+00
HGS	0.681	1.603	0.000E+00
MAX	0.681	1.603	0.000E+00
PROSER3	0.681	1.603	0.000E+00
FAM98C	0.681	1.604	0.000E+00
AC087500.1	0.681	1.604	1.127E-08
TSPAN31	0.681	1.604	2.632E-08
EHBP1L1	0.682	1.604	0.000E+00
ASLP1	0.682	1.604	1.497E-03
AC242988.2	0.682	1.605	4.108E-04
ATP6V0E2	0.683	1.605	2.154E-06
PKN3	0.683	1.606	2.897E-05
ZNF219	0.683	1.606	6.765E-05
TRAC	0.684	1.606	2.967E-05
RELL2	0.684	1.607	1.632E-07
Z82243.1	0.684	1.607	5.065E-03
TPST1	0.685	1.607	7.943E-03
RNF25	0.685	1.607	8.754E-07
CLEC4E	0.685	1.608	2.233E-03
PEX6	0.685	1.608	3.000E-07
GSKIP	0.685	1.608	6.572E-07
RASAL3	0.685	1.608	0.000E+00
AC093726.2	0.686	1.608	3.292E-03
AC016065.1	0.686	1.608	4.190E-04
CR1	0.686	1.609	3.655E-04
IPO5P1	0.687	1.610	2.905E-08
KCP	0.687	1.610	8.997E-04
CCDC194	0.687	1.610	7.831E-03
LCAT	0.687	1.610	3.930E-09
ARPC1B	0.688	1.610	8.768E-06
TSPO	0.688	1.612	1.682E-03
ALG1	0.689	1.612	4.621E-10
MPLKIP	0.689	1.612	2.722E-09
GPSM1	0.690	1.613	2.910E-05
SEPTIN1	0.690	1.614	0.000E+00
PNPLA6	0.690	1.614	0.000E+00
GPAT3	0.690	1.614	4.448E-05
CPLANE2	0.690	1.614	4.280E-05
RNF112	0.691	1.614	1.278E-03
AC010883.2	0.691	1.615	3.170E-03
C1orf115	0.692	1.615	1.218E-04
SLC35F6	0.692	1.616	0.000E+00
Z97192.3	0.692	1.616	3.478E-05
WDR13	0.693	1.617	1.452E-08
RRAGA	0.693	1.617	0.000E+00
TMEM101	0.694	1.617	3.594E-07
PTDSS2	0.694	1.617	1.253E-08
PARD6A	0.694	1.617	2.477E-05
NTHL1	0.694	1.617	5.675E-05
ASTN2	0.694	1.617	5.570E-05
FBRS	0.694	1.618	1.570E-09
PYM1	0.694	1.618	2.832E-09
TRAV5	0.694	1.618	2.115E-03
CRIP1	0.694	1.618	2.291E-03
LMF2	0.695	1.619	1.488E-07
TRAV9-2	0.695	1.619	1.075E-04
MUS81	0.695	1.619	0.000E+00
TRIM25	0.696	1.620	3.787E-05
TYK2	0.697	1.621	0.000E+00
ALDH5A1	0.697	1.621	4.101E-08
SGTA	0.697	1.621	0.000E+00
VDR	0.697	1.621	7.689E-07
LINC01094	0.697	1.622	3.868E-03
RAB30-DT	0.698	1.622	2.144E-05
CPQ	0.698	1.622	1.653E-08
YIF1A	0.698	1.622	1.162E-09
METTL2A	0.698	1.623	8.600E-10
KLC2	0.699	1.623	0.000E+00
ATP1A1-AS1	0.699	1.623	7.095E-05
EVI5L	0.699	1.623	6.557E-10
BIN3	0.699	1.623	0.000E+00
AC010327.6	0.699	1.623	3.610E-10
FAM78A	0.699	1.624	0.000E+00
AL391825.1	0.700	1.624	5.087E-03
DPF2	0.700	1.624	0.000E+00
PRCC	0.700	1.625	0.000E+00
AK1	0.701	1.625	2.743E-05
SPOUT1	0.701	1.625	4.809E-04
AC011603.2	0.701	1.626	2.214E-04
AL008729.1	0.702	1.626	1.086E-04
RNASEH1-AS1	0.702	1.627	3.064E-04
RAB33B-AS1	0.702	1.627	2.568E-07
SLC25A10	0.702	1.627	4.588E-03
GZMM	0.703	1.627	1.460E-03
OSTF1	0.703	1.628	1.232E-10
UNC119	0.703	1.628	0.000E+00
LRCH4	0.704	1.629	0.000E+00
LRRC56	0.704	1.629	2.745E-03
C16orf86	0.705	1.630	1.067E-04
SIRPD	0.705	1.630	1.297E-03
KMT2E-AS1	0.705	1.630	2.071E-04
MAP2K7	0.706	1.631	0.000E+00
CCDC71	0.706	1.631	0.000E+00
TRAFD1	0.706	1.631	3.543E-06
MRTFA	0.706	1.631	0.000E+00
WDR92	0.706	1.632	3.525E-04
ACTR1A	0.707	1.632	0.000E+00
CTDNEP1	0.707	1.632	5.343E-10
LMNB1	0.707	1.633	1.648E-05
FASTK	0.708	1.633	7.699E-07
AMN1	0.708	1.633	3.617E-08
NFYC	0.708	1.634	0.000E+00
SH3BGRL3	0.709	1.634	2.783E-06
AL035071.1	0.709	1.635	3.679E-04
PACS1	0.709	1.635	0.000E+00
LAMTOR4	0.710	1.636	1.634E-04
ZNF552	0.710	1.636	4.611E-08
MSL1	0.711	1.637	1.105E-06
FCMR	0.712	1.638	7.105E-09
PIK3CD	0.712	1.638	0.000E+00

TBX21	0.712	1.638	2.860E-07
AC105020.6	0.712	1.638	7.277E-04
ZNF692	0.712	1.638	3.109E-08
RAB5B	0.712	1.638	0.000E+00
AC241585.1	0.712	1.638	3.857E-03
XRCC1	0.713	1.639	0.000E+00
CD7	0.713	1.639	4.083E-05
YBX1P10	0.713	1.639	2.491E-03
ZNF540	0.713	1.639	6.522E-07
TMEM179B	0.715	1.641	0.000E+00
CAMKK2	0.715	1.642	0.000E+00
ZNF429	0.715	1.642	1.837E-03
GRK2	0.715	1.642	0.000E+00
LAMA5	0.716	1.643	6.754E-03
FANCE	0.716	1.643	2.730E-06
LARGE1	0.716	1.643	1.395E-03
SMIM27	0.716	1.643	7.038E-04
AC010536.1	0.717	1.644	1.572E-03
TRBC1	0.717	1.644	2.368E-06
ALDH1B1	0.717	1.644	1.365E-06
DNAL4	0.718	1.644	1.793E-07
SRM	0.718	1.644	4.539E-10
RNF34	0.718	1.645	0.000E+00
LINC02724	0.718	1.645	1.290E-03
CD274	0.718	1.645	7.786E-03
CRIP2	0.719	1.646	8.920E-03
FAAP20	0.719	1.646	5.807E-06
SLC14A1	0.719	1.646	2.633E-05
CSAD	0.719	1.646	0.000E+00
AC073111.3	0.719	1.646	2.486E-07
SUSD3	0.720	1.647	9.172E-07
HEIH	0.720	1.647	5.237E-08
TMEM223	0.720	1.648	2.437E-06
TPRA1	0.720	1.648	2.908E-09
PSAT1	0.721	1.648	6.634E-05
GPR19	0.721	1.648	3.227E-04
C5orf51	0.722	1.649	5.422E-06
CAP1	0.722	1.649	0.000E+00
REEP6	0.722	1.649	6.180E-05
SPIB	0.722	1.650	3.178E-04
PRSS27	0.722	1.650	4.969E-03
MARCHF3	0.723	1.650	3.189E-05
PLTP	0.723	1.651	3.537E-04
ZFP36L1	0.724	1.652	2.693E-07
CHRNE	0.724	1.652	5.189E-05
AC131009.4	0.724	1.652	5.623E-03
AL021707.3	0.724	1.652	5.747E-04
AC087501.4	0.724	1.652	1.337E-05
XYLT2	0.724	1.652	0.000E+00
FRRS1	0.725	1.652	5.470E-06
AL078621.1	0.725	1.653	1.376E-04
CCDC114	0.725	1.653	8.467E-05
RENBP	0.725	1.653	4.222E-07
ANKRD13B	0.726	1.654	4.782E-06
ADGRE2	0.726	1.654	8.240E-06

SRRD	0.726	1.655	4.235E-10
AL121753.2	0.727	1.655	0.000E+00
WDCP	0.727	1.655	1.883E-08
CBLN3	0.727	1.655	1.395E-03
CLDN15	0.727	1.655	0.000E+00
C1orf74	0.727	1.655	9.064E-06
SNRNP70	0.728	1.656	0.000E+00
GNB1L	0.728	1.656	1.225E-04
HDAC11	0.728	1.656	1.239E-07
CKLF	0.728	1.657	4.956E-04
AL139095.2	0.728	1.657	3.003E-03
ARHGEF40	0.729	1.657	4.794E-05
ADPRS	0.729	1.657	8.351E-10
SLC9A3R2	0.729	1.657	2.223E-04
FAAP100	0.729	1.657	8.624E-10
CRISPLD2	0.729	1.658	2.606E-04
CD200	0.730	1.658	9.556E-04
SLC15A4	0.730	1.658	5.629E-06
MGC16275	0.731	1.659	4.614E-03
IL10RB-DT	0.731	1.660	3.996E-03
ZBTB48	0.732	1.661	0.000E+00
LRSAM1	0.732	1.661	0.000E+00
AC012358.2	0.733	1.662	1.074E-04
OTUD5	0.733	1.662	0.000E+00
TCF3	0.734	1.663	2.202E-10
DBF4B	0.734	1.663	2.780E-09
LRP3	0.734	1.663	1.112E-03
CCR1	0.734	1.663	4.807E-03
FDXR	0.734	1.663	3.707E-07
TAS1R3	0.734	1.664	1.037E-03
TIGAR	0.734	1.664	5.247E-06
TRBC2	0.735	1.664	1.737E-07
TIAF1	0.735	1.664	3.689E-08
ILK	0.735	1.664	0.000E+00
FBXO10	0.735	1.664	3.919E-05
AC073073.2	0.736	1.665	1.106E-03
RASSF3	0.736	1.665	3.197E-07
NBEAL2	0.736	1.665	1.063E-09
PRMT5	0.736	1.666	3.302E-08
ASPSCR1	0.736	1.666	3.717E-09
B3GALT6	0.736	1.666	3.185E-06
BRI3	0.736	1.666	5.493E-08
GPR132	0.737	1.666	3.371E-07
TBL1X	0.737	1.667	1.426E-08
ZNF232-AS1	0.737	1.667	2.385E-03
UQCC3	0.737	1.667	8.582E-04
KCTD13	0.737	1.667	0.000E+00
FAM131A	0.738	1.667	0.000E+00
SNX20	0.738	1.668	0.000E+00
LINC01503	0.738	1.668	7.122E-05
TRIP6	0.739	1.669	8.595E-09
BBS10	0.739	1.669	7.073E-04
IRF2-DT	0.739	1.669	6.221E-10
DHTKD1	0.739	1.669	1.162E-10
SPINDOC	0.740	1.670	0.000E+00

TCL6	0.740	1.670	9.630E-03
RTP4	0.741	1.671	4.771E-04
HTRA2	0.741	1.671	0.000E+00
LINC00963	0.741	1.671	8.435E-10
AC002310.1	0.741	1.672	4.817E-04
TRIM56	0.741	1.672	0.000E+00
ZFR2	0.742	1.672	1.869E-03
NOS3	0.743	1.673	2.873E-04
ZC3H10	0.743	1.673	0.000E+00
STAT6	0.743	1.674	0.000E+00
AC127496.7	0.743	1.674	6.304E-05
RAB11FIP1	0.743	1.674	2.497E-05
DYNLL1	0.744	1.675	2.491E-04
ECE1	0.744	1.675	3.330E-08
OR4D1	0.744	1.675	2.399E-03
ATP6V0B	0.745	1.676	2.793E-07
LBX2-AS1	0.746	1.677	9.489E-04
EEF2KMT	0.746	1.677	2.150E-10
AL161756.3	0.746	1.677	1.118E-03
THAP3	0.746	1.678	7.165E-10
UCP2	0.748	1.679	2.659E-10
MTFMT	0.748	1.679	1.023E-08
TRABD	0.748	1.679	1.026E-05
MSRB2	0.748	1.679	9.404E-05
DHRS7	0.748	1.680	0.000E+00
RBM4B	0.749	1.681	6.809E-07
C2orf42	0.749	1.681	5.412E-10
IFITM1	0.750	1.681	5.900E-05
ISOC2	0.750	1.682	6.297E-06
AL031846.1	0.750	1.682	3.039E-04
TMEM69	0.750	1.682	0.000E+00
LRRC45	0.751	1.683	3.590E-10
OCEL1	0.751	1.683	5.396E-08
PRR12	0.752	1.684	5.225E-07
MMP24OS	0.752	1.685	0.000E+00
NFS1	0.753	1.685	0.000E+00
FES	0.753	1.685	5.603E-07
FLYWCH2	0.753	1.685	3.525E-07
STRN4	0.754	1.687	0.000E+00
PPP1R12C	0.754	1.687	0.000E+00
ARRDC5	0.754	1.687	2.555E-10
C11orf24	0.754	1.687	0.000E+00
OPA3	0.754	1.687	0.000E+00
DHFR2	0.755	1.687	2.877E-05
GGT1	0.755	1.688	9.935E-03
FBXL8	0.755	1.688	3.574E-07
BACE2	0.756	1.689	2.699E-04
CAPZA1	0.756	1.689	4.041E-10
ELOA-AS1	0.756	1.689	1.098E-06
SLC2A1	0.757	1.690	1.322E-07
AL158206.1	0.757	1.690	4.355E-06
H1-10	0.757	1.691	2.892E-05
ERFL	0.758	1.691	2.462E-06
SMPD2	0.758	1.691	0.000E+00
SSNA1	0.758	1.692	6.300E-07
PTOV1-AS1	0.759	1.692	3.103E-03
SLC9A1	0.759	1.692	0.000E+00
GEMIN6	0.759	1.692	1.953E-04
LINC00092	0.759	1.692	1.009E-03
GOLPH3L	0.760	1.694	8.277E-07
KRTCAP2	0.760	1.694	0.000E+00
RRAGD	0.760	1.694	4.324E-06
RBM14	0.760	1.694	0.000E+00
PPP1CA	0.761	1.694	0.000E+00
FAM83G	0.761	1.694	1.682E-08
TAPBPL	0.761	1.694	0.000E+00
TRAV25	0.761	1.695	2.762E-04
SIRT7	0.761	1.695	0.000E+00
FAAP24	0.762	1.696	6.327E-05
TM6SF1	0.763	1.697	6.835E-06
INPP5A	0.763	1.698	0.000E+00
RTL8A	0.764	1.698	6.066E-10
TMEM35B	0.764	1.698	5.305E-03
ARPC1A	0.764	1.698	2.271E-10
PGAM1P8	0.764	1.699	5.356E-03
RPL13P5	0.765	1.699	8.084E-07
POLH	0.765	1.700	1.161E-05
WDR18	0.765	1.700	1.768E-06
LINC01137	0.766	1.701	2.054E-04
C19orf44	0.766	1.701	3.661E-06
FBLN5	0.767	1.701	3.172E-03
FURIN	0.767	1.702	5.951E-08
HIP1	0.767	1.702	3.074E-04
PLEKHO1	0.767	1.702	9.072E-09
MRPL20-AS1	0.768	1.703	0.000E+00
AL031283.1	0.768	1.703	6.214E-05
UBE2J2	0.768	1.703	0.000E+00
LINC02422	0.768	1.703	4.123E-04
PTOV1	0.768	1.703	0.000E+00
AP001273.1	0.768	1.703	2.647E-04
CRYBG2	0.769	1.704	3.333E-04
MYADM	0.769	1.704	1.874E-05
ACOT11	0.769	1.704	1.611E-05
PSD4	0.770	1.705	0.000E+00
MICAL2	0.770	1.705	1.480E-06
PPP1R9B	0.770	1.705	0.000E+00
SELENOO	0.771	1.706	1.611E-08
FMNL1	0.771	1.707	0.000E+00
IL1RAP	0.772	1.707	7.480E-03
YIPF1	0.772	1.707	1.738E-09
FAM229A	0.772	1.707	0.000E+00
AC110285.2	0.772	1.708	3.227E-03
GUCD1	0.772	1.708	0.000E+00
ECSIT	0.772	1.708	9.072E-10
PIM1	0.773	1.708	0.000E+00
YIPF2	0.773	1.709	0.000E+00
TMEM216	0.773	1.709	1.418E-09
MCAT	0.773	1.709	7.622E-09
ZNF414	0.773	1.709	7.389E-06
GDI1	0.773	1.709	0.000E+00

SLC25A22	0.773	1.709	1.604E-09
ZNF282	0.774	1.710	0.000E+00
SYTL1	0.775	1.711	0.000E+00
TUT1	0.775	1.711	0.000E+00
C20orf27	0.775	1.712	1.608E-06
RNF19B	0.775	1.712	2.319E-05
B4GALNT3	0.776	1.713	8.905E-03
CCR2	0.776	1.713	1.367E-03
RNF185	0.777	1.713	0.000E+00
AC074117.1	0.777	1.713	0.000E+00
TSR3	0.777	1.713	3.553E-06
AC079322.1	0.777	1.713	5.869E-03
CEP63	0.777	1.714	3.265E-06
TNFRSF8	0.777	1.714	1.467E-05
MRPL41	0.777	1.714	9.003E-04
CDKN2A	0.777	1.714	5.111E-05
DSC2	0.778	1.714	1.873E-03
TM7SF2	0.778	1.714	6.765E-10
DOLPP1	0.778	1.714	0.000E+00
ARL2	0.778	1.715	1.769E-06
RFXANK	0.778	1.715	3.380E-10
DGLUCY	0.778	1.715	0.000E+00
RTL8C	0.779	1.716	0.000E+00
ABCG1	0.779	1.716	5.499E-07
SCN1B	0.779	1.716	2.256E-03
PLIN3	0.779	1.716	1.025E-08
ARHGAP4	0.780	1.717	9.580E-10
AC102953.2	0.780	1.717	2.512E-06
TNFSF12	0.780	1.717	0.000E+00
AC012467.2	0.780	1.717	1.343E-06
GPR34	0.780	1.717	2.754E-03
CTNNAL1	0.780	1.717	8.459E-04
PIGV	0.781	1.718	0.000E+00
RN7SKP80	0.781	1.718	1.761E-03
PRICKLE1	0.782	1.719	1.295E-03
CTBP1	0.782	1.720	0.000E+00
FRMD8	0.782	1.720	0.000E+00
GPSM2	0.783	1.720	7.229E-06
TAOK2	0.783	1.721	0.000E+00
IL13RA1	0.783	1.721	6.282E-06
HYLS1	0.784	1.721	4.532E-08
ATP5F1D	0.784	1.721	1.273E-04
KIAA1614-AS1	0.784	1.722	7.023E-04
CAVIN1	0.784	1.722	5.651E-03
SPSB3	0.784	1.722	3.841E-07
CFAP251	0.785	1.723	2.840E-04
ASB16-AS1	0.785	1.723	0.000E+00
SUGP1	0.785	1.723	0.000E+00
NUP43	0.785	1.723	1.247E-09
TADA3	0.786	1.724	0.000E+00
NDUFAF3	0.786	1.724	2.568E-09
PIK3R3	0.786	1.724	4.310E-07
AC107375.1	0.786	1.724	3.595E-08
SERF2	0.787	1.725	7.780E-06
REX1BD	0.787	1.725	8.942E-05
FAM86C2P	0.787	1.726	9.747E-05
ZNF230	0.788	1.726	1.443E-05
DYNC2I2	0.788	1.727	8.082E-06
GFUS	0.788	1.727	2.189E-04
VPS72	0.789	1.727	5.950E-08
POLR2A	0.789	1.728	1.469E-09
PNP	0.790	1.729	1.997E-06
AL512625.3	0.790	1.729	6.634E-04
FIS1	0.790	1.729	7.192E-06
CCDC125	0.790	1.729	8.167E-05
MTARC1	0.790	1.729	5.502E-05
RAB37	0.790	1.729	0.000E+00
SMAP2	0.790	1.729	2.568E-07
PSMB10	0.790	1.729	1.361E-08
SNAPC2	0.790	1.730	3.886E-09
ABI3	0.790	1.730	1.281E-09
APOBEC3C	0.791	1.730	4.493E-10
AGTRAP	0.791	1.730	1.661E-09
KYAT1	0.791	1.730	4.376E-05
CCDC28B	0.792	1.731	1.471E-05
GNAS	0.792	1.731	0.000E+00
FBXO2	0.792	1.731	1.415E-04
LINC02846	0.792	1.732	8.232E-05
SULT1A3	0.792	1.732	7.655E-03
SLC25A11	0.792	1.732	0.000E+00
IL17RA	0.792	1.732	7.762E-10
IGIP	0.793	1.732	2.692E-04
DBNDD2	0.793	1.732	1.176E-05
UBE2S	0.793	1.732	9.558E-08
AC002116.2	0.793	1.733	7.178E-04
CCR5AS	0.793	1.733	3.060E-03
CASC3	0.793	1.733	0.000E+00
NSUN5	0.793	1.733	0.000E+00
ZNF486	0.794	1.734	8.266E-06
HRH4	0.795	1.735	2.312E-03
IGFLR1	0.795	1.735	4.262E-05
USP10	0.795	1.735	0.000E+00
METRN	0.795	1.735	1.919E-05
GIPC1	0.796	1.736	1.363E-08
ODF3B	0.796	1.736	5.662E-04
ST20-AS1	0.796	1.736	2.603E-06
CSNK1G2	0.797	1.737	7.809E-10
VLDLR	0.797	1.738	2.918E-03
AC090061.1	0.797	1.738	2.927E-04
RBM47	0.797	1.738	6.695E-05
TREM1	0.797	1.738	3.691E-03
OCM	0.798	1.739	8.064E-05
AL627309.5	0.798	1.739	1.930E-03
TNRC18	0.799	1.740	8.169E-08
LPCAT2	0.799	1.740	1.158E-04
PPP1R35	0.799	1.740	8.785E-06
MAMSTR	0.799	1.740	1.678E-03
ZFYVE21	0.799	1.740	0.000E+00
ACTA2	0.800	1.742	1.817E-03
CSKMT	0.801	1.742	2.754E-05

PRKCD	0.801	1.742	0.000E+00
SLC1A5	0.801	1.742	3.746E-07
RPN1	0.801	1.742	0.000E+00
MIR17HG	0.801	1.743	1.772E-04
POU2AF1	0.801	1.743	4.975E-05
CALHM2	0.802	1.743	1.927E-10
DHRS7B	0.802	1.743	2.131E-08
AL391684.1	0.802	1.744	1.545E-05
DEF6	0.802	1.744	0.000E+00
ATG9B	0.802	1.744	1.544E-07
CD24	0.803	1.745	4.337E-03
AC133552.2	0.803	1.745	6.280E-04
PCBP1-AS1	0.803	1.745	0.000E+00
UBE2D3-AS1	0.803	1.745	2.039E-03
AL137127.1	0.804	1.746	3.029E-04
BNIP3L	0.804	1.746	6.949E-08
AP001453.3	0.805	1.747	2.016E-03
OPTN	0.805	1.747	0.000E+00
LHPP	0.805	1.747	0.000E+00
AC132192.2	0.805	1.747	2.705E-04
CYB5R3	0.805	1.747	0.000E+00
STXBP2	0.805	1.747	0.000E+00
AC138035.1	0.805	1.748	4.598E-05
ABCB9	0.805	1.748	6.058E-08
CLTB	0.805	1.748	7.051E-07
RAD9A	0.806	1.748	0.000E+00
AL008729.2	0.806	1.748	5.182E-04
FAM53A	0.807	1.750	6.698E-03
CCDC137	0.808	1.750	0.000E+00
TMEM229B	0.808	1.751	2.813E-10
AC113143.2	0.808	1.751	5.362E-03
C9orf64	0.808	1.751	3.103E-04
HHLA3	0.809	1.752	3.478E-05
ASTE1	0.809	1.752	0.000E+00
P2RX5	0.809	1.752	1.179E-04
CNN2P9	0.809	1.752	5.118E-04
BHLHE40-AS1	0.810	1.753	4.644E-05
AC006504.5	0.810	1.753	2.492E-06
CDC42EP1	0.810	1.753	4.168E-05
ALAS1	0.811	1.754	0.000E+00
MFSD3	0.811	1.755	8.385E-06
ARHGEF39	0.812	1.755	3.070E-04
FAM89B	0.812	1.755	1.183E-06
AC007541.1	0.812	1.756	2.395E-03
RMI2	0.812	1.756	4.640E-06
RFFL	0.813	1.756	0.000E+00
DUX4L50	0.813	1.757	2.303E-03
NDUFAF1	0.813	1.757	1.705E-04
AC008555.6	0.813	1.757	1.820E-05
MAF1	0.813	1.757	1.978E-10
PKIG	0.815	1.759	9.175E-04
IRF1	0.815	1.760	3.130E-07
NCLN	0.816	1.760	6.907E-10
NUP153-AS1	0.816	1.761	1.564E-04
IL4R	0.816	1.761	0.000E+00

ZNF174	0.816	1.761	0.000E+00
WASH4P	0.816	1.761	9.781E-07
LGALS3	0.817	1.761	5.204E-05
GPX3	0.817	1.762	7.878E-04
AL139011.1	0.817	1.762	2.365E-03
ZNF239	0.817	1.762	5.674E-07
BTBD2	0.817	1.762	0.000E+00
ZBTB47	0.819	1.764	3.445E-08
MOB3C	0.819	1.764	0.000E+00
LINC02193	0.819	1.765	2.957E-03
AC022400.7	0.819	1.765	7.666E-05
DBP	0.820	1.765	1.539E-07
PTPN6	0.820	1.765	0.000E+00
PCGF5	0.820	1.765	2.013E-06
ALKBH7	0.820	1.765	5.013E-04
AC108047.1	0.820	1.765	8.135E-03
FAM90A1	0.820	1.766	6.666E-03
GALNS	0.820	1.766	0.000E+00
PXMP4	0.820	1.766	1.295E-10
GPBAR1	0.820	1.766	7.989E-06
CLPP	0.821	1.767	0.000E+00
P3H4	0.821	1.767	1.691E-07
ERGIC1	0.822	1.768	0.000E+00
LINC01191	0.823	1.769	3.217E-03
AC005840.4	0.824	1.770	7.508E-08
MRPL2	0.824	1.771	1.077E-07
TRAF2	0.825	1.771	0.000E+00
FAM174C	0.825	1.771	3.702E-05
TRAV14DV4	0.825	1.772	1.613E-03
AC008764.8	0.825	1.772	1.945E-07
AC018766.1	0.826	1.772	3.868E-03
AC040970.1	0.826	1.772	1.790E-09
LRFN4	0.826	1.773	2.818E-04
CIAO3	0.826	1.773	0.000E+00
MOSPD2	0.826	1.773	4.933E-04
PLPP7	0.826	1.773	2.390E-03
HDHD3	0.827	1.774	0.000E+00
LBH	0.827	1.774	2.234E-06
AP5Z1	0.828	1.775	0.000E+00
APOBEC3F	0.828	1.775	2.175E-09
P2RX5-TAX1BP3	0.828	1.776	3.562E-04
CDIP1	0.829	1.776	3.072E-04
LINC02207	0.829	1.777	9.962E-04
TSPAN2	0.829	1.777	1.135E-03
AGPAT2	0.829	1.777	2.827E-07
AC009093.2	0.830	1.777	7.622E-04
RPARP-AS1	0.830	1.777	1.795E-10
MAML1	0.830	1.778	4.387E-09
MPV17L2	0.831	1.779	0.000E+00
AL035461.2	0.831	1.779	6.045E-05
H2AC6	0.832	1.780	2.460E-03
JPT2	0.832	1.780	2.504E-07
RN7SL172P	0.832	1.781	8.471E-04
AC012306.2	0.833	1.781	7.246E-04
FXYD2	0.833	1.781	1.187E-03

AC100861.1	0.833	1.781	1.925E-07
AC091133.1	0.833	1.781	9.828E-05
KLHL22	0.833	1.782	0.000E+00
EIF2S2P4	0.833	1.782	3.200E-03
CD19	0.834	1.783	7.347E-04
PI16	0.834	1.783	3.074E-04
ZNF74	0.834	1.783	7.939E-08
PPP1R12B	0.834	1.783	6.023E-07
H3-3A	0.835	1.783	1.329E-09
THNSL1	0.835	1.783	1.504E-05
AP000873.1	0.835	1.784	2.192E-07
CHST11	0.836	1.785	0.000E+00
SDCBP2	0.836	1.786	8.415E-05
PRR13	0.836	1.786	4.236E-10
AL162431.1	0.836	1.786	7.960E-07
FCGR2B	0.837	1.786	1.367E-04
AC009133.3	0.837	1.786	0.000E+00
B3GNT10	0.837	1.786	9.752E-04
TUBA1A	0.838	1.787	8.013E-05
ARID3A	0.839	1.789	3.728E-10
FGFR4	0.839	1.789	3.578E-05
GNG7	0.839	1.789	8.839E-09
BAIAP2L2	0.839	1.789	4.905E-07
SLC16A5	0.840	1.790	0.000E+00
SSBP4	0.840	1.790	1.360E-06
CPNE7	0.840	1.790	6.141E-03
PXN	0.840	1.791	0.000E+00
COL9A2	0.841	1.791	5.263E-09
FAM167B	0.841	1.791	1.596E-03
MAZ	0.841	1.791	0.000E+00
KATNBL1	0.842	1.792	6.600E-07
TNFAIP1	0.842	1.793	0.000E+00
GLIPR2	0.842	1.793	0.000E+00
AL513534.2	0.843	1.793	3.215E-03
DHDH	0.843	1.794	2.206E-03
FXR2	0.844	1.794	0.000E+00
KCNIP2	0.844	1.795	1.274E-08
AL160272.1	0.844	1.795	3.212E-04
NCOA4	0.844	1.796	8.736E-06
IFNAR1	0.845	1.796	2.167E-09
PGS1	0.845	1.796	0.000E+00
CAMK2G	0.845	1.796	0.000E+00
TRIOBP	0.845	1.797	0.000E+00
RPS2P55	0.845	1.797	2.561E-03
SCYL1	0.846	1.797	0.000E+00
LINC01800	0.846	1.797	5.533E-03
AC108479.1	0.846	1.798	1.960E-03
GPER1	0.847	1.798	4.847E-03
HEXIM2	0.847	1.799	2.990E-06
CHKB-CPT1B	0.847	1.799	1.912E-06
USB1	0.848	1.799	0.000E+00
HDAC4-AS1	0.848	1.800	8.471E-04
SGCA	0.849	1.801	7.120E-03
AL022328.4	0.849	1.801	8.737E-05
DUSP23	0.849	1.802	2.242E-06
PAK1	0.850	1.802	4.088E-08
NRADDP	0.850	1.802	7.084E-04
ADRB2	0.850	1.802	4.539E-10
ZNF674	0.850	1.803	3.634E-08
TRAPPC12	0.850	1.803	2.999E-08
GID4	0.850	1.803	8.063E-10
NFIC	0.851	1.803	0.000E+00
SCAP	0.851	1.803	0.000E+00
AL133410.1	0.851	1.803	1.243E-07
SCAMP4	0.851	1.804	0.000E+00
CBR1	0.851	1.804	9.801E-07
ABCB6	0.851	1.804	4.862E-06
ZKSCAN4	0.852	1.805	3.432E-09
SOCS1	0.853	1.806	3.920E-03
CCDC189	0.853	1.806	3.212E-07
LAMP2	0.853	1.806	1.493E-06
SLC25A1	0.854	1.807	0.000E+00
AC068888.2	0.854	1.807	6.411E-03
FBXL15	0.854	1.807	8.312E-04
SMIM29	0.854	1.808	0.000E+00
FAM104A	0.854	1.808	0.000E+00
CBY1	0.855	1.809	0.000E+00
FAM207A	0.855	1.809	7.333E-08
TRDV1	0.856	1.810	1.184E-03
TRMT1L	0.856	1.810	3.054E-10
DUSP7	0.856	1.810	1.060E-10
CLEC17A	0.856	1.810	1.680E-04
EIF4EBP3	0.856	1.811	2.442E-05
PRAM1	0.856	1.811	4.056E-08
FKBP1C	0.857	1.811	3.074E-06
AC012306.3	0.857	1.812	1.081E-04
PRRT3	0.858	1.812	6.801E-10
ZNF564	0.858	1.813	1.302E-03
IFNAR2	0.858	1.813	0.000E+00
AC016747.1	0.858	1.813	1.256E-05
AL355574.1	0.859	1.814	8.326E-07
TTC26	0.859	1.814	1.493E-03
UCN	0.859	1.814	7.077E-05
AC008895.1	0.859	1.814	3.368E-04
SLA	0.859	1.814	2.038E-10
ZNF358	0.860	1.815	1.075E-04
CAPS	0.860	1.816	1.038E-10
WNT1	0.861	1.816	2.400E-04
SULT1B1	0.861	1.816	6.129E-04
TPRN	0.861	1.816	5.804E-10
AC026367.3	0.861	1.817	1.078E-05
ATP6V1B2	0.861	1.817	6.549E-10
FARSA	0.862	1.817	5.342E-10
ABHD5	0.862	1.817	2.063E-06
ATP2A3	0.862	1.818	0.000E+00
AJM1	0.862	1.818	1.378E-04
SDCBP	0.863	1.819	5.956E-06
ZNF319	0.864	1.819	0.000E+00
POLR2J3	0.864	1.820	5.702E-07
CLCN4	0.864	1.820	1.509E-05

APTR	0.864	1.820	1.051E-06
TP53RK	0.865	1.821	0.000E+00
KLHL25	0.865	1.821	0.000E+00
DNLZ	0.865	1.821	7.195E-04
AC135721.1	0.865	1.821	1.226E-04
S100A5	0.866	1.822	2.112E-03
PARP10	0.866	1.823	9.756E-09
CERS4	0.867	1.824	0.000E+00
TBC1D10B	0.867	1.824	0.000E+00
CST7	0.867	1.824	3.577E-08
AMACR	0.867	1.824	1.182E-04
CFP	0.868	1.826	0.000E+00
TUBA4A	0.869	1.826	0.000E+00
SSTR3	0.869	1.826	1.871E-05
CD68	0.869	1.826	9.907E-05
PEX11G	0.869	1.827	4.743E-03
TIAM2	0.869	1.827	6.418E-09
BCORL1	0.870	1.828	0.000E+00
DPCD	0.870	1.828	1.003E-05
SLC6A6	0.870	1.828	1.577E-06
LGALS12	0.871	1.828	1.784E-05
FOXP3	0.871	1.829	5.168E-07
BAIAP3	0.871	1.829	1.842E-04
MCOLN1	0.872	1.830	0.000E+00
CBWD4P	0.872	1.830	3.992E-03
SIRT4	0.872	1.830	7.965E-04
MAEA	0.872	1.831	0.000E+00
PRDM8	0.873	1.832	0.000E+00
FIZ1	0.874	1.833	0.000E+00
ZNF763	0.874	1.833	1.783E-09
MIDN	0.876	1.835	7.263E-08
RBM42	0.878	1.837	0.000E+00
RALBP1	0.878	1.838	5.098E-09
TDP2	0.878	1.838	1.093E-07
EEF1AKNMT	0.878	1.838	3.735E-05
AC016582.1	0.878	1.838	7.803E-03
MVB12A	0.878	1.838	0.000E+00
NUBP2	0.878	1.838	1.641E-09
BID	0.879	1.840	1.631E-09
AC007780.1	0.880	1.840	0.000E+00
PPP1R37	0.880	1.840	0.000E+00
LYPLA2	0.880	1.841	0.000E+00
SMIM35	0.881	1.841	5.617E-05
MARCHF8	0.881	1.842	1.028E-05
ABCA17P	0.881	1.842	1.843E-03
NFKB2	0.881	1.842	0.000E+00
AD000864.1	0.881	1.842	3.123E-04
CCR5	0.881	1.842	1.762E-05
BAK1P1	0.882	1.843	7.831E-03
GLI4	0.882	1.843	1.699E-10
SDHAF1	0.882	1.843	1.579E-07
ANTKMT	0.882	1.843	5.724E-04
SRPK1	0.883	1.844	3.584E-10
POLA2	0.883	1.844	0.000E+00
AL592295.4	0.883	1.845	9.580E-05
FBXL19	0.884	1.845	0.000E+00
ARRDC1	0.884	1.846	0.000E+00
METTL2B	0.884	1.846	0.000E+00
MVP	0.885	1.846	0.000E+00
STX10	0.885	1.847	0.000E+00
MARCHF2	0.886	1.848	3.266E-07
C18orf32	0.886	1.848	0.000E+00
DND1	0.886	1.849	4.078E-08
TLR8	0.886	1.849	4.130E-05
DLGAP1-AS1	0.887	1.850	0.000E+00
GRK6	0.888	1.850	0.000E+00
ZNF444	0.889	1.852	0.000E+00
LIMD1-AS1	0.889	1.852	3.406E-05
HELZ2	0.889	1.852	8.125E-06
PTOV1-AS2	0.889	1.853	0.000E+00
DRAP1	0.890	1.853	0.000E+00
PRDX2	0.890	1.853	1.899E-09
MKNK1	0.893	1.857	0.000E+00
HNRNPKP4	0.893	1.857	2.025E-07
ZNF688	0.893	1.857	7.198E-09
AC009133.4	0.894	1.858	5.714E-03
PPP2R3B	0.894	1.858	8.378E-04
ARFIP1	0.894	1.858	2.297E-06
BCL2L12	0.895	1.859	0.000E+00
SOX15	0.895	1.859	4.629E-03
MMP17	0.895	1.860	1.385E-05
CBX7	0.895	1.860	0.000E+00
RABEP2	0.895	1.860	0.000E+00
ARPC5	0.895	1.860	0.000E+00
CCDC71L	0.895	1.860	3.533E-07
MBD3	0.896	1.860	1.934E-09
CD320	0.896	1.861	2.898E-09
TUSC2	0.896	1.861	0.000E+00
CDPF1	0.896	1.861	9.961E-08
BCDIN3D	0.896	1.861	1.498E-10
AC084125.4	0.897	1.862	9.265E-05
U47924.3	0.898	1.864	1.119E-03
SLBP-DT	0.898	1.864	5.027E-08
UBE2M	0.898	1.864	0.000E+00
FANCF	0.899	1.864	0.000E+00
DOLK	0.899	1.865	0.000E+00
Z97634.1	0.900	1.866	9.219E-06
DLEU2	0.901	1.867	8.377E-09
ZNF710	0.901	1.867	0.000E+00
C9orf16	0.901	1.867	1.301E-05
AL162274.2	0.901	1.868	2.741E-04
RARG	0.902	1.868	0.000E+00
AC008537.3	0.902	1.869	6.108E-07
THEM6	0.902	1.869	2.504E-07
RASL11A	0.902	1.869	3.688E-04
AC211476.6	0.903	1.869	2.657E-03
CTDP1	0.903	1.870	0.000E+00
PPP2R5B	0.904	1.872	0.000E+00
TMEM63B	0.904	1.872	0.000E+00
PIGQ	0.905	1.872	0.000E+00

RASSF2	0.905	1.873	1.554E-08
RNU7-40P	0.905	1.873	1.380E-04
LXN	0.905	1.873	8.178E-06
CHMP1A	0.906	1.874	0.000E+00
CRACR2B	0.907	1.876	1.708E-04
IL2RG	0.907	1.876	0.000E+00
INO80E	0.908	1.876	0.000E+00
OGFR	0.908	1.876	0.000E+00
AC072061.1	0.908	1.876	1.469E-05
ARMH2	0.908	1.877	1.712E-03
MIEN1	0.908	1.877	0.000E+00
KIAA0040	0.909	1.878	7.468E-07
NRGN	0.909	1.878	1.406E-03
AC139100.2	0.909	1.878	6.095E-03
TFR2	0.910	1.879	0.000E+00
NHSL2	0.910	1.879	1.445E-05
PRR14	0.910	1.880	0.000E+00
CARD8-AS1	0.911	1.880	6.295E-06
ITPKC	0.911	1.880	0.000E+00
GIMAP6	0.911	1.881	7.500E-10
AGAP5	0.911	1.881	6.250E-03
BCL3	0.911	1.881	5.239E-05
BOLA1	0.912	1.881	1.106E-06
MIR635	0.912	1.882	3.978E-03
SORD	0.912	1.882	0.000E+00
LILRA3	0.912	1.882	3.035E-03
RHOB	0.913	1.883	2.807E-06
C9orf106	0.913	1.883	2.414E-05
AC129492.1	0.913	1.883	1.118E-06
GNAI2	0.913	1.883	0.000E+00
HGH1	0.913	1.883	2.815E-08
KEAP1	0.914	1.884	0.000E+00
TNFRSF4	0.914	1.884	7.674E-04
TMEM273	0.914	1.885	2.836E-08
FSCN1	0.915	1.885	2.720E-06
HEXD	0.915	1.885	1.170E-10
GATD1	0.915	1.886	0.000E+00
AL355377.4	0.915	1.886	4.119E-04
UBXN2B	0.915	1.886	1.403E-07
C11orf98	0.916	1.886	4.319E-03
THOC5	0.916	1.887	0.000E+00
ACTB	0.918	1.889	0.000E+00
NLRC4	0.918	1.889	4.436E-05
LTBR	0.918	1.890	1.023E-10
CMTM8	0.919	1.890	6.250E-07
PAQR8	0.919	1.890	2.970E-04
PSTPIP1	0.919	1.891	0.000E+00
SEC1P	0.919	1.891	1.674E-03
NMI	0.920	1.891	5.677E-07
AC021106.1	0.920	1.892	4.279E-05
UBE2B	0.921	1.894	1.364E-07
SEMA4A	0.921	1.894	2.236E-09
SPACA9	0.922	1.894	9.124E-09
AL606760.2	0.922	1.895	2.967E-04
RELB	0.922	1.895	2.458E-09
PGLS	0.923	1.896	0.000E+00
MOSPD3	0.924	1.897	0.000E+00
CAMKK1	0.924	1.897	0.000E+00
AGAP2	0.924	1.897	0.000E+00
WDTC1	0.925	1.899	0.000E+00
ISG20	0.925	1.899	0.000E+00
FKBP2	0.925	1.899	5.443E-04
TASL	0.925	1.899	1.586E-07
TENT5C	0.925	1.899	4.880E-04
BBS1	0.925	1.899	4.549E-07
ANXA11	0.926	1.900	0.000E+00
MAP7D1	0.926	1.900	0.000E+00
FAM53C	0.927	1.901	1.352E-07
RNU6-892P	0.928	1.902	2.037E-03
MKRN1	0.928	1.902	0.000E+00
SUV39H2-DT	0.928	1.903	4.453E-03
ADORA2A	0.928	1.903	6.098E-06
RIPOR3	0.929	1.903	2.720E-03
RNU6-611P	0.929	1.904	4.328E-03
LMTK3	0.929	1.904	1.952E-09
HYAL3	0.930	1.905	1.356E-08
WWC3	0.930	1.905	0.000E+00
BBS12	0.930	1.905	1.251E-08
CHPT1	0.930	1.906	6.379E-10
COL7A1	0.931	1.906	1.061E-06
GIMAP8	0.931	1.907	1.001E-05
RGS16	0.932	1.908	1.295E-03
TMEM115	0.932	1.908	0.000E+00
YBX1	0.934	1.910	0.000E+00
HMGA1P4	0.934	1.911	5.124E-05
GP9	0.935	1.912	7.827E-03
LAGE3	0.935	1.912	1.039E-07
UBE2D1	0.936	1.913	6.478E-05
GUCA1B	0.936	1.913	0.000E+00
AC005498.3	0.937	1.914	2.878E-03
LASP1	0.937	1.914	0.000E+00
AL513190.1	0.937	1.915	2.724E-03
LMAN2	0.937	1.915	0.000E+00
TRIM61	0.937	1.915	1.900E-03
ZSWIM1	0.938	1.915	0.000E+00
C1orf116	0.938	1.915	4.581E-03
NIBAN3	0.938	1.916	1.415E-06
RITA1	0.938	1.916	0.000E+00
WNT5B	0.938	1.916	3.487E-03
AC022217.3	0.939	1.917	6.116E-04
SIGLEC14	0.939	1.917	1.864E-05
LINC02870	0.939	1.918	1.471E-03
SLAMF8	0.940	1.919	3.757E-05
NSFL1C	0.940	1.919	0.000E+00
POU5F2	0.940	1.919	2.253E-03
AL844908.1	0.941	1.920	1.756E-07
TRAV24	0.941	1.920	4.180E-04
GTF2IP13	0.942	1.921	1.964E-08
MLF2	0.942	1.922	0.000E+00
RALB	0.943	1.923	4.086E-07

SLC35C1	0.943	1.923	0.000E+00
NMNAT1	0.944	1.923	1.097E-08
GNG5	0.944	1.923	0.000E+00
TEX22	0.944	1.924	3.882E-07
AC010618.3	0.944	1.924	0.000E+00
AC027601.5	0.945	1.925	3.682E-03
TRBJ2-3	0.945	1.925	1.255E-06
YBX1P1	0.945	1.925	1.057E-07
DOCK9-DT	0.946	1.926	7.577E-06
ZNF580	0.946	1.926	0.000E+00
ARHGAP45	0.946	1.927	0.000E+00
FNDC10	0.946	1.927	8.762E-09
AC005785.2	0.948	1.929	7.040E-06
AC093297.2	0.948	1.929	5.317E-07
MARCKSL1	0.948	1.930	0.000E+00
CNPPD1	0.948	1.930	0.000E+00
GRAMD1A	0.949	1.930	0.000E+00
AC024270.4	0.949	1.931	8.874E-04
OSGIN1	0.949	1.931	2.540E-09
AC016957.2	0.950	1.932	8.377E-09
IQCC	0.950	1.932	6.892E-08
RPL21P35	0.950	1.932	5.743E-03
AC007292.1	0.951	1.933	4.488E-04
LRRC75A	0.951	1.933	0.000E+00
AC090114.2	0.951	1.933	0.000E+00
AP006621.5	0.952	1.934	6.589E-06
MIR25	0.952	1.934	4.819E-05
MMP25-AS1	0.952	1.934	0.000E+00
RILPL1	0.952	1.935	0.000E+00
AL135844.1	0.952	1.935	5.897E-04
ZNF132	0.953	1.935	1.661E-06
LINC01138	0.953	1.936	0.000E+00
ABTB2	0.954	1.938	9.347E-06
TOX2	0.954	1.938	1.638E-06
AC021127.1	0.955	1.939	1.087E-03
SYNPO	0.956	1.940	7.722E-05
FTL	0.956	1.940	1.692E-10
DNAJC27-AS1	0.956	1.940	6.529E-05
LRIF1	0.956	1.940	5.032E-05
AL133367.1	0.956	1.941	3.940E-10
ZNF771	0.956	1.941	7.361E-06
ARSA	0.957	1.941	0.000E+00
SLC44A2	0.957	1.941	0.000E+00
AL139289.1	0.957	1.941	2.594E-03
AL096701.3	0.957	1.941	2.478E-07
APBA3	0.957	1.941	0.000E+00
HAGHL	0.957	1.942	2.590E-05
CD27	0.957	1.942	3.525E-07
SPINT1	0.958	1.942	1.853E-09
AC127070.4	0.958	1.943	8.292E-07
AC024909.1	0.958	1.943	5.584E-07
PPP1R13L	0.958	1.943	0.000E+00
HES4	0.959	1.944	6.674E-03
IGKV3-11	0.959	1.944	3.991E-03
PCTP	0.959	1.944	0.000E+00

PFN1	0.959	1.945	0.000E+00
PPP1R14A	0.960	1.945	5.546E-03
AC114936.2	0.960	1.945	9.925E-03
JOSD2	0.960	1.945	2.358E-05
AL683842.1	0.960	1.945	7.205E-03
GFOD2	0.960	1.946	0.000E+00
AL138995.1	0.961	1.946	1.653E-05
AC011676.5	0.961	1.947	6.096E-03
AC011447.7	0.962	1.948	1.299E-04
PPM1F	0.962	1.948	0.000E+00
RBCK1	0.962	1.949	0.000E+00
DEDD2	0.963	1.949	0.000E+00
ZFPM1	0.963	1.950	4.618E-06
RNU4ATAC18P	0.963	1.950	3.855E-03
TMOD4	0.964	1.950	6.482E-08
LINC00324	0.964	1.951	5.193E-09
AC022211.1	0.965	1.952	2.683E-03
TAGAP	0.965	1.953	4.258E-10
CORO1A	0.965	1.953	0.000E+00
CBX8	0.966	1.953	1.282E-09
AC138028.6	0.966	1.953	1.536E-04
GRINA	0.966	1.953	0.000E+00
KDF1	0.966	1.953	1.126E-06
ARHGEF2	0.966	1.954	0.000E+00
AC124248.1	0.967	1.955	3.804E-04
ZNRD2-AS1	0.967	1.955	2.841E-08
TAGLN2P1	0.968	1.956	7.310E-04
ARFRP1	0.968	1.957	0.000E+00
COPE	0.969	1.958	0.000E+00
AC063977.1	0.970	1.958	1.722E-03
BCKDK	0.971	1.960	0.000E+00
RN7SL832P	0.971	1.960	4.980E-03
ACD	0.971	1.960	0.000E+00
TBC1D10C	0.971	1.960	0.000E+00
CYBA	0.971	1.961	0.000E+00
HRH2	0.971	1.961	1.847E-06
KMT5C	0.971	1.961	0.000E+00
TYMP	0.972	1.961	1.183E-07
AP000787.1	0.972	1.961	2.008E-10
GABARAP	0.973	1.963	0.000E+00
MRPL12	0.974	1.964	3.058E-03
AL359532.1	0.975	1.965	1.470E-05
ALPK3	0.976	1.967	5.222E-06
NDEL1	0.977	1.969	0.000E+00
PPP4C	0.978	1.969	0.000E+00
AC022415.1	0.978	1.969	8.644E-03
CRIP3	0.978	1.969	4.805E-10
MLLT1	0.978	1.970	0.000E+00
XXYLT1-AS2	0.979	1.972	7.200E-03
MTX1	0.980	1.972	0.000E+00
GPR137	0.980	1.973	0.000E+00
SNX11	0.981	1.973	0.000E+00
AC021016.1	0.981	1.974	2.892E-10
BATF	0.982	1.975	8.753E-08
AL022341.1	0.982	1.975	2.232E-04

MED19	0.983	1.976	0.000E+00
AC008079.1	0.983	1.976	1.307E-03
AC097382.3	0.983	1.976	3.353E-07
AL133351.4	0.983	1.977	3.895E-07
LETM2	0.984	1.978	6.092E-08
ARAP1	0.985	1.979	0.000E+00
CAT	0.985	1.979	0.000E+00
PRDX6	0.985	1.980	3.097E-10
SOX8	0.985	1.980	1.393E-06
AL135818.2	0.986	1.980	1.150E-04
FAM200A	0.986	1.981	0.000E+00
ZBTB7A	0.986	1.981	0.000E+00
MX2	0.986	1.981	1.295E-07
AC004069.1	0.986	1.981	1.812E-06
CCDC61	0.987	1.982	0.000E+00
H2BC12	0.987	1.982	1.232E-06
MEX3D	0.988	1.983	1.142E-10
TLNRD1	0.988	1.983	0.000E+00
HPCAL1	0.988	1.984	0.000E+00
PDE2A-AS2	0.988	1.984	1.187E-07
AC010542.3	0.988	1.984	4.431E-03
HYAL2	0.989	1.984	0.000E+00
BLOC1S3	0.990	1.986	0.000E+00
AC027801.1	0.990	1.986	0.000E+00
LINC00899	0.990	1.987	1.803E-08
SLC66A2	0.991	1.987	0.000E+00
LIPE	0.991	1.987	0.000E+00
PCP2	0.993	1.990	1.103E-05
AMDHD2	0.993	1.991	0.000E+00
TRIM46	0.993	1.991	0.000E+00
HDAC7	0.993	1.991	0.000E+00
GDPGP1	0.994	1.991	0.000E+00
CEL	0.994	1.991	1.584E-05
AL357033.3	0.994	1.992	1.592E-06
TREX2	0.994	1.992	1.287E-07
HSDL2	0.994	1.992	1.222E-07
LINC00847	0.995	1.993	0.000E+00
TRADD	0.995	1.993	0.000E+00
MFAP1	0.995	1.994	0.000E+00
AC015819.1	0.996	1.994	1.965E-09
AC027682.1	0.996	1.994	6.209E-04
AC110285.6	0.996	1.995	2.845E-03
AF106564.1	0.996	1.995	1.316E-04
UNC13D	0.996	1.995	0.000E+00
PLXNC1	0.996	1.995	2.871E-07
AC008105.2	0.997	1.995	0.000E+00
AC087286.4	0.997	1.995	1.327E-03
PBX1	0.998	1.997	1.142E-06
TWF2	0.998	1.998	0.000E+00
TSPAN10	0.999	1.999	7.974E-03
F11R	0.999	1.999	0.000E+00
CSK	0.999	1.999	0.000E+00
AC009119.1	0.999	1.999	2.045E-05
CASP5	1.000	2.000	2.398E-03
GPX1	1.000	2.000	2.803E-05

VSIG10	1.001	2.001	1.415E-06
CD300A	1.001	2.002	0.000E+00
NPDC1	1.001	2.002	4.391E-05
RAB40C	1.002	2.002	0.000E+00
PPIAP45	1.002	2.003	3.369E-03
FAM214B	1.002	2.003	0.000E+00
N4BP3	1.003	2.004	4.732E-07
GFI1B	1.003	2.004	1.298E-04
PPP1R16A	1.003	2.004	6.878E-09
ZNF341	1.003	2.004	0.000E+00
TMEM184B	1.003	2.005	0.000E+00
SAMD10	1.003	2.005	0.000E+00
LIMD2	1.004	2.005	0.000E+00
NR6A1	1.004	2.005	1.013E-10
AC060766.3	1.004	2.005	3.112E-05
BRAT1	1.004	2.005	0.000E+00
SLC11A1	1.004	2.006	3.780E-09
G6PD	1.004	2.006	0.000E+00
H2BC6	1.004	2.006	4.311E-05
AL117336.2	1.004	2.006	4.810E-09
ESRRA	1.004	2.006	0.000E+00
ANKRD13D	1.005	2.006	0.000E+00
FZD2	1.005	2.007	6.441E-06
TTC9C	1.006	2.008	0.000E+00
CTDSP1	1.006	2.008	0.000E+00
EFNA4	1.007	2.009	0.000E+00
TRPC6	1.007	2.010	6.730E-03
KBTBD6	1.007	2.010	1.900E-10
RGS3	1.008	2.011	2.807E-09
AC016831.1	1.008	2.012	3.188E-04
U47924.2	1.009	2.013	3.128E-04
VNN1	1.009	2.013	1.401E-04
RAB4B	1.010	2.014	0.000E+00
RNF149	1.010	2.014	2.141E-08
CDC42-IT1	1.010	2.014	2.786E-04
AL358781.1	1.010	2.014	2.248E-04
MIR6753	1.011	2.015	2.710E-05
AC132872.1	1.011	2.015	0.000E+00
TBC1D17	1.011	2.015	0.000E+00
OR52K3P	1.012	2.017	2.256E-05
DNTTIP1	1.012	2.017	1.192E-10
PNPLA2	1.013	2.018	0.000E+00
AL358472.4	1.013	2.018	4.190E-03
ELOF1	1.013	2.019	0.000E+00
ZNF865	1.014	2.019	2.482E-09
SLC40A1	1.014	2.019	1.442E-06
CHST15	1.014	2.020	1.180E-07
ACY1	1.014	2.020	3.928E-05
MPPE1	1.014	2.020	0.000E+00
CHAC2	1.014	2.020	5.214E-04
MZT2B	1.015	2.021	3.413E-04
CNIH2	1.016	2.022	1.483E-04
UPF1	1.016	2.022	0.000E+00
PDLIM2	1.016	2.023	0.000E+00
ARPC4	1.017	2.023	0.000E+00

S100A9	1.017	2.024	1.526E-05
RTN3	1.018	2.025	0.000E+00
PNOC	1.018	2.025	5.153E-06
RTN4RL1	1.019	2.026	3.425E-03
AC108134.3	1.019	2.027	0.000E+00
AC004241.1	1.019	2.027	5.501E-06
AC004466.1	1.020	2.028	2.091E-05
CUEDC1	1.020	2.028	0.000E+00
AL031733.2	1.020	2.028	4.606E-04
HDAC5	1.021	2.029	0.000E+00
MIR3671	1.021	2.029	1.355E-03
VPS9D1	1.022	2.030	0.000E+00
AL109741.1	1.022	2.031	1.812E-06
EGLN1	1.022	2.031	1.215E-06
CCDC102A	1.022	2.031	0.000E+00
TMEM234	1.023	2.032	0.000E+00
CHMP2A	1.023	2.033	0.000E+00
NECTIN2	1.024	2.033	1.877E-03
LINC01814	1.024	2.034	3.421E-06
CCM2	1.025	2.034	0.000E+00
SERPINA1	1.025	2.035	2.639E-09
SCARF2	1.025	2.035	2.015E-05
CYP27A1	1.025	2.036	3.161E-07
AC106037.2	1.026	2.036	3.446E-03
LINC02273	1.026	2.036	8.238E-04
PCBP3	1.026	2.037	1.362E-05
UNKL	1.026	2.037	0.000E+00
AC015871.8	1.027	2.038	1.631E-03
MED11	1.027	2.038	0.000E+00
CAVIN3	1.027	2.038	1.752E-03
C14orf119	1.027	2.038	1.448E-07
CD79B	1.028	2.039	8.419E-05
RANBP10	1.029	2.040	0.000E+00
TMCC3	1.029	2.040	2.101E-05
MAPK3	1.029	2.040	0.000E+00
RAVER1	1.029	2.041	1.709E-09
AC004466.2	1.030	2.042	3.429E-05
MON1A	1.030	2.042	2.953E-07
FCRLA	1.030	2.042	1.129E-04
TMEM139	1.031	2.043	7.719E-04
TPGS1	1.031	2.043	1.102E-04
ZNF460-AS1	1.031	2.044	0.000E+00
FBXL16	1.032	2.044	2.214E-09
NARF	1.033	2.046	0.000E+00
ANKRD22	1.033	2.046	1.057E-03
AC011448.1	1.034	2.047	9.247E-05
AL162377.1	1.034	2.047	1.183E-09
HOXB-AS3	1.034	2.048	1.839E-03
PRICKLE3	1.034	2.048	0.000E+00
NOTCH1	1.034	2.048	5.990E-08
AURKAIP1	1.034	2.048	2.336E-09
PRKCZ	1.034	2.048	0.000E+00
TLE3	1.034	2.048	3.747E-08
TRAPPC1	1.035	2.049	0.000E+00
AL391832.3	1.035	2.049	4.319E-05
FAM71F2	1.035	2.049	2.398E-08
AC017116.2	1.035	2.050	1.730E-08
FSCN2	1.036	2.050	2.005E-05
GSK3A	1.036	2.050	0.000E+00
TGFB1	1.036	2.050	0.000E+00
JAK3	1.036	2.051	0.000E+00
AC009974.1	1.036	2.051	2.754E-03
CYHR1	1.037	2.052	0.000E+00
ZNF547	1.037	2.052	2.143E-05
AC004494.1	1.037	2.053	5.189E-03
NUSAP1	1.038	2.053	3.190E-04
FCGR1A	1.038	2.053	1.552E-05
TMEM79	1.038	2.053	0.000E+00
NOG	1.038	2.054	1.030E-03
C15orf39	1.039	2.054	1.162E-10
C3orf62	1.039	2.055	0.000E+00
SLC2A4RG	1.039	2.055	0.000E+00
TPRG1-AS1	1.040	2.056	9.875E-03
SH2D4B	1.040	2.056	9.986E-03
CPPED1	1.040	2.056	3.061E-07
HSH2D	1.040	2.057	0.000E+00
HSD17B1	1.040	2.057	4.478E-04
AC136475.5	1.041	2.058	9.403E-03
TCIRG1	1.041	2.058	0.000E+00
HDGFL2	1.042	2.059	0.000E+00
GRAP	1.042	2.059	2.228E-10
IL10RB	1.042	2.059	0.000E+00
AC096887.2	1.043	2.061	5.352E-03
NT5M	1.044	2.062	2.201E-04
SF3A2	1.045	2.063	0.000E+00
KRT8P12	1.045	2.063	3.814E-08
LIN7A	1.045	2.063	3.837E-06
RNPEPL1	1.047	2.067	0.000E+00
MPST	1.047	2.067	0.000E+00
PSPN	1.048	2.067	1.089E-10
AC008894.3	1.048	2.067	2.356E-06
FBXW5	1.050	2.071	0.000E+00
AC110048.2	1.050	2.071	1.658E-03
KLF16	1.050	2.071	0.000E+00
MSANTD1	1.051	2.071	1.036E-08
GSPT1	1.051	2.072	0.000E+00
TTI2	1.051	2.072	0.000E+00
EFCAB12	1.052	2.073	0.000E+00
PLK1	1.052	2.074	7.126E-07
KIAA1614	1.053	2.075	5.275E-03
LINC00528	1.053	2.075	0.000E+00
ANKRD44-IT1	1.053	2.075	5.334E-06
MIR503HG	1.054	2.076	5.104E-04
AC005224.2	1.054	2.076	3.777E-03
MSC	1.054	2.076	9.580E-05
RGS6	1.054	2.077	2.441E-04
PROB1	1.054	2.077	2.727E-09
SLC16A4	1.055	2.078	6.572E-04
AC027601.3	1.055	2.078	3.399E-03
H3P14	1.057	2.081	1.090E-03

LOXL1	1.057	2.081	8.835E-06
RNF167	1.058	2.082	0.000E+00
AC024060.2	1.058	2.082	8.089E-07
CBX3P9	1.058	2.082	8.867E-03
AP006621.3	1.058	2.082	6.930E-09
AP001462.1	1.058	2.082	2.867E-06
AC025279.1	1.059	2.083	7.116E-06
CBR3	1.060	2.085	6.361E-05
TPTEP2	1.061	2.086	0.000E+00
RMI1	1.061	2.086	4.798E-06
CAP1P2	1.061	2.086	3.634E-03
EPN1	1.061	2.086	0.000E+00
AC099343.3	1.061	2.086	1.173E-06
TNFSF10	1.061	2.087	1.979E-05
HOXC4	1.061	2.087	6.049E-07
AC120057.4	1.062	2.087	8.471E-03
TMUB1	1.062	2.087	1.999E-10
SIRT6	1.062	2.088	0.000E+00
AC127024.4	1.062	2.088	2.072E-03
CCDC88B	1.063	2.089	0.000E+00
EXOSC4	1.063	2.089	9.644E-09
MAPK13	1.065	2.091	0.000E+00
ZNF526	1.065	2.092	0.000E+00
KRT18P31	1.065	2.092	7.346E-03
STK11	1.066	2.094	0.000E+00
TMEM222	1.066	2.094	0.000E+00
AC009318.3	1.067	2.095	3.589E-03
SPATA2L	1.068	2.096	2.845E-08
AC012313.10	1.068	2.097	2.033E-07
ULK1	1.070	2.099	0.000E+00
DNAJC30	1.070	2.099	0.000E+00
VSIR	1.070	2.100	0.000E+00
FKBP1B	1.070	2.100	2.879E-05
BX640514.2	1.070	2.100	7.946E-04
DAXX	1.071	2.100	2.110E-05
FBXO46	1.071	2.100	0.000E+00
AC116353.6	1.071	2.100	4.909E-08
HCFC1R1	1.071	2.102	0.000E+00
CARD6	1.071	2.102	4.514E-07
AL133520.1	1.072	2.103	4.372E-06
AL355297.3	1.073	2.103	1.226E-03
ASS1P1	1.073	2.103	1.899E-06
MACROD2	1.074	2.105	2.740E-03
FAHD1	1.076	2.108	0.000E+00
AC116651.1	1.076	2.109	1.533E-03
PYCARD-AS1	1.077	2.109	2.183E-06
AL596087.1	1.077	2.110	5.851E-05
RABAC1	1.077	2.110	8.304E-09
CD37	1.078	2.111	0.000E+00
CPLX1	1.078	2.111	3.802E-03
PLB1	1.078	2.112	7.348E-08
AP001505.1	1.078	2.112	3.551E-08
ADAP1	1.079	2.112	2.474E-08
AP006222.1	1.081	2.115	3.381E-04
KANK3	1.081	2.116	1.037E-07
LINC02062	1.082	2.116	1.765E-09
AC108488.3	1.082	2.117	5.124E-06
ZNF574	1.084	2.120	0.000E+00
SIGLEC9	1.084	2.120	0.000E+00
ABHD4	1.084	2.121	0.000E+00
RAPGEFL1	1.085	2.121	0.000E+00
PAK4	1.085	2.121	0.000E+00
AP003108.2	1.085	2.122	0.000E+00
PMEPA1	1.085	2.122	2.276E-09
FLVCR1-DT	1.086	2.123	8.399E-05
MGAT3	1.086	2.124	8.801E-07
IL1RN	1.087	2.124	3.147E-03
AC093788.1	1.087	2.124	1.293E-03
PSMF1	1.087	2.125	0.000E+00
LINC01004	1.088	2.125	0.000E+00
TPGS2	1.088	2.126	0.000E+00
APOL1	1.088	2.126	0.000E+00
AL133371.2	1.089	2.127	5.312E-05
AC108066.2	1.089	2.127	1.668E-03
AC010654.1	1.090	2.129	2.204E-05
ZNF236-DT	1.090	2.129	0.000E+00
PRSS36	1.091	2.130	2.049E-10
NABP1	1.091	2.130	6.040E-07
NACC1	1.092	2.132	0.000E+00
AL096701.4	1.092	2.132	5.223E-05
AC092428.1	1.092	2.132	7.399E-06
CHTF8	1.093	2.134	0.000E+00
CC2D1A	1.093	2.134	4.267E-04
SIAH2	1.094	2.134	0.000E+00
AP000919.1	1.095	2.136	6.086E-03
NCF2	1.095	2.136	2.733E-10
AC009237.14	1.096	2.137	2.795E-03
CCDC142	1.096	2.137	0.000E+00
AC100778.2	1.097	2.139	3.830E-05
CENPBD1	1.097	2.139	7.283E-10
EPN2	1.097	2.140	0.000E+00
TMEM60	1.097	2.140	2.737E-09
SIPA1	1.098	2.140	0.000E+00
DGCR2	1.099	2.143	0.000E+00
H3P6	1.102	2.147	1.164E-06
ZNF425	1.102	2.147	2.600E-07
AL132780.1	1.102	2.147	4.037E-05
CALML4	1.103	2.149	3.494E-07
COMTD1	1.104	2.149	1.978E-07
AC145423.2	1.104	2.150	6.571E-05
AC134407.2	1.104	2.150	1.395E-03
AL078600.1	1.105	2.150	1.729E-03
CSGALNACT2-DT	1.105	2.150	7.789E-06
RPP25L	1.105	2.151	4.298E-09
CABIN1	1.105	2.152	0.000E+00
KLHDC8B	1.106	2.152	1.831E-06
WNT10A	1.106	2.152	4.556E-09
H2BC5	1.106	2.153	6.652E-04
CLIC3	1.107	2.154	1.633E-05
DAPK3	1.107	2.154	0.000E+00

SLC25A44	1.109	2.156	0.000E+00
PDCD4-AS1	1.109	2.157	0.000E+00
MBLAC1	1.109	2.157	1.134E-05
IGHM	1.109	2.157	3.892E-05
TMEM238	1.109	2.157	2.204E-04
AC034236.2	1.109	2.157	1.580E-08
AC111000.4	1.109	2.157	3.183E-03
CD72	1.109	2.157	6.031E-09
ANPEP	1.111	2.159	3.075E-06
KDM4B	1.111	2.160	0.000E+00
SMPD5	1.111	2.160	7.910E-03
MAPRE3	1.111	2.160	0.000E+00
AC008966.1	1.111	2.160	2.372E-03
SCN4A	1.111	2.160	7.199E-05
ARHGAP25	1.112	2.161	0.000E+00
ZNF613	1.112	2.161	1.283E-09
TUBA3FP	1.113	2.163	2.488E-03
AC241585.2	1.113	2.163	1.381E-03
FAM174B	1.113	2.164	3.835E-08
HK2-DT	1.114	2.164	5.262E-06
IP6K1	1.114	2.164	0.000E+00
NUP50-DT	1.115	2.166	0.000E+00
CCDC106	1.116	2.167	1.444E-08
CCDC124	1.117	2.168	0.000E+00
ZEB1-AS1	1.117	2.168	0.000E+00
FLOT2	1.117	2.169	0.000E+00
PDGFB	1.117	2.169	2.657E-06
AC022968.1	1.117	2.169	8.474E-03
MIR3174	1.118	2.171	7.419E-03
AC004678.2	1.119	2.172	7.046E-03
TSSK6	1.119	2.172	6.798E-09
CEP170B	1.119	2.172	1.108E-05
AC125494.2	1.119	2.172	7.513E-08
ERF	1.120	2.173	0.000E+00
AC104958.2	1.120	2.173	0.000E+00
EPHB4	1.120	2.174	0.000E+00
AL118506.1	1.121	2.174	0.000E+00
TMEM169	1.121	2.175	5.587E-07
CDIPT	1.121	2.175	0.000E+00
ZNF579	1.122	2.176	7.704E-08
PROCA1	1.122	2.177	0.000E+00
UBALD2	1.122	2.177	3.471E-10
IL32	1.123	2.179	0.000E+00
AC004951.2	1.124	2.179	2.495E-09
ANKRD34A	1.124	2.180	0.000E+00
RAB33A	1.124	2.180	0.000E+00
FTLP3	1.124	2.180	1.148E-09
AC023157.2	1.125	2.180	1.482E-07
AC036176.1	1.125	2.181	4.024E-04
YIPF3	1.125	2.181	0.000E+00
ZNF576	1.125	2.181	0.000E+00
GIMAP4	1.125	2.181	7.687E-07
AC132938.5	1.125	2.181	6.492E-09
AC136475.7	1.126	2.183	7.934E-03
AP1M2	1.126	2.183	1.455E-03

POMC	1.127	2.183	1.188E-03
LINC02363	1.127	2.183	5.673E-05
ASCL2	1.127	2.185	1.347E-08
PLAAT5	1.128	2.185	1.655E-03
FAM117A	1.128	2.185	0.000E+00
ELMO3	1.129	2.187	2.082E-10
ATP6V0C	1.129	2.188	6.407E-09
AC009093.8	1.130	2.188	2.791E-06
AP004609.3	1.130	2.189	8.870E-08
MALAT1	1.130	2.189	0.000E+00
AC079331.2	1.131	2.190	9.040E-05
AGFG2	1.131	2.191	0.000E+00
RN7SL336P	1.132	2.191	5.621E-03
AC015971.1	1.132	2.191	4.719E-04
ZNF200	1.132	2.191	5.263E-09
MOCS3	1.132	2.192	0.000E+00
AC008875.1	1.133	2.192	6.021E-05
JAML	1.133	2.193	0.000E+00
CCDC17	1.133	2.193	0.000E+00
TACC3	1.133	2.193	0.000E+00
AL583722.1	1.134	2.194	1.732E-03
FASLG	1.134	2.195	5.604E-08
RNASEL	1.135	2.195	1.361E-09
AL604028.1	1.135	2.195	2.281E-08
AC007342.4	1.135	2.196	1.672E-04
MMACHC	1.135	2.196	4.301E-07
ANKRD9	1.136	2.197	5.975E-07
AC022211.3	1.136	2.198	4.740E-05
SAP25	1.136	2.198	1.799E-05
INKA1	1.136	2.198	1.366E-05
REEP2	1.137	2.199	1.170E-03
IFNGR2	1.137	2.199	3.403E-07
AP000926.2	1.137	2.199	2.705E-04
GALK1	1.137	2.200	4.481E-10
CCPG1	1.138	2.200	4.455E-08
MANEAL	1.138	2.201	1.418E-08
IGHG2	1.138	2.201	3.292E-03
ZNF396	1.139	2.202	6.314E-10
MAP2K2	1.139	2.202	0.000E+00
CLEC18B	1.139	2.203	3.413E-05
LYSMD2	1.140	2.203	0.000E+00
MARK2	1.140	2.204	0.000E+00
CNOT3	1.140	2.205	0.000E+00
AL390208.1	1.142	2.206	2.047E-03
PIP4P2	1.142	2.207	6.579E-10
TNIP1	1.142	2.207	0.000E+00
ZNF785	1.142	2.207	0.000E+00
AC007216.4	1.143	2.208	8.826E-03
PDF	1.143	2.209	7.914E-04
SPSB2	1.144	2.209	5.599E-09
NHLRC4	1.144	2.210	2.016E-07
AC009403.1	1.145	2.212	0.000E+00
KIAA2013	1.146	2.212	0.000E+00
AC007342.8	1.146	2.213	9.792E-05
FCHO1	1.146	2.213	0.000E+00

AC005520.5	1.146	2.213	3.434E-04
AC046185.3	1.146	2.214	0.000E+00
OPLAH	1.147	2.215	1.202E-06
BANP	1.147	2.215	0.000E+00
EVI2B	1.148	2.216	0.000E+00
TRBV4-1	1.148	2.216	1.997E-05
AC092868.1	1.148	2.217	1.179E-03
AP000763.3	1.149	2.217	2.551E-09
AC007598.3	1.149	2.218	6.299E-03
KLHDC7B	1.150	2.219	0.000E+00
ZNF672	1.151	2.220	0.000E+00
PDXP	1.151	2.220	3.682E-04
ACOX1	1.151	2.221	0.000E+00
RPGRIP1	1.151	2.221	1.814E-07
LINC00482	1.152	2.222	2.476E-05
SPRYD3	1.152	2.222	0.000E+00
DUSP19	1.152	2.222	2.444E-06
DLGAP3	1.152	2.223	3.474E-10
PTMAP4	1.153	2.223	0.000E+00
SPHK2	1.153	2.223	0.000E+00
MKNK2	1.153	2.223	0.000E+00
AL021707.5	1.153	2.224	3.854E-03
ARG1	1.153	2.225	2.876E-03
CHCHD2P6	1.154	2.225	2.998E-03
DTX1	1.154	2.225	2.210E-10
PTRH1	1.155	2.226	0.000E+00
PIAS4	1.155	2.228	0.000E+00
MIR4518	1.156	2.229	3.051E-03
P2RX6	1.157	2.229	5.111E-05
AL138820.1	1.157	2.230	2.622E-07
MEFV	1.158	2.231	1.499E-07
LAT2	1.159	2.233	0.000E+00
YIPF4	1.160	2.234	0.000E+00
CPNE5	1.160	2.234	3.607E-08
AP002358.1	1.161	2.235	2.967E-04
HYAL1	1.161	2.236	7.304E-06
PACSIN2	1.161	2.236	0.000E+00
SELL	1.161	2.237	0.000E+00
Z84480.1	1.161	2.237	6.268E-04
PAQR6	1.162	2.237	1.037E-07
LIN37	1.162	2.238	0.000E+00
RXRA	1.162	2.238	0.000E+00
AC022182.2	1.163	2.239	3.657E-04
LRFN3	1.163	2.239	0.000E+00
TREML3P	1.163	2.240	1.457E-03
PIP5KL1	1.164	2.241	3.966E-05
ARRB2	1.165	2.242	0.000E+00
EMC3	1.165	2.242	0.000E+00
PELO	1.165	2.242	0.000E+00
AC016957.1	1.165	2.242	6.437E-03
IRF2	1.166	2.243	0.000E+00
FAUP1	1.166	2.244	1.112E-04
RAB43	1.166	2.244	4.297E-07
ITPKB-IT1	1.166	2.245	8.969E-08
LINC02202	1.167	2.245	6.746E-03

AC002094.4	1.167	2.246	6.255E-04
AC130324.3	1.167	2.246	6.934E-05
EEF1A2	1.167	2.246	2.826E-03
SIRPA	1.168	2.248	0.000E+00
AC016727.1	1.168	2.248	0.000E+00
NR2F6	1.169	2.248	7.142E-08
CNR2	1.169	2.249	0.000E+00
ZNF296	1.170	2.250	7.101E-08
MGRN1	1.170	2.250	0.000E+00
POLD4	1.170	2.251	0.000E+00
KCNH2	1.171	2.252	0.000E+00
B9D2	1.172	2.253	0.000E+00
NAIPP1	1.172	2.254	7.283E-04
CD22	1.172	2.254	3.507E-10
TEKT4P2	1.173	2.254	9.099E-03
GIMAP1	1.173	2.255	0.000E+00
AL031709.1	1.174	2.256	6.924E-03
SOBP	1.174	2.256	1.735E-04
H1-3	1.174	2.256	7.709E-03
RAC2	1.174	2.256	0.000E+00
LRRC29	1.174	2.256	0.000E+00
PIGX	1.175	2.257	0.000E+00
ATP6V0D1	1.175	2.257	0.000E+00
AL121845.3	1.175	2.258	4.299E-09
EHD1	1.175	2.258	0.000E+00
SOD2	1.176	2.260	8.830E-05
ECM1	1.176	2.260	1.903E-07
MIB2	1.177	2.261	0.000E+00
CEACAM21	1.177	2.261	3.508E-07
KCNE1	1.177	2.262	2.149E-10
AC127024.5	1.178	2.262	4.330E-09
ABHD8	1.178	2.263	0.000E+00
SCX	1.178	2.263	1.150E-05
RASSF7	1.179	2.264	1.935E-10
AC011374.2	1.179	2.264	4.758E-08
AC009065.4	1.180	2.265	3.335E-08
TMED2-DT	1.180	2.266	0.000E+00
TAGLN2	1.181	2.267	0.000E+00
AL354920.1	1.181	2.267	7.949E-06
GMIP	1.182	2.269	0.000E+00
RAB1B	1.183	2.271	0.000E+00
PRDM5	1.183	2.271	6.792E-03
GPR18	1.185	2.274	0.000E+00
IRF2BP1	1.185	2.274	0.000E+00
PGAM1P11	1.186	2.275	5.520E-03
AL807752.6	1.186	2.275	1.360E-04
AL121583.1	1.186	2.275	3.041E-04
C17orf100	1.187	2.276	7.144E-07
WDR5-DT	1.188	2.278	9.267E-04
MIR29B2CHG	1.188	2.278	1.921E-07
AC007342.3	1.188	2.278	2.936E-04
AC011495.1	1.188	2.279	1.832E-04
CREB5	1.188	2.279	1.677E-07
TTC22	1.189	2.279	0.000E+00
CYTH4	1.189	2.280	0.000E+00

PTGES3P3	1.189	2.280	3.486E-03
PTPRN2	1.190	2.282	5.200E-10
C19orf71	1.190	2.282	0.000E+00
TMEM167B-DT	1.191	2.283	1.572E-07
ORAI2	1.192	2.284	0.000E+00
FUZ	1.192	2.284	0.000E+00
HLA-B	1.192	2.285	0.000E+00
AL592166.1	1.192	2.285	1.420E-05
H3P47	1.193	2.286	8.844E-05
FAM157C	1.193	2.286	4.929E-06
SNX32	1.193	2.287	8.637E-06
POLR1G	1.193	2.287	1.358E-07
PWWP2B	1.194	2.288	2.460E-09
SLC16A11	1.194	2.288	6.164E-06
TRIM21	1.194	2.288	9.603E-09
FLJ20021	1.195	2.289	5.379E-08
AC009318.4	1.195	2.290	3.578E-05
WDFY3-AS2	1.197	2.293	3.695E-04
KLHL21	1.197	2.293	0.000E+00
CLDND2	1.197	2.293	4.188E-10
HMBS	1.198	2.294	0.000E+00
OAZ1	1.198	2.295	0.000E+00
MIR6859-1	1.199	2.295	3.252E-09
ZNF646	1.200	2.298	0.000E+00
FHL3	1.201	2.299	0.000E+00
AC055839.2	1.202	2.300	0.000E+00
AC011468.1	1.202	2.301	1.772E-09
RHOG	1.203	2.302	0.000E+00
PYCARD	1.203	2.302	1.197E-10
AC087623.2	1.203	2.303	3.244E-04
RGL4	1.203	2.303	0.000E+00
MIR5194	1.205	2.305	8.073E-03
PPP1R3B	1.206	2.306	5.292E-04
ALDOA	1.207	2.308	0.000E+00
NALT1	1.208	2.309	2.883E-09
AL121820.3	1.208	2.310	1.008E-05
TMEM120A	1.208	2.311	0.000E+00
AC008569.2	1.209	2.311	3.028E-09
SMURF2P1	1.210	2.313	7.912E-03
PDCD1LG2	1.210	2.313	2.756E-04
AC063977.2	1.210	2.314	1.570E-07
DOHH	1.211	2.314	0.000E+00
RNASET2	1.211	2.314	0.000E+00
FCGRT	1.212	2.316	0.000E+00
PIK3R6	1.212	2.316	0.000E+00
ZBTB7B	1.212	2.316	0.000E+00
LILRB3	1.213	2.317	1.038E-08
PSPC1-AS2	1.214	2.319	2.807E-04
AC137630.4	1.214	2.320	2.311E-04
SEC14L1	1.214	2.320	6.065E-08
ZKSCAN2-DT	1.214	2.320	0.000E+00
MED16	1.214	2.320	0.000E+00
PHBP19	1.216	2.323	9.133E-05
PELATON	1.217	2.324	3.862E-10
CCDC97	1.217	2.324	0.000E+00
CEP295NL	1.217	2.324	5.125E-05
H1-2	1.217	2.324	3.116E-08
IFNL3P1	1.218	2.326	1.156E-03
PRKD2	1.218	2.326	0.000E+00
AC092135.3	1.218	2.327	7.983E-05
AC008676.1	1.219	2.328	8.990E-03
MAP6D1	1.219	2.328	1.568E-03
SIGLEC10	1.219	2.328	0.000E+00
PYGM	1.221	2.331	0.000E+00
LILRA6	1.221	2.332	3.020E-04
FNDC11	1.222	2.332	2.651E-08
AC104836.1	1.222	2.333	1.640E-03
RAB24	1.223	2.334	0.000E+00
CD2BP2-DT	1.225	2.337	8.853E-07
H2BC19P	1.226	2.339	0.000E+00
CHST7	1.226	2.339	0.000E+00
AC106782.5	1.227	2.340	9.518E-04
AC105020.5	1.227	2.341	4.511E-07
SLC9A3R1	1.227	2.341	0.000E+00
ST8SIA4	1.228	2.343	1.851E-10
LGALSL	1.229	2.344	1.739E-05
FOXD2	1.231	2.347	2.356E-06
CYP2T1P	1.231	2.348	4.065E-05
AP006287.2	1.231	2.348	8.363E-03
TCAP	1.231	2.348	0.000E+00
SERPING1	1.231	2.348	1.342E-04
LRTOMT	1.233	2.351	1.446E-08
AC099521.2	1.234	2.351	9.919E-06
AC010326.4	1.234	2.353	0.000E+00
KCNJ2-AS1	1.235	2.354	4.516E-03
PILRA	1.236	2.355	0.000E+00
OAZ2	1.236	2.356	0.000E+00
AC138150.2	1.236	2.356	6.204E-09
MAOA	1.237	2.356	5.066E-04
ZNF575	1.238	2.359	0.000E+00
LINC01311	1.239	2.361	2.513E-06
AC012368.2	1.239	2.361	2.040E-03
YBX1P2	1.239	2.361	2.416E-06
ELL	1.240	2.362	0.000E+00
GPR157	1.241	2.364	0.000E+00
OR52K1	1.243	2.366	5.630E-04
BIK	1.243	2.367	2.493E-08
TMEM253	1.244	2.369	6.218E-04
PNMT	1.244	2.369	1.112E-03
VIPR1-AS1	1.245	2.371	1.822E-03
CCR10	1.246	2.372	5.528E-05
RNPS1P1	1.246	2.372	1.423E-04
AL136141.1	1.247	2.374	4.725E-03
DENND3	1.248	2.375	0.000E+00
LRRC25	1.248	2.375	0.000E+00
NLRP9P1	1.248	2.375	9.388E-05
ZNF691	1.249	2.377	0.000E+00
HOMEZ	1.250	2.378	0.000E+00
AL590385.2	1.251	2.380	2.074E-03
DHRS12	1.251	2.381	0.000E+00

CCR8	1.252	2.382	7.098E-04
AC135050.3	1.252	2.382	3.098E-10
SLFN14	1.252	2.382	1.190E-04
RBM38	1.252	2.382	1.505E-07
PYGO2	1.253	2.383	0.000E+00
SAP30L-AS1	1.253	2.383	0.000E+00
ABCA7	1.253	2.384	0.000E+00
IL21R-AS1	1.253	2.384	7.658E-03
KLF2	1.254	2.385	0.000E+00
CALHM6	1.254	2.385	4.282E-05
AC006330.1	1.254	2.386	0.000E+00
AC096733.2	1.255	2.386	4.195E-06
ACTN1	1.255	2.386	0.000E+00
PNPLA1	1.256	2.388	1.239E-10
ZNHIT2	1.256	2.389	2.389E-07
KLK14	1.257	2.391	4.102E-08
DOK1	1.258	2.392	0.000E+00
TMEM81	1.259	2.393	1.978E-08
VMP1	1.259	2.394	1.743E-09
SLC22A18	1.259	2.394	0.000E+00
PRDX5	1.259	2.394	0.000E+00
RAB19	1.261	2.396	1.918E-09
MPP1	1.262	2.399	0.000E+00
BRWD1-AS2	1.264	2.401	1.455E-03
AC124319.1	1.264	2.402	1.262E-05
AL021707.8	1.264	2.402	0.000E+00
AC007620.2	1.265	2.404	2.789E-07
AC041005.1	1.267	2.407	1.403E-03
MBNL3	1.267	2.407	2.558E-10
SIRPB1	1.268	2.408	0.000E+00
LYSMD1	1.268	2.408	6.930E-10
AC007686.3	1.268	2.409	0.000E+00
NYAP1	1.268	2.409	1.224E-09
AL359878.1	1.268	2.409	1.896E-04
CARM1	1.269	2.409	0.000E+00
ORAI1	1.269	2.409	0.000E+00
AC073439.1	1.269	2.410	1.657E-04
TANGO2	1.269	2.411	0.000E+00
AC120053.1	1.270	2.411	0.000E+00
AC069281.2	1.270	2.411	0.000E+00
DOCK8-AS1	1.271	2.413	7.043E-10
HMGA1P8	1.272	2.415	5.597E-03
TBX6	1.273	2.416	0.000E+00
GATA3-AS1	1.273	2.416	1.004E-03
UBA52	1.273	2.417	0.000E+00
ITM2B	1.275	2.421	0.000E+00
BHLHE41	1.276	2.421	2.460E-04
JPT1	1.276	2.421	0.000E+00
MAST3	1.276	2.421	0.000E+00
EFHD2	1.276	2.422	0.000E+00
AC009005.1	1.276	2.422	6.532E-06
NEAT1	1.277	2.423	0.000E+00
AC120498.9	1.277	2.424	8.936E-05
AC012645.3	1.277	2.424	0.000E+00
AC093249.6	1.278	2.424	3.012E-08

RPS12P23	1.278	2.425	4.206E-03
RSPH9	1.279	2.426	8.312E-04
AP001057.1	1.279	2.426	6.913E-03
VPS37C	1.279	2.427	0.000E+00
AP005136.3	1.281	2.431	7.241E-06
IER5L	1.282	2.431	7.014E-05
PRAG1	1.282	2.432	0.000E+00
PGAP6	1.283	2.433	0.000E+00
CRB2	1.284	2.434	5.463E-03
AC008764.10	1.284	2.435	1.649E-03
AL049776.1	1.285	2.436	1.376E-04
DEF8	1.285	2.437	0.000E+00
CXCR6	1.285	2.437	2.820E-06
GBGT1	1.285	2.437	0.000E+00
GK	1.285	2.437	8.407E-07
AC020917.4	1.287	2.440	0.000E+00
SLC48A1	1.287	2.440	0.000E+00
TOLLIP	1.287	2.440	0.000E+00
APBB1IP	1.289	2.443	0.000E+00
AC020911.2	1.289	2.444	8.010E-03
SPNS3	1.290	2.445	0.000E+00
AC111182.1	1.291	2.447	2.319E-08
AL031775.1	1.292	2.448	0.000E+00
AC004596.1	1.292	2.449	7.236E-07
ST6GALNAC4P1	1.292	2.449	2.353E-04
ENKD1	1.293	2.451	0.000E+00
SHISA5	1.293	2.451	0.000E+00
AL096865.1	1.293	2.451	0.000E+00
FAM166B	1.294	2.452	1.453E-03
AL161781.1	1.295	2.453	1.000E-05
IL6R	1.295	2.453	0.000E+00
TFE3	1.296	2.455	0.000E+00
LINC01134	1.296	2.456	2.012E-07
LINC01715	1.297	2.457	2.957E-03
AC012184.1	1.297	2.458	4.882E-06
LINC01819	1.298	2.458	5.706E-05
SNAI3-AS1	1.298	2.459	0.000E+00
FAXDC2	1.299	2.461	6.724E-07
TRIM69	1.303	2.467	0.000E+00
CD79A	1.303	2.467	3.876E-09
SEMA4B	1.303	2.467	0.000E+00
AC010335.3	1.304	2.468	4.479E-03
ZNF746	1.304	2.470	0.000E+00
NRN1	1.304	2.470	5.069E-04
NLRX1	1.305	2.471	0.000E+00
THAP8	1.306	2.472	0.000E+00
C19orf73	1.306	2.473	2.622E-05
MAP7	1.307	2.475	9.097E-09
TMEM204	1.307	2.475	6.577E-10
FGD3	1.308	2.475	0.000E+00
PRR22	1.308	2.475	2.395E-09
NLGN3	1.308	2.475	0.000E+00
STOX1	1.308	2.476	2.764E-03
SH3D21	1.308	2.476	0.000E+00
ZC3H3	1.308	2.477	0.000E+00

ATP1B2	1.309	2.477	4.957E-04
DLK2	1.310	2.479	1.804E-03
GPR68	1.311	2.482	0.000E+00
AC007224.2	1.313	2.485	6.685E-07
AJ003147.3	1.314	2.486	0.000E+00
UBTD1	1.317	2.491	1.200E-10
SLC29A1	1.318	2.493	0.000E+00
RAB11B	1.318	2.493	0.000E+00
RNU6-762P	1.319	2.494	1.732E-03
AC005786.3	1.319	2.495	0.000E+00
LRRC46	1.319	2.495	2.296E-08
CCDC153	1.320	2.496	2.608E-09
AC215522.2	1.320	2.497	7.352E-07
TRBJ2-4	1.320	2.498	1.017E-03
AC016027.1	1.323	2.501	1.182E-06
AL109809.1	1.324	2.504	1.742E-04
RN7SL812P	1.324	2.504	1.489E-03
GNB2	1.325	2.505	0.000E+00
BRICD5	1.325	2.505	0.000E+00
AC005220.1	1.325	2.505	6.334E-04
MFSD6L	1.325	2.505	1.643E-07
PLEKHO2	1.327	2.508	0.000E+00
AC004263.2	1.327	2.509	1.617E-04
NPL	1.328	2.511	0.000E+00
KRT18P4	1.329	2.512	2.656E-04
FCER2	1.329	2.513	0.000E+00
AC009133.1	1.331	2.515	0.000E+00
TTC23L-AS1	1.331	2.515	6.194E-04
MIR3677	1.331	2.516	7.927E-03
LINC02362	1.331	2.516	3.469E-06
AC022007.1	1.332	2.517	1.407E-04
EIF2S2P3	1.333	2.519	3.262E-05
SND1-IT1	1.334	2.520	3.349E-03
PLEKHG3	1.334	2.520	0.000E+00
PRAF2	1.334	2.521	0.000E+00
ISCA1P1	1.335	2.523	8.685E-03
ETV7	1.335	2.524	4.046E-05
ATP2C2	1.336	2.524	3.504E-04
PLEKHF1	1.336	2.525	0.000E+00
IRF2BPL	1.336	2.525	0.000E+00
UBR5-AS1	1.337	2.526	0.000E+00
LINC00638	1.337	2.527	2.535E-06
CXCR5	1.339	2.529	0.000E+00
AL109804.1	1.339	2.530	2.124E-03
AGAP2-AS1	1.340	2.531	7.259E-04
LINC00412	1.340	2.531	2.752E-03
SH2D3C	1.340	2.532	0.000E+00
PEX12	1.340	2.532	0.000E+00
AL589765.4	1.340	2.532	6.173E-07
AC005840.2	1.341	2.533	1.560E-09
C9orf78	1.341	2.533	0.000E+00
SHKBP1	1.341	2.533	0.000E+00
NLRP12	1.343	2.536	4.826E-10
LCMT2	1.343	2.536	0.000E+00
ORAI3	1.345	2.540	0.000E+00

MMP23B	1.346	2.541	1.315E-04
UBBP4	1.346	2.541	1.563E-03
OPRL1	1.346	2.541	0.000E+00
NKX3-1	1.346	2.543	4.376E-03
AP000240.1	1.346	2.543	7.744E-06
MAP3K11	1.347	2.543	0.000E+00
HLA-A	1.347	2.543	2.895E-04
MIR93	1.347	2.543	2.256E-03
MTG2	1.347	2.544	0.000E+00
APOL2	1.347	2.544	0.000E+00
AC087289.5	1.347	2.544	1.923E-03
FGF22	1.348	2.545	4.029E-04
SNAI3	1.348	2.546	0.000E+00
AC020636.1	1.349	2.547	9.977E-03
TLR6	1.351	2.550	2.697E-09
AC110769.2	1.351	2.550	9.132E-05
AC040934.1	1.352	2.552	2.637E-03
PRRT3-AS1	1.354	2.555	2.143E-05
ZNF696	1.354	2.555	0.000E+00
RPP25	1.354	2.557	1.073E-09
AL031777.1	1.356	2.559	1.179E-03
AC079174.2	1.358	2.564	0.000E+00
AP006621.6	1.359	2.565	4.724E-07
LRRC6	1.360	2.567	1.102E-04
NQO2	1.360	2.567	2.307E-10
CADM4	1.360	2.568	0.000E+00
KRT18P5	1.362	2.570	1.740E-07
TRBV10-2	1.362	2.570	7.810E-04
AC020703.1	1.363	2.572	8.646E-05
SMIM5	1.363	2.573	1.782E-08
AC007342.7	1.363	2.573	4.034E-08
FCGR1CP	1.364	2.575	2.050E-05
AC021097.1	1.365	2.575	0.000E+00
ITGAD	1.365	2.576	2.940E-06
LINC00896	1.366	2.577	4.207E-06
AC018695.6	1.366	2.578	6.919E-06
AL359762.3	1.366	2.578	6.217E-09
AC023389.2	1.367	2.579	1.389E-03
XPO6	1.367	2.579	0.000E+00
AC015912.3	1.367	2.579	1.392E-05
AL731569.1	1.367	2.580	0.000E+00
FAM83E	1.368	2.581	2.222E-03
PISD	1.368	2.581	0.000E+00
ERFE	1.369	2.583	2.174E-03
CEBPB	1.369	2.584	0.000E+00
AC018809.2	1.371	2.587	6.568E-09
SLC12A9	1.372	2.587	0.000E+00
AC036176.2	1.372	2.588	4.040E-03
XBP1P1	1.372	2.589	3.126E-03
DDIT4L	1.374	2.591	7.415E-03
AP005482.2	1.374	2.591	5.058E-07
PGLYRP2	1.374	2.592	9.842E-03
ZNF837	1.374	2.592	4.639E-09
TGFBR3L	1.378	2.598	1.080E-03
UBE2C	1.379	2.600	2.676E-07

LINC02656	1.380	2.602	2.272E-10
FBXL18	1.380	2.602	0.000E+00
KBTBD7	1.382	2.607	4.365E-07
FO393419.3	1.383	2.608	3.438E-07
AC099791.2	1.383	2.608	7.740E-03
ALOX5AP	1.383	2.609	0.000E+00
PHRF1	1.384	2.610	0.000E+00
SCAF1	1.384	2.610	0.000E+00
AC012236.1	1.386	2.614	3.261E-03
ALPK1	1.387	2.616	0.000E+00
SIRPB2	1.388	2.617	0.000E+00
INAFM1	1.388	2.617	0.000E+00
AC067852.2	1.389	2.618	0.000E+00
AL512652.1	1.389	2.619	2.263E-03
ZBTB42	1.389	2.619	0.000E+00
AC007823.1	1.389	2.620	2.473E-03
SLC23A3	1.390	2.620	0.000E+00
RPL13P12	1.390	2.621	1.638E-03
TRIM47	1.391	2.622	0.000E+00
MAP11	1.391	2.622	0.000E+00
SH3BP1	1.391	2.623	0.000E+00
BORCS8	1.392	2.624	0.000E+00
CD82	1.392	2.625	0.000E+00
BTBD6P1	1.393	2.626	4.409E-07
AL133517.1	1.393	2.626	1.742E-05
DNAJB2	1.393	2.627	0.000E+00
AC044787.1	1.394	2.627	2.142E-09
AC025287.3	1.394	2.627	1.349E-05
CALHM1	1.394	2.628	1.279E-03
PPP1R3D	1.394	2.628	0.000E+00
LINC01480	1.394	2.629	4.245E-04
MID1IP1	1.397	2.634	0.000E+00
LINC00921	1.398	2.636	0.000E+00
ZNF653	1.398	2.636	0.000E+00
EPOR	1.399	2.637	0.000E+00
CX3CR1	1.400	2.640	1.334E-06
AC024257.3	1.400	2.640	0.000E+00
AC008758.4	1.400	2.640	6.025E-03
AC117382.1	1.401	2.641	1.949E-04
CCDC85B	1.401	2.641	1.026E-05
HSD3B7	1.401	2.642	0.000E+00
AC011466.4	1.402	2.642	6.583E-06
FIGNL2	1.402	2.642	0.000E+00
TRIM9	1.403	2.644	4.475E-07
AC026803.1	1.403	2.644	3.480E-04
AC092171.5	1.403	2.645	3.353E-04
IMPDH1	1.404	2.646	0.000E+00
MYO1F	1.404	2.646	0.000E+00
HOTAIRM1	1.405	2.648	0.000E+00
PRR5	1.406	2.649	0.000E+00
AC018926.3	1.407	2.653	3.599E-07
AC005833.2	1.410	2.657	9.852E-03
AC100814.1	1.410	2.657	4.544E-04
AP001160.2	1.410	2.658	7.570E-03
LARGE2	1.411	2.658	0.000E+00
CEBPA-DT	1.412	2.661	8.555E-09
SAC3D1	1.412	2.662	0.000E+00
AL161729.4	1.413	2.663	1.379E-03
TBXA2R	1.414	2.664	2.328E-10
RNF208	1.414	2.665	2.015E-07
STK24-AS1	1.415	2.667	2.778E-04
AL031123.2	1.415	2.667	9.365E-04
ZER1	1.416	2.668	0.000E+00
PTPRN	1.416	2.668	8.857E-04
IFIT3	1.417	2.670	5.333E-03
LTBP2	1.417	2.670	0.000E+00
AK8	1.417	2.671	3.077E-04
CANT1	1.417	2.671	0.000E+00
MMP23A	1.419	2.674	8.118E-03
ACSL1	1.421	2.677	3.718E-06
AC005841.1	1.422	2.679	3.798E-03
SEMA3B	1.422	2.680	2.009E-08
C5AR1	1.423	2.681	0.000E+00
RFLNB	1.423	2.681	9.200E-06
AL442128.2	1.423	2.681	1.504E-09
H2AZ2P1	1.423	2.681	3.889E-07
NXN	1.423	2.682	5.585E-04
C19orf25	1.424	2.682	0.000E+00
CNKSR3	1.424	2.683	8.780E-04
MAP2K3	1.424	2.683	0.000E+00
AC116667.1	1.424	2.684	6.636E-07
AC021016.2	1.426	2.686	2.826E-08
BCL6	1.426	2.688	1.247E-09
AC092809.4	1.427	2.689	4.951E-03
MAP1S	1.428	2.690	0.000E+00
NINJ1	1.428	2.691	0.000E+00
ZBED8	1.428	2.691	9.709E-07
EVA1B	1.428	2.692	2.000E-06
EEPD1	1.429	2.693	0.000E+00
IL1R1	1.430	2.694	6.979E-07
AC064847.1	1.430	2.694	6.080E-03
HVCN1	1.430	2.695	0.000E+00
AC012511.1	1.430	2.695	1.550E-03
AL080317.1	1.431	2.695	1.352E-05
TST	1.431	2.695	0.000E+00
C9orf40	1.431	2.697	0.000E+00
AC016571.1	1.432	2.698	6.121E-04
AC010531.6	1.433	2.699	1.336E-03
AC005837.1	1.433	2.700	2.956E-04
CAMTA1-DT	1.433	2.700	2.897E-05
TEAD3	1.433	2.701	2.224E-03
ST6GALNAC6	1.433	2.701	0.000E+00
ATP2A1	1.434	2.702	4.458E-09
CDC25C	1.434	2.702	1.180E-03
SORD2P	1.434	2.702	1.759E-04
PPIAP29	1.435	2.704	6.472E-10
RARA	1.436	2.705	0.000E+00
AC034102.6	1.436	2.706	3.176E-07
ZNF787	1.436	2.706	0.000E+00
AC024257.2	1.437	2.707	3.187E-03

POLL	1.441	2.715	0.000E+00
CASS4	1.442	2.716	0.000E+00
TMEM154	1.442	2.716	3.494E-10
EXOC3L1	1.442	2.716	4.397E-05
AP000866.5	1.442	2.717	1.253E-08
OSER1-DT	1.442	2.718	1.294E-09
ARL11	1.443	2.719	0.000E+00
VIT	1.444	2.721	5.605E-04
P2RY14	1.444	2.722	1.833E-06
GJC2	1.445	2.723	1.458E-06
H1-10-AS1	1.446	2.725	0.000E+00
BX539320.1	1.447	2.726	0.000E+00
NADK	1.447	2.726	0.000E+00
Z94721.3	1.448	2.728	3.106E-06
AC079336.5	1.450	2.732	3.778E-05
GAPT	1.451	2.735	0.000E+00
DEGS2	1.452	2.735	3.320E-07
DOK3	1.453	2.737	0.000E+00
LLPH-DT	1.455	2.741	1.819E-03
NAGS	1.455	2.742	0.000E+00
GNG10	1.456	2.743	8.841E-07
RN7SL239P	1.456	2.744	7.118E-03
C11orf68	1.458	2.747	0.000E+00
TMEM91	1.459	2.748	0.000E+00
LRRC61	1.460	2.751	4.015E-08
CHST13	1.460	2.751	9.135E-07
AC253576.2	1.460	2.751	1.593E-03
GOLGA5P1	1.461	2.752	4.538E-09
AC010343.1	1.462	2.754	0.000E+00
ADM2	1.462	2.754	2.064E-09
AL591043.2	1.464	2.758	3.353E-04
TUBB4A	1.464	2.759	2.631E-09
AC010359.3	1.465	2.760	6.459E-06
DPEP2	1.465	2.761	0.000E+00
SULT1A2	1.465	2.762	7.140E-05
ARHGAP9	1.467	2.765	0.000E+00
AC092117.1	1.468	2.766	3.805E-10
ZDHHC12	1.468	2.766	0.000E+00
ZNF2	1.469	2.768	2.911E-07
H2AX	1.469	2.768	0.000E+00
LINC02809	1.469	2.769	1.143E-03
TLCD4	1.469	2.769	9.556E-07
IMPA2	1.470	2.770	0.000E+00
VNN3	1.470	2.770	2.458E-07
AL117335.1	1.471	2.772	0.000E+00
AC104561.4	1.472	2.774	9.501E-04
TMEM187	1.472	2.775	0.000E+00
AP001160.4	1.475	2.779	4.377E-06
CD180	1.475	2.779	2.286E-09
FOXO4	1.475	2.779	0.000E+00
HCK	1.475	2.780	0.000E+00
KCNE3	1.475	2.780	0.000E+00
RFX2	1.476	2.781	0.000E+00
CLEC18A	1.476	2.782	4.742E-08
B3GNT9	1.477	2.783	0.000E+00
OR2T8	1.477	2.784	2.529E-03
ZNF775	1.477	2.784	0.000E+00
AC087294.1	1.478	2.785	5.722E-10
AL049780.1	1.478	2.786	2.492E-03
AC136475.9	1.479	2.788	8.130E-03
TMEM158	1.480	2.789	2.521E-05
TLR10	1.480	2.790	1.929E-07
REPS2	1.481	2.791	1.509E-10
IGLC5	1.481	2.792	8.491E-04
AC023055.1	1.482	2.793	1.898E-03
ADAT3	1.482	2.793	6.215E-09
AL627171.1	1.482	2.793	7.186E-05
AC018755.3	1.483	2.795	1.087E-05
AL139123.1	1.484	2.796	1.617E-07
NIBAN1	1.485	2.799	2.133E-07
SSH3	1.485	2.799	0.000E+00
AC011462.4	1.485	2.799	0.000E+00
ZNNT1	1.485	2.800	2.507E-10
YJEFN3	1.485	2.800	0.000E+00
AC091133.6	1.486	2.801	4.725E-03
GCAT	1.486	2.801	4.119E-09
GCA	1.486	2.802	1.055E-07
AC007569.1	1.486	2.802	8.330E-10
RGS19	1.488	2.804	0.000E+00
ZBTB45	1.488	2.804	0.000E+00
AC004223.2	1.489	2.807	1.407E-03
ARMC7	1.490	2.809	0.000E+00
ZNF835	1.491	2.812	1.106E-09
APOBEC3B	1.492	2.813	1.545E-03
AC126474.2	1.492	2.813	0.000E+00
SSPN	1.492	2.813	2.388E-06
ZNF683	1.493	2.814	1.292E-05
AC002467.1	1.493	2.815	1.131E-09
PHF23	1.493	2.816	0.000E+00
AC083862.1	1.494	2.816	0.000E+00
UGT2B17	1.495	2.819	3.056E-03
AL391095.3	1.495	2.819	6.241E-04
FAM3D	1.495	2.819	7.282E-03
PDXP-DT	1.497	2.822	1.516E-04
AC003072.1	1.497	2.823	0.000E+00
SPAG6	1.497	2.823	4.348E-03
AL121890.2	1.499	2.826	8.882E-04
CDK2AP2	1.499	2.827	0.000E+00
AL353708.3	1.501	2.830	0.000E+00
AC022211.4	1.501	2.830	2.097E-06
TMEM121	1.502	2.833	6.381E-07
ITGAX	1.502	2.833	0.000E+00
RPL7AP6	1.502	2.833	0.000E+00
CYBC1	1.503	2.833	0.000E+00
AC092910.3	1.503	2.834	0.000E+00
RN7SKP160	1.504	2.837	9.876E-03
Z92544.1	1.505	2.838	0.000E+00
KCTD21	1.505	2.838	0.000E+00
DTX2	1.505	2.839	0.000E+00
TBC1D3L	1.506	2.839	6.743E-05

RERE-AS1	1.506	2.841	0.000E+00
MPZL1	1.506	2.841	9.038E-09
Z84466.1	1.507	2.842	3.217E-03
AC016876.3	1.509	2.846	6.759E-03
GSN	1.509	2.847	0.000E+00
MIR766	1.510	2.848	3.950E-03
DTX2P1	1.511	2.850	6.303E-06
PYGL	1.514	2.855	0.000E+00
KLHL31	1.515	2.857	7.761E-04
FAM71E1	1.515	2.859	8.270E-03
C1orf220	1.516	2.860	2.427E-07
CCDC121	1.517	2.862	1.087E-05
FAM157B	1.517	2.863	9.077E-08
MOB3A	1.517	2.863	0.000E+00
STK40	1.518	2.863	0.000E+00
WNT9A	1.518	2.864	1.226E-04
SELPLG	1.518	2.864	0.000E+00
SLC2A1-AS1	1.521	2.869	1.240E-10
NINJ2	1.521	2.871	0.000E+00
AC091045.1	1.521	2.871	1.822E-03
DBNDD1	1.522	2.872	2.206E-10
ADRB1	1.523	2.875	1.749E-03
FAM50B	1.525	2.879	0.000E+00
TSC22D4	1.526	2.880	0.000E+00
PROC	1.526	2.880	5.689E-07
AP000944.5	1.527	2.881	5.850E-07
RNU6-610P	1.527	2.881	2.421E-03
WBP2	1.528	2.883	0.000E+00
APOBEC3A	1.528	2.884	1.847E-10
RNF122	1.528	2.884	0.000E+00
LITAF	1.532	2.891	0.000E+00
SNAP25	1.532	2.892	8.063E-03
QPCT	1.532	2.893	0.000E+00
AGER	1.533	2.893	1.135E-04
ZBTB3	1.533	2.894	0.000E+00
SLC22A4	1.534	2.895	1.505E-10
AC006504.7	1.535	2.897	1.110E-08
SLC51A	1.535	2.898	4.853E-04
ASCC2	1.538	2.905	0.000E+00
MYO7B	1.539	2.906	0.000E+00
ANO7L1	1.541	2.911	2.459E-10
AC004832.4	1.542	2.911	8.633E-03
PLPPR2	1.543	2.914	0.000E+00
ST3GAL4	1.543	2.915	0.000E+00
AL356740.1	1.544	2.917	8.084E-04
RGS14	1.546	2.919	0.000E+00
PFKFB4	1.546	2.920	0.000E+00
PRR13P2	1.547	2.922	2.337E-03
MT1L	1.547	2.923	3.526E-03
AL731563.3	1.548	2.923	0.000E+00
NRBF2	1.548	2.924	9.345E-10
GTSE1-DT	1.548	2.924	7.782E-07
AC018521.7	1.548	2.925	2.437E-03
NPB	1.549	2.925	1.187E-03
ST3GAL2	1.550	2.929	0.000E+00
LINC01762	1.551	2.931	5.516E-09
APOBR	1.552	2.932	0.000E+00
HDAC10	1.554	2.937	0.000E+00
TBKBP1	1.556	2.941	0.000E+00
IFNL1	1.556	2.941	8.945E-04
CBFA2T3	1.557	2.942	0.000E+00
LIMS2	1.558	2.945	0.000E+00
NOL12	1.559	2.946	0.000E+00
AP000763.4	1.559	2.946	3.993E-03
AC006538.3	1.560	2.949	9.758E-04
HSPB1	1.561	2.950	0.000E+00
LY6E-DT	1.563	2.955	4.058E-05
PTEN	1.564	2.957	3.946E-07
Z94721.1	1.565	2.958	0.000E+00
SCARNA21	1.566	2.961	2.061E-08
PPCDC	1.566	2.961	0.000E+00
CDH2	1.567	2.962	1.292E-04
TALDO1	1.567	2.963	0.000E+00
TOR4A	1.569	2.966	0.000E+00
PLA2G2D	1.570	2.969	7.175E-05
AL162458.1	1.571	2.970	0.000E+00
MIR4489	1.571	2.971	7.739E-10
GBP6	1.571	2.971	4.114E-07
RIMBP3B	1.571	2.972	6.992E-03
AC007292.2	1.571	2.972	0.000E+00
MORC2-AS1	1.572	2.973	2.408E-07
AL139243.1	1.573	2.975	5.348E-05
PHLDA3	1.573	2.976	2.311E-07
AP000346.2	1.573	2.976	0.000E+00
CAP1P1	1.574	2.978	2.148E-10
FAM174A	1.574	2.978	0.000E+00
AC010883.3	1.576	2.982	7.082E-05
AL357033.4	1.577	2.984	0.000E+00
NTNG2	1.579	2.987	0.000E+00
HCN2	1.579	2.988	0.000E+00
F12	1.579	2.989	0.000E+00
RNF10	1.581	2.991	0.000E+00
AC008850.1	1.581	2.993	1.570E-03
AC018638.7	1.582	2.994	0.000E+00
GLT1D1	1.582	2.995	0.000E+00
SH3BP5L	1.583	2.995	0.000E+00
AC127024.6	1.583	2.995	3.795E-04
RHOXF1P2	1.583	2.997	4.038E-04
NSMCE1-DT	1.584	2.997	1.129E-09
MBOAT2	1.585	3.000	0.000E+00
TGFA	1.585	3.001	2.058E-08
GRAMD1C	1.586	3.001	1.878E-08
AC011466.1	1.586	3.003	6.814E-03
BORCS6	1.587	3.003	0.000E+00
GGT5	1.587	3.005	5.559E-05
ITCH-IT1	1.588	3.007	4.244E-03
RNU6-33P	1.588	3.007	9.045E-03
LPAR5	1.589	3.007	0.000E+00
ADAM8	1.589	3.008	0.000E+00
AC027307.2	1.589	3.008	0.000E+00

BMERB1	1.590	3.010	2.673E-03
TNFRSF9	1.590	3.011	0.000E+00
IGLJ3	1.590	3.011	1.396E-03
MBOAT7	1.591	3.012	6.672E-04
YPEL4	1.591	3.012	1.857E-10
MRM1	1.591	3.012	1.163E-05
AC002456.1	1.591	3.012	2.059E-07
AL354977.2	1.591	3.013	3.881E-04
MYL12BP1	1.591	3.014	6.580E-04
MIRLET7G	1.592	3.015	6.046E-03
HES7	1.593	3.017	1.229E-05
AC012213.3	1.594	3.018	2.278E-03
AC011676.1	1.596	3.023	8.429E-05
AC005696.1	1.596	3.023	5.096E-05
C1orf56	1.596	3.024	0.000E+00
TMEM121B	1.598	3.027	0.000E+00
AC145285.6	1.599	3.030	5.305E-08
AC008622.2	1.601	3.034	1.580E-08
TFEB	1.601	3.034	0.000E+00
MYPOP	1.602	3.035	0.000E+00
PPP1R14BP3	1.602	3.036	0.000E+00
ZNF524	1.603	3.037	0.000E+00
FGF13	1.603	3.038	4.969E-03
AC007342.5	1.603	3.039	4.076E-08
UBALD1	1.603	3.039	0.000E+00
CA15P1	1.604	3.039	0.000E+00
IKBIP	1.604	3.039	0.000E+00
AP000759.1	1.604	3.040	6.054E-03
KDM7A-DT	1.605	3.041	0.000E+00
AL356752.1	1.605	3.043	4.863E-03
AC025031.5	1.606	3.043	1.951E-05
MXD3	1.606	3.044	0.000E+00
AC135803.1	1.609	3.050	1.095E-03
AC004241.2	1.609	3.051	1.554E-07
AC112496.1	1.609	3.051	1.326E-07
Z97832.2	1.610	3.052	0.000E+00
C16orf54	1.611	3.054	0.000E+00
MBD6	1.611	3.054	0.000E+00
AC004678.1	1.611	3.054	1.292E-05
CLEC9A	1.611	3.055	2.189E-05
POU5F1P5	1.612	3.058	3.317E-03
IFITM3	1.613	3.059	5.371E-06
ARF1P2	1.614	3.061	6.920E-03
WNK2	1.615	3.063	2.367E-06
AC008738.5	1.615	3.063	4.448E-04
MINDY1	1.615	3.064	0.000E+00
AC016168.3	1.615	3.064	1.005E-03
DPM2	1.616	3.065	0.000E+00
DYRK3	1.616	3.065	1.924E-09
RNU1-134P	1.616	3.066	2.457E-04
DHRS13	1.617	3.067	0.000E+00
AC040904.1	1.618	3.069	7.083E-04
ATG16L2	1.619	3.071	0.000E+00
AP000845.1	1.620	3.074	1.143E-03
RNA5SP207	1.621	3.075	2.881E-03

C2CD4D	1.622	3.078	5.309E-05
MIR548AA2	1.622	3.078	4.408E-03
XKR8	1.623	3.080	0.000E+00
SNX22	1.623	3.080	0.000E+00
AC136475.4	1.624	3.082	1.556E-03
AC068025.2	1.625	3.084	2.264E-03
UFSP1	1.627	3.088	0.000E+00
STX3	1.628	3.091	0.000E+00
AL158071.4	1.630	3.094	6.110E-03
MPZL3	1.630	3.095	0.000E+00
AL022316.1	1.630	3.096	8.266E-06
DBN1	1.631	3.096	0.000E+00
AC040160.2	1.631	3.098	0.000E+00
LINC02166	1.631	3.098	1.386E-06
AL136441.1	1.631	3.098	9.285E-04
PTAFR	1.632	3.099	0.000E+00
HAL	1.632	3.100	0.000E+00
FECH	1.632	3.100	0.000E+00
DRC1	1.633	3.101	6.325E-03
GADD45G	1.633	3.101	6.121E-10
DNAJC3-DT	1.634	3.104	0.000E+00
MYBPC3	1.634	3.104	0.000E+00
AC020656.2	1.635	3.105	1.160E-07
MST1R	1.635	3.105	7.405E-05
AC061992.2	1.635	3.107	6.525E-04
WAS	1.636	3.107	0.000E+00
S100A11	1.636	3.108	0.000E+00
SLC22A14	1.637	3.109	3.390E-05
HLA-C	1.637	3.110	4.343E-10
AC009061.2	1.639	3.113	0.000E+00
C20orf204	1.639	3.115	4.212E-08
BEAN1-AS1	1.640	3.116	2.532E-08
ABHD17AP4	1.640	3.116	1.366E-03
SENCR	1.641	3.119	0.000E+00
VPREB3	1.641	3.119	6.324E-09
PDZD3	1.641	3.120	2.098E-07
AP000351.2	1.642	3.120	8.885E-05
TMEM86B	1.643	3.123	0.000E+00
FLJ38576	1.644	3.125	3.490E-04
AC018638.6	1.645	3.128	2.707E-09
BBC3	1.646	3.129	0.000E+00
UPK3B	1.646	3.129	5.262E-09
MXD1	1.646	3.130	1.183E-08
Z82188.2	1.647	3.131	3.512E-05
NUAK2	1.647	3.133	0.000E+00
GDPD3	1.647	3.133	0.000E+00
ALDH1A2	1.648	3.133	6.968E-06
UPB1	1.648	3.134	0.000E+00
ADAMTS7P1	1.649	3.137	1.418E-07
RPL12P27	1.650	3.138	1.410E-06
IRF7	1.651	3.140	5.083E-09
SPI1	1.652	3.142	0.000E+00
KCNE5	1.652	3.144	1.579E-06
CRYBB3	1.653	3.144	8.669E-03
LINC02132	1.653	3.145	4.355E-08

CNN2	1.654	3.146	0.000E+00
AC092384.1	1.654	3.147	3.004E-09
C9orf139	1.655	3.149	0.000E+00
TOB1-AS1	1.655	3.149	0.000E+00
SCARNA9	1.655	3.150	1.465E-10
AC006141.1	1.655	3.150	1.691E-04
RNU6-920P	1.656	3.152	9.572E-08
AL135838.1	1.657	3.154	4.549E-03
TUBBP5	1.658	3.155	4.438E-04
AC109347.2	1.659	3.157	9.781E-04
AC084781.1	1.660	3.160	7.742E-03
AL121832.2	1.660	3.161	0.000E+00
IGF2BP2	1.662	3.165	0.000E+00
U62317.3	1.662	3.166	2.747E-07
MIR106B	1.663	3.167	3.721E-05
SPATA32	1.664	3.169	3.922E-04
PHC2	1.665	3.171	0.000E+00
SHARPIN	1.666	3.172	0.000E+00
CKLF-CMTM1	1.666	3.173	1.170E-06
TMEM45B	1.666	3.173	0.000E+00
AC010203.2	1.668	3.177	1.293E-03
ZSWIM3	1.668	3.178	0.000E+00
HECW2	1.669	3.180	1.673E-07
AP000944.7	1.669	3.180	1.288E-09
AL078604.2	1.671	3.185	5.717E-05
AL133415.1	1.673	3.188	1.372E-04
WLS	1.673	3.189	1.038E-10
PDE6H	1.673	3.189	2.708E-04
LINC01918	1.674	3.191	1.109E-03
DOK4	1.675	3.193	0.000E+00
AC092127.1	1.678	3.199	8.443E-08
AC127521.1	1.678	3.199	0.000E+00
DYSF	1.678	3.199	1.453E-10
LINC02680	1.678	3.200	1.273E-08
PPDPF	1.679	3.202	0.000E+00
SLC38A5	1.679	3.203	0.000E+00
AC007546.3	1.680	3.205	2.768E-06
TCL1A	1.681	3.206	3.355E-08
SMC2-AS1	1.681	3.206	7.891E-04
DOK7	1.682	3.208	5.965E-08
MEF2B	1.682	3.209	0.000E+00
AL158212.2	1.682	3.209	1.396E-10
GPR27	1.682	3.209	9.006E-08
TMEM250	1.684	3.213	0.000E+00
LFNG	1.685	3.216	0.000E+00
TCTEX1D4	1.687	3.219	3.254E-05
LINC00671	1.688	3.221	4.493E-10
AL021707.1	1.690	3.227	0.000E+00
CDC42EP2	1.690	3.227	2.431E-04
CEBPD	1.691	3.230	0.000E+00
AC009403.2	1.691	3.230	2.302E-04
AC069528.2	1.692	3.230	0.000E+00
AL162591.2	1.692	3.230	3.676E-05
AC087289.2	1.692	3.231	2.785E-03
AC027682.4	1.692	3.232	1.008E-10

TP53I11	1.693	3.233	0.000E+00
CSF2RB	1.693	3.233	1.009E-10
FCGR1B	1.694	3.235	0.000E+00
KANK2	1.694	3.235	0.000E+00
NOC2LP2	1.695	3.238	2.714E-03
SMPD3	1.696	3.239	0.000E+00
BLZF2P	1.697	3.243	5.523E-06
AL355073.2	1.697	3.243	4.052E-05
SV2B	1.699	3.247	7.551E-04
CDA	1.700	3.250	0.000E+00
RPS4XP11	1.700	3.250	9.006E-07
AC012368.1	1.701	3.252	0.000E+00
SIRPB3P	1.703	3.255	4.195E-06
AL021707.7	1.703	3.255	2.551E-06
MIR26B	1.704	3.257	4.858E-04
FADD	1.704	3.257	0.000E+00
BMP3	1.704	3.259	5.373E-03
PAN3-AS1	1.704	3.259	0.000E+00
CACNG6	1.707	3.264	2.767E-08
SIT1	1.707	3.265	0.000E+00
FDX2	1.708	3.267	2.081E-04
AL391832.2	1.708	3.268	0.000E+00
AP006621.2	1.710	3.271	0.000E+00
AL138787.2	1.710	3.272	5.205E-03
RAET1K	1.713	3.278	8.895E-03
E2F3-IT1	1.713	3.279	5.212E-03
AC008610.1	1.714	3.280	0.000E+00
AC010422.4	1.715	3.283	8.371E-07
RPL36AP45	1.716	3.285	7.609E-03
PHF5CP	1.717	3.288	2.369E-04
LINC00211	1.717	3.288	1.177E-06
AC011472.2	1.719	3.291	8.151E-08
AL163051.1	1.719	3.291	1.456E-07
CXCR3	1.722	3.298	0.000E+00
ZMYND10	1.722	3.299	0.000E+00
AC023830.3	1.723	3.301	1.158E-07
CNN2P4	1.724	3.304	6.393E-04
HSPA8P14	1.728	3.312	1.111E-03
RPS3AP18	1.728	3.312	8.863E-10
CEND1	1.729	3.315	5.106E-04
THBD	1.730	3.317	2.554E-09
RAB3D	1.733	3.323	0.000E+00
ZNF213	1.735	3.329	0.000E+00
AC105339.2	1.735	3.330	0.000E+00
AL033527.2	1.736	3.331	7.375E-05
ICAM3	1.737	3.333	0.000E+00
MRPL20-DT	1.739	3.337	0.000E+00
RNU6-925P	1.740	3.340	3.717E-04
AC068946.1	1.741	3.342	3.533E-07
AC125612.1	1.744	3.350	5.367E-03
AC048341.2	1.744	3.351	0.000E+00
AC139887.5	1.745	3.353	1.305E-04
AC010619.2	1.745	3.353	0.000E+00
BSG	1.746	3.355	0.000E+00
AC004057.1	1.747	3.356	2.636E-03

AC080112.4	1.748	3.359	1.778E-09
AL355490.1	1.749	3.361	4.593E-08
LINC02649	1.749	3.361	0.000E+00
B3GALT2	1.750	3.364	8.701E-07
AC087277.2	1.751	3.365	2.556E-06
LINC02818	1.751	3.365	3.549E-03
RHOXF1P3	1.751	3.366	4.888E-03
AL590822.2	1.751	3.367	2.069E-07
TMEM132C	1.752	3.368	9.135E-04
VASP	1.752	3.369	0.000E+00
RAB36	1.753	3.369	0.000E+00
CEACAM1	1.753	3.370	1.936E-06
MAP1LC3B2	1.754	3.372	3.944E-10
LINC01127	1.754	3.373	0.000E+00
RUNDC3A-AS1	1.756	3.377	5.920E-08
AL590822.1	1.760	3.386	3.399E-03
LRP10	1.760	3.387	0.000E+00
AL390067.1	1.762	3.392	2.699E-05
RTN2	1.762	3.393	0.000E+00
CPTP	1.763	3.395	0.000E+00
TNFAIP6	1.763	3.395	1.226E-03
KDM6B	1.765	3.398	0.000E+00
RASGRP4	1.766	3.401	0.000E+00
TNFAIP8L2	1.766	3.401	0.000E+00
TMEM132E	1.766	3.402	3.086E-04
WNT6	1.767	3.403	3.831E-05
PADI2	1.767	3.403	2.553E-09
BCL6B	1.769	3.408	7.145E-03
RNF24	1.769	3.408	0.000E+00
POU5F1P6	1.770	3.410	4.797E-05
AC116407.1	1.770	3.412	1.724E-03
TMEM240	1.771	3.412	1.624E-06
DAAM2	1.772	3.415	4.190E-03
SLFNL1	1.772	3.415	1.182E-06
AC123912.4	1.773	3.418	5.207E-05
HEPH	1.775	3.423	2.657E-04
AC023424.1	1.775	3.423	2.740E-05
MED25	1.779	3.433	0.000E+00
CSF1	1.782	3.440	0.000E+00
CAPN10-DT	1.786	3.449	0.000E+00
AC093281.2	1.787	3.451	5.122E-03
CORO2B	1.788	3.453	5.430E-06
RNA5SP283	1.788	3.454	2.967E-04
AC112253.1	1.789	3.457	2.346E-04
AC079944.2	1.790	3.458	1.157E-09
SRXN1	1.790	3.458	2.421E-06
AC092171.3	1.790	3.459	3.029E-04
RPS2P17	1.791	3.461	0.000E+00
AP000769.1	1.793	3.465	0.000E+00
SECTM1	1.794	3.467	0.000E+00
RPS27AP6	1.795	3.470	8.923E-03
AC008676.3	1.798	3.477	4.373E-04
MXI1	1.798	3.478	0.000E+00
AL137246.1	1.799	3.480	5.573E-03
MIR1203	1.801	3.486	6.036E-03

MIR1249	1.802	3.487	1.588E-04
SH2B2	1.803	3.489	0.000E+00
AC097641.2	1.803	3.490	5.645E-09
LINC01001	1.803	3.491	0.000E+00
SLC2A3P2	1.804	3.492	1.124E-05
RPL7P18	1.806	3.496	2.232E-04
AL355432.1	1.806	3.496	6.192E-03
AMH	1.806	3.497	2.785E-08
LIMK2	1.806	3.497	1.590E-10
RPS2P44	1.806	3.497	1.450E-03
LPAR2	1.806	3.498	0.000E+00
ACOXL	1.807	3.500	3.351E-05
STIM1-AS1	1.808	3.502	1.010E-03
AP000640.1	1.808	3.502	0.000E+00
CA3-AS1	1.809	3.504	0.000E+00
AC092368.3	1.810	3.506	0.000E+00
GRK6P1	1.811	3.509	5.319E-03
AL356481.2	1.811	3.510	9.811E-05
AC092746.1	1.812	3.510	5.674E-05
TRAPPC5	1.812	3.512	0.000E+00
INSL3	1.814	3.515	0.000E+00
AC066616.2	1.817	3.524	1.471E-03
PRRG4	1.818	3.527	0.000E+00
AL021707.6	1.820	3.530	0.000E+00
STARD10	1.820	3.531	0.000E+00
SPCS2P4	1.824	3.540	0.000E+00
F2RL1	1.826	3.545	1.984E-08
AC021766.1	1.826	3.546	6.110E-03
TAL1	1.826	3.546	0.000E+00
AC027279.4	1.828	3.550	3.719E-03
LIX1L-AS1	1.828	3.550	8.428E-05
ZNF467	1.829	3.552	0.000E+00
AP000944.1	1.829	3.553	5.575E-03
AL162724.1	1.830	3.556	3.292E-04
RNU7-45P	1.831	3.557	9.559E-06
EYS	1.833	3.563	4.436E-05
RPL5P13	1.833	3.563	6.097E-03
AC025682.1	1.834	3.564	0.000E+00
AC138696.2	1.834	3.565	6.723E-04
BEND7	1.835	3.568	1.144E-06
OLIG1	1.838	3.574	0.000E+00
RHD	1.838	3.575	4.052E-05
CREB3L3	1.839	3.578	4.476E-04
HLX	1.842	3.586	0.000E+00
AC009716.2	1.843	3.588	4.746E-06
AC016747.3	1.844	3.590	1.875E-07
COX6B2	1.844	3.590	1.611E-03
TM4SF19	1.845	3.594	5.998E-07
PIK3CD-AS1	1.847	3.599	0.000E+00
TMLHE-AS1	1.850	3.605	8.194E-05
SCAT8	1.852	3.610	0.000E+00
AP001107.1	1.852	3.610	5.552E-03
BPGM	1.855	3.618	0.000E+00
AC008750.7	1.856	3.621	8.472E-09
AC072054.1	1.857	3.621	1.238E-03

AC103702.2	1.858	3.626	1.146E-04
MAK	1.858	3.626	1.059E-08
LIME1	1.861	3.632	0.000E+00
H3-3A-DT	1.861	3.634	0.000E+00
TECPR2	1.862	3.636	0.000E+00
AC006435.3	1.864	3.639	7.166E-03
MTHFS	1.864	3.641	0.000E+00
AC015802.4	1.865	3.642	0.000E+00
TSC22D1-AS1	1.865	3.643	0.000E+00
MIR339	1.868	3.651	4.493E-06
AC027682.5	1.869	3.653	6.329E-05
WFIKKN1	1.869	3.653	0.000E+00
AL391121.1	1.870	3.654	0.000E+00
MIR320E	1.870	3.655	0.000E+00
SLC16A13	1.871	3.657	0.000E+00
EMID1	1.871	3.658	0.000E+00
AP001160.1	1.871	3.659	5.674E-10
USP12-AS2	1.872	3.661	1.156E-03
AC104530.1	1.872	3.661	0.000E+00
PTP4A3	1.873	3.663	1.899E-09
HS3ST1	1.876	3.671	2.981E-10
RN7SL45P	1.880	3.680	1.478E-04
METTL21AP1	1.881	3.683	4.374E-03
AC018442.1	1.881	3.684	3.274E-04
AL135818.1	1.881	3.684	0.000E+00
AL035661.1	1.883	3.687	1.222E-04
NFAM1	1.884	3.692	0.000E+00
MAP1B	1.884	3.692	1.994E-03
U91328.3	1.888	3.702	4.781E-08
MTND5P32	1.889	3.705	7.863E-04
AC004771.3	1.891	3.710	5.063E-04
MTCYBP23	1.892	3.711	4.729E-04
LGALS8-AS1	1.893	3.714	9.131E-04
CNIH3	1.894	3.718	0.000E+00
AC005746.1	1.895	3.720	3.052E-09
AC068491.2	1.895	3.720	3.356E-03
AL162274.1	1.896	3.721	4.863E-05
AC022431.1	1.897	3.723	3.940E-04
MIR3945HG	1.897	3.723	3.953E-05
H1-6	1.898	3.726	2.768E-03
PTMS	1.899	3.729	0.000E+00
CISH	1.899	3.730	0.000E+00
LINC00639	1.900	3.732	7.803E-03
AC132872.5	1.900	3.733	0.000E+00
IRAG1	1.902	3.738	3.672E-09
AC022306.3	1.903	3.741	3.665E-04
AC068205.2	1.905	3.746	0.000E+00
AC078962.1	1.910	3.759	9.375E-05
FTH1P12	1.911	3.761	7.407E-08
AP006545.1	1.911	3.762	6.112E-05
MIR548O	1.915	3.771	2.881E-03
AL031775.2	1.917	3.775	4.820E-06
AL022318.2	1.917	3.777	6.292E-04
XK	1.918	3.779	0.000E+00
TMEM140	1.918	3.779	0.000E+00
GRIN1	1.919	3.781	5.965E-03
AC025809.2	1.919	3.781	3.148E-03
AL031432.5	1.920	3.785	7.226E-05
ELAPOR1	1.921	3.786	1.272E-08
NKAPL	1.921	3.787	5.898E-07
MAFA	1.923	3.791	2.573E-03
CTU1	1.923	3.792	0.000E+00
AC027682.7	1.927	3.802	0.000E+00
AC089999.2	1.928	3.804	1.673E-05
ADAMTSL4-AS1	1.929	3.807	2.764E-10
ANXA3	1.929	3.809	1.698E-05
RNA5S9	1.932	3.815	7.174E-03
FLJ42393	1.932	3.816	3.672E-07
SPNS2	1.936	3.827	0.000E+00
AC006064.4	1.936	3.827	0.000E+00
AC034102.5	1.937	3.828	7.788E-09
AC112503.1	1.939	3.834	3.591E-05
SBNO2	1.940	3.836	6.979E-09
AC120057.3	1.945	3.851	4.591E-09
UBB	1.947	3.855	0.000E+00
NT5DC4	1.947	3.857	4.229E-08
AC103858.3	1.947	3.857	0.000E+00
RNU7-181P	1.948	3.858	3.806E-05
AC115284.4	1.949	3.860	6.171E-06
AL358473.1	1.950	3.863	1.207E-07
AC015813.5	1.950	3.865	1.335E-03
AC001226.1	1.951	3.866	9.912E-04
AC245140.2	1.952	3.868	0.000E+00
MPZ	1.952	3.870	0.000E+00
NATD1	1.953	3.871	0.000E+00
HAGH	1.953	3.872	0.000E+00
AC098476.1	1.953	3.872	3.825E-03
AC121338.1	1.957	3.882	1.743E-05
FRAT1	1.959	3.888	0.000E+00
AF129075.3	1.959	3.889	9.048E-03
AC004687.1	1.960	3.890	0.000E+00
AC124068.1	1.962	3.895	1.823E-04
AL139020.1	1.963	3.899	4.839E-08
LINGO3	1.963	3.899	0.000E+00
LST1	1.965	3.905	2.699E-09
LSMEM1	1.967	3.910	2.800E-10
RPS6KB2-AS1	1.969	3.914	1.570E-07
AC139099.2	1.969	3.916	1.444E-04
FCGR2A	1.972	3.922	0.000E+00
BATF2	1.972	3.923	2.036E-07
TGM2	1.972	3.924	0.000E+00
ADM	1.972	3.924	1.335E-07
RPL32P32	1.973	3.926	3.968E-05
RNA5SP298	1.973	3.926	1.522E-03
SMPDL3B	1.974	3.927	0.000E+00
AC087672.2	1.976	3.935	3.847E-10
AC092802.1	1.976	3.935	8.166E-04
AL355377.3	1.977	3.936	0.000E+00
AC010615.2	1.978	3.940	5.154E-06
CCN2	1.979	3.941	6.499E-04

C5orf67	1.980	3.944	4.270E-06
LINC01136	1.980	3.946	3.138E-06
AC005696.3	1.982	3.949	2.505E-09
MIR3605	1.982	3.951	2.755E-08
MIR326	1.984	3.955	4.448E-03
VNN2	1.985	3.959	0.000E+00
AC113361.1	1.989	3.970	1.993E-04
CACNA1A	1.989	3.970	0.000E+00
AL500527.1	1.994	3.985	1.906E-04
C10orf55	1.995	3.986	9.041E-04
FUT7	1.997	3.992	0.000E+00
LINC00685	1.997	3.992	1.141E-03
DCST1	1.997	3.993	2.509E-07
LYVE1	1.998	3.993	2.948E-05
AC018737.2	1.998	3.995	9.611E-03
LINC00173	2.001	4.002	0.000E+00
LRWD1	2.003	4.009	0.000E+00
AC022973.1	2.003	4.009	5.808E-03
AC129492.6	2.005	4.013	8.608E-05
LINC01529	2.005	4.014	8.146E-05
CAMK2B	2.005	4.014	6.587E-04
AC145285.3	2.006	4.018	2.914E-06
AC103810.3	2.011	4.029	1.082E-09
AQP9	2.011	4.031	6.500E-08
AC099489.2	2.012	4.033	5.029E-08
AL137002.1	2.013	4.036	8.513E-09
MNDA	2.013	4.036	0.000E+00
AL138963.1	2.014	4.039	6.735E-09
LRRK2-DT	2.015	4.041	0.000E+00
AL157871.1	2.015	4.042	3.385E-03
ADAMTSL4-AS2	2.016	4.043	0.000E+00
CYSTM1	2.016	4.043	0.000E+00
AC005944.1	2.016	4.045	5.280E-07
AL356218.1	2.019	4.054	4.885E-05
AL117187.1	2.021	4.059	4.545E-03
AC011816.1	2.021	4.060	2.218E-05
AC112173.1	2.022	4.062	1.257E-03
SLC16A3	2.023	4.065	0.000E+00
CFAP73	2.025	4.070	5.240E-05
AC019254.1	2.027	4.075	3.668E-05
COL9A3	2.029	4.081	0.000E+00
RNA5SP352	2.029	4.082	3.301E-03
RNU6-761P	2.030	4.083	2.343E-07
HPN	2.035	4.098	2.304E-06
GPR82	2.036	4.101	1.569E-06
LRRN2	2.038	4.106	1.309E-03
FOLR3	2.041	4.115	2.175E-09
AC013731.1	2.041	4.116	9.951E-07
TRMT112P6	2.041	4.117	3.447E-03
HSPA1B	2.042	4.118	1.940E-06
RSPH14	2.042	4.119	6.443E-06
HIGD1AP1	2.044	4.123	7.137E-03
PABPN1L	2.045	4.128	0.000E+00
TRPM6	2.049	4.139	1.826E-07
PARS2	2.054	4.152	0.000E+00
RN7SL683P	2.054	4.152	3.384E-03
BANF1P3	2.055	4.154	1.589E-03
PGAM2	2.058	4.164	1.152E-09
HEMGN	2.059	4.166	0.000E+00
H4C5	2.060	4.171	1.976E-10
RNU6-678P	2.061	4.172	1.550E-03
SNORC	2.061	4.174	0.000E+00
AL161785.1	2.062	4.175	0.000E+00
SUMO1P1	2.062	4.175	3.726E-06
EFNA3	2.063	4.179	0.000E+00
ARAP3	2.064	4.183	0.000E+00
BBOF1	2.067	4.189	0.000E+00
C9orf163	2.067	4.190	5.904E-07
RNU6-529P	2.068	4.192	1.751E-03
MIRLET7I	2.070	4.199	5.933E-04
AC006538.1	2.070	4.199	0.000E+00
BNIPL	2.071	4.201	0.000E+00
AC090907.1	2.074	4.212	1.469E-06
COL18A1	2.075	4.214	0.000E+00
SYN2	2.075	4.215	1.717E-06
Z97192.2	2.076	4.217	0.000E+00
AC027279.1	2.077	4.220	9.657E-08
AL031658.1	2.079	4.226	1.475E-05
AL731559.1	2.082	4.235	1.816E-08
AC008750.3	2.083	4.238	8.237E-03
XPC-AS1	2.084	4.240	1.143E-10
H2BC4	2.090	4.257	1.463E-09
NDUFAF2P2	2.091	4.260	9.847E-04
AC005041.4	2.092	4.262	1.134E-05
AC092068.1	2.094	4.269	4.752E-03
AC023908.3	2.094	4.271	0.000E+00
FPR1	2.097	4.279	0.000E+00
AL359762.1	2.098	4.281	0.000E+00
FFAR3	2.098	4.282	5.779E-04
AC016876.2	2.100	4.288	2.090E-05
AL031666.3	2.102	4.293	2.590E-04
RCN3	2.103	4.296	0.000E+00
DRAIC	2.104	4.299	0.000E+00
AC087284.1	2.105	4.303	0.000E+00
RN7SL648P	2.106	4.306	1.258E-08
CR388215.1	2.108	4.309	8.978E-03
SPINK8	2.109	4.315	1.174E-05
AL132655.2	2.110	4.316	2.387E-04
COX20P1	2.110	4.317	3.384E-03
BTBD17	2.111	4.320	6.489E-04
ITGB4	2.112	4.322	0.000E+00
ASAP1-IT2	2.116	4.334	0.000E+00
TMEM88	2.121	4.350	0.000E+00
TNFRSF1A	2.121	4.350	0.000E+00
TEK	2.121	4.350	1.052E-03
CXCL6	2.122	4.352	7.391E-04
AL355490.2	2.125	4.362	1.231E-03
P2RY13	2.126	4.366	0.000E+00
CLC	2.126	4.366	0.000E+00
AL022313.2	2.127	4.367	6.141E-04

AC002044.2	2.128	4.371	9.926E-04
EPHB1	2.129	4.373	4.624E-10
PPIAP30	2.130	4.377	1.108E-03
AC099778.1	2.131	4.379	0.000E+00
ATE1-AS1	2.132	4.385	4.003E-08
SLC45A4	2.133	4.387	0.000E+00
NLRP6	2.135	4.393	0.000E+00
MESP2	2.136	4.395	6.090E-03
TTC25	2.136	4.395	0.000E+00
FHDC1	2.136	4.396	0.000E+00
AC010719.1	2.138	4.403	7.248E-05
HNRNPA1P21	2.140	4.407	0.000E+00
YPEL3	2.141	4.409	0.000E+00
AL357500.1	2.143	4.416	1.094E-03
AC007877.1	2.144	4.419	0.000E+00
MIR4480	2.145	4.423	7.089E-03
MIR4653	2.146	4.426	2.756E-03
AC098818.1	2.148	4.432	6.714E-03
FBXO7	2.149	4.436	0.000E+00
DNASE1L2	2.154	4.452	0.000E+00
CNTNAP3C	2.155	4.455	1.573E-03
AC080080.1	2.156	4.456	0.000E+00
OR7E100P	2.158	4.464	1.011E-03
AL360091.2	2.159	4.465	8.531E-05
AC006960.3	2.159	4.466	6.515E-06
AC011446.2	2.159	4.467	1.697E-10
Z97986.1	2.159	4.467	2.054E-04
AP001267.1	2.159	4.467	4.604E-03
ST20	2.160	4.468	0.000E+00
S1PR4	2.162	4.476	0.000E+00
RNU6-945P	2.164	4.481	4.560E-04
AC004923.4	2.164	4.482	2.825E-05
TFF3	2.165	4.484	3.551E-05
HSP90AB4P	2.165	4.486	6.646E-08
AC015883.1	2.166	4.489	9.979E-07
DHRS9	2.167	4.492	0.000E+00
AC005041.3	2.168	4.494	1.598E-08
POTEE	2.172	4.505	2.477E-06
RPL35AP2	2.172	4.506	8.601E-05
PTGDR2	2.175	4.515	0.000E+00
AC060234.1	2.177	4.521	3.752E-03
BAG1	2.177	4.521	0.000E+00
PCF11-AS1	2.178	4.526	0.000E+00
LINC00862	2.181	4.535	0.000E+00
AC018521.4	2.182	4.539	7.628E-03
GHRL	2.183	4.540	0.000E+00
ST6GALNAC4	2.188	4.555	0.000E+00
AC005529.1	2.190	4.562	4.755E-06
AC130448.1	2.190	4.564	7.230E-09
AL031846.2	2.190	4.564	1.631E-03
LINC01303	2.191	4.568	8.733E-07
IRAG1-AS1	2.192	4.568	5.963E-10
NFE2L1-DT	2.193	4.573	0.000E+00
AC013451.2	2.196	4.581	1.012E-04
RN7SL473P	2.198	4.588	0.000E+00

AC012615.6	2.198	4.588	3.500E-04
AL109809.4	2.198	4.589	0.000E+00
AL133551.1	2.199	4.592	1.602E-06
AC113139.1	2.201	4.598	1.750E-05
AC133555.1	2.203	4.605	4.915E-04
ZDHHC18	2.203	4.606	0.000E+00
AC099524.1	2.204	4.607	0.000E+00
TMED11P	2.207	4.618	5.519E-03
GUK1	2.209	4.624	0.000E+00
RPS15AP29	2.210	4.628	1.554E-06
MIR1282	2.210	4.628	2.449E-09
LIPC-AS1	2.211	4.630	4.683E-03
OLAH	2.212	4.632	2.786E-04
AC005726.1	2.212	4.633	1.526E-07
SHISA8	2.214	4.640	1.410E-06
AC090527.3	2.217	4.648	1.097E-05
SIGLEC5	2.219	4.655	0.000E+00
REM2	2.219	4.657	0.000E+00
CCL23	2.221	4.662	9.290E-03
MIR140	2.223	4.670	4.613E-03
SNORD56B	2.224	4.673	6.996E-03
AC068580.3	2.228	4.684	1.768E-04
MIR6813	2.229	4.688	1.270E-10
AC016888.1	2.229	4.688	5.480E-05
SLC22A1	2.230	4.693	2.639E-10
RN7SL3	2.231	4.694	1.250E-03
CRACDL	2.231	4.694	0.000E+00
HDHD5-AS1	2.232	4.699	2.126E-04
AL031600.3	2.233	4.701	8.548E-04
CMTM1	2.233	4.702	0.000E+00
H2BC18	2.234	4.703	5.230E-09
RN7SL288P	2.234	4.705	1.619E-05
AL356652.1	2.235	4.707	4.974E-04
AC073195.1	2.240	4.724	0.000E+00
AL592078.1	2.240	4.725	4.277E-07
PTX4	2.242	4.730	1.010E-07
KCNG1	2.244	4.736	0.000E+00
AC092828.1	2.244	4.736	2.744E-03
AC020763.4	2.245	4.740	5.049E-03
GLRX5	2.245	4.742	0.000E+00
RN7SL743P	2.247	4.745	5.664E-03
PGLYRP1	2.247	4.747	3.073E-08
AC092384.3	2.247	4.747	0.000E+00
AL590133.2	2.250	4.757	1.844E-07
AC008440.1	2.252	4.762	6.040E-04
NPRL3	2.255	4.772	0.000E+00
CACNG8	2.255	4.773	0.000E+00
PINK1	2.259	4.787	0.000E+00
AC010331.1	2.259	4.788	0.000E+00
ACSS3	2.259	4.788	1.481E-08
AGAP7P	2.260	4.789	3.312E-07
CFAP58-DT	2.260	4.790	8.986E-07
CAPN13	2.261	4.792	4.453E-03
AC016590.3	2.263	4.801	2.713E-04
ACTL10	2.264	4.802	0.000E+00

TSPAN16	2.264	4.802	4.330E-03
TSPAN5	2.264	4.803	0.000E+00
MIR378D2HG	2.267	4.813	2.053E-03
SMARCE1P1	2.269	4.818	3.001E-03
ANK1	2.269	4.819	0.000E+00
AC093323.3	2.269	4.821	3.786E-05
MIR3661	2.270	4.822	2.012E-03
FAM157A	2.270	4.824	1.106E-09
H3C6	2.272	4.831	0.000E+00
FBXL13	2.273	4.833	6.514E-08
DHX34	2.275	4.841	0.000E+00
PPP4R1-AS1	2.276	4.844	8.861E-06
MIR194-2HG	2.278	4.848	9.814E-10
BGLAP	2.284	4.869	0.000E+00
MFSD2B	2.286	4.877	0.000E+00
AC004477.2	2.286	4.879	3.295E-03
RN7SL600P	2.287	4.879	0.000E+00
MAP3K15	2.287	4.880	8.015E-06
AL627309.7	2.287	4.881	0.000E+00
AC097637.2	2.289	4.888	0.000E+00
STEAP4	2.289	4.889	7.525E-10
AC018926.1	2.291	4.894	0.000E+00
AP000879.1	2.292	4.899	3.248E-03
AC036222.2	2.293	4.900	2.616E-04
RARA-AS1	2.293	4.902	0.000E+00
AC007342.9	2.294	4.904	0.000E+00
GPR25	2.295	4.906	0.000E+00
FAM83A	2.297	4.914	7.006E-06
AC010997.6	2.297	4.915	3.822E-04
AC135050.1	2.298	4.918	2.257E-06
BLVRB	2.299	4.921	0.000E+00
HMGB1P9	2.300	4.924	4.165E-03
AL512288.1	2.300	4.926	1.683E-03
AL357552.2	2.302	4.932	7.144E-04
ABTB1	2.306	4.946	0.000E+00
MYO16-AS1	2.307	4.948	3.346E-04
AL158211.1	2.307	4.948	1.341E-04
AC106820.2	2.308	4.951	4.054E-05
LINC00957	2.308	4.953	0.000E+00
LINC01002	2.309	4.957	0.000E+00
MTND6P3	2.310	4.958	1.866E-03
RNU4-40P	2.310	4.960	4.485E-04
NCF4	2.311	4.961	0.000E+00
AC010547.1	2.312	4.967	1.092E-03
MIR4477B	2.316	4.979	0.000E+00
AP001099.1	2.316	4.981	2.681E-07
CD177	2.317	4.984	5.408E-05
AC090907.2	2.318	4.985	2.017E-06
LINC01816	2.320	4.993	4.149E-04
AL606500.1	2.321	4.997	1.742E-05
AP003170.4	2.323	5.003	2.991E-03
ST6GALNAC2	2.323	5.004	0.000E+00
DAPK1-IT1	2.323	5.005	1.480E-03
AC007950.1	2.325	5.012	3.620E-03
GLIS2	2.327	5.018	9.204E-03
CKAP2LP1	2.328	5.020	4.435E-06
IL5RA	2.335	5.044	0.000E+00
ARHGAP27P2	2.336	5.048	0.000E+00
AC005072.1	2.337	5.053	3.996E-08
AC073534.2	2.337	5.053	9.461E-06
AC138028.3	2.338	5.058	5.638E-05
AC092068.3	2.341	5.068	9.235E-08
RTEL1P1	2.344	5.077	0.000E+00
FPR2	2.346	5.083	0.000E+00
LEP	2.348	5.091	8.057E-06
GAS2L1P2	2.348	5.093	4.244E-03
SLC26A8	2.349	5.096	0.000E+00
AC073517.1	2.351	5.101	1.860E-04
C7orf61	2.351	5.102	0.000E+00
AC139530.3	2.353	5.108	0.000E+00
AC114271.1	2.354	5.111	0.000E+00
CBS	2.354	5.113	7.980E-03
BIN2P1	2.355	5.115	0.000E+00
CHRNA10	2.355	5.117	0.000E+00
MIR4285	2.356	5.118	1.970E-03
AL031595.1	2.356	5.119	3.025E-05
MTDHP1	2.357	5.124	1.970E-03
GPR17	2.358	5.128	1.407E-03
AC105052.5	2.361	5.136	3.370E-07
LINC00885	2.364	5.146	2.723E-04
AC079804.3	2.364	5.147	6.052E-06
AL132708.1	2.365	5.153	2.199E-03
AL627309.1	2.367	5.158	1.980E-10
CPLX2	2.368	5.161	1.147E-03
LINC02158	2.368	5.162	0.000E+00
CLRN1-AS1	2.368	5.163	3.147E-03
AC092139.2	2.369	5.164	2.175E-09
SLC5A9	2.370	5.169	0.000E+00
AC007663.3	2.370	5.169	8.150E-06
AP003031.1	2.371	5.174	1.293E-03
AL355472.3	2.371	5.175	2.013E-05
CDC34	2.372	5.176	0.000E+00
RN7SL801P	2.374	5.185	4.694E-03
RN7SL698P	2.375	5.188	1.036E-03
AC005329.3	2.377	5.194	3.943E-04
FAM43A	2.377	5.196	0.000E+00
INKA2	2.379	5.201	0.000E+00
AL356535.1	2.381	5.211	3.286E-10
MAP1LC3A	2.383	5.216	0.000E+00
CYP4F12	2.383	5.218	0.000E+00
TRHDE-AS1	2.385	5.225	1.282E-03
DAPK2	2.386	5.226	0.000E+00
AC105137.2	2.387	5.229	4.210E-08
PRR7-AS1	2.390	5.240	0.000E+00
IGFBP7-AS1	2.390	5.242	4.448E-03
DPRXP2	2.391	5.246	1.032E-04
HLA-J	2.392	5.250	1.736E-03
NCF1	2.393	5.254	0.000E+00
CNTNAP3	2.397	5.267	1.192E-04
GK-IT1	2.398	5.272	8.282E-09

RTN4R	2.401	5.282	0.000E+00
AC097015.1	2.402	5.286	5.710E-06
AC009065.2	2.402	5.287	2.449E-03
C5AR2	2.406	5.299	0.000E+00
AC022506.2	2.408	5.306	0.000E+00
S100P	2.408	5.307	1.189E-09
AL118508.3	2.412	5.322	0.000E+00
AL355310.2	2.413	5.327	1.661E-03
AL049757.1	2.414	5.328	0.000E+00
AC011933.2	2.414	5.329	0.000E+00
LINC01305	2.416	5.336	2.422E-05
AC090617.1	2.418	5.344	9.903E-03
CFAP58	2.419	5.347	0.000E+00
KCNJ2	2.421	5.355	4.227E-07
TESC	2.424	5.368	0.000E+00
DNAJB6P7	2.425	5.369	6.669E-05
RN7SL735P	2.427	5.380	2.940E-03
RNU6-1003P	2.430	5.388	1.672E-05
SPACA6	2.433	5.398	0.000E+00
AL358072.1	2.433	5.399	0.000E+00
C10orf105	2.433	5.399	0.000E+00
ABHD16B	2.434	5.405	1.774E-03
TMEM92	2.435	5.407	2.606E-03
LINC02218	2.435	5.408	3.834E-04
AC246785.2	2.435	5.409	1.536E-03
BEST1	2.437	5.415	0.000E+00
YBX3	2.438	5.419	0.000E+00
CCR3	2.439	5.422	0.000E+00
A4GALT	2.441	5.430	2.253E-06
AC010359.2	2.443	5.436	0.000E+00
MSRB1	2.445	5.445	0.000E+00
EPCAM	2.445	5.447	1.553E-04
AC008073.1	2.446	5.449	1.994E-03
TUBB2A	2.447	5.452	1.064E-08
ADGRE3	2.448	5.456	0.000E+00
RAB1AP1	2.450	5.463	2.936E-10
RALGPS2-AS1	2.451	5.469	3.039E-09
ISL2	2.452	5.473	0.000E+00
NFIX	2.453	5.476	0.000E+00
AL031777.2	2.454	5.481	1.729E-10
RC3H1-IT1	2.456	5.487	2.939E-07
AC005392.2	2.456	5.488	4.753E-05
AC009133.2	2.459	5.499	0.000E+00
NCF1C	2.461	5.508	0.000E+00
AP003721.4	2.462	5.511	4.010E-06
HCAR3	2.463	5.514	8.623E-09
AC002551.1	2.465	5.522	5.293E-03
LRRC4	2.466	5.525	0.000E+00
AC079316.2	2.468	5.531	0.000E+00
DUSP13	2.468	5.534	1.154E-07
R3HDM4	2.469	5.536	0.000E+00
IGHG3	2.469	5.536	3.038E-06
ACTBP2	2.470	5.542	0.000E+00
AVIL	2.471	5.543	0.000E+00
AC111170.3	2.472	5.548	0.000E+00
AC009121.2	2.474	5.554	4.122E-03
AL590326.1	2.474	5.556	1.664E-09
ARHGEF37	2.474	5.557	0.000E+00
PROK2	2.477	5.569	1.909E-08
AC011726.1	2.479	5.576	4.230E-03
MIR3613	2.480	5.577	4.194E-07
TIMMDC1-DT	2.481	5.583	8.386E-04
AC016831.4	2.482	5.586	0.000E+00
AC107982.3	2.482	5.587	1.230E-06
RNU6-176P	2.483	5.591	7.642E-07
AC138123.1	2.484	5.596	2.000E-05
HMGN2P6	2.488	5.609	9.470E-03
AC090559.2	2.490	5.616	4.085E-07
RPL17P10	2.490	5.617	0.000E+00
AC009120.5	2.491	5.620	2.216E-04
AC012150.2	2.491	5.621	2.826E-07
ASPRV1	2.492	5.626	0.000E+00
AC107993.1	2.495	5.639	1.761E-04
PDLIM7	2.496	5.639	0.000E+00
AC145207.9	2.505	5.676	8.162E-10
AC005280.2	2.506	5.680	0.000E+00
CTBP1-AS	2.508	5.686	6.227E-07
AL390065.1	2.509	5.691	3.902E-04
RPRM	2.512	5.702	4.335E-03
NCF1B	2.512	5.703	0.000E+00
OLFM2	2.512	5.704	0.000E+00
OR51R1P	2.512	5.705	3.343E-06
HPCA	2.513	5.709	3.876E-05
MIR548C	2.514	5.712	2.688E-05
MIR223HG	2.515	5.714	0.000E+00
AC069366.1	2.515	5.716	2.495E-06
AC019294.3	2.515	5.716	8.972E-04
CSF3R	2.516	5.718	0.000E+00
AC092069.1	2.516	5.719	1.558E-04
CFD	2.519	5.730	4.289E-04
GCM1	2.520	5.735	0.000E+00
SLC19A1	2.522	5.742	0.000E+00
RNA5SP145	2.522	5.745	2.700E-04
MRGPRE	2.525	5.755	9.674E-03
AQP1	2.528	5.767	0.000E+00
FRAT2	2.529	5.771	0.000E+00
AL590491.2	2.529	5.771	7.825E-03
IFITM2	2.531	5.781	0.000E+00
AC093591.1	2.532	5.783	1.205E-06
NFE2	2.534	5.794	0.000E+00
NSUN7	2.537	5.804	0.000E+00
MRC2	2.538	5.806	0.000E+00
OXER1	2.538	5.806	0.000E+00
AOC2	2.538	5.807	0.000E+00
AC083843.3	2.542	5.826	4.474E-10
ADIPOR1	2.543	5.829	0.000E+00
AC087441.1	2.545	5.836	4.005E-05
MICOS10P2	2.553	5.868	3.412E-04
RN7SKP95	2.555	5.875	6.701E-03
RIMBP3	2.555	5.876	0.000E+00

TREML2	2.555	5.877	0.000E+00
AL049198.1	2.560	5.895	2.882E-04
GIMAP3P	2.560	5.898	1.901E-06
HHATL	2.561	5.899	1.923E-06
PCSK1N	2.561	5.902	0.000E+00
AC136603.1	2.565	5.918	8.993E-03
GRIN3B	2.569	5.934	0.000E+00
IL1R2	2.573	5.950	0.000E+00
NKX6-3	2.573	5.952	3.751E-04
AC010894.4	2.573	5.952	0.000E+00
LINC02555	2.574	5.956	4.340E-08
RN7SL233P	2.574	5.956	2.610E-05
AC068669.1	2.574	5.956	3.544E-09
AC006441.3	2.575	5.960	3.977E-03
AC021424.4	2.577	5.966	4.812E-03
INHBB	2.578	5.971	1.585E-07
BASP1-AS1	2.580	5.978	5.015E-07
LINC02352	2.580	5.979	0.000E+00
AC010491.1	2.582	5.989	0.000E+00
C3orf86	2.583	5.992	0.000E+00
SOWAHD	2.584	5.995	0.000E+00
AC079171.1	2.586	6.003	4.325E-05
PPIAL4C	2.589	6.015	5.014E-05
RN7SKP9	2.590	6.019	1.802E-05
AL158063.1	2.591	6.025	4.044E-09
AC008496.3	2.594	6.039	7.318E-03
AC145098.2	2.595	6.042	0.000E+00
AP006621.1	2.597	6.050	4.556E-09
HEY1	2.599	6.059	3.062E-09
ARHGEF2-AS2	2.600	6.061	0.000E+00
SCN2B	2.600	6.064	1.352E-05
ROPN1L	2.601	6.065	0.000E+00
AC145423.1	2.601	6.069	1.465E-05
LINC02863	2.603	6.073	0.000E+00
U73166.1	2.603	6.075	0.000E+00
KIAA0319	2.605	6.085	1.363E-09
AC025430.1	2.606	6.087	3.818E-03
PLVAP	2.607	6.093	0.000E+00
TSPAN7	2.608	6.095	1.260E-08
CCNJL	2.608	6.098	0.000E+00
OOSP3	2.612	6.112	5.994E-04
AC020558.5	2.612	6.115	1.345E-03
AC091769.1	2.613	6.117	5.292E-05
AC093484.2	2.617	6.136	1.239E-10
TUBB8B	2.624	6.163	5.356E-03
TUBB8	2.629	6.185	5.219E-03
IFITM3P1	2.633	6.201	1.652E-04
AC008764.6	2.637	6.219	0.000E+00
ZDHHC19	2.644	6.250	0.000E+00
RN7SL57P	2.646	6.258	1.595E-04
MIRLET7BHG	2.646	6.260	0.000E+00
H1-4	2.650	6.275	0.000E+00
AC124947.1	2.652	6.284	3.710E-03
AC016735.1	2.653	6.288	1.737E-06
AC099336.1	2.653	6.290	5.167E-06

AC091231.1	2.654	6.292	6.293E-08
RNA5SP149	2.659	6.315	3.669E-03
NKAIN4	2.661	6.326	4.379E-03
RN7SL344P	2.661	6.326	7.989E-04
AP000777.2	2.662	6.329	9.226E-04
AC009065.7	2.666	6.347	1.545E-03
CEP19	2.666	6.348	0.000E+00
GSEC	2.667	6.349	0.000E+00
CMBL	2.670	6.365	5.658E-10
BASP1	2.670	6.366	0.000E+00
IGSF6	2.671	6.371	0.000E+00
AC004490.1	2.672	6.372	6.705E-04
GMPR	2.677	6.393	0.000E+00
AC106801.1	2.678	6.399	8.831E-05
RN7SKP50	2.680	6.407	9.811E-03
PACERR	2.680	6.408	9.667E-07
INSC	2.681	6.413	0.000E+00
AC092068.2	2.681	6.415	9.383E-08
TNFSF14	2.686	6.436	0.000E+00
AP001885.2	2.691	6.458	3.279E-03
AC005606.1	2.692	6.460	3.892E-03
RGL3	2.694	6.469	0.000E+00
AC005306.1	2.694	6.470	0.000E+00
FRMD4A	2.701	6.502	0.000E+00
AC008074.1	2.702	6.507	8.847E-03
TRIM58	2.703	6.512	0.000E+00
AC091196.1	2.703	6.513	0.000E+00
AC022966.1	2.706	6.524	2.603E-03
AC005785.1	2.706	6.526	0.000E+00
AC092384.2	2.707	6.529	1.430E-05
HPD	2.712	6.553	1.488E-07
DGAT2	2.718	6.579	0.000E+00
AC015660.1	2.718	6.582	2.340E-05
AC145098.1	2.720	6.589	5.642E-04
AC106028.3	2.722	6.600	0.000E+00
AC008569.1	2.724	6.605	1.790E-06
AC078856.1	2.729	6.629	1.173E-03
RPL30P7	2.729	6.630	3.806E-06
SLPI	2.729	6.631	1.060E-07
AP000553.8	2.734	6.651	4.018E-07
CCL5	2.738	6.669	5.398E-06
LRFN1	2.738	6.670	0.000E+00
RNA5SP150	2.740	6.679	5.802E-03
AC110769.3	2.740	6.679	1.969E-09
ANKUB1	2.740	6.681	2.060E-04
RN7SL364P	2.740	6.681	0.000E+00
CEBPE	2.742	6.688	0.000E+00
KIF1A	2.743	6.697	8.191E-03
EFCAB1	2.748	6.716	2.939E-03
IDO1	2.748	6.718	0.000E+00
MME	2.757	6.762	4.366E-06
AC080112.3	2.758	6.762	4.571E-03
SPTB	2.759	6.768	0.000E+00
KRT79	2.762	6.782	1.187E-03
AC015871.4	2.762	6.785	0.000E+00

AC097634.1	2.764	6.795	0.000E+00
AC016737.1	2.765	6.795	0.000E+00
AC017083.1	2.765	6.799	0.000E+00
DPEP3	2.766	6.802	0.000E+00
AC008026.3	2.769	6.815	1.941E-10
CFAP92	2.770	6.820	0.000E+00
MIA	2.773	6.834	7.594E-09
HCAR2	2.774	6.839	6.538E-09
NDUFB8P2	2.776	6.851	2.303E-06
AC008395.1	2.778	6.859	8.357E-04
PPIAL4H	2.780	6.870	1.364E-06
HSPA6	2.784	6.885	0.000E+00
ITLN1	2.784	6.887	0.000E+00
ZNF252P-AS1	2.785	6.894	0.000E+00
PHF24	2.788	6.905	1.768E-06
AC073342.1	2.793	6.929	1.703E-06
AC130895.1	2.793	6.930	3.093E-07
FTLP2	2.793	6.931	0.000E+00
UBXN6	2.794	6.938	0.000E+00
AC091117.2	2.803	6.977	5.094E-05
AC009506.1	2.805	6.989	0.000E+00
AC008649.1	2.805	6.991	3.615E-04
SPTA1	2.806	6.994	0.000E+00
AL035685.1	2.808	7.005	1.968E-06
AC012291.3	2.809	7.007	0.000E+00
MKRN4P	2.816	7.040	6.253E-03
BMX	2.816	7.042	0.000E+00
LINC02073	2.822	7.070	1.275E-05
AC018755.5	2.822	7.072	1.667E-06
AC022762.2	2.823	7.075	0.000E+00
AL138921.2	2.827	7.095	8.169E-03
ADGRG3	2.834	7.128	5.167E-10
AC003043.2	2.847	7.193	3.405E-03
VSIG8	2.848	7.201	1.005E-06
RPL21P123	2.850	7.208	0.000E+00
TIGD3	2.853	7.223	0.000E+00
NRXN3	2.853	7.226	4.643E-03
PPIAP4	2.854	7.230	4.119E-06
AC243772.2	2.856	7.241	7.499E-06
LINC00683	2.866	7.289	1.587E-09
AC009501.2	2.867	7.294	1.904E-04
CYP4F3	2.871	7.314	3.213E-09
AL161431.1	2.876	7.342	6.861E-04
AC020917.3	2.880	7.361	4.914E-08
LYL1	2.882	7.369	0.000E+00
CACNA1E	2.886	7.392	4.054E-08
LINC02596	2.890	7.410	1.294E-06
AC005363.2	2.894	7.433	9.731E-06
AL031432.4	2.895	7.436	0.000E+00
AC007192.2	2.896	7.445	2.868E-06
AC097065.2	2.897	7.446	4.911E-03
JSRP1	2.898	7.453	0.000E+00
FAM210B	2.899	7.457	0.000E+00
GALNT14	2.899	7.457	0.000E+00
AC025262.1	2.900	7.463	7.122E-10

SNHG22	2.910	7.517	0.000E+00
AC009145.4	2.914	7.535	8.756E-06
LINC01513	2.921	7.575	1.158E-09
MIR150	2.923	7.582	9.285E-04
LUNAR1	2.925	7.595	1.097E-10
AP000974.1	2.925	7.595	8.258E-07
AS3MT	2.929	7.613	3.658E-10
BX649601.1	2.929	7.615	1.222E-03
AC011498.4	2.940	7.672	1.096E-09
CYP4F10P	2.942	7.683	2.369E-04
AC011498.6	2.949	7.723	0.000E+00
LINC01270	2.949	7.723	0.000E+00
KISS1R	2.951	7.732	0.000E+00
HNRNPA1P52	2.955	7.754	8.330E-10
AC104695.2	2.956	7.758	0.000E+00
AL359510.2	2.960	7.781	0.000E+00
RNU6-646P	2.960	7.781	7.163E-04
AICDA	2.960	7.783	9.222E-09
RNU6-942P	2.968	7.827	7.399E-06
AC025162.2	2.972	7.846	1.525E-10
AC007298.2	2.974	7.859	0.000E+00
LRG1	2.980	7.893	0.000E+00
LCN10	2.982	7.898	0.000E+00
AC120114.2	2.984	7.913	1.316E-06
AC106028.2	2.986	7.921	0.000E+00
TMCC2	2.991	7.952	0.000E+00
AC099343.2	2.992	7.954	0.000E+00
LINC02217	2.996	7.975	1.820E-07
IGKV1D-27	2.996	7.980	6.910E-03
TMEM252-DT	2.999	7.995	7.175E-05
AC002511.1	3.001	8.004	9.858E-09
AC015920.1	3.001	8.005	4.786E-07
AC123912.2	3.002	8.013	8.709E-05
MMP9	3.003	8.014	0.000E+00
RPSAP47	3.005	8.028	1.094E-06
AC131235.2	3.008	8.042	8.782E-05
AC104463.2	3.008	8.042	0.000E+00
MTCO2P33	3.018	8.102	2.718E-05
RNA5SP99	3.029	8.160	1.871E-03
AC074143.1	3.036	8.201	1.335E-08
AC114811.2	3.036	8.203	0.000E+00
MIR338	3.040	8.225	4.843E-03
MIR6124	3.043	8.241	4.344E-07
TMEM252	3.043	8.245	1.134E-04
LYPD1	3.044	8.247	4.599E-04
AP001412.1	3.044	8.248	0.000E+00
AC010287.1	3.044	8.250	8.082E-06
AL627309.2	3.045	8.253	4.135E-07
AC006023.2	3.046	8.260	7.640E-10
AC021424.3	3.047	8.267	2.096E-06
AC113208.2	3.062	8.350	7.429E-06
SCARNA7	3.062	8.350	0.000E+00
LUCAT1	3.065	8.370	0.000E+00
AF129408.1	3.068	8.385	6.522E-09
SNORD7	3.069	8.390	6.038E-09

LSM14A	3.069	8.392	0.000E+00
AP005264.4	3.069	8.394	1.288E-07
AP002907.1	3.071	8.405	0.000E+00
KIF19BP	3.085	8.485	3.385E-04
AL627309.6	3.085	8.486	0.000E+00
KREMEN1	3.086	8.491	0.000E+00
MIR4673	3.089	8.511	9.625E-08
DMRTC2	3.092	8.528	2.063E-08
AC092692.1	3.094	8.540	3.478E-08
AC113208.5	3.098	8.562	0.000E+00
XKR3	3.103	8.592	1.657E-05
BCL2L1	3.105	8.606	0.000E+00
RHCE	3.109	8.626	0.000E+00
AC011446.1	3.109	8.629	8.679E-10
AATK	3.110	8.632	0.000E+00
AC018445.5	3.111	8.639	0.000E+00
MADCAM1	3.116	8.670	0.000E+00
AC012615.2	3.116	8.671	7.315E-08
AP003065.1	3.118	8.683	1.745E-06
AL162151.2	3.118	8.684	0.000E+00
ABALON	3.122	8.706	5.338E-10
AP005432.1	3.124	8.720	0.000E+00
AC012291.2	3.130	8.756	6.391E-08
B3GNT8	3.132	8.768	0.000E+00
LINC01359	3.136	8.792	0.000E+00
AC007271.1	3.137	8.798	4.823E-08
TMOD1	3.137	8.799	0.000E+00
ITLN2	3.141	8.819	2.786E-05
STAM-AS1	3.141	8.820	0.000E+00
PTGES2-AS1	3.146	8.849	0.000E+00
MIR3609	3.146	8.851	2.794E-05
CR1L	3.146	8.855	0.000E+00
AC093484.3	3.147	8.860	0.000E+00
ACTBP8	3.152	8.892	1.403E-03
CMTM2	3.155	8.906	4.054E-10
AOC3	3.158	8.924	0.000E+00
RN7SL521P	3.159	8.933	2.863E-08
CCN3	3.171	9.010	0.000E+00
ARAP1-AS1	3.172	9.012	3.328E-09
TSPEAR	3.172	9.015	4.244E-06
AC004233.2	3.181	9.067	0.000E+00
HCG27	3.183	9.079	1.431E-06
FFAR2	3.183	9.082	1.922E-10
AC006483.2	3.192	9.140	0.000E+00
AC006517.2	3.196	9.162	9.308E-06
LSMEM2	3.201	9.196	3.617E-08
DCAF12	3.202	9.200	0.000E+00
AC006942.1	3.203	9.209	0.000E+00
AOC4P	3.205	9.223	0.000E+00
RNA5SP211	3.206	9.229	2.582E-04
ARL4AP2	3.210	9.256	1.521E-09
IFITM3P2	3.212	9.263	0.000E+00
AC092145.1	3.217	9.296	0.000E+00
GPR146	3.219	9.312	0.000E+00
AC010201.2	3.229	9.374	0.000E+00
AC099489.1	3.236	9.420	0.000E+00
AL357078.2	3.241	9.453	0.000E+00
AC012441.1	3.248	9.498	5.779E-04
AC023787.3	3.250	9.512	1.405E-10
SEPTIN5	3.263	9.600	0.000E+00
AC104389.7	3.264	9.605	4.057E-03
AL391314.1	3.272	9.660	5.846E-07
NRXN1	3.274	9.672	2.257E-10
KCNJ15	3.277	9.695	1.205E-09
AC009682.1	3.279	9.707	7.617E-09
AP006259.1	3.281	9.718	1.884E-09
RNA5SP267	3.285	9.749	2.797E-03
AL732372.3	3.289	9.777	1.041E-03
RPSAP22	3.291	9.787	1.306E-08
AL049651.1	3.291	9.790	3.068E-10
ADORA3	3.295	9.815	0.000E+00
AC004528.2	3.306	9.887	3.382E-08
SHISA4	3.307	9.896	0.000E+00
CA4	3.317	9.965	4.556E-09
KCNS1	3.318	9.971	9.221E-06
LINC02289	3.319	9.983	9.859E-05
YBX3P1	3.326	10.028	2.638E-06
AL391001.1	3.333	10.080	0.000E+00
AL591506.1	3.336	10.096	3.353E-05
AC018607.1	3.340	10.129	0.000E+00
AC087741.3	3.347	10.174	0.000E+00
PDZK1IP1	3.348	10.183	0.000E+00
H3P16	3.355	10.234	0.000E+00
AC007298.1	3.356	10.236	0.000E+00
FAM169B	3.356	10.240	3.252E-10
IL34	3.365	10.305	0.000E+00
ACHE	3.374	10.365	0.000E+00
AC107959.4	3.375	10.373	0.000E+00
BTNL8	3.377	10.387	1.051E-07
MIR4802	3.378	10.397	1.231E-04
C17orf99	3.389	10.473	0.000E+00
TMEM92-AS1	3.404	10.584	0.000E+00
AC126118.1	3.417	10.681	0.000E+00
CORIN	3.423	10.729	1.402E-09
HNRNPA3P2	3.426	10.747	0.000E+00
AC000093.1	3.438	10.838	0.000E+00
TMC5	3.440	10.854	0.000E+00
LINC01331	3.441	10.862	2.619E-07
H4C4	3.443	10.873	0.000E+00
MIR657	3.452	10.944	0.000E+00
AC108704.1	3.455	10.964	0.000E+00
AC012645.1	3.465	11.040	0.000E+00
CHI3L1	3.466	11.049	6.519E-10
SLC25A37	3.468	11.062	0.000E+00
AC124319.3	3.468	11.063	0.000E+00
TNS1	3.475	11.121	0.000E+00
SCARNA20	3.479	11.149	0.000E+00
AC125603.4	3.481	11.163	0.000E+00
LINC01887	3.481	11.166	2.475E-10
AC007686.2	3.493	11.262	2.742E-07

AC096970.1	3.501	11.321	5.003E-09
SNORA11B	3.505	11.355	1.891E-04
CXCR2	3.506	11.357	2.883E-09
AC092171.4	3.527	11.527	0.000E+00
AP002381.2	3.540	11.628	0.000E+00
AC092794.2	3.543	11.659	9.996E-09
ENTPD2	3.544	11.665	0.000E+00
STRADB	3.552	11.729	0.000E+00
AL589765.6	3.564	11.830	0.000E+00
LINC00958	3.578	11.944	3.868E-04
KAZN	3.580	11.959	0.000E+00
NECAB2	3.586	12.008	0.000E+00
AC004491.1	3.588	12.024	0.000E+00
AC087741.2	3.589	12.031	0.000E+00
RNY1	3.606	12.180	9.383E-06
AL009031.1	3.623	12.316	2.217E-07
KRT77	3.629	12.369	2.817E-03
AL354877.1	3.641	12.478	0.000E+00
AC010201.1	3.647	12.528	0.000E+00
TNFRSF10C	3.652	12.574	0.000E+00
APOBEC2	3.653	12.582	7.600E-06
SLC6A9	3.655	12.598	0.000E+00
PANX2	3.657	12.614	0.000E+00
PLIN5	3.657	12.616	0.000E+00
RNU6-759P	3.661	12.650	3.312E-10
AC005387.1	3.663	12.667	3.600E-03
MED6P1	3.682	12.831	3.466E-08
CXCR1	3.683	12.846	9.113E-10
AC007686.4	3.691	12.916	0.000E+00
SNCA	3.709	13.074	0.000E+00
AC074132.1	3.717	13.149	0.000E+00
PEAK3	3.725	13.227	0.000E+00
MTND6P11	3.726	13.229	8.586E-10
AL513320.1	3.730	13.269	0.000E+00
AL356968.2	3.738	13.345	0.000E+00
PRSS41	3.744	13.402	0.000E+00
ALPL	3.757	13.520	2.554E-08
SEMG1	3.758	13.527	0.000E+00
RMRP	3.760	13.547	1.127E-07
AC011558.1	3.760	13.550	3.471E-10
PLIN4	3.762	13.565	0.000E+00
TAF11L2	3.768	13.626	7.610E-08
AC138827.2	3.792	13.855	0.000E+00
AC145207.2	3.822	14.139	0.000E+00
AL137792.1	3.836	14.281	0.000E+00
NFE4	3.852	14.436	5.294E-10
RHAG	3.856	14.477	0.000E+00
AL109945.1	3.858	14.497	2.321E-06
AC015802.6	3.860	14.524	0.000E+00
IGKV1-8	3.865	14.576	4.978E-05
AC009148.1	3.873	14.648	0.000E+00
MMP25	3.874	14.663	0.000E+00
MYBPH	3.880	14.727	0.000E+00
AC079316.1	3.884	14.768	0.000E+00
DMTN	3.886	14.784	0.000E+00
OR51AB1P	3.894	14.871	9.334E-05
AC002511.2	3.942	15.365	0.000E+00
SOX6	3.951	15.460	0.000E+00
AC123595.1	3.951	15.470	0.000E+00
CEACAM3	3.973	15.706	0.000E+00
PNMA6A	3.983	15.810	0.000E+00
AP003498.2	3.984	15.824	1.551E-06
ATP6V0CP4	3.989	15.883	5.401E-09
KRT1	3.993	15.927	0.000E+00
AC022167.3	3.998	15.979	0.000E+00
AC107959.3	4.007	16.080	0.000E+00
AOC1	4.011	16.121	4.422E-10
OSBP2	4.033	16.375	0.000E+00
GALNT5	4.041	16.461	3.077E-10
LINC01342	4.047	16.532	0.000E+00
RNU6-1176P	4.067	16.756	0.000E+00
PAGE2B	4.085	16.971	0.000E+00
RN7SL1	4.103	17.184	0.000E+00
AC005622.1	4.106	17.222	0.000E+00
RNU6ATAC39P	4.117	17.350	0.000E+00
AC100835.2	4.145	17.689	0.000E+00
SMIM24	4.146	17.705	0.000E+00
AD000090.1	4.148	17.727	0.000E+00
GATA1	4.162	17.897	0.000E+00
AP001107.2	4.171	18.010	0.000E+00
GYPC	4.176	18.078	0.000E+00
CA1	4.180	18.123	0.000E+00
AL008582.1	4.181	18.145	0.000E+00
GYPA	4.192	18.279	0.000E+00
FKBP8	4.215	18.571	0.000E+00
OTX1	4.219	18.628	1.531E-08
CSF2RBP1	4.236	18.848	3.814E-10
AC073172.1	4.247	18.985	4.215E-09
HEPACAM2	4.260	19.162	0.000E+00
ESPN	4.329	20.095	0.000E+00
AC007342.2	4.354	20.445	0.000E+00
SLC25A39	4.387	20.925	0.000E+00
CLDN9	4.399	21.095	0.000E+00
PAQR9	4.457	21.968	0.000E+00
OR2W3	4.557	23.541	0.000E+00
PLEK2	4.576	23.845	0.000E+00
SLC6A8	4.581	23.941	0.000E+00
RNF182	4.596	24.176	6.514E-08
SEC14L4	4.602	24.286	0.000E+00
LINC02772	4.609	24.398	0.000E+00
TSPO2	4.669	25.448	0.000E+00
LMOD1	4.670	25.462	0.000E+00
BICDL2	4.721	26.381	0.000E+00
BTNL3	4.722	26.394	5.118E-05
CAMK2A	4.733	26.593	0.000E+00
CTSE	4.750	26.914	0.000E+00
RNA5SP259	4.792	27.694	2.483E-08
PHOSPHO1	4.803	27.909	0.000E+00
IFIT1B	4.825	28.339	0.000E+00
Z98886.1	4.846	28.765	0.000E+00

AC093599.2	4.905	29.951	0.000E+00
AC012645.2	4.946	30.828	0.000E+00
KLHDC8A	4.962	31.173	0.000E+00
BCAM	5.000	31.999	0.000E+00
HBD	5.034	32.766	0.000E+00
SGIP1	5.035	32.777	0.000E+00
ALOX15	5.058	33.318	0.000E+00
ALAS2	5.059	33.340	0.000E+00
AC012645.4	5.091	34.094	0.000E+00
SELENBP1	5.098	34.260	0.000E+00
HBG2	5.107	34.461	0.000E+00
AHSP	5.158	35.711	0.000E+00
TMPRSS9	5.160	35.746	0.000E+00
RNA5SP225	5.209	36.994	1.270E-08
RNA5S17	5.221	37.301	1.714E-10
AC073210.3	5.259	38.306	0.000E+00
SLC4A1	5.261	38.341	0.000E+00
RN7SL4P	5.288	39.082	0.000E+00
PRSS33	5.341	40.531	0.000E+00
OLIG2	5.353	40.872	0.000E+00
RAP1GAP	5.363	41.168	0.000E+00
SIGLEC8	5.389	41.913	0.000E+00
KLF1	5.390	41.942	0.000E+00
EPB42	5.411	42.561	0.000E+00
AC104389.6	5.425	42.977	0.000E+00
AL928646.1	5.427	43.009	0.000E+00
ABCC13	5.427	43.011	0.000E+00
VWCE	5.452	43.768	0.000E+00
SMIM1	5.480	44.645	0.000E+00
SHISA7	5.543	46.636	0.000E+00
AC006205.2	5.587	48.073	0.000E+00
LINC00570	5.651	50.263	0.000E+00
AC011472.1	5.676	51.111	0.000E+00
ESRG	5.728	52.992	0.000E+00
AC104561.3	5.743	53.572	0.000E+00
RNA5SP242	5.796	55.575	0.000E+00
HBQ1	5.800	55.729	0.000E+00
GYPB	5.878	58.808	0.000E+00
MYL4	5.910	60.112	0.000E+00
ACKR1	5.932	61.052	0.000E+00
RAB3IL1	5.951	61.871	0.000E+00
SFRP2	5.995	63.769	0.000E+00
AL139220.2	6.057	66.586	0.000E+00
HBZ	6.160	71.531	0.000E+00
RUNDC3A	6.326	80.222	0.000E+00
HBB	6.462	88.142	0.000E+00
HBM	6.585	96.008	0.000E+00
HBA2	6.737	106.664	0.000E+00
KLC3	6.816	112.643	0.000E+00
HBA1	6.862	116.286	0.000E+00
AC092490.1	6.991	127.243	0.000E+00
RNA5SP226	7.148	141.814	0.000E+00
AC130456.3	7.305	158.135	0.000E+00
